# Synthetic Ionizable Colloidal Drug Aggregates Enable Endosomal Disruption

**DOI:** 10.1002/advs.202300311

**Published:** 2023-03-11

**Authors:** Eric N. Donders, Kai V. Slaughter, Christian Dank, Ahil N. Ganesh, Brian K. Shoichet, Mark Lautens, Molly S. Shoichet

**Affiliations:** ^1^ Department of Chemical Engineering & Applied Chemistry University of Toronto 200 College Street Toronto ON M5S 3E5 Canada; ^2^ Institute of Biomedical Engineering University of Toronto 164 College Street Toronto ON M5S 3G9 Canada; ^3^ Donnelly Centre University of Toronto 160 College Street Toronto ON M5S3E1 Canada; ^4^ Department of Chemistry University of Toronto 80 St. George Street Toronto ON M5S 3H6 Canada; ^5^ Department of Pharmaceutical Chemistry University of California San Francisco 1700 Fourth Street, Mail Box 2550 San Francisco CA 94143 USA

**Keywords:** colloidal drug aggregates, drug delivery, endosomal disruption, fulvestrant, nanoparticles

## Abstract

Colloidal drug aggregates enable the design of drug‐rich nanoparticles; however, the efficacy of stabilized colloidal drug aggregates is limited by entrapment in the endo‐lysosomal pathway. Although ionizable drugs are used to elicit lysosomal escape, this approach is hindered by toxicity associated with phospholipidosis. It is hypothesized that tuning the p*K*_a_ of the drug would enable endosomal disruption while avoiding phospholipidosis and minimizing toxicity. To test this idea, 12 analogs of the nonionizable colloidal drug fulvestrant are synthesized with ionizable groups to enable pH‐dependent endosomal disruption while maintaining bioactivity. Lipid‐stabilized fulvestrant analog colloids are endocytosed by cancer cells, and the p*K*_a_ of these ionizable colloids influenced the mechanism of endosomal and lysosomal disruption. Four fulvestrant analogs—those with p*K*_a_ values between 5.1 and 5.7—disrupted endo‐lysosomes without measurable phospholipidosis. Thus, by manipulating the p*K*_a_ of colloid‐forming drugs, a tunable and generalizable strategy for endosomal disruption is established.

## Graphical abstract

Ionizable drug analogs induce endo‐lysosomal disruption. Chemical modification of fulvestrant, a drug that forms colloidal aggregates, results in ionizable analogs. Colloids of analogs with p*K*_a_ values between 5.1 and 5.7 enable endo‐lysosomal disruption without causing phospholipidosis, thereby avoiding a potentially harmful side effect. This strategy can be useful when tuning drug structure during development.

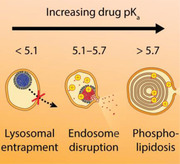

## 1 Introduction

Many small molecule drugs form amorphous aggregates in aqueous media, resulting in anomalous experimental results.^[^ ^1^ ^]^ These colloidal drug aggregates can cause false‐positive hits in enzyme inhibition assays due to promiscuous enzyme adsorption to the colloids.^[^ ^2^, ^3^, ^4^, ^5^ ^]^ They can also cause false‐negative results in cell‐based assays because the colloids are too large to enter cells.^[^ ^6^, ^7^ ^]^ To prevent the formation of colloids, solubilizers are often used in conventional formulations, but these excipients are frequently dose‐limiting due to side effects.^[^ ^8^, ^9^ ^]^

The drug‐rich nature of colloidal drug aggregates may be exploited to improve drug efficacy, but critical obstacles remain. For example, the typical instability of colloidal drug aggregates has been overcome by coformulation and macromolecule adsorption,^[^ ^10^, ^11^, ^12^, ^13^, ^14^ ^]^ while limited cell uptake has been overcome by modifying the colloid surface with targeting proteins, resulting in receptor‐mediated endocytosis, as demonstrated with lipid fluorophore dye‐labeled colloids.^[^ ^15^, ^16^ ^]^ However, escape from the endo‐lysosomal pathway remains a key challenge for drug efficacy.^[^ ^16^ ^]^

Ionizable lipids and polymers have been shown to facilitate disruption of endo‐lysosomal membranes.^[^ ^17^, ^18^, ^19^ ^]^ These species are neutral at physiological pH but cationic in the acidic milieu of endosomes and lysosomes, where they cause endosomal disruption through varied mechanisms.^[^ ^18^, ^20^, ^21^, ^22^, ^23^ ^]^ Most researchers design vehicles that disrupt endosomes because the harsh environment of the lysosomes can degrade sensitive cargoes.^[^ ^24^ ^]^

More recently, endo‐lysosomal disruption has been achieved with ionizable small molecule drugs. For example, ionizable drug adjuvants have improved the cytosolic delivery of nucleic acid and protein drugs.^[^ ^25^, ^26^, ^27^, ^28^ ^]^ The mechanism for this behavior is poorly understood, but it is thought to be driven by the accumulation of individual cationic drug molecules in the lysosomes. The drugs disrupt lipid homeostasis, leading to phospholipidosis—a condition marked by the accumulation of lipids within the lysosomes.^[^ ^29^, ^30^ ^]^ Phospholipidosis often co‐occurs with permeabilization of the lysosomal membrane and leakage of lysosomal contents into the cytosol.^[^ ^31^ ^]^ Although drugs may be released this way, they are accompanied by apoptosis‐inducing enzymes.^[^ ^24^ ^]^ Phospholipidosis and its related toxic effects also pose safety concerns in vivo.^[^ ^32^ ^]^

We previously showed that ionizable drug colloids can induce endo‐lysosomal escape: colloids containing the drug lapatinib become positively charged in the endo‐lysosomal pathway, leading to membrane disruption and subsequent drug release into the cytoplasm.^[^ ^15^ ^]^ However, this strategy is limited to inherently ionizable drugs at the appropriate pH.

**Figure 1:**
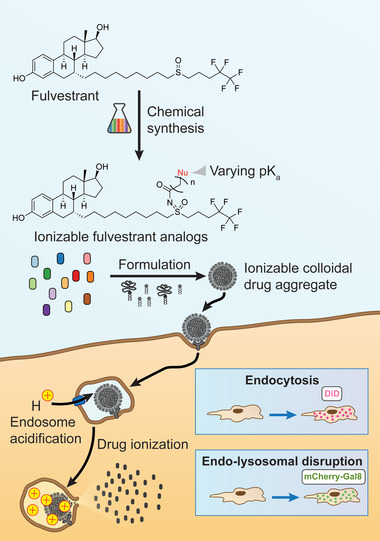
Ionizable fulvestrant analogs induce endo‐lysosomal disruption. Fulvestrant, a drug that forms colloidal aggregates, was chemically modified with functional groups, resulting in ionizable analogs with a range of p*K*_a_ values. Colloid endocytosis was assessed using formulations containing a fluorescent dye (DiD). The endo‐lysosomal disruption caused by ionization‐mediated mechanisms was measured using cells with a fluorescent reporter (mCherry‐Gal8).

We investigated a tunable strategy to facilitate endo‐lysosomal disruption with colloid‐forming drugs since not all drugs are ionizable at endosomal pH and the optimal p*K*_a_ for membrane disruption is unknown (**Figure** **1**). Importantly, we aimed to achieve endo‐lysosomal disruption without the drawbacks associated with phospholipidosis and lysosomal membrane permeabilization.

We hypothesized that the p*K*_a_ of colloidal drug aggregates would affect endo‐lysosomal disruption. To test this hypothesis, we first synthesized a series of ionizable fulvestrant analogs, each bearing a different ionizable group. Fulvestrant is a relevant model drug as it is used clinically to treat breast cancer, yet it readily forms colloidal aggregates^[^ ^10^, ^11^ ^]^ that can lose their potency^[^ ^6^, ^7^ ^]^ by becoming trapped in lysosomes.^[^ ^16^ ^]^ Interestingly, fulvestrant has a reactive functional group separate from the active site that is available for covalent modification.^[^ ^33^ ^]^ As fulvestrant binds to the estrogen receptor with the 4‐ring steroid,^[^ ^34^ ^]^ modification of the tail region should not affect potency.^[^ ^35^ ^]^ Thus, we first synthesized a sulfoximine analog and then incorporated different amine‐containing functional groups through acylation of the sulfoximine.

We show, for the first time, how to turn the nuisance typically associated with colloidal drug aggregates into an asset with bioactive, ionizable, fulvestrant analog colloidal drug aggregates that disrupt endo‐lysosomes. We demonstrate that the p*K*_a_ of the fulvestrant analog affects the disruption efficiency and mechanism, thereby providing insight for the design of future colloidal aggregates.

## 2 Results and Discussion

### 2.1 Amine‐Containing Fulvestrant Analogs are Ionizable

**Figure 2:**
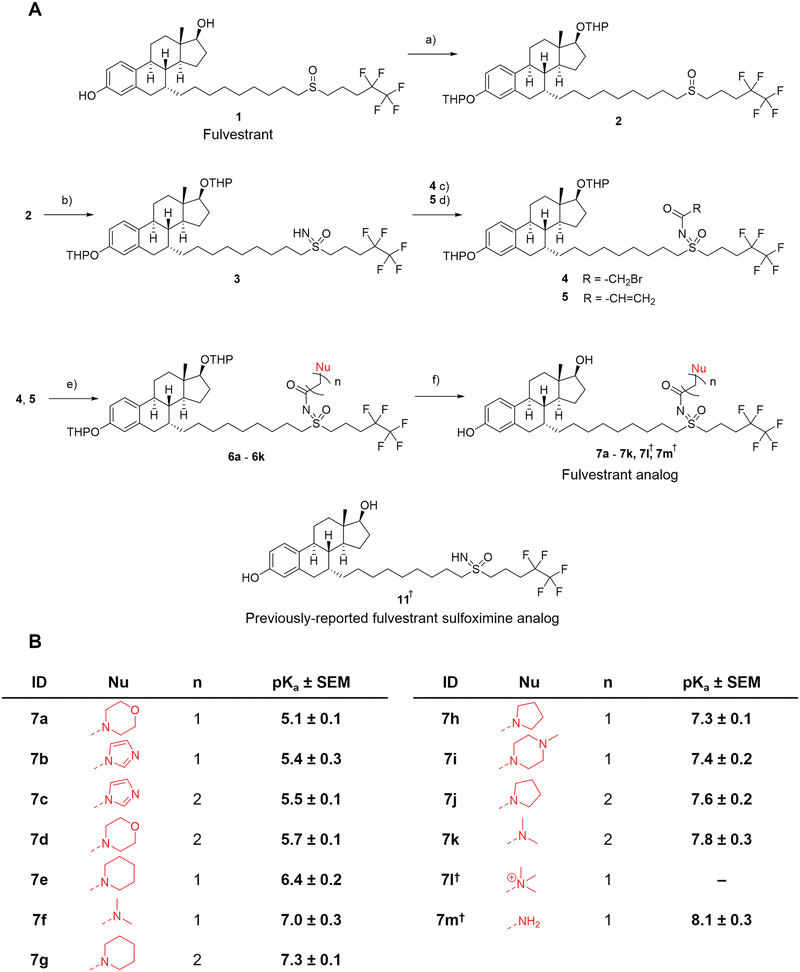
Chemical modification of fulvestrant with amines results in ionizable analogs. A) Synthetic scheme for the synthesis of ionizable fulvestrant analogs. a) Dihydropyran (3 equiv), trifluoroacetic acid (TFA, 20 mol%), dichloromethane (DCM), 22 °C, 3 d. b) (diacetoxyiodo)benzene (3 equiv), ammonium carbamate (4 equiv), *N*,*N*‐diisopropylethylamine (DIPEA, 0.1 equiv), methanol, 22 °C, 4 h. c) Bromoacetyl bromide (2 equiv), DIPEA (5 equiv), DCM, 0 °C → 22 °C, 30 min. d) 3‐bromopropanoyl chloride (2 equiv), DIPEA (5 equiv), DCM, 0 °C → 22 °C, 1 h. e) NuH (10 equiv) or NuH·HCl (50 equiv) and triethylamine (100 equiv), 22 °C, 4 h. f) 2% (v v^−1^) TFA in methanol, 22 °C, 24 h. B) Tabulated identifiers, structures, and p*K*_a_ values of the synthesized ionizable fulvestrant analogs. † **7l**, **7m**, and **11** were synthesized using different routes, as shown in Scheme S1 (Supporting Information) (**7l**) and Scheme S2 (Supporting Information) (**7m** and **11**).

Fulvestrant was modified by a series of reactions of the sulfoxide group to introduce ionizable amines with varying p*K*_a_ values while ensuring that the steroid warhead was unaffected (**Figure** **2**A). We started by protecting the fulvestrant hydroxyl groups as tetrahydropyran (THP) ethers to form intermediate **2**. We imiated **2** by a previously reported reaction^[^ ^33^ ^]^ to yield **3**. Next, we acylated intermediate **3** with either bromoacetyl bromide or 3‐bromopropanoyl chloride to yield intermediates **4** and **5**, respectively. These intermediates contain electrophilic sites at the *α*‐carbon (**4**) or *β*‐carbon (**5**) of the amide. Next, we reacted intermediates **4** and **5** with different nucleophiles to yield **6a,b,e,f,h,i,l** and **6c,d,g,j,k**, respectively. In the final step of the synthesis, we removed the THP ether protecting groups with acidic methanol, yielding fulvestrant analogs **7a–7l**. We synthesized the primary amine **7m,** along with the previously‐reported unmodified sulfoximine **11**, using an alternative synthetic pathway (Scheme S2, Supporting Information).

Our synthetic scheme builds from the fulvestrant sulfoximine analog with these new amino derivatives. Furthermore, we demonstrate that tetrahydropyranyl (THP) ethers are stable under the imination conditions and can be removed without impacting the sulfoximine. These findings further our understanding of *N*‐acyl sulfoximines, which have been sparsely studied despite the utility of sulfoximines in medicinal chemistry.^[^ ^36^ ^]^

Next, we evaluated the p*K*_a_ of each of the fulvestrant analogs using a method that has been described for ionizable amino lipids.^[^ ^17^, ^37^ ^]^ 2‐(*p*‐toluidino) naphthalene‐6‐sulfonic acid (TNS), an environment‐sensitive fluorophore, fluoresces in the presence of hydrophobic cations, such as the protonated forms of our ionizable fulvestrant analogs. We measured TNS fluorescence between pH 3 and 10 in the presence of fulvestrant, **7a–7m**, or **11** (Figure S1, Supporting Information) and calculated p*K*_a_ values from these curves. Fulvestrant was not protonated at any pH studied, which is consistent with its lack of a basic nitrogen atom. Analogs **7a–7k** and **7m** had p*K*_a_ values between 5.1 and 8.1 (Figure 2B), most of which fall between the pH of the extracellular space (7.4) and that of the lysosome (4.5–5).^[^ ^38^ ^]^ Decreasing the length of the carbon spacer between the ionizable nitrogen and carbonyl group resulted in a lower p*K*_a_, likely due to the electron‐withdrawing effect of the carbonyl group, which makes the nitrogen less electron‐rich and thus less basic. Surprisingly, analog **7l** exhibited a p*K*_a_ of 10.5 despite the quaternary ammonium nitrogen bearing a permanent positive charge. We hypothesize that this p*K*_a_ corresponds to the deprotonation of the phenolic hydroxyl group at high pH values, resulting in a zwitterion that does not activate the fluorescence of TNS, unlike the cation that predominates at lower pH values. The unmodified sulfoximine analog **11** became protonated below pH 4, which suggests that the sulfoximine nitrogen is weakly basic; however, this pH value is lower than that typically found in the endo‐lysosomal pathway, so **11** is unlikely to ionize in cells.

We then tested our hypothesis that ionizable fulvestrant analogs would be more soluble at the acidic pH of the endo‐lysosomal pathway by measuring the critical aggregation concentration (CAC) of representative analogs at a range of pH values. We found that the CAC of ionizable analogs **7a**, **7b**, **7f**, and **7m** increased as the pH was reduced from 7.4 to 4.5, whereas the CAC of nonionizable fulvestrant was unchanged (Figure S2, Supporting Information). This behavior is similar to other ionizable colloid‐forming drugs, which have been shown to rapidly release from stable colloids under acidic conditions.^[^ ^15^ ^]^ Overall, these results confirm the acid‐responsiveness of our ionizable fulvestrant analogs.

### 2.2 Stable Colloidal Drug Aggregates are Efficiently Endocytosed

Using fulvestrant as a proxy for the analogs, we tested how combinations of small amounts of stabilizing excipients affected colloid stability and endocytosis. We first formulated fulvestrant with one of two phospholipids—distearoylphosphatidylcholine (DSPC) or dilaurylphosphatidylcholine—in combination with one of two other surfactants—1,2‐dimyristoyl‐rac‐glycero‐3‐methoxypolyethylene glycol‐2000 (DMG‐PEG‐2000) or polysorbate 80 (Figure S3, Supporting Information). These formulations were prepared in phosphate‐buffered saline (PBS) and incubated at 37 °C between measurements. We also added the lipid dye 1,1“‐dioctadecyl‐3,3,3”,3'‐tetramethylindodicarbocyanine (DiD), which is typically used to label lipid membranes^[^ ^39^ ^]^ but does not readily transfer between lipid compartments,^[^ ^40^ ^]^ to facilitate imaging of the colloids. The initial diameters of dye‐labeled colloids, which remained between 100 and 150 nm, increased to ≈200 nm over 24 h (Figure S4A, Supporting Information). Importantly, their polydispersity index values remained below 0.2 (Figure S4B, Supporting Information), indicating a lack of flocculation; in addition, the scattering intensity of the colloids did not decrease over time, further supporting the lack of precipitation (Figure S4C, Supporting Information). Drug loadings varied from 68% to 82%, with the DMG‐PEG‐2000 formulations exhibiting the highest loading (Figure S4D, Supporting Information), highlighting the drug‐rich nature of the colloids. These values are significantly higher than traditional nanoparticle formulations, which rarely exceed 10%.^[^ ^41^ ^]^ We next evaluated endocytosis by treating cells with these DiD‐labeled colloids, imaging with a wide‐field fluorescence microscope, and counting the number of colloid‐containing vesicles (DiD puncta) per cell. Interestingly, fulvestrant colloids stabilized with DSPC and DMG‐PEG‐2000 were endocytosed to the greatest extent, as observed in SKOV3 cells in vitro (Figure S5, Supporting Information). We reasoned that endocytosis, rather than simple surface binding, occurred because the colloids occupied the same z‐plane as the nuclei and were concentrated around the nucleus rather than uniformly distributed over the whole cell (Figure S6, Supporting Information). Given the relative size of the colloids (approx. 150 nm) and endosomes (at most 400 nm with multiple compartments),^[^ ^42^ ^]^ it is unlikely that many colloids would reside within the same endosome. However, lysosomes may appear as a single punctum yet contain multiple colloids, so we approximate colloid uptake by the number of DiD puncta.

**Figure 3:**
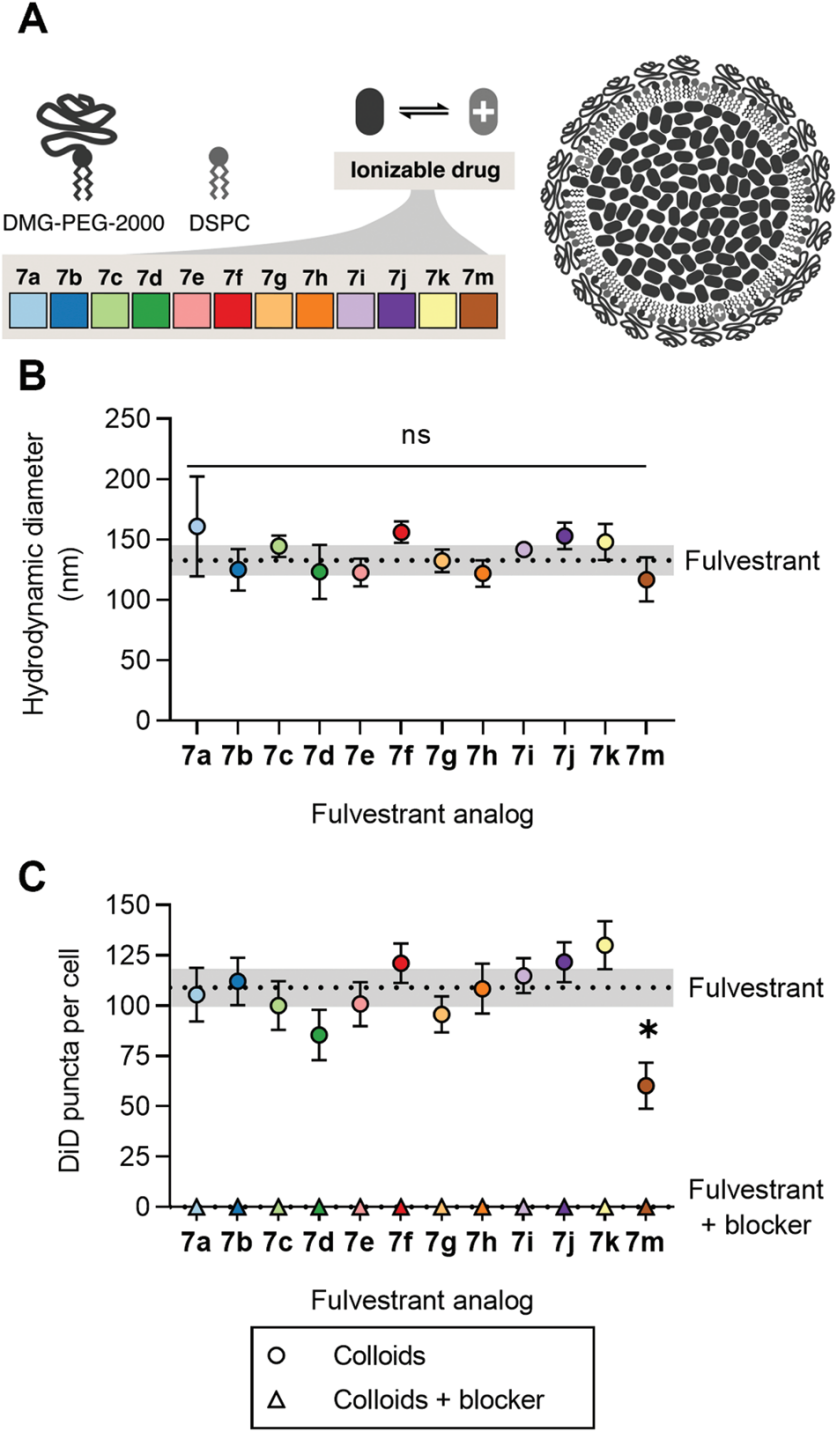
Surfactant excipients stabilize fulvestrant analog colloids and facilitate uptake into cells. A) Diagram depicting the ionizable colloidal formulations and their components. B) Hydrodynamic diameter of fulvestrant analog colloidal drug aggregates immediately after formulation with DSPC, DMG‐PEG 2000, and DiD (*n* ≥ 3, mean ± SD, two‐way ANOVA with Tukey's posthoc test comparing all groups to fulvestrant, ns *p* ≥ 0.05). C) Number of DiD puncta (colloid‐containing vesicles) per cell after 3 h of incubation with 5 µm of colloidal fulvestrant analog (*n* ≥ 12, mean ± SEM, Brown–Forsythe and Welch ANOVA with Dunnett T3 post‐hoc tests comparing each group to fulvestrant with or without dynamin‐mediated endocytosis blocker, *p < 0.05). No colloids were observed in the cells when endocytosis was blocked with 20 µm hydroxydynasore.

Bare colloids of most fulvestrant analogs flocculated in PBS (Figure S7, Supporting Information), but coformulation with DSPC and DMG‐PEG‐2000 resulted in stabilized colloids (**Figure** **3**A) that had diameters between 100 and 150 nm (Figure 3B). The p*K*_a_ values of these stabilized fulvestrant analogs were similar to measurements of the drug alone (Figure S8, Supporting Information), demonstrating that the formulated fulvestrant analogs remain ionizable. Colloids of fulvestrant analogs with higher p*K*_a_ values tended to have higher zeta potentials (Figure S9, Supporting Information), which may reflect their higher average charge at physiological pH or enrichment in the stabilizer coating. We used DiD dye‐labeled colloids of analogs **7a**–**7m** to measure endocytosis of the colloidal fulvestrant analogs, again by counting the number of DiD puncta per cell. We found levels of endocytosis similar to that of fulvestrant alone for all except **7m**.

We hypothesized that colloid uptake proceeded through receptor‐mediated endocytosis driven by adsorbed proteins, such as apolipoprotein E (ApoE), which has been shown to facilitate the uptake of other lipid‐stabilized nanoparticles.^[^ ^43^, ^44^, ^45^ ^]^ To confirm this mechanism, we first added hydroxydynasore, a blocker of dynamin‐mediated endocytosis pathways^[^ ^46^, ^47^ ^]^ and found no evidence of colloids of either analogs (Figure 3C) or fulvestrant/analog combinations (Figure S10, Supporting Information) in the cells. Furthermore, hydroxydynasore did not meaningfully change the number of cells observed in each image (Figure S11, Supporting Information), suggesting no acute toxicity. These results are consistent with experiments in the literature demonstrating complete abrogation of dynamin‐mediated endocytosis with nontoxic concentrations of hydroxydynasore.^[^ ^47^ ^]^ Furthermore, they support our hypothesis that the colloids were endocytosed rather than bound to the cell surface because dynamin is not required for surface binding. To test the specific effect of ApoE, we treated cells with colloids diluted into media containing different combinations of FBS and ApoE (Figure S12, Supporting Information). We found that ApoE improved endocytosis, with statistical significance observed for fulvestrant and **7f**. However, we found that FBS resulted in greater uptake than ApoE for the other analogs tested (**7d** and **7** **g**) . Thus, other serum components must also contribute to endocytosis. This result is corroborated by recent findings that some lipid nanoparticles can be endocytosed by mechanisms that are independent of classical lipoprotein trafficking pathways.^[^ ^48^ ^]^

### 2.3 Fulvestrant Analog Colloids are Biologically Active

When we designed the fulvestrant analogs, we were careful to avoid modification near the steroid warhead of the drug. Fulvestrant is an antagonist of the estrogen receptor, which nominally resides in the cytosol.^[^ ^35^ ^]^ To test activity, we examined whether the growth of estrogen receptor‐expressing (ER+) MCF7 breast cancer cells was inhibited by both fulvestrant and our fulvestrant analogs in a concentration‐dependent manner (Figure S13, Supporting Information). Fulvestrant, ionizable analogs **7a–7k**, **7m**, and sulfoximine analog **11** all have low nanomolar IC_50_ values (Figure S14, Supporting Information), consistent with previous studies on MCF7 growth inhibition by fulvestrant.^[^ ^33^, ^49^, ^50^ ^]^ The anti‐estrogen activity of fulvestrant is maintained in the analogs that have tail region sulfoxide modified. Two ionizable analogs (**7d** and **7m**), and one non‐ionizable analog, **7l**, were significantly less potent than fulvestrant, with a twofold and sevenfold increase in IC_50,_ respectively. Although the tail region of fulvestrant is thought to tolerate some modifications, the reduction in potency of analogs **7d** and **7m** could nevertheless be explained by variations in estrogen receptor binding affinity between analogs. The permanent cationic nature of **7l** may impede its diffusion into cells^[^ ^51^ ^]^ and thereby account for its greater IC_50_. Taken together, these results add nuance to previous work showing that modifications to the tail of fulvestrant have little impact on its potency.^[^ ^33^, ^35^ ^]^

As most ionizable fulvestrant analogs remained potent against their molecular target, we wondered whether their colloidal formulations would show the same efficacy. We observed complex concentration‐response relationships following the treatment of another ER+ breast cancer cell line, BT474, with colloids (Figure S15, Supporting Information). At sub‐micromolar (i.e., soluble) drug concentrations, the formulations slowed the growth of these cancer cells, which is consistent with the antiestrogen activity of fulvestrant.^[^ ^34^, ^52^ ^]^ However, at micromolar drug concentrations, the formulations were cytotoxic. Ionizable fulvestrant analogs with the highest p*K*_a_ values were generally the most toxic (Figure S16, Supporting Information).

Although the mechanism of action of fulvestrant—suppressing proliferation through degradation of the estrogen receptor—has been well studied, it is possible that the MCF7 and BT474 growth inhibition that we observed was due to a different, nonspecific mechanism. Thus, we treated estrogen receptor‐negative healthy lung fibroblasts with colloidal fulvestrant analogs and measured their growth. Unlike ER+ MCF7 and BT474 cells, the growth of these fibroblasts was unaffected by sub‐micromolar concentrations of fulvestrant or its analogs (Figure S17, Supporting Information). This experiment also allowed us to probe toxicity in healthy cells. We found that fulvestrant analogs **7a**–**7d** exhibited similar toxicity to fulvestrant, whereas the higher p*K*_a_ analogs (**7e**–**7k** and **7m**) were more toxic (Figure S18, Supporting Information), likely due to phospholipidosis, as discussed more thoroughly in the discussion. Overall, these experiments demonstrate that our ionizable fulvestrant analogs are biologically active and selective for ER+ cancer cells.

### 2.4 Colloidal Fulvestrant Analogs Cause Endo‐Lysosomal Disruption

**Figure 4:**
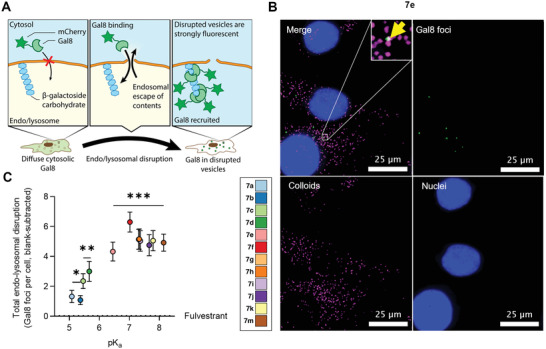
Ionizable fulvestrant analog colloids disrupt endo‐lysosomes. A) Graphical representation of the fluorescent endosomal and lysosome disruption reporter used for assessing endo‐lysosomal disruption. A constitutively expressed mCherry and galectin 8 (Gal8) fusion is recruited to disrupted endosomes and lysosomes, resulting in spots (foci) when viewed by fluorescence microscopy. B) Representative fluorescence images obtained after treating ovarian SKOV3 reporter cancer cells with colloids of ionizable fulvestrant analog **7e** showing robust uptake of colloids (magenta DiD puncta) and endosome disruption (Gal8 foci, green). The yellow arrow highlights an overlap between a colloid‐containing vesicle and a disrupted endosome. These images have been processed to remove background and diffuse Gal8 fluorescence. C) Quantification of Gal8 foci per cell. Cells treated with fulvestrant analog colloids had more disrupted endosomes and lysosomes than cells treated with fulvestrant colloids (*n* ≥ 12 biological replicates, Brown–Forsythe and Welch ANOVA tests with Dunnett T3 post hoc test, *p < 0.05, **p < 0.01, ***p < 0.001 relative to fulvestrant).

We next investigated whether stabilized, endocytosed colloids of ionizable fulvestrant analogs disrupted endo‐lysosomal membranes. We used a fluorescent galectin 8 (Gal8) reporter system^[^ ^19^, ^25^, ^26^ ^]^ to visualize the endosomal disruption with colloids as galectins are recruited to damaged endosomes and lysosomes (**Figure** **4**A). Gal8 binds to *β*‐galactoside carbohydrates on the inside of endosomal and lysosomal membranes that are only accessible when the membranes of these vesicles are disrupted. In contrast, other endosomal disruption indicators, such as dextran, fluorescein, or protein fragments, change their intracellular distribution following membrane disruption,^[^ ^53^ ^]^ but the signals are difficult to quantify. Other methods, such as colocalization analysis, Förster resonance energy transfer (FRET), and electron microscopy, quantify the number of endosomal disruption events;^[^ ^53^ ^]^ however, it is often difficult to distinguish real events from imaging artifacts due to background noise. In contrast, galectin recruitment assays are quantitative, unbiased, and shown to predict the endosomal escape of functional drugs.^[^ ^19^, ^54^ ^]^ We transduced the ovarian SKOV3 cancer cell line with an mCherry‐Gal8 reporter (Figure S19, Supporting Information) to investigate endo‐lysosomal disruption of fulvestrant analog colloids as these cells were previously shown to trap fulvestrant colloids within the endo‐lysosomal pathway.^[^ ^16^ ^]^ We then used a MATLAB script to remove the diffuse fluorescence in the cells and segment the nuclei, mCherry‐Gal8 foci, and DiD puncta into distinct objects, which we then counted (Figure S20, Supporting Information). This representative image also shows that colloid uptake is quite uniform rather than concentrated in a few cells.

We found that some ionizable fulvestrant analog colloids caused endo‐lysosomal disruption, as shown by an increased number of Gal8 foci compared to blank and fulvestrant colloid controls (Figure 4B for 7e and Figures S21–S24 for the remaining conditions, Supporting Information). Some of these formulations appeared to result in small Gal8 foci overlapping with the colloids. In contrast, others resulted in large and separate Gal8 foci that were present even when colloid endocytosis was blocked. Quantifying this effect, we found that ionizable fulvestrant analog colloids resulted in more Gal8 foci per cell and, thus, more endo‐lysosomal disruption than fulvestrant colloids (Figure 4C). Further, colloids formulated from ionizable fulvestrant analogs with higher p*K*_a_ values resulted in greater endo‐lysosomal disruption.

These findings confirm our hypothesis that endo‐lysosomal disruption can be triggered by adding ionizable groups to colloid‐forming small molecule drugs. Furthermore, p*K*_a_, which determines the pH at which ionization occurs, controls endo‐lysosomal disruption. Although this relationship has been well explored in nanoparticles composed of ionizable lipids and polymers,^[^ ^17^, ^18^, ^55^ ^]^ this phenomenon has not, to our knowledge, been investigated and manipulated for small molecule drug colloids until now.

### 2.5 Fulvestrant Analogs Disrupt Endo‐Lysosomes by Multiple Mechanisms

Since we observed two distinct patterns of Gal8 foci fluorescence, we wondered whether our fulvestrant analog colloids could disrupt endosomes and lysosomes by multiple mechanisms. For those formulations that only resulted in Gal8 foci when endocytosed, the colloidal drugs could become protonated, leading to endosomal disruption through membrane fusion or permeabilization.^[^ ^56^, ^57^, ^58^, ^59^ ^]^ However, the phospholipidosis‐associated, free drug‐driven mechanism could explain the formulations that resulted in large, separate Gal8 foci, even when endocytosis was blocked. While the equilibrium favors the colloidal form above the critical aggregation concentration (≈1 µm for fulvestrant^[^ ^7^ ^]^), there is always some free drug which could lead to these different mechanisms of endo‐lysosomal disruption.^[^ ^60^, ^61^ ^]^ To further probe colloidal stability, we incorporated a FRET pair in the colloids and used fluorescence as a proxy for colloid stability. We observed greater fluorescence in colloids dispersed in PBS versus 10% FBS (Figure S25, Supporting Information), confirming earlier demonstrations that serum proteins further solubilize colloidal drugs.^[^ ^12^ ^]^

**Figure 5:**
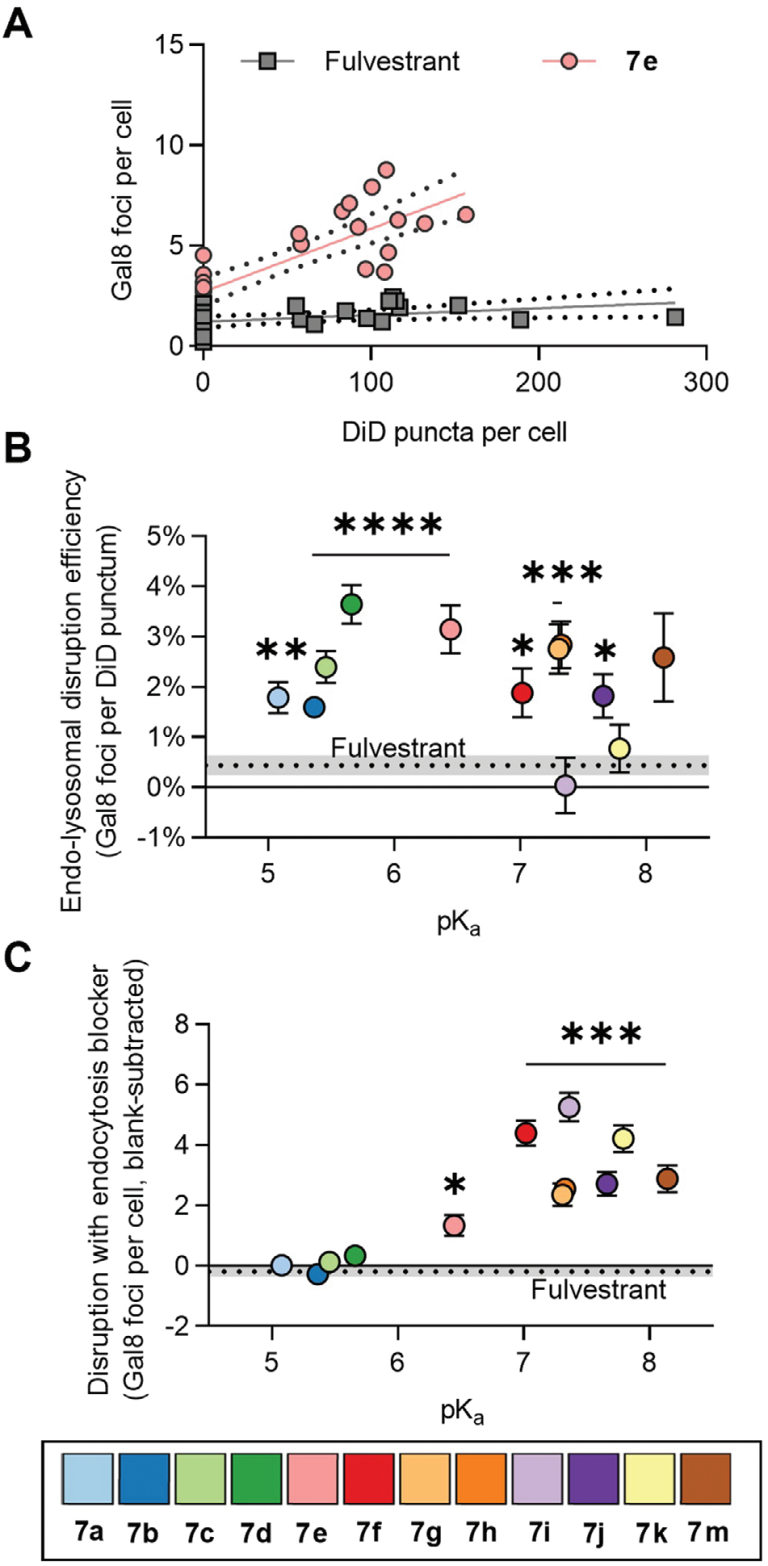
Regression analysis disentangles the endo‐lysosomal disruption caused by endocytosed colloids. A) The number of Gal8 foci as a function of the uptake of fulvestrant or **7e** colloid‐containing vesicles (DiD puncta). Each data point represents the average of three technical replicates. B) Endosome disruption efficiency as a function of ionizable fulvestrant analog p*K*_a_. C) Endocytosis‐independent endosomal disruption as a function of ionizable fulvestrant analog p*K*_a_ B,C) *n* ≥ 12 biological replicates, Brown–Forsythe and Welch ANOVA tests with Dunnett T3 post hoc test, *p < 0.05, **p < 0.01, ***p < 0.001, ****p < 0.0001 compared to fulvestrant).

We reasoned that blocking endocytosis would allow us to disentangle these mechanisms by isolating the effect of the free drug, which can enter cells via passive diffusion. Thus, we treated cells with colloids with or without 20 µm hydroxydynasore, which we previously showed to prevent colloid endocytosis. We then plotted the number of Gal8 foci per cell as a function of colloid uptake (**Figure** **5**A for fulvestrant and 7e, Figure S26 for the remaining conditions, Supporting Information). We performed linear regressions to yield slopes, representing the efficiency of endo‐lysosomal disruption by colloids, and y‐intercepts, indicating free drug‐mediated disruption. Using this method, we found that colloids of fulvestrant analogs **7a–7 h** and **7j** had significantly higher endo‐lysosome disruption efficiencies than colloids of fulvestrant (Figure 5B). Additionally, free analogs **7e–7k** and **7m** caused significantly greater endo‐lysosomal disruption than free fulvestrant (Figure 5C). Neither the cationic **7l** nor the unmodified sulfoximine analog **11** resulted in significant endo‐lysosomal disruption (Figure S27A—C, Supporting Information), potentially because they do not transition from neutral to cationic in the endo‐lysosomal pathway. Interestingly, colloids comprised of 90% fulvestrant and 10% of analogs **7f**—**7k** (Figure S27D,E, Supporting Information), but not colloids of either analog **7i** or **7k** alone (Figure 5B), had significantly higher endosomal disruption efficiency than fulvestrant colloids, possibly because co‐formulation decreases overall p*K*_a_ and solubility.^[^ ^61^ ^]^ To confirm the ability of free drugs to elicit endo‐lysosomal disruption, we treated cells with non‐colloidal fulvestrant analog formulations for 24 h. Under these conditions, analogs **7f**, **7** **h**, and **7j** resulted in significantly greater endo‐lysosomal disruption than fulvestrant (Figure S28, Supporting Information).

**Figure 6:**
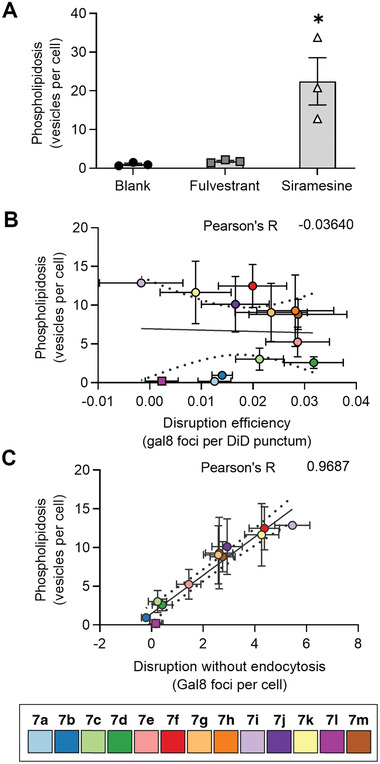
The endo‐lysosomal disruption that occurs independently of colloid endocytosis is associated with phospholipidosis (*n* ≥ 3 biological replicates, mean ± SEM). A) The fluorescent lipid‐based phospholipidosis assay functions as intended, as shown with two negative controls (media without colloids and 5 µm colloidal fulvestrant) and one positive control (20 µm siramesine; ordinary one‐way ANOVA with Holm–Sidak posthoc test, *p < 0.05 compared to all other groups). B) Phospholipidosis is poorly correlated (*R* = −0.1317) with endosome disruption efficiency. C) Phospholipidosis is strongly correlated (*R* = 0.9664) with endocytosis‐independent endo‐lysosomal disruption.

To verify the disruption mechanism caused by free fulvestrant analogs **7e–7k** and **7m**, we measured phospholipidosis, which is tightly correlated with cationic drug‐mediated lysosomal membrane permeabilization.^[^ ^31^ ^]^ We quantified phospholipidosis using a commonly‐used fluorescent lipid that accumulates in lysosomes: nitrobenzoxadiazole dipalmitoyl phosphatidylethanolamine (NBD‐PE).^[^ ^32^, ^62^, ^63^ ^]^ We collected images of cells co‐treated with NBD‐PE and drugs (Figure S29, Supporting Information) and counted the number of these vesicles. As expected, the known phospholipidosis inducer siramesine (p*K*_a_ 7.4, Figure S30, Supporting Information) resulted in more phospholipidosis vesicles per cell than cells treated with either blank control (i.e., media without colloids) or fulvestrant colloids (**Figure** **6**A). We also treated the cells with colloids of analogs **7a–7m** and **11** and found that phospholipidosis was poorly correlated with endo‐lysosomal disruption efficiency (*R* = −0.1317, Figure 6B) but strongly correlated with disruption mediated by free drug (*R* = 0.9664, **Figure** **6**C). To confirm the propensity of some of the soluble fulvestrant analogs to cause phospholipidosis, we treated cells with noncolloidal formulations: soluble **7f–7k** resulted in significant phospholipidosis, whereas soluble **7a–7e** and **7m** did not (Figure S31, Supporting Information). These results show a p*K*_a_‐dependent endolysosomal disruption mechanism in the absence of phospholipidosis for those analogs, **7a–7e**, with p*K*_a_’s less than 5.7. The data also further validate our hypothesis that endo‐lysosomal disruption can occur in an endocytosis‐independent manner by residual free drug molecules diffusing into lysosomes and interfering with lipid homeostasis.

These results show that colloidal formulations of ionizable fulvestrant analogs disrupt endo‐lysosomes through two distinct mechanisms. First, many ionizable colloids can cause endo‐lysosomal disruption directly. However, with higher p*K*_a_ analogs, this effect is often replaced, or at least overshadowed, by that of free drug‐mediated lysosomal membrane permeabilization. Although we show that this free drug can originate outside the cell, colloids within the lysosomes could also bring about endo‐lysosomal disruption through this mechanism.

The colloid‐driven endosome disruption observed with lower p*K*_a_ fulvestrant analogs is similar to the behavior of ionizable lipid or polymer‐based nanoparticles.^[^ ^53^, ^56^, ^57^, ^64^, ^65^ ^]^ The mechanisms whereby nanoparticles cause endosomal disruption rely on a high local concentration of ionizable groups, as was observed for colloids of ionizable analogs **7a**–**7d** (p*K*_a_ 5.1–5.7) where endosome disruption was observed only when the colloids, which typically comprise 10^5^–10^8^ drug molecules,^[^ ^66^ ^]^ were endocytosed. In contrast, the endo‐lysosomal disruption efficiencies of fulvestrant and **11** (p*K*_a_ values < 5.1) were poor, likely due to their inefficient ionization at endosomal pH. We note that endo‐lysosomal disruption was generally inefficient, with the number of colloid puncta per cells exceeding that of Gal8 foci. This result is consistent with reports of low endosome escape efficiency in the literature.^[^ ^25^, ^53^, ^54^, ^65^ ^]^

The higher p*K*_a_ fulvestrant analogs likely disrupt cellular membranes by a mechanism similar to that of cationic amphiphilic drugs, which are thought to disrupt lysosomes and cause phospholipidosis.^[^ ^29^, ^67^ ^]^ This behavior does not require the presence of intact colloids. Our results indicate that ionizable analogs **7e–7k** and **7m** (p*K*_a_ ≥ 6.4) act through this mechanism because they caused significant endo‐lysosomal disruption even when colloid endocytosis was blocked. These findings are consistent with literature reports that drugs containing tertiary amines with p*K*_a_ values of 6.8 or greater are likely to induce phospholipidosis.^[^ ^31^ ^]^ This mechanism may explain the heightened toxicity of these ionizable analogs to both cancer cells and lung fibroblasts—although phospholipidosis is typically transient and lysosomal membrane permeabilization has been exploited in drug delivery,^[^ ^25^, ^26^, ^27^, ^28^ ^]^ they can eventually cause cell death through apoptosis.^[^ ^24^ ^]^ Additionally, cancer cells, which were used herein, are particularly sensitive to this effect.^[^ ^67^, ^68^ ^]^ Interestingly, colloidal formulations of analogs **7e–7 h** and **7j** caused endo‐lysosomal disruption mediated by colloids and free drug, demonstrating that the two disruption mechanisms are not mutually exclusive.

Using a range of ionizable chemical motifs, we unraveled the complex role of p*K*_a_ on the potency and mechanism of endo‐lysosomal disruption. Not all ionizable drugs disrupted endo‐lysosomal membranes equally: those with p*K*_a_ values of less than 5.1 did not disrupt endosomes, and those with p*K*_a_ values above 6.4 tended to disrupt lysosomes as free drug rather than as colloids. We found optimal endo‐lysosomal disruption with colloidal drug aggregates with p*K*_a_ values between 5.1 and 5.7, which is similar to that of ionizable lipid p*K*_a_ values for endosomal disruption with lipid nanoparticles.^[^ ^43^ ^]^ Our colloidal drug aggregate approach overcomes drug loading challenges associated with traditional lipid or polymeric nanoparticles. Furthermore, we identify drug analogs that enable endosomal disruption without causing phospholipidosis—an undesirable side effect associated with toxicity in vitro and in vivo.

## 3 Conclusions

We demonstrate a generalizable strategy for achieving endo‐lysosomal disruption with colloidal drug aggregates. We show that modifying a neutral drug, in this case fulvestrant, with ionizable functional groups allows it to disrupt endo‐lysosomes, allowing mass transfer between these vesicles and the cytosol. Additionally, we identify a p*K*_a_ range, 5.1–5.7, that enables endosomal disruption without causing phospholipidosis, thereby avoiding a potentially harmful side effect. This strategy may be most useful during drug development, where the structures of drugs are tuned to optimize their pharmacokinetics and tolerability. As medicinal chemists explore new chemical space for small‐molecule drugs, colloid formulations could help overcome issues with solubility and permeability. Incorporating ionizable groups in these drugs will allow pH‐triggered release from endosomes and lysosomes.

## 4 Experimental Section

### Materials

Fulvestrant and siramesine (hydrochloride) were purchased from MedChemExpress (Monmouth Junction, NJ). 3,4‐Dihydro‐2*H*‐pyran, trifluoroacetic acid, sodium bicarbonate, sodium carbonate, sodium chloride, magnesium sulfate, ammonium carbamate, (diacetoxyiodo)benzene, triethylamine, *N*,*N*‐diisopropylethylamine, *tert*‐butyldimethylsilyl chloride, bromopropanoyl chloride, bromoacetyl bromide, ammonium chloride, dimethylamine hydrochloride, trimethylammonium chloride, pyrrolidine, morpholine, 2‐(*p*‐toluidino) naphthalene‐6‐sulfonic acid, dimethyl sulfoxide (DMSO), ampicillin, l‐glutamine, polybrene, hydroxydynasore, RPMI 1640, DMEM, DMEM high glucose, GlutaMAX (35050‐061), sodium pyruvate solution (11360‐070), and MEM nonessential amino acids solution (11140‐050) were purchased from Sigma‐Aldrich (St. Louis, MO). Deuterated solvents were purchased from Cambridge Isotope Laboratories (Tewksbury, MA). Dichloromethane, hexanes, ethyl acetate, hexanes, methanol, acetone, ethanol, acetonitrile, and piperidine were purchased from Caledon Laboratories (Georgetown, ON, Canada). Dimethylformamide was purchased from Alfa Aesar (Ward Hill, MA). Imidazole, paraformaldehyde (PFA), Lennox broth and agar were purchased from BioShop Canada (Burlington, ON, Canada). *N*‐Boc glycine was purchased from Nova Biochem/Merck (Darmstadt, Germany). Phosphate buffered saline (PBS), Hank's balanced salt solution (HBSS), fetal bovine serum (FBS), penicillin‐streptomycin, and trypsin‐EDTA were purchased from Wisent Bioproducts (St. Bruno, QC, Canada). 1,2‐Distearoyl‐sn‐glycero‐3‐phosphocholine, 1,2‐dilauroyl‐sn‐glycero‐3‐phosphocholine, and 1,2‐dimyristoyl‐rac‐glycero‐3‐methoxypolyethylene glycol‐2000 were purchased from Avanti Polar Lipids (Alabaster, AB). Ultra‐pure polysorbate 80 was purchased from NOF America Corporation (White Plains, NY). OneShot Top10 Chemically Competent *E. coli*, *N*‐(7‐nitrobenz‐2‐oxa‐1,3‐diazol‐4‐yl)‐1,2‐dihexadecanoyl‐*sn*‐glycero‐3‐phosphoethanolamine, triethylammonium salt, 1,1“‐dioctadecyl‐3,3,3”,3'‐tetramethylindodicarbocyanine, 4‐chlorobenzenesulfonate salt (DiD), CholEsteryl BODIPY FL C12, CholEsteryl BODIPY 542/563 C11, DMEM, RPMI 1640, recombinant human insulin, and PrestoBlue HS Cell Viability Reagent were purchased from Thermo Fisher Scientific. Hoechst 33 342 was purchased from Cell Signaling Technology (Danvers, MA). The plasmid encoding the mCherry‐galectin 8 fusion was a generous gift from F. Randow.^[^ ^69^ ^]^ Viral vector plasmids pCMV‐VSV‐G (8454) and pUMVC (8449) were obtained from Addgene (Teddington, UK) as DH5*α* stab cultures.^[^ ^70^ ^]^ A QIAprep Spin Miniprep Kit was obtained from Qiagen (Germantown, MD). Recombinant human apolipoprotein E3 was obtained from Abcam (Cambridge, UK).

### Chemical Synthesis

Detailed experimental procedures for chemical synthesis and characterization of the products can be found in the Supporting Information.

### p*K*_a_ Measurement

Compound p*K*_a_ values were measured by a fluorescence assay as previously described.^[^ ^17^, ^37^ ^]^ Solutions containing 1.2 mm TNS and 2 mm test compound in DMSO were prepared. 2 µL of this solution was mixed with 200 µL of pH‐adjusted PBS (137 mm NaCl, 11.8 mm phosphate as a mixture of NaH_2_PO_4_ and Na_2_HPO_4_). The fluorescence of the TNS was measured on a plate reader (*λ*_ex_ = 322 nm, *λ*_em_ = 431 nm). p*K*_a_ values were obtained by fitting fluorescence versus pH data with Equation (1)

(1) $$\mathrm{Fluorescence}\;=\;\mathrm{Background}+\;\frac{\mathrm{Maximum}-\mathrm{Background}}{1+10^{\mathrm{pH}-{\mathrm{pK}}_{a}}}$$

### Colloid Formulation

Colloidal drug aggregates were formulated as described previously.^[^ ^11^ ^]^ Briefly, a 50 mm solution of fulvestrant or fulvestrant analog in DMSO was mixed with solutions of excipients in ethanol. Extra ethanol was added to bring the solution to 50x its final concentration. Then, PBS was added by pipette to form colloids at a fulvestrant concentration of 200 µm and an ethanol concentration of 2% (v v^−1^).

### Characterization by Dynamic Light Scattering

Hydrodynamic diameter (z‐average), polydispersity index (PDI), and scattering intensity were measured by dynamic light scattering using a DynaPro Plate Reader II (Wyatt Technologies) that the manufacturer optimized for detecting colloidal aggregates (i.e., 100–1000 nm particles). The instrument was configured with a 60 mW 830 nm laser and a detector angle of 158°. A 25 µL sample of each formulation was pipetted into each well of a 384‐well plate and measured with 20 acquisitions per sample at 25 °C.

### Cell Culture

HEK293T, SKOV3, MCF7, BT474, and normal human primary lung fibroblast (PCS201‐013) cells were obtained from ATCC. Cells were maintained in a humified incubator at 37 °C with 5% atmospheric CO_2_. DMEM was used as the base media for the HEK293T cells, DMEM high glucose was used as the base media for the lung fibroblasts, and RPMI 1640 was used for the others. Cells were grown in 75 cm^2^ tissue culture flasks with 10 mL of media supplemented with 10% FBS. For SKOV3, MCF7, lung fibroblast, and BT474 cells, the media was supplemented with 10 UI mL^−1^ penicillin and 10 µg mL^−1^ streptomycin. For MCF7 cells, the media was also supplemented with 10 µg mL^−1^ of human insulin. For the lung fibroblasts, the media was also supplemented with 1% v/v GlutaMAX, 1% v/v sodium pyruvate solution, and 1% v/v MEM nonessential amino acids solution. The cells were passaged once per week following detachment with trypsin‐EDTA, replacement of the supernatant with fresh media, and subculture into a new flask with fresh media. Subculture ratios varied from 1:50 (SKOV3 cells) to 1:3 (MCF7 and BT474 cells).

### Generation of Endosome Disruption Reporter Cells

The mCherryGal8 plasmid was reconstituted from a filter paper spot by vortexing in water for 2 min. Next, the plasmid was transformed into OneShot Top10 chemically competent E. coli following the manufacturer's protocol. Colonies of the transformed bacteria were then grown on agar plates that contained 100 µg mL^−1^ of ampicillin. Next, plasmid‐expressing E. coli colonies from these agar plates or commercially available stab cultures were expanded on a shaker plate for 24 h at 37 °C in 10 mL of Lennox broth that contained 100 µg mL^−1^ of ampicillin. The plasmids were then isolated using a QIAprep Spin Miniprep Kit.

A viral vector containing mCherryGal8 was prepared using HEK293T cells in a biosafety level 2 facility as described by Stewart et al.^[^ ^71^ ^]^ First, 300 000 cells were plated in one well of a 12‐well tissue culture polystyrene plate. Then, 445 ng of pUMVC, 55 ng of pCMV‐VSV‐G, and 500 ng of pmCherryGal8 were mixed with 3 µL of Fugene 6. The mixture was incubated for 30 min at room temperature and then added to the HEK293T cells. After 24 h, the media was replaced. After an additional 24 h, the media supernatant was collected and filtered through a 0.45 µm syringe filter, yielding the viral vector in the filtrate.

Next, SKOV3 cells were infected with the viral vector. First, 1.25 × 10^5^ cells were plated in each well of a 12‐well plate and allowed to adhere overnight. Then, 500 µL of the virus suspension was mixed with 0.5 µL of polybrene. This mixture was added to the cells for 2 h and then replaced with fresh media. After an additional 2 d, the media was replaced with media containing 500 ng mL^−1^ puromycin. The cells were incubated in this media for 3 weeks with weekly media replacement to select transfected cells resistant to puromycin. Finally, surviving cells were expanded and then purified using fluorescence‐assisted cell sorting, yielding a population of cells that stably express the mCherry‐Gal8 construct.

### Cell Treatment for Imaging Experiments

2.5 × 10^3^ SKOV3‐mCherry‐Gal8 cells in 25 µL were plated in each well of a 384‐well plate (Greiner Bio‐One 781 097) and allowed to adhere overnight. Then, 10 µL of media with or without 100 µm hydroxydynasore was added to the cells, and the plate was incubated for 30 min. Next, colloidal drug aggregates were prepared, and 1.2 µL was pipetted into each well, followed by 10 µL of PBS to facilitate good mixing. The plate was incubated for a variable length of time (usually 3 h); then, the media was removed, and the cells were washed with HBSS containing BSA, fixed for 15 min with 4% (m v^−1^) PFA in PBS, and stained for 15 min with 5 µg mL^−1^ Hoechst in PBS. The media was finally replaced with PBS before imaging.

For experiments investigating phospholipidosis, 10 µL of 50 µm NBD‐PE in RPMI 1640 (previously filtered through a 0.22 µm syringe filter to remove aggregates) was added to each well before allowing the cells to adhere overnight.

### Wide‐field Fluorescence Microscopy Image Acquisition

Fluorescence images were acquired using a Zeiss Apotome Live Cell System (Axio Observer Z.1 inverted fluorescent microscope) with a long working distance 40x Plan Neofluor objective (NA 0.6) (Carl Zeiss Canada), an X‐Cite 120 LED fluorescent lamp (Lumen Dynamics), and an Axiocam 506 mono camera (Carl Zeiss Canada). Zen Blue 2.3 software was used to capture images (Carl Zeiss Canada). The focal plane selection was automated based on the nuclei channel (Hoechst). Four tiles per well were collected and stitched into a single image. For Hoechst, an excitation band of 359–371 nm and an emission band of >397 nm were used. For NBD‐PE, an excitation band of 475–495 nm and an emission band of 515–565 nm were used. For mCherry‐Gal8, an excitation band of 540–552 nm and an emission band of 575–640 nm were used. For DiD, an excitation band of 625–655 and an emission band of 665–715 nm were used. Illumination was typically carried out at 100% laser power, with illumination times ranging from 50 to 2000 ms, depending on the channel. Black‐walled plates were used to minimize photobleaching from stray light. Illumination and detector settings were held constant across different wells and plates.

### Confocal Microscopy Image Acquisition

Confocal fluorescence images were acquired using a LSM 880 Elyra super‐resolution microscope. Samples were prepared on glass slides with removable chambers. After treatment and staining, the chambers were removed, and the sample was sealed with a cover slip and ProLong Gold antifade mountant. The samples were then imaged using a 63x oil immersion objective. Illumination was typically carried out at 1% laser power with a dwell time of 8 µs per pixel. Z‐sections spanning the cells from top to bottom were collected with 1.15 µm spacing.

### Image Processing

Image processing was performed with MATLAB based on an algorithm originally developed by Kameron Kilchrist.^[^ ^55^ ^]^ The modified code can be found on GitHub (https://github.com/kaislaughter/mChG8_image_processing). The primary purpose of this code is to identify and count cell nuclei, DiD puncta (endocytosed colloidal drug aggregates), galectin 8 foci (disrupted endo‐lysosomes), and phospholipidosis vesicles. First, images were treated with a top hat transform and threshold to remove background fluorescence, including diffuse cytosolic galectin 8 fluorescence. Next, the images were binarized, and a watershed algorithm was applied to split up partially overlapping features, such as two side‐by‐side nuclei. Finally, the number of features was counted and tabulated. The results of three images, each containing ≈100 cells and originating from a separate well, were averaged to yield the value for each biological replicate.

### Cell Viability and Proliferation Experiments

5 × 10^3^ MCF7 or BT474 cells in 150 µL of media were added to each well of a transparent polystyrene 96‐well plate and allowed to adhere overnight. For lung fibroblasts, 2.5 × 10^3^ cells were used. The pretreatment total cellular metabolic activity for proliferation experiments was measured using PrestoBlue according to the manufacturer's instructions; the reagent was then removed and replaced with 150 µL of fresh media in preparation for treatment. Treatments were prepared at 4x the final concentration. Then, 50 µL of these solutions were added to each well, and the cells were incubated for the indicated time. The post‐treatment total cellular metabolic activity was then measured using PrestoBlue. For regular experiments, metabolic activity relative to the blank‐treated control was calculated using Equation (2). For proliferation experiments, metabolic activity relative to the pretreatment value was calculated using Equation (3). IC_50_ values were then calculated by curve fitting Equation (4) or Equation (5) to the metabolic activity (Y) versus [Drug] data. Since the blank‐treatment and pre‐treatment are constant scaling factors that do not affect the shape of the curve, the IC_50_ values are unaffected by changing from %Relative to %Original metabolic activity (or vice versa)

(2) $$\begin{array}{c} \%\mathrm{Relative}\;\mathrm{metabolic}\;\mathrm{activity}=\frac{I_{\mathrm{sample},\;6\;d}-I_{\mathrm{background},6\;d}}{I_{\mathrm{control},6\;d}-I_{\mathrm{background},6\;d}}\times 100\% \end{array}$$

(3) $$\begin{array}{c} \%\mathrm{Original}\;\mathrm{metabolic}\;\mathrm{activity}=\frac{I_{\mathrm{sample},\;6\;d}-I_{\mathrm{background},\;6\;d}}{I_{\mathrm{sample},\;0\;d}-I_{\mathrm{background},\;0\;d}}\times 100\% \end{array}$$

(4) $$Y\;=\;\mathrm{Bottom}+\;\frac{\mathrm{Top}-\mathrm{Bottom}}{1+{\left(\frac{IC_{50}}{\left[\mathrm{Drug}\right]}\right)}^{\mathrm{Hill}}}$$

(5) $$Y\;=\;\mathrm{Bottom}+\;\frac{\mathrm{Plateau}-\mathrm{Bottom}}{1+{\left(\frac{IC_{50,\mathrm{tox}}}{\left[\mathrm{Drug}\right]}\right)}^{{\mathrm{Hill}}_{\mathrm{tox}}}}+\frac{\mathrm{Top}-\mathrm{Plateau}}{1+{\left(\frac{IC_{50,\mathrm{stat}}}{\left[\mathrm{Drug}\right]}\right)}^{{\mathrm{Hill}}_{\mathrm{stat}}}}$$

## Conflict of Interest

The authors declare the following competing financial interest(s): we have submitted a patent based on this paper.

## Supporting information

Supporting Information

## Data Availability

The data that support the findings of this study are available from the corresponding author upon reasonable request.
