# Supplementary material for: Synthetic Ionizable Colloidal Drug Aggregates Enable Endosomal Disruption
Source: Adv Sci (Weinh). 2023 Mar 11;10(13):2300311. doi: 10.1002/advs.202300311 (PMC10161099; doi:10.1002/advs.202300311)
Supplement: Supplementary file 1 — Supporting Information [file ADVS-10-2300311-s001.pdf]

ADVANCED SCIENCE
Open Access

Supporting Information

for *Adv. Sci.*, DOI 10.1002/advs.202300311

Synthetic Ionizable Colloidal Drug Aggregates Enable Endosomal Disruption

*Eric N. Donders, Kai V. Slaughter, Christian Dank, Ahil N. Ganesh, Brian K. Shoichet, Mark Lautens and Molly S. Shoichet**

Synthetic Ionizable Colloidal Drug Aggregates Enable Endosomal Disruption

*Eric N. Donders, Kai V. Slaughter, Christian Dank, Ahil N. Ganesh, Brian K. Shoichet, Mark Lautens, Molly S. Shoichet**

E. Donders, A. Ganesh, M. Shoichet
Department of Chemical Engineering & Applied Chemistry, University of Toronto, 200 College Street, Toronto, Ontario M5S 3E5, Canada

E. Donders, K. Slaughter, A. Ganesh, M. Shoichet
Institute of Biomedical Engineering, University of Toronto, 164 College Street, Toronto, Ontario M5S 3G9, Canada

E. Donders, K. Slaughter, A. Ganesh, M. Shoichet
Donnelly Centre, University of Toronto, 160 College Street, Toronto, Ontario M5S3E1, Canada

C. Dank, M. Lautens
Department of Chemistry, University of Toronto, 80 St. George Street, Toronto, Ontario M5S 3H6, Canada

B. Shoichet
Department of Pharmaceutical Chemistry, University of California San Francisco, 1700 Fourth Street, Mail Box 2550, San Francisco, California 94143, United States

E-mail: molly.shoichet@utoronto.ca

Keywords: Colloidal drug aggregates, nanoparticles, endosomal disruption, fulvestrant, drug delivery

ABBREVIATIONS

ApoE, apolipoprotein E; CAC, critical aggregation concentration; DCM, dichloromethane; DiD, 1,1'-dioctadecyl-3,3,3',3'-tetramethylindodicarbocyanine, 4-chlorobenzenesulfonate salt; DIPEA, *N*,*N*-diisopropylethylamine; DLPC, dilauroylphosphatidylcholine; DLS, dynamic light scattering; DMF, dimethylformamide; DMG-PEG 2000, 1,2-dimyristoyl-rac-glycero-3-methoxypolyethylene glycol-2000; DMSO, dimethylsulfoxide; DSPC, distearoylphosphatidylcholine; ER, estrogen receptor; FBS, fetal bovine serum; FRET, Förster resonance energy transfer; Gal8, galectin 8; HBSS, Hank's balanced salt solution; HCTU, *O*-(1*H*-6-Chlorobenzotriazole-1-yl)-1,1,3,3-tetramethyluronium hexafluorophosphate; HER2, human epidermal growth factor receptor 2; IC_{50}, half-maximal inhibitory concentration; NBD-PE, *N*-(7-nitrobenz-2-oxa-1,3-diazol-4-yl)-1,2-dihexadecanoyl-sn-glycero-3-phosphoethanolamine, triethylammonium salt; PBS, phosphate buffered saline; PDI, polydispersity index; PEG, polyethylene glycol; PFA, paraformaldehyde; PTFE, polytetrafluoroethylene; SEM, standard error of the mean; TBS, *tert*-butyldimethylsilyl; TBSCl, *tert*-butyldimethylsilyl chloride; TFA; trifluoroacetic acid; TNS, 2-(*p*-toluidino) naphthalene-6-sulfonic acid; THP, tetrahydropyran.

Supplementary figures

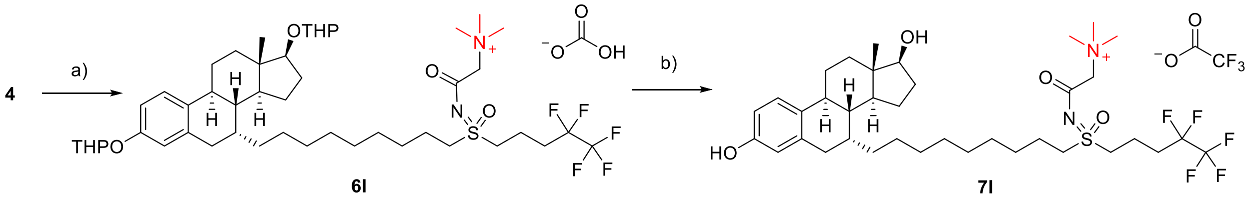

Scheme S1. Synthesis of fulvestrant analog **7l**. Reagents and conditions: a) 1) trimethylamine hydrochloride, triethylamine, dichloromethane, rt, 4 h, 2) $NaHCO_3$ quench; b) 2% TFA (>10 equiv) in methanol, rt, 16 h. This reaction was unsuccessful with acrylamide **5**. Similarly, the reaction of intermediate **4** with ammonium chloride and triethylamine resulted in a triethylammonium side product as opposed to the intended primary amine; however, intermediate **5** did not react at all under these conditions. These results indicate that either the alkyl bromide **4** is more electrophilic than the acrylamide **5** or the addition of tertiary amines to the acrylamide is reversible.

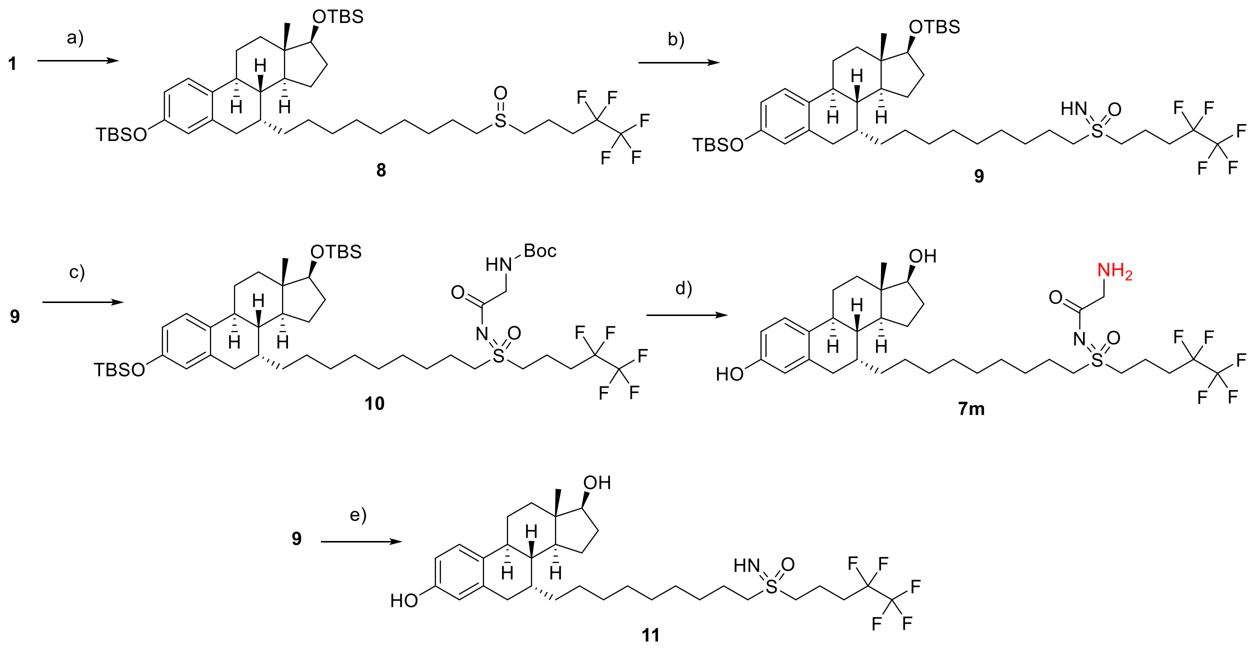

Scheme S2. Syntheses of fulvestrant analogs **7m** and **11**. Reagents and conditions: a) TBSCl (3.2 equiv), imidazole, DMF, rt, 18 h; b) (diacetoxyiodo)benzene (3 equiv), ammonium carbamate (4 equiv), methanol, rt, 4 h; c) N-Boc-glycine, HCTU, DIPEA, 0 °C→rt, 18 h; d) 1) TBAF, acetic acid, THF, 60 °C, 4 h, 2) 2% TFA (>10 equiv) in methanol, rt, 16 h; e) HCl, MeOH, rt, 18 h.

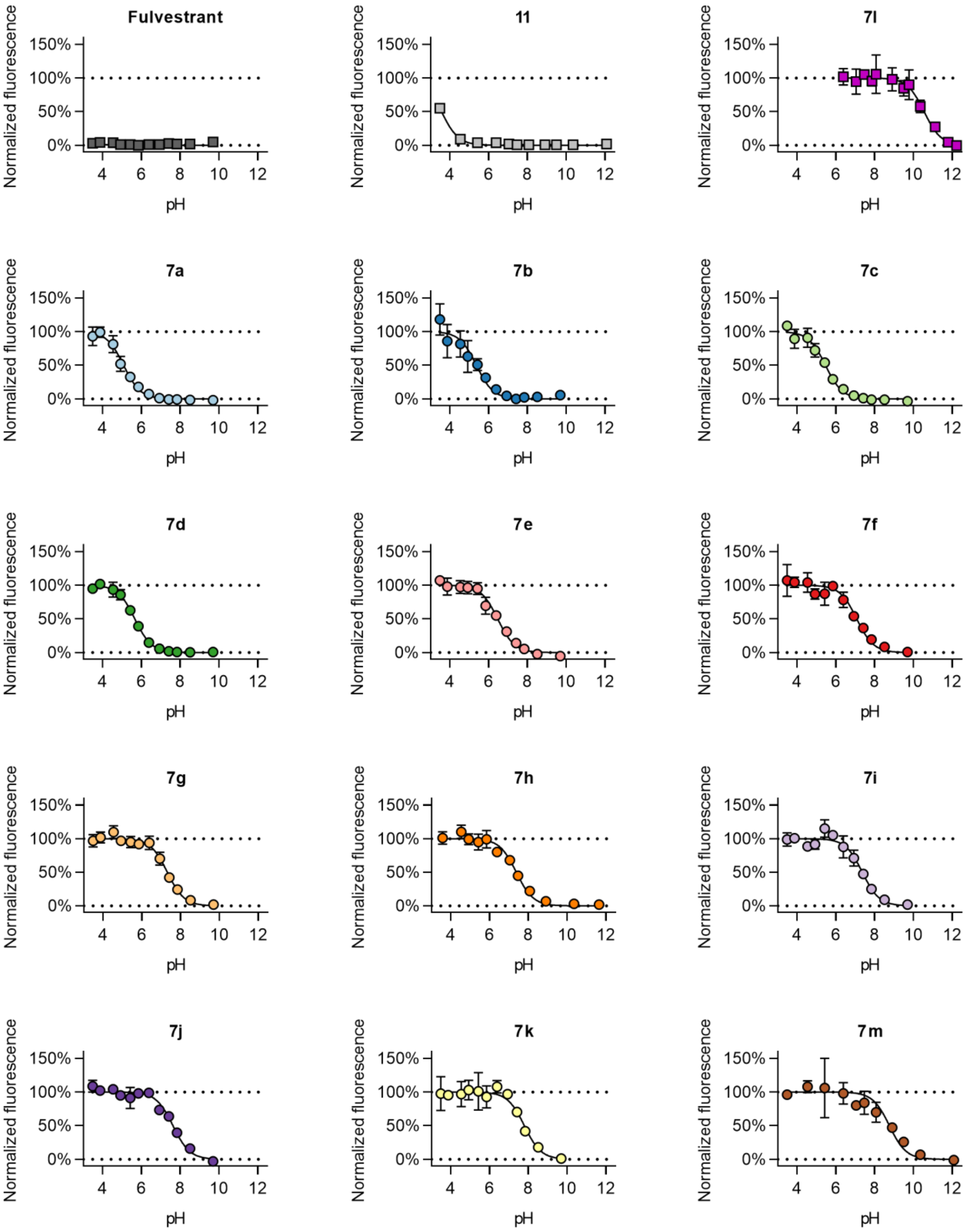

Figure S1. Plots of 6-(*p*-Toluidino)-2-naphthalenesulfonic acid (TNS) fluorescence versus pH that were used to measure pK_a. The raw fluorescence values were baseline-subtracted and normalized to the signal of the top plateau. The pK_a is calculated as the pH at which the normalized TNS fluorescence is 50% (n = 3, mean ± SEM).

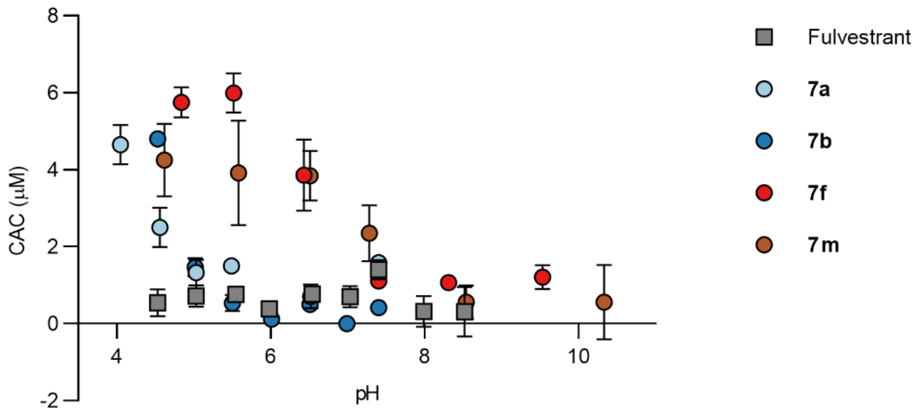

Figure S2. The critical aggregation concentration (CAC) of ionizable fulvestrant analogs (but not fulvestrant) increases in acidic media. Solutions of fulvestrant analogs in dimethyl sulfoxide were diluted with pH-adjusted PBS and analyzed by dynamic light scattering. CAC values were calculated by curve fitting as previously described[1] ($n \geq 3$, mean ± SEM).

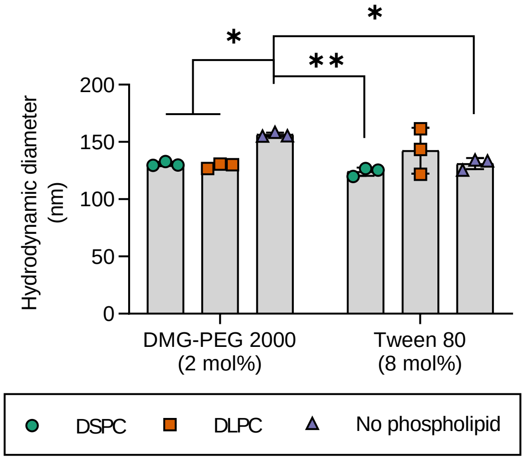

Figure S3. Phospholipid and surfactant excipients stabilize 200 µM fulvestrant colloids in PBS. Hydrodynamic diameter of stabilized fulvestrant colloids immediately after formulation ($n \geq 3$, mean ± SD, two-way ANOVA with Tukey's post-hoc test comparing all groups, *$p < 0.05$, **$p < 0.01$).

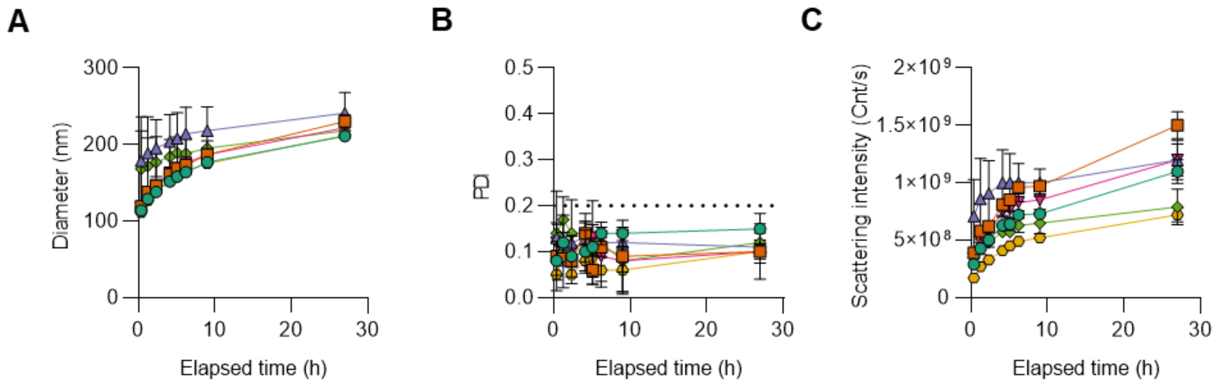

D

Symbol	Name	Fulvestrant µM	Fulvestrant mass %	DSPC µM	DSPC mass %	DLPC µM	DLPC mass %	DMG-PEG 2000 µM	DMG-PEG 2000 mass %	Tween 80 µM	Tween 80 mass %	DiD nM	DiD mass %
	DMG-PEG 2000 + DSPC	200	72.1	40	18.8	0	0	6	8.95	0	0	400	0.13
	DMG-PEG 2000 + DLPC	200	75.1	0	0	40	15.4	6	9.32	0	0	400	0.13
	DMG-PEG 2000	200	88.8	0	0	0	0	6	11.0	0	0	400	0.15
	Tween 80 + DSPC	200	67.7	40	17.6	0	0	0	0	20	14.6	400	0.12
	Tween 80 + DLPC	200	70.3	0	0	40	14.4	0	0	20	15.2	400	0.12
	Tween 80	200	82.13	0	0	0	0	0	0	20	17.7	400	0.14

Figure S4. Characterization of 200 µM fulvestrant colloids formulated with different lipid and polymer excipients in PBS. **A** Hydrodynamic diameter of the colloids over time. **B** Polydispersity index (PDI) of the colloids over time. A PDI value of less than 0.2 is generally considered to indicate a narrow size distribution. **C** Scattering intensity of the colloids over time. Scattering intensity is proportional to the number of colloids remaining in suspension and is a function of colloid size. **D** Legend entries for **A**-**C** and compositions of each formulation. Statistics for **A**-**C**: n = 3 separate colloid formulations, mean ± SD.

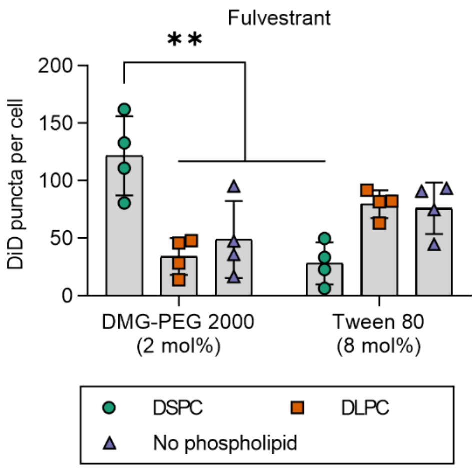

Figure S5. Quantification of colloid uptake in SKOV3 cells. Number of DiD puncta (each containing one or more endocytosed colloids) per cell after 3 h of incubation with 5 µM of fulvestrant formulated with different excipients. Colloids were imaged using a widefield fluorescence microscope with a large depth of field (N ≥ 3, mean ± SD, two-way ANOVA with Tukey's post-hoc test comparing all groups, **$p < 0.01$).

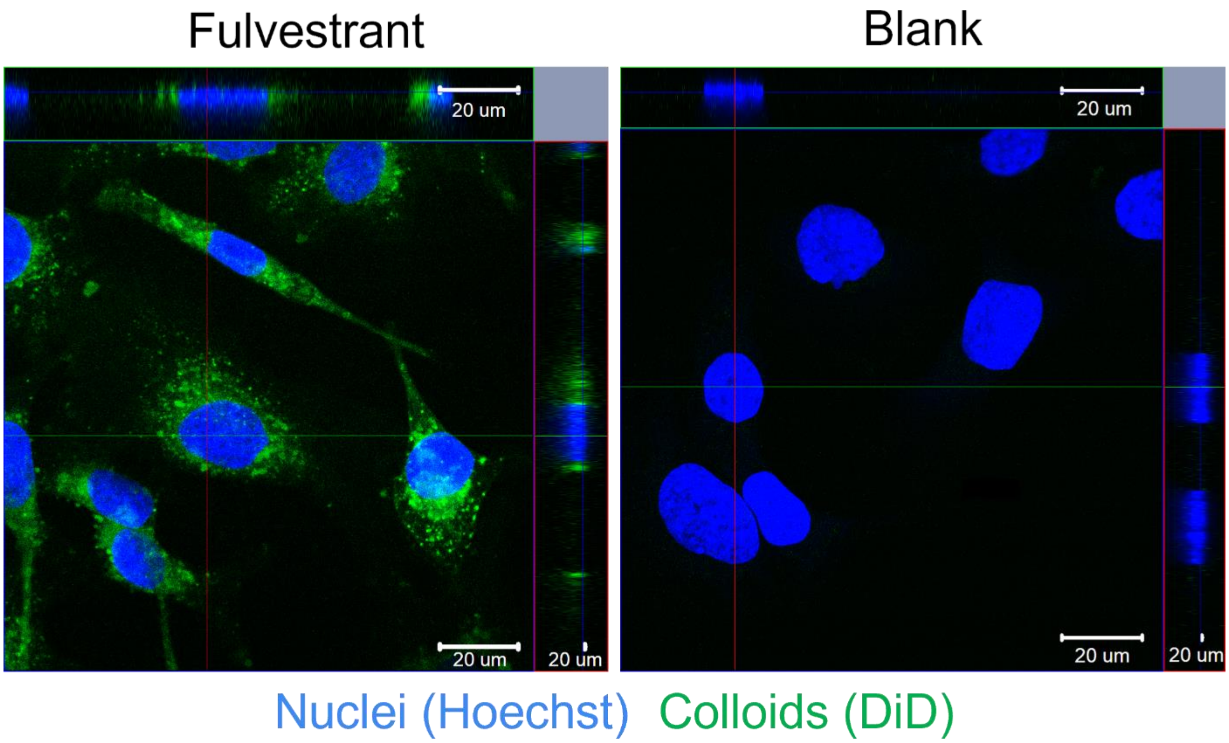

Figure S6. Visualization of colloid uptake by confocal microscopy. SKOV3 cells were treated for 3 h with 5 μM of fulvestrant formulated with DSPC and DMG-PEG2000 and tagged with DiD. The cells were fixed, stained, and imaged (nuclei, blue; colloids, green).

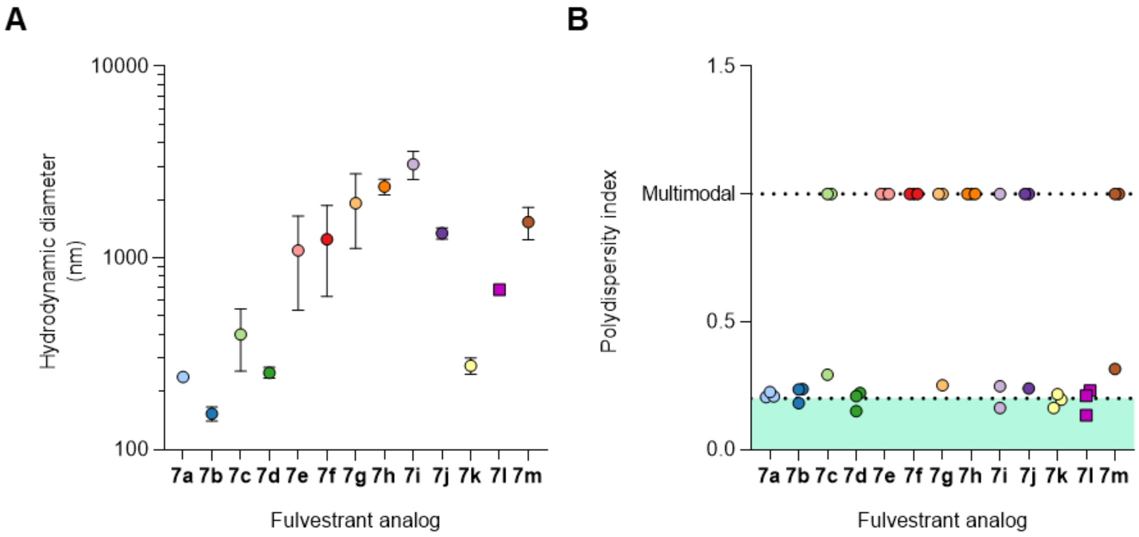

Figure S7. Fulvestrant analog colloids are not uniformly stable in PBS. **A** Hydrodynamic diameter of non-stabilized fulvestrant analog colloidal drug aggregates immediately after formulation (n = 3, mean ± SD). **B** Polydispersity index (PDI) of non-stabilized fulvestrant analog colloids immediately after formulation. A PDI value of less than 0.2 is generally considered to indicate a narrow size distribution; a multimodal PDI indicates the presence of both flocs and colloids.

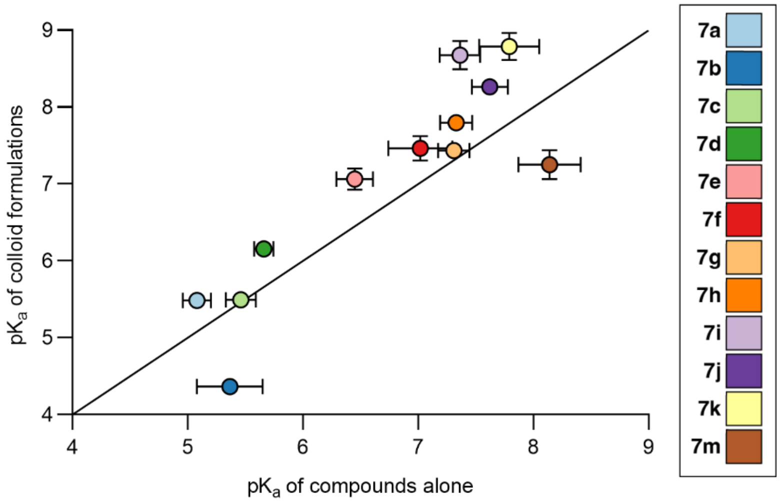

Figure S8. pK_a values of colloidal fulvestrant analog formulations compared to compounds alone. Fulvestrant analogs were prepared with 20 µM analog, 4 µM DSPC, and 0.6 µM DMG-PEG2000. pK_a values were determined by fitting a sigmoidal curve to TNS fluorescence vs. pH data as described in Figure S1 (n = 3, mean ± SEM).

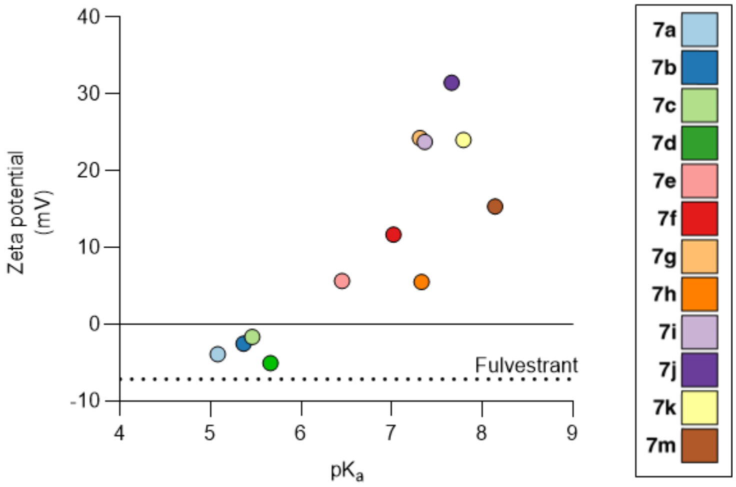

Figure S9. Zeta potential characterization of fulvestrant analog colloids as function of pK_a. Colloids were formulated with 50 µM fulvestrant analog, 10 µM DSPC, and 1.5 µM DMG-PEG 2000. Each data point represents the average of three technical replicates.

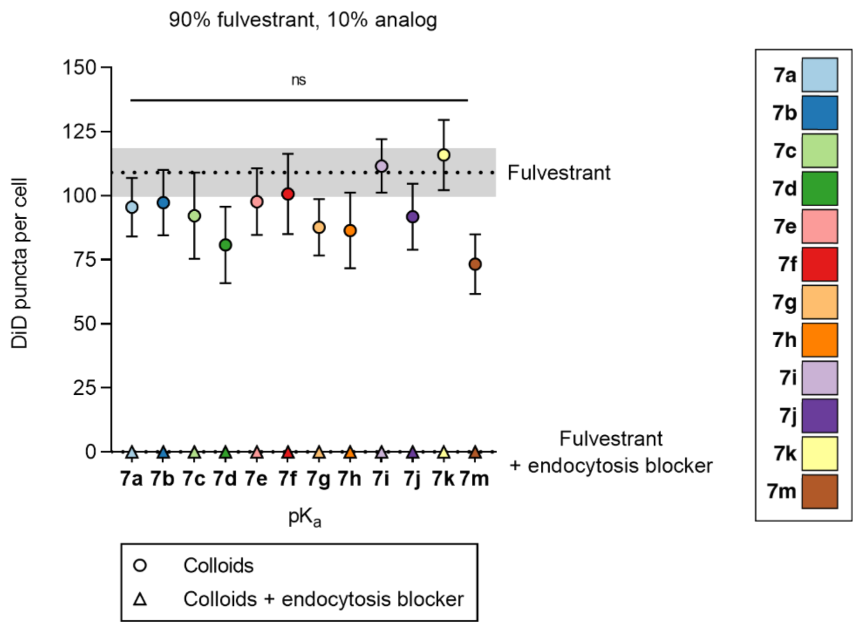

Figure S10. Quantification of fulvestrant analog colloid uptake in SKOV3 cells. Number of DiD puncta per cell as a function of ionizable fulvestrant analog pK_a. All 10% ionizable colloids were endocytosed to a similar degree as fulvestrant colloids. Endocytosis was blocked using 20 µM hydroxydynasore ($N \geq 9$, mean ± SEM, Brown-Forsythe and Welch ANOVA tests comparing all groups without endocytosis blocker to fulvestrant, ns $p > 0.05$).

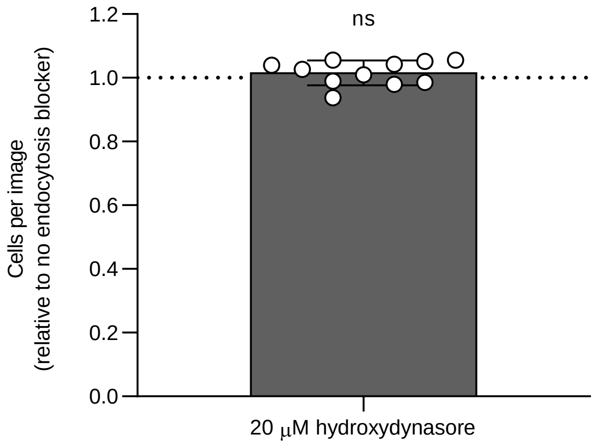

Figure S11. Quantification of cell number following treatment with the endocytosis blocker and colloidal formulations. No meaningful change in the number of cells per image, which would suggest toxicity, was observed following treatment with 20 µM hydroxydynasore for 3.5 h and 5 µM colloidal formulations for 3 h. Images were collected from fixed locations within each well, so the number of cells in each image is not subject to operator bias. Cell numbers were normalized to wells not treated with the endocytosis blocker, and then these ratios were averaged across the entire plate. Each data point represents one such replicate (ns $p > 0.05$, one-sample t-test comparing the results to unity).

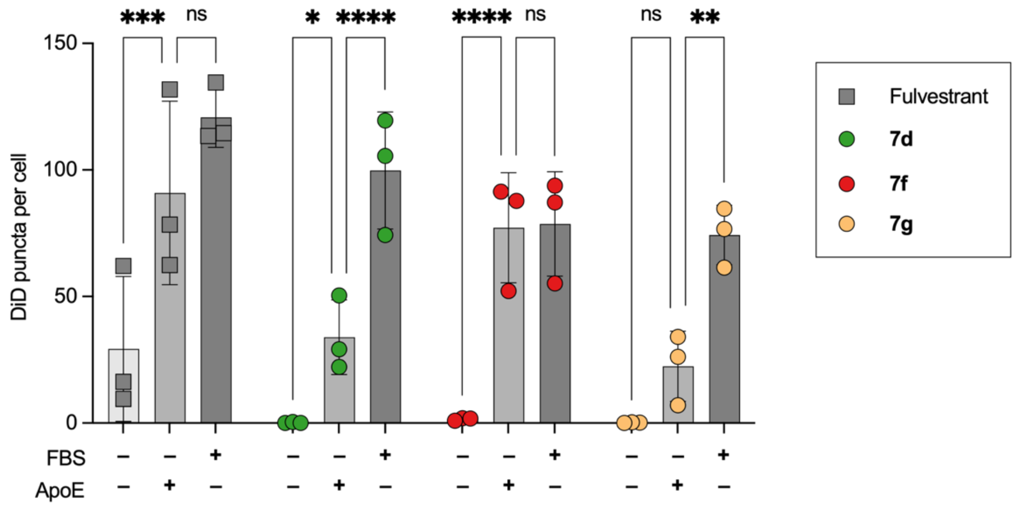

Figure S12. Effect of serum and apolipoprotein E on colloid endocytosis. Number of DiD puncta (each containing one or more endocytosed colloids) per SKOV3 cell after 3 h of incubation with 5 μM of fulvestrant or analog formulated with DSPC, DMG-PEG2000, and DiD. Before treatment, the cell media were washed with HBSS and the media was replaced with fresh RPMI 1640 with or without either 10% FBS (+ / – FBS) or 1.5 μg/mL ApoE (+ / – ApoE). Colloids were imaged using a widefield fluorescence microscope with a large depth of field (N = 3, mean ± SD, two-way ANOVA with Dunnett's post-hoc test comparing among treatments for each fulvestrant/analog colloid, ns $p > 0.05$, *$p < 0.05$, **$p < 0.01$, ***$p < 0.001$).

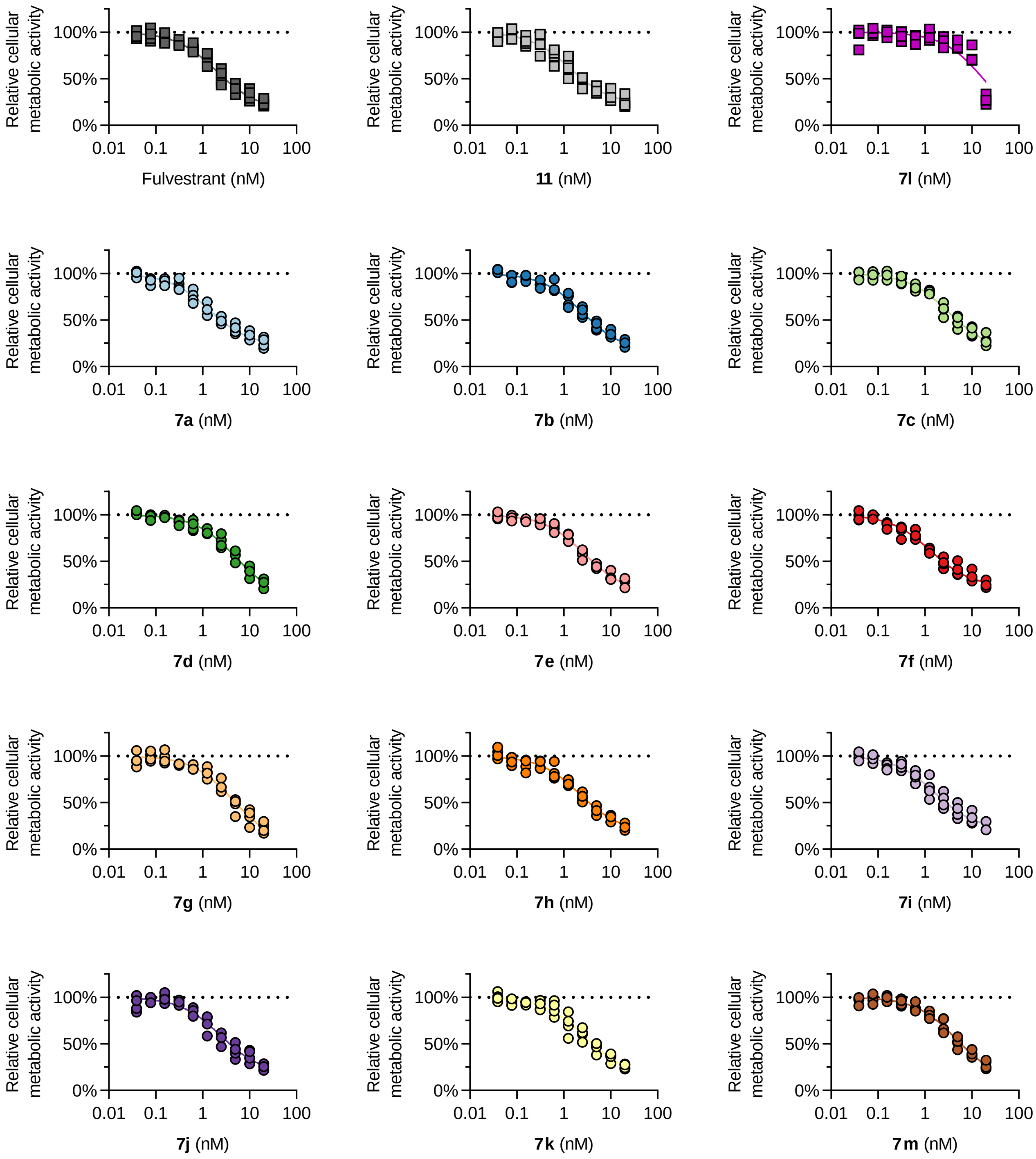

Figure S13. Dose-response curves showing total MCF7 cellular metabolic activity as a function of drug concentration after 6 d of treatment. Each data point represents the average of 3 technical replicates.

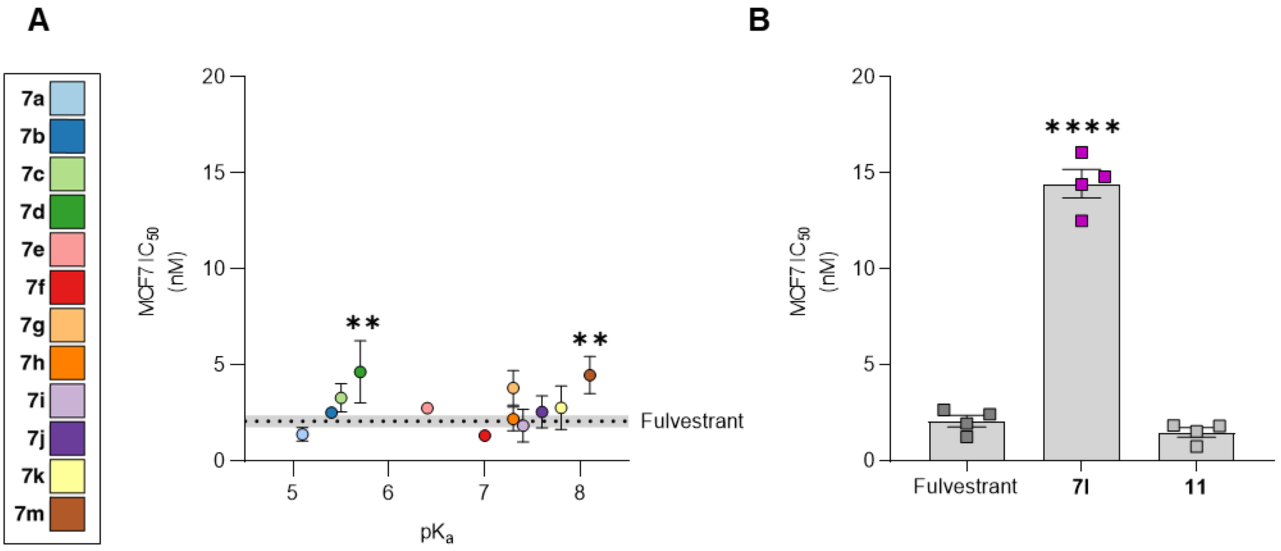

Figure S14. Ionizable fulvestrant analogs are active against estrogen receptor-positive MCF7 breast cancer cells. **A** IC_{50} values of ionizable fulvestrant analogs (n = 4 biological replicates, one-way ANOVA with Dunnett post-hoc test comparing all groups to fulvestrant, $^{**}p < 0.01$). **B** IC_{50} values of fulvestrant and non-ionizable fulvestrant analogs, **7l** and **11** (n = 4 biological replicates, one-way ANOVA with Tukey's post-hoc tests comparing all groups, $^{****}p < 0.0001$).

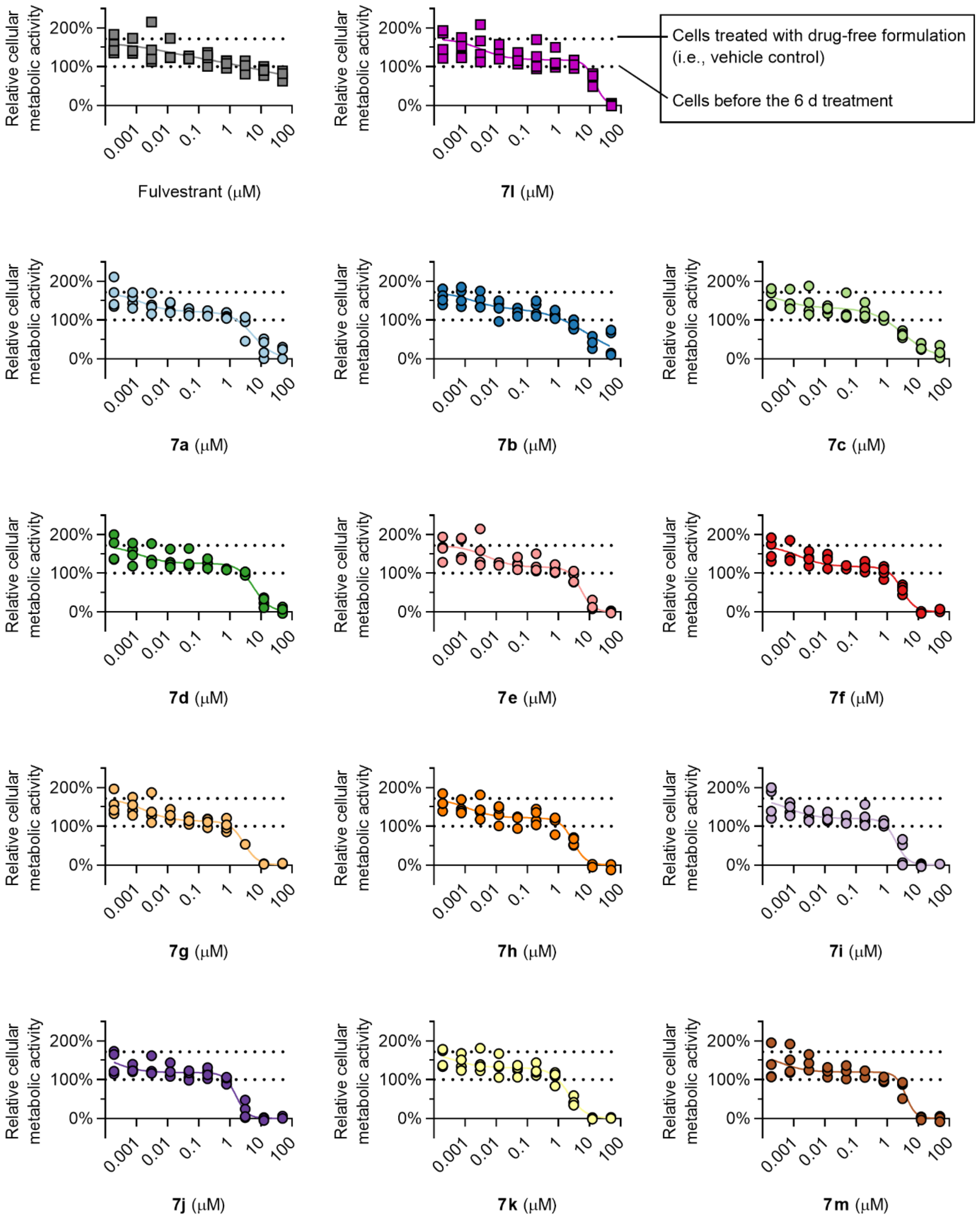

Figure S15. Concentration-response curves showing total BT474 cellular metabolic activity as a function of drug concentration after being treated with colloids for 6 d. The values were normalized to the pre-treatment signals (dotted line at 100%) on a per-well basis to measure proliferation over the course of the experiment. Formulations were prepared with 200 μM fulvestrant equivalent in PBS and then diluted to the final concentration in cell culture media. Thus, all groups contained 0.5% (v v^{-1}) PBS, 200 nM DSPC, and 30 nM DMG-PEG 2000 per μM drug. The dotted line at 170% represents the metabolic activity of cells treated with a drug-

free (i.e., lipid excipients in PBS) formulation. Each data point represents the average of 3 technical replicates.

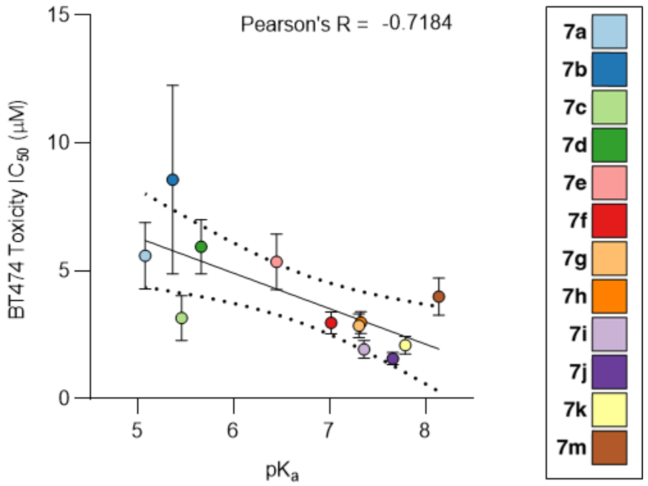

Figure S16. Toxicity has a positive correlation with pK_a of ionizable fulvestrant analogs, meaning that analogs with higher pK_a values are generally more toxic. Toxicity IC_{50} values were calculated from BT474 cell metabolic activity dose-response curves (Figure S15).

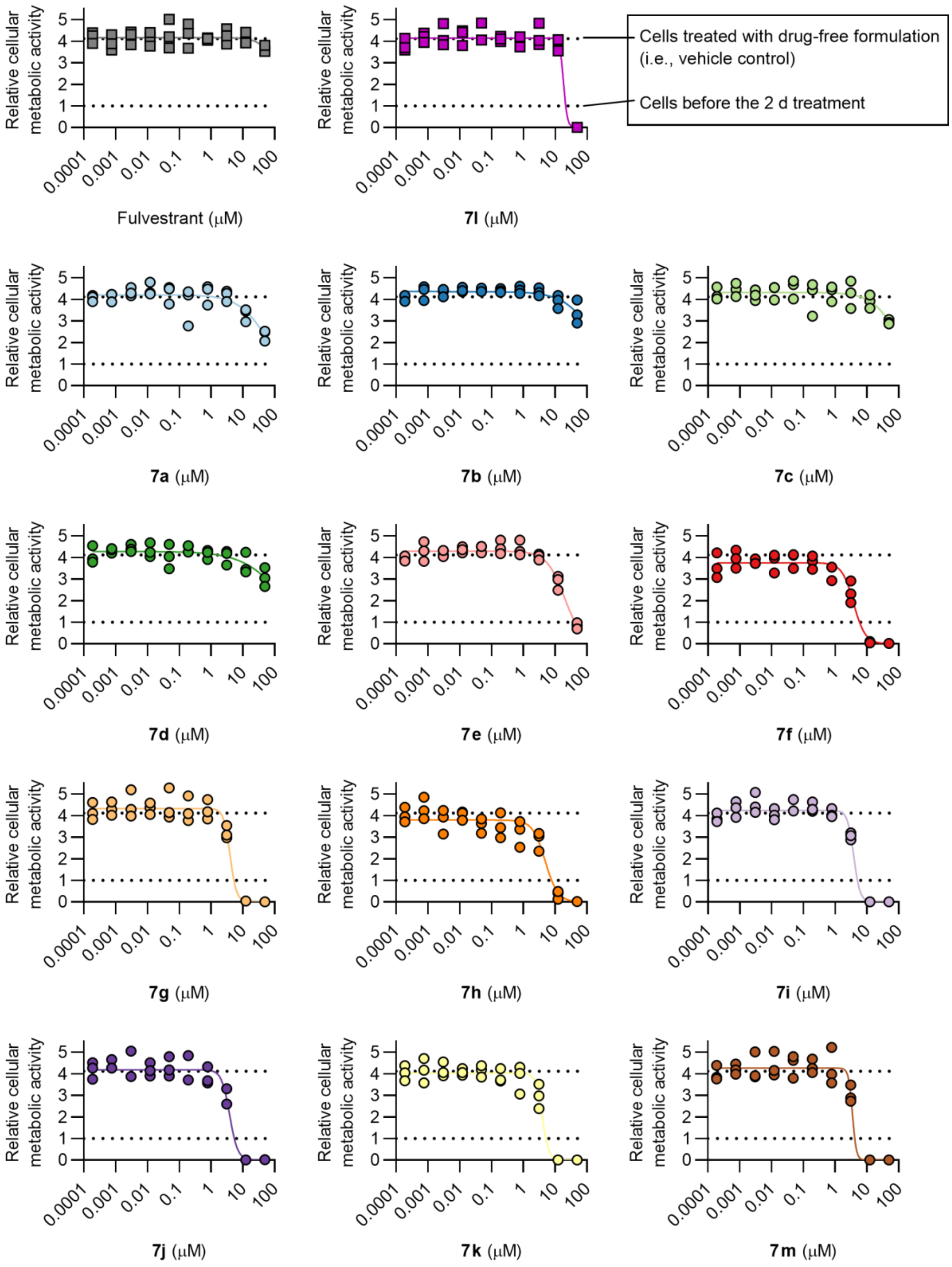

Figure S17. Concentration-response curves showing total lung fibroblast cellular metabolic activity as a function of drug concentration after being treated with colloids for 2 d. The values were normalized to the pre-treatment signals (dotted line at 1) on a per-well basis to measure proliferation over the course of the experiment. Formulations were prepared with 200 μM fulvestrant equivalent in PBS and then diluted to the final concentration in cell culture media. Thus, all groups contained 0.5% (v/v) PBS, 200 nM DSPC, and 30 nM DMG-PEG2000 per μM drug. The dotted line at 4 represents the metabolic activity of cells treated with a drug-free (i.e., lipid excipients in PBS) formulation. Each data point represents the average of 3 replicates.

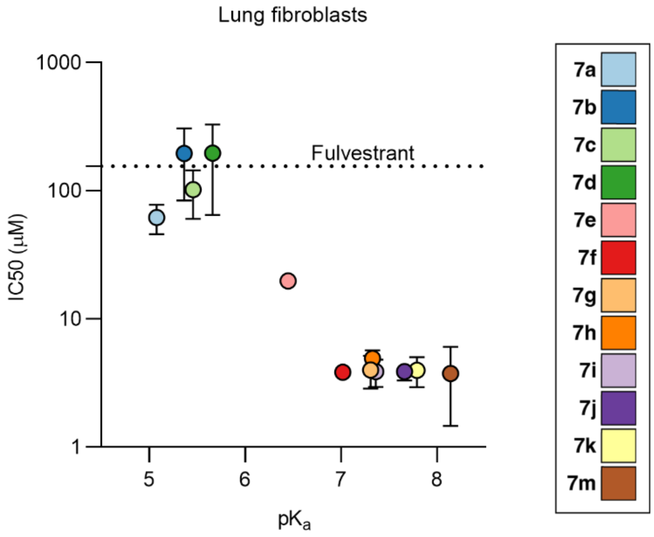

Figure S18. Higher pK_a of ionizable fulvestrant analogs are generally more toxic to lung fibroblasts. IC_{50} values were calculated from cell metabolic activity dose-response curves (Figure S17).

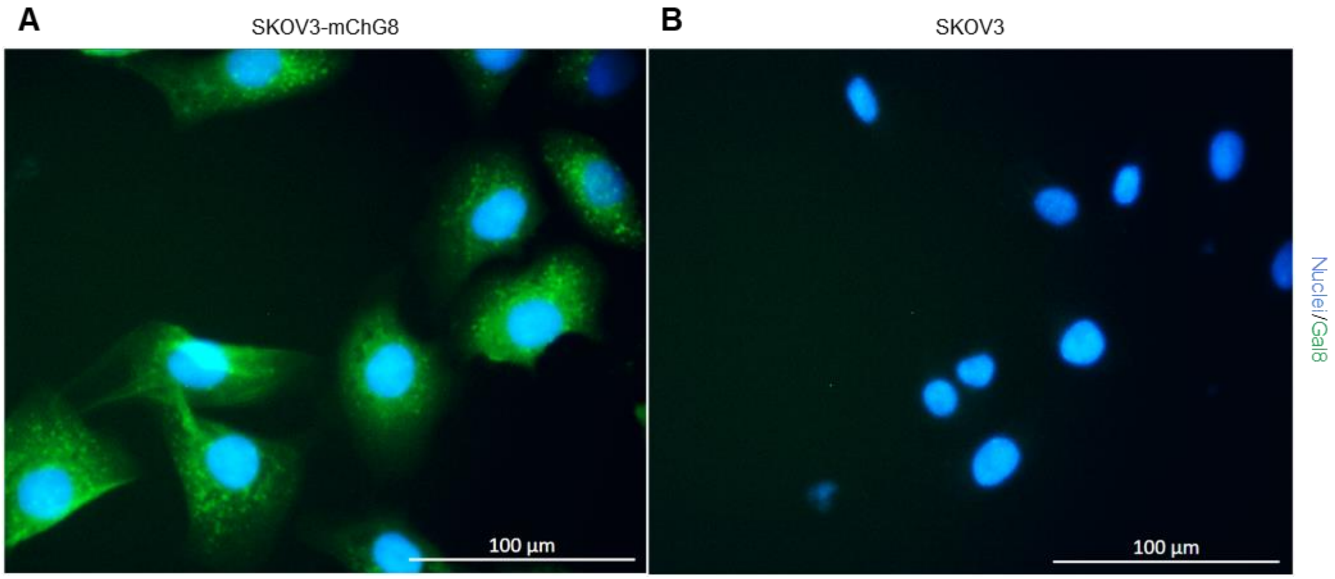

Figure S19. Characterization of the fluorescent Gal8-expressing reporter cell line (SKOV3-mChG8). **A** The reporter cells exhibit cytosolic fluorescence in the mCherry channel whereas **B** the parent cells do not. Note that these images were not processed to remove diffuse mCherry-Gal8 fluorescence, unlike most of the other images.

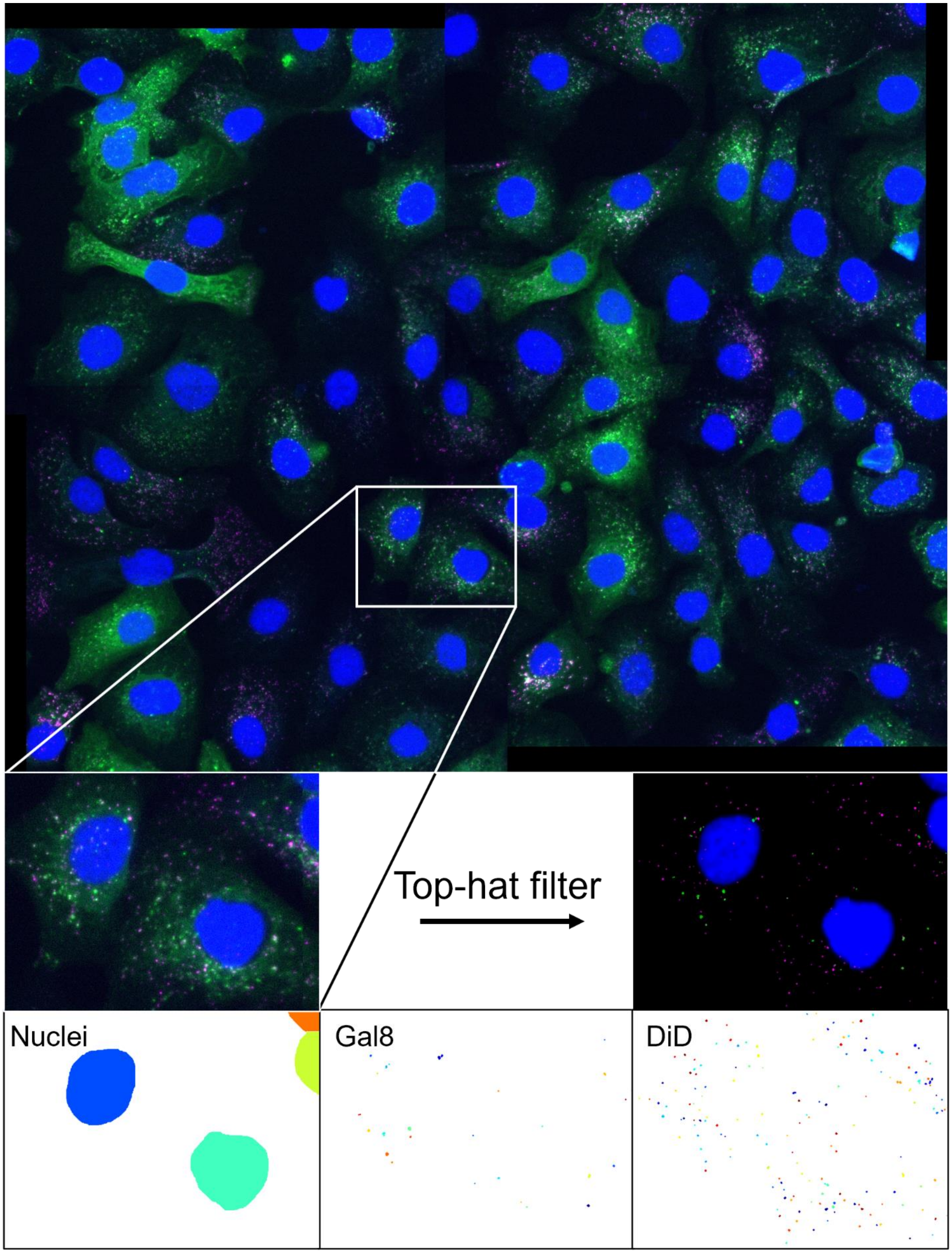

Figure S20. Representative image depicting the processing procedure used to quantify its features. SKOV3-mChG8 cells were treated with a colloidal formulation containing 5 μM of analog **7e** for 3 h. Four wide-field fluorescence fields of view were stitched together to form a single large image, which was then filtered to remove diffuse background fluorescence. Then, nuclei, Gal8 foci, and DiD puncta were segmented into distinct regions (to counteract partial overlaps) and counted.

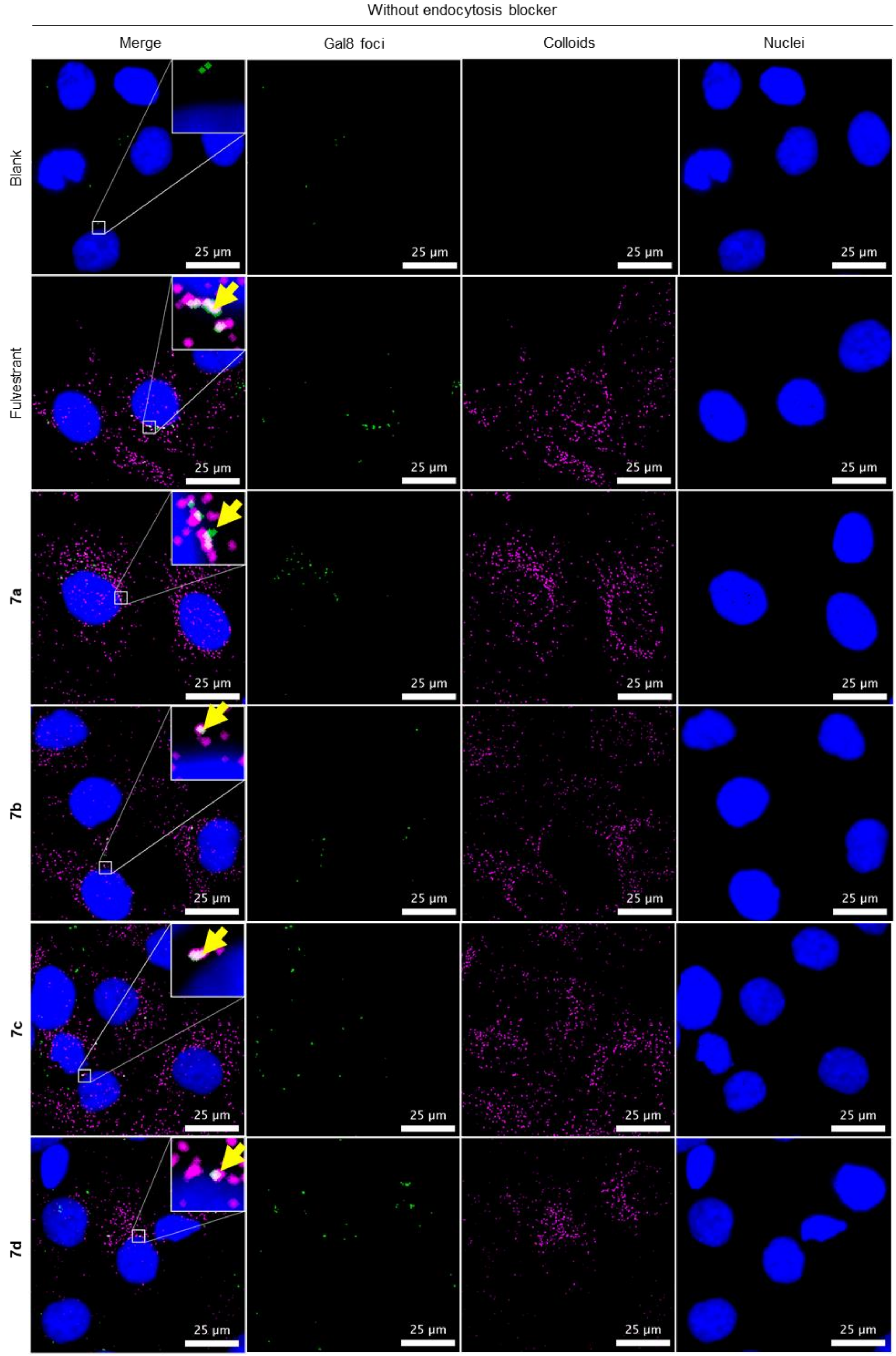
Without endocytosis blocker
Merge
Gal8 foci
Colloids
Nuclei
Blank
Fulvestrant
7a
7b
7c
7d
25 μm

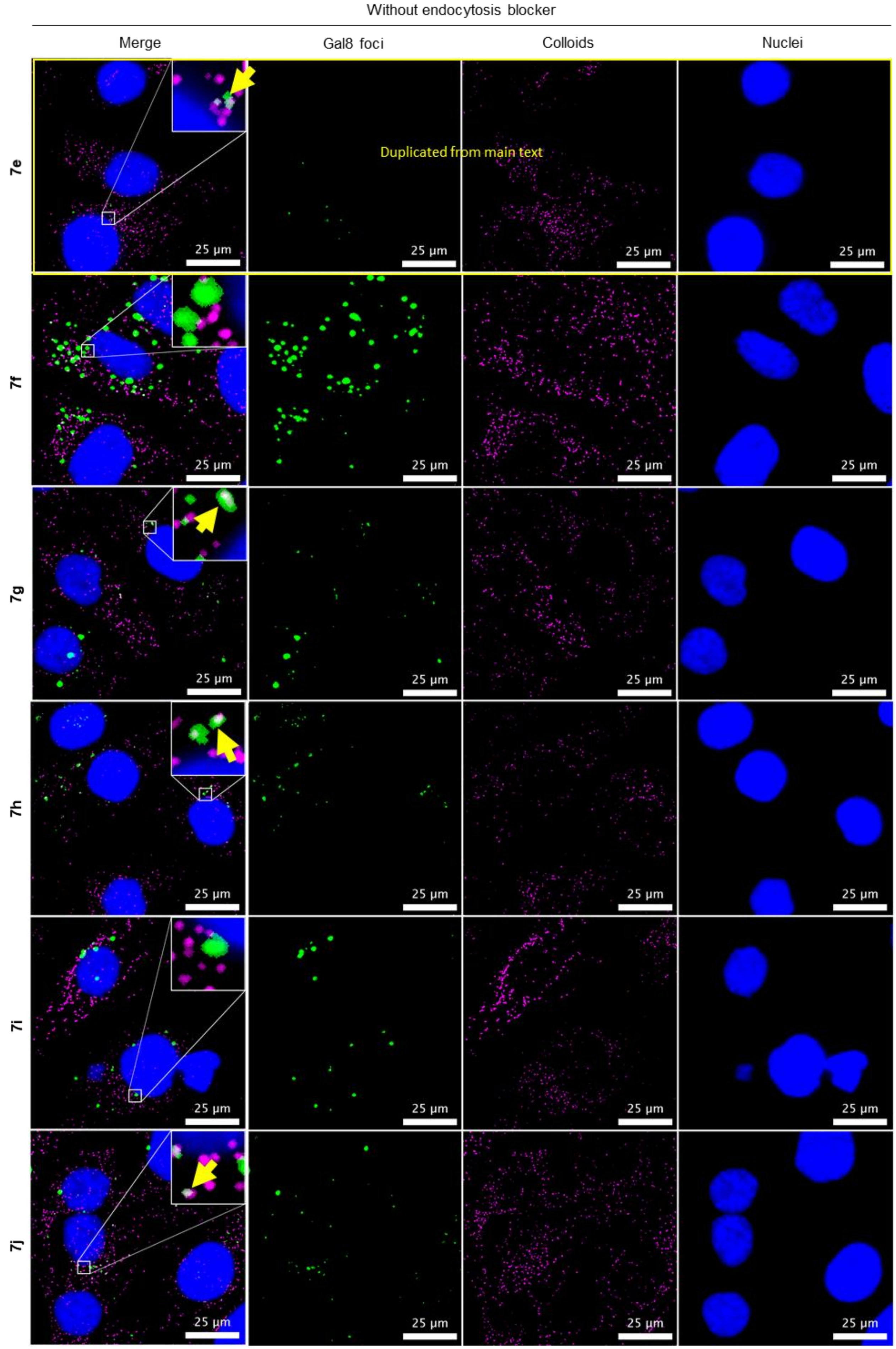
Without endocytosis blocker
Merge
Gal8 foci
Colloids
Nuclei
7e
7f
7g
7h
7i
7j
Duplicated from main text
25 μm

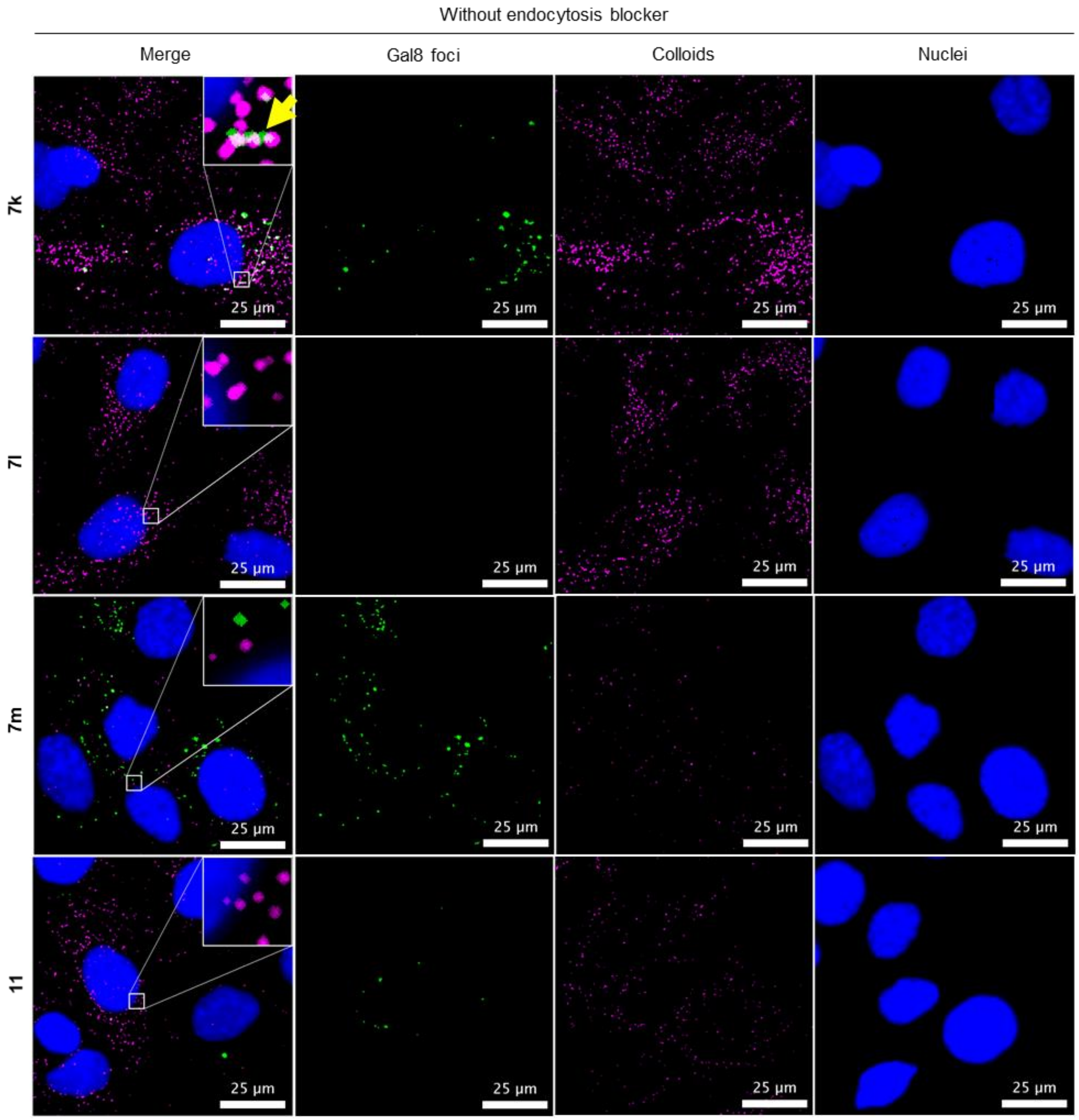

Figure S21. Sample fluorescence images of SKOV3-mChG8 cells after 3 h of treatment with colloidal formulations containing 5 µM fulvestrant or fulvestrant analog. Galectin 8 (mCherry-Gal8) foci are shown in green, colloids (DiD puncta) are shown in magenta, and nuclei (Hoechst) are shown in blue.

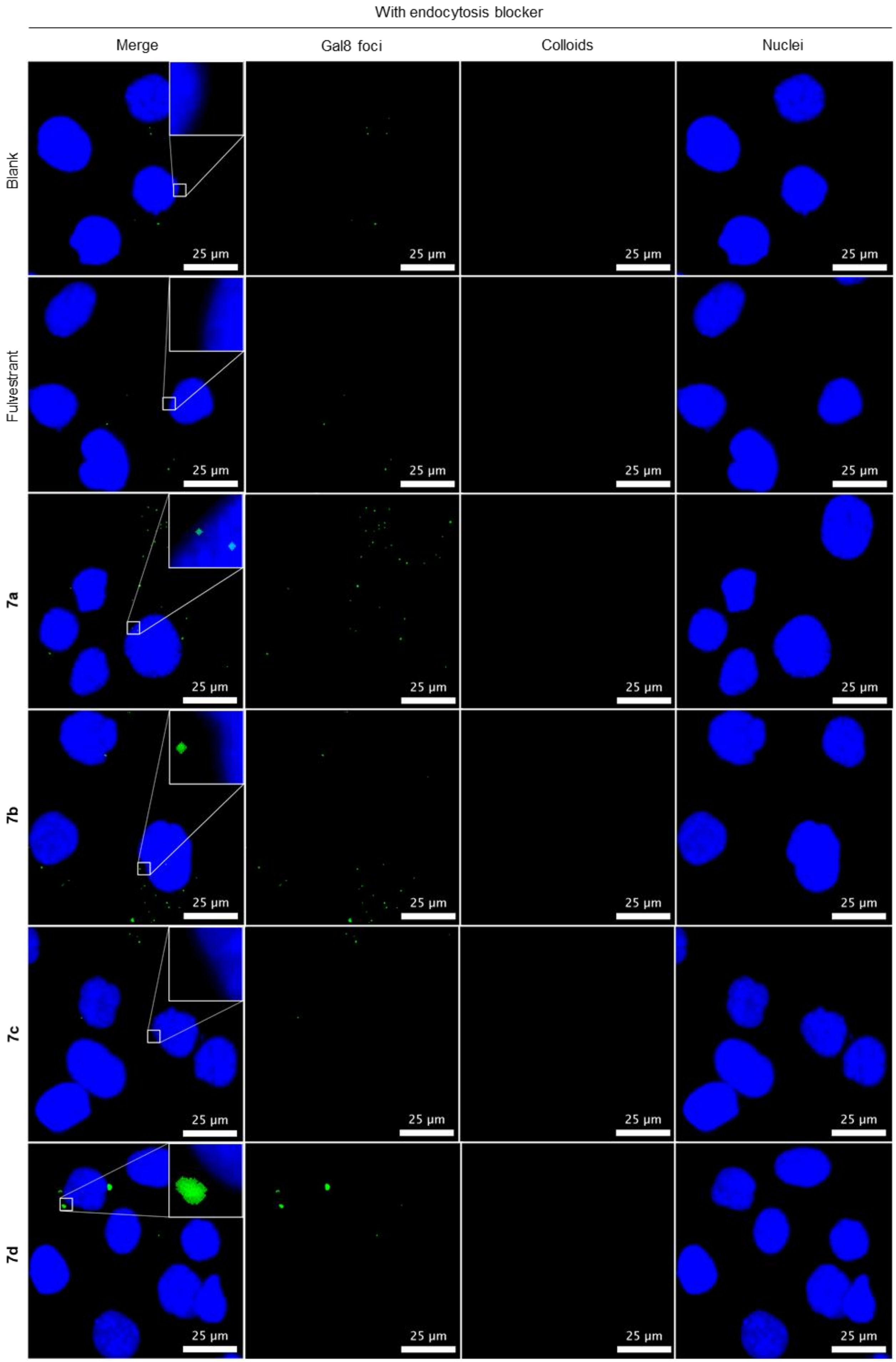
With endocytosis blocker
Merge
Gal8 foci
Colloids
Nuclei
Blank
Fulvestrant
7a
7b
7c
7d
25 μm

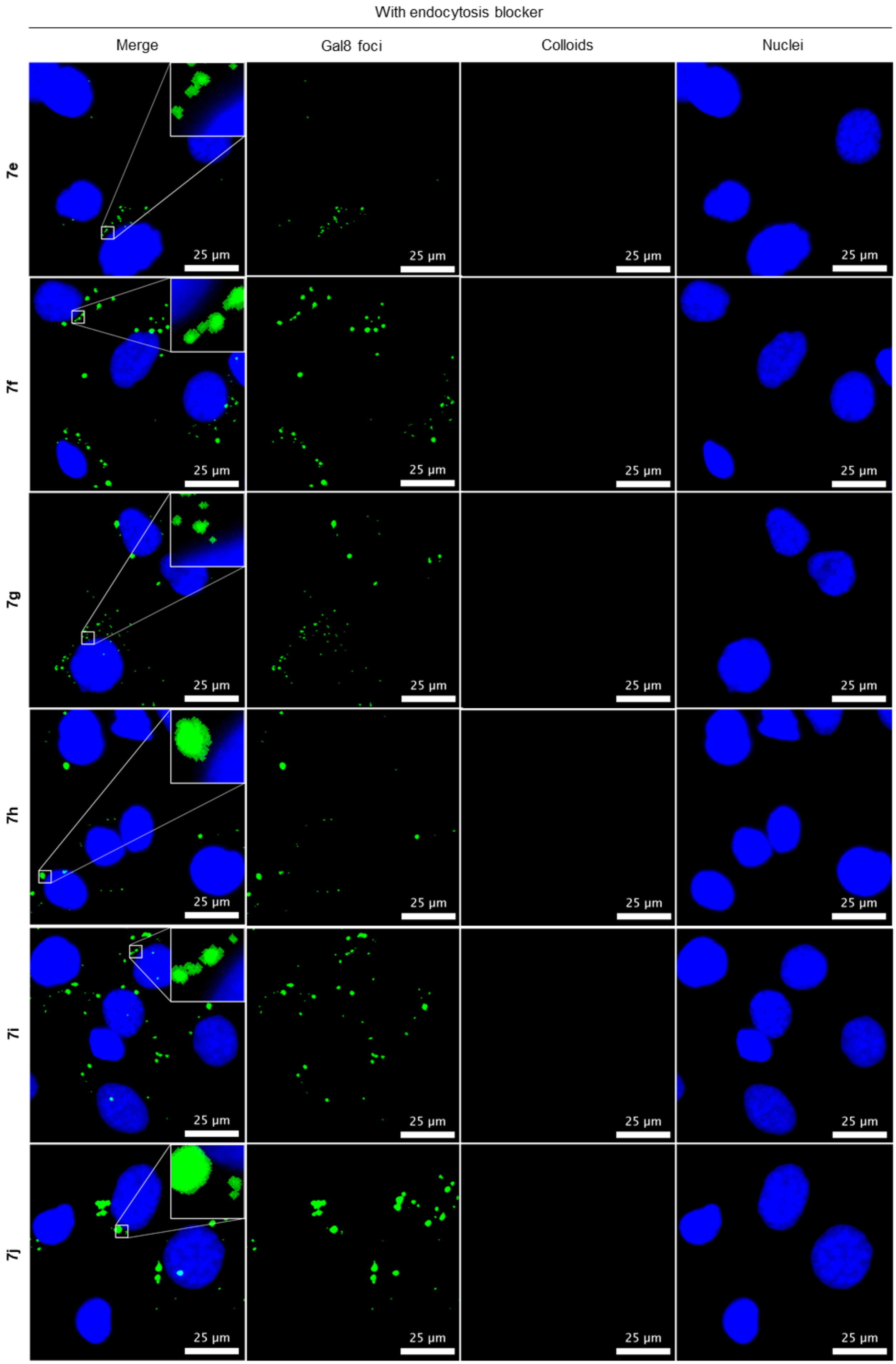

With endocytosis blocker
Merge
Gal8 foci
Colloids
Nuclei
7e
7f
7g
7h
7i
7j
25 µm

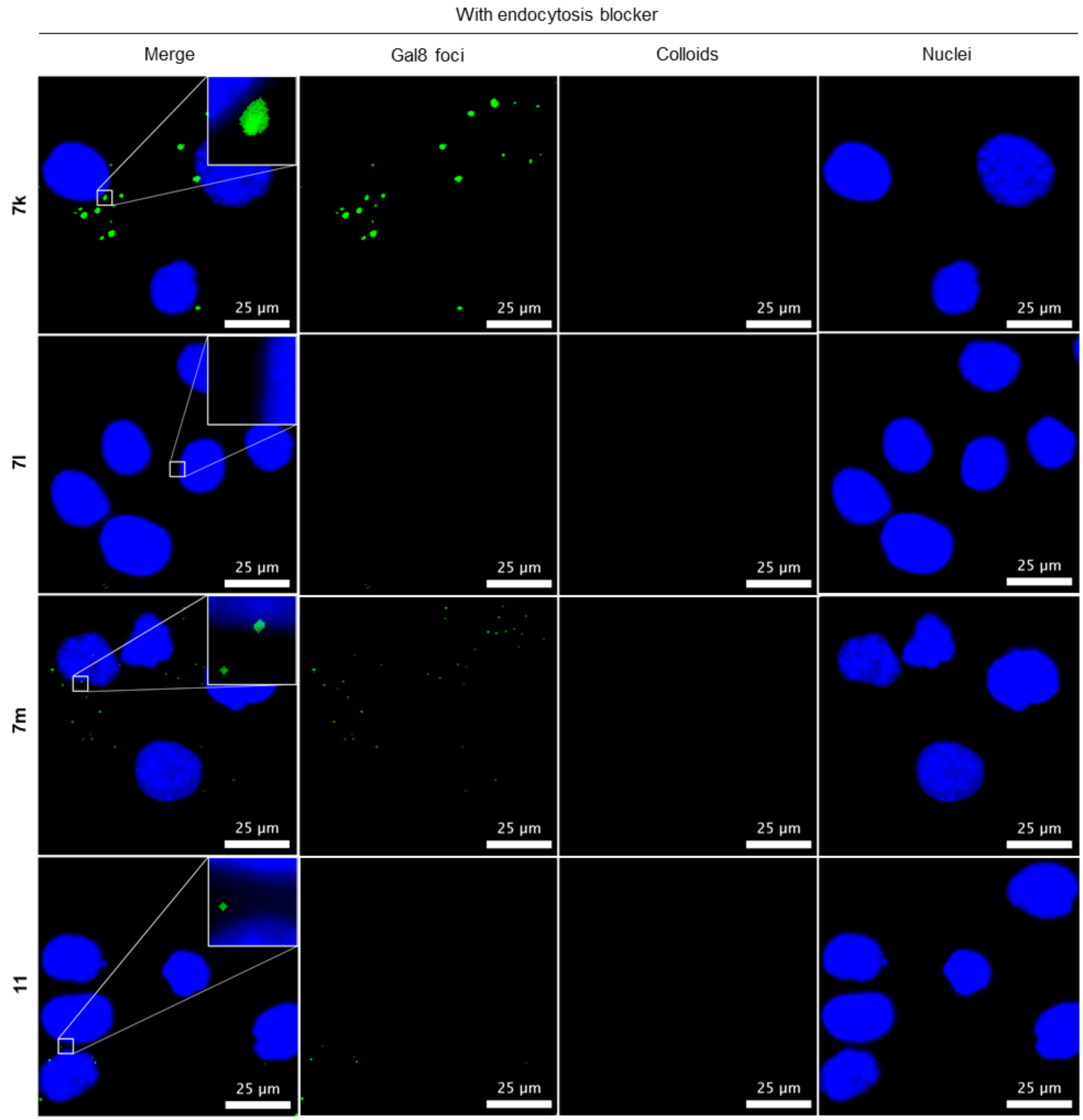

Figure S22. Sample fluorescence images of SKOV3-mChG8 cells after 3 h of treatment with colloidal formulations containing 5 µM fulvestrant or fulvestrant analog. The cells were pre-treated with 20 µM hydroxy-dynasore for 30 min prior to the addition of the colloids. Galectin 8 (mCherry-Gal8) foci are shown in green, colloids (DiD puncta) are shown in magenta, and nuclei (Hoechst) are shown in blue.

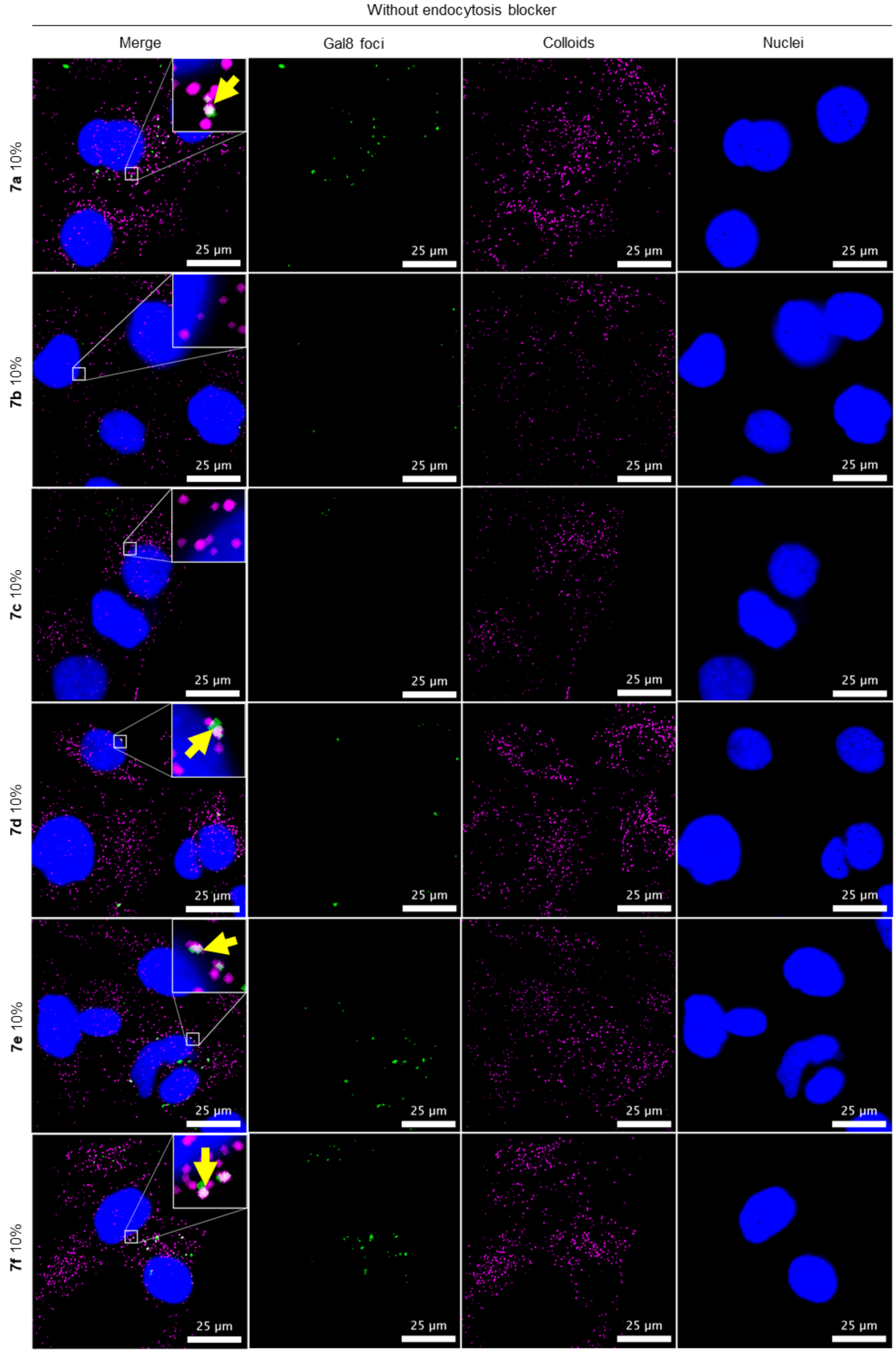
Without endocytosis blocker
Merge
Gal8 foci
Colloids
Nuclei
7a 10%
7b 10%
7c 10%
7d 10%
7e 10%
7f 10%
25 µm

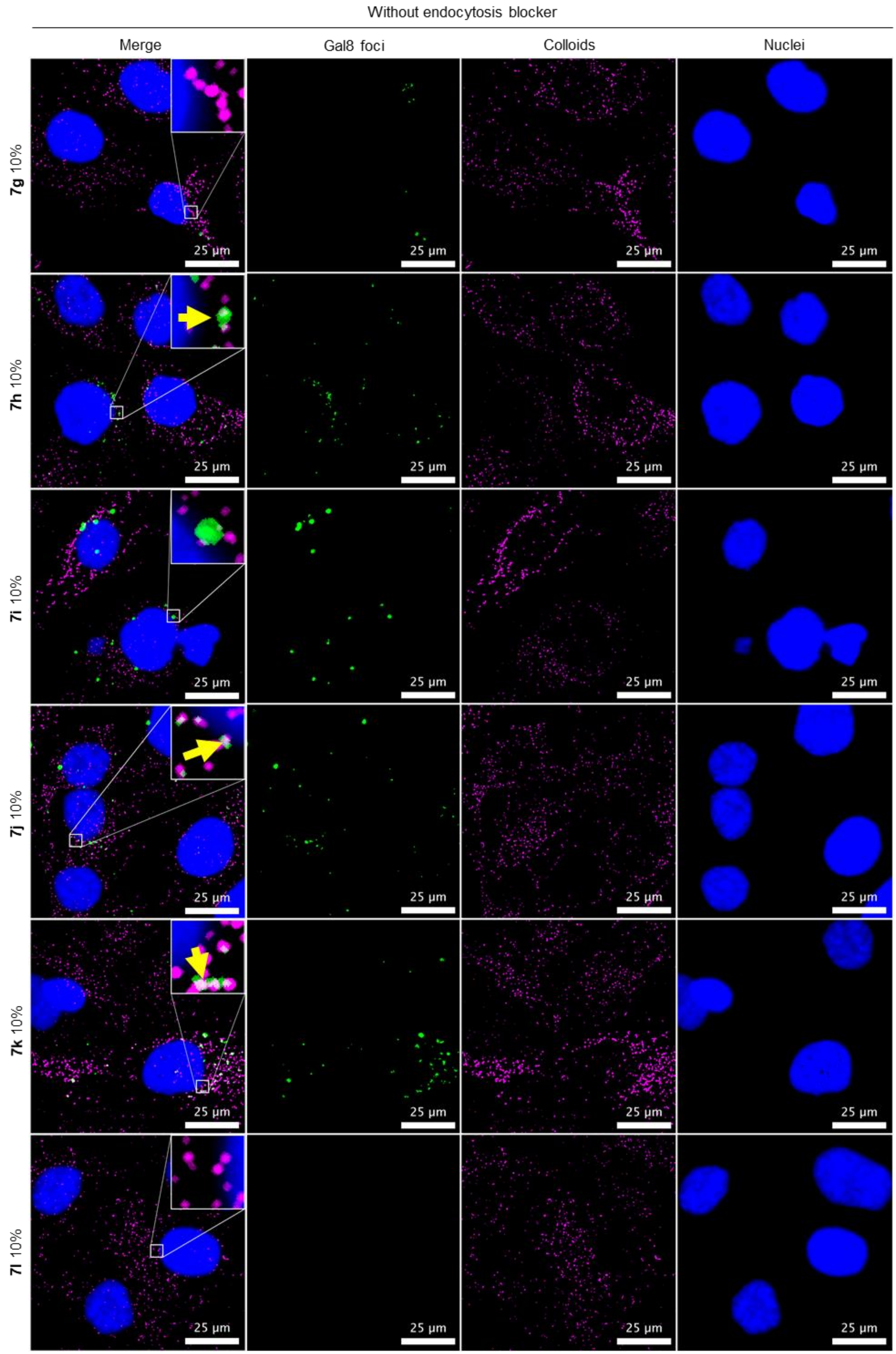

Without endocytosis blocker
Merge
Gal8 foci
Colloids
Nuclei
7g 10%
7h 10%
7i 10%
7j 10%
7k 10%
7l 10%
25 µm

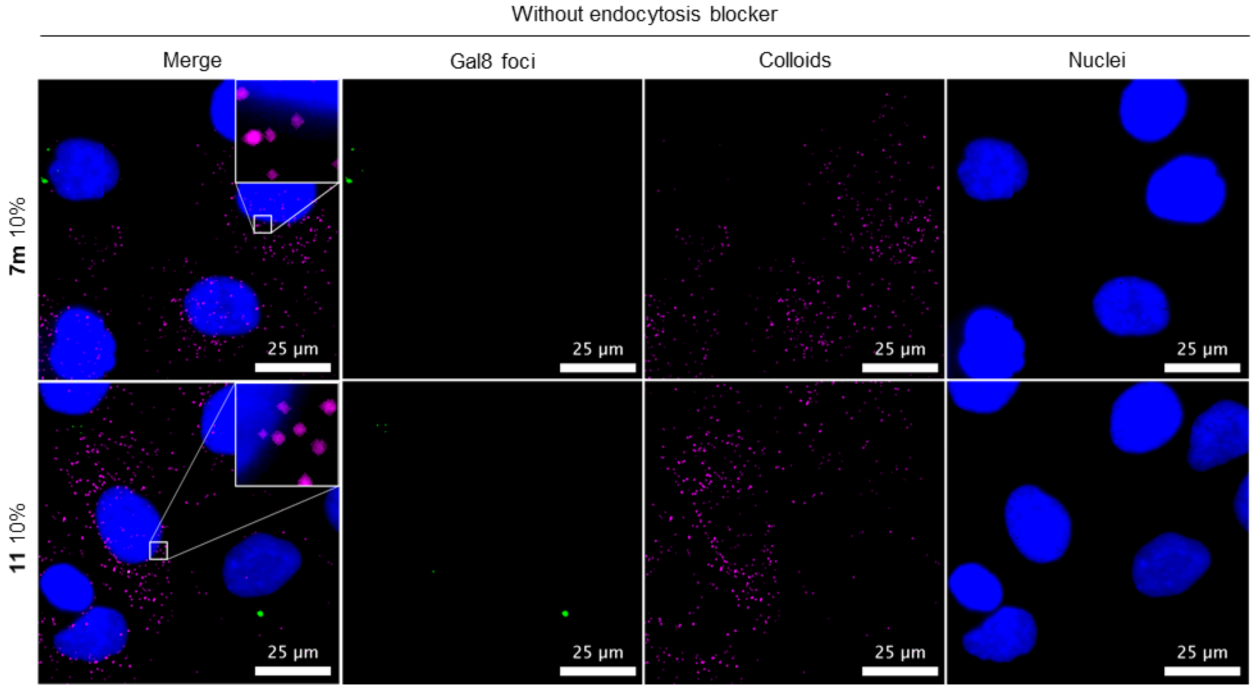

Figure S23. Sample fluorescence images of SKOV3-mChG8 cells after 3 h of treatment with colloidal formulations containing 5 µM fulvestrant or fulvestrant/analog mixture. Galectin 8 (mCherry-Gal8) foci are shown in green, colloids (DiD puncta) are shown in magenta, and nuclei (Hoechst) are shown in blue. “10%” indicates fulvestrant colloids with 10 mol% of the relevant analog (and 90 mol% fulvestrant).

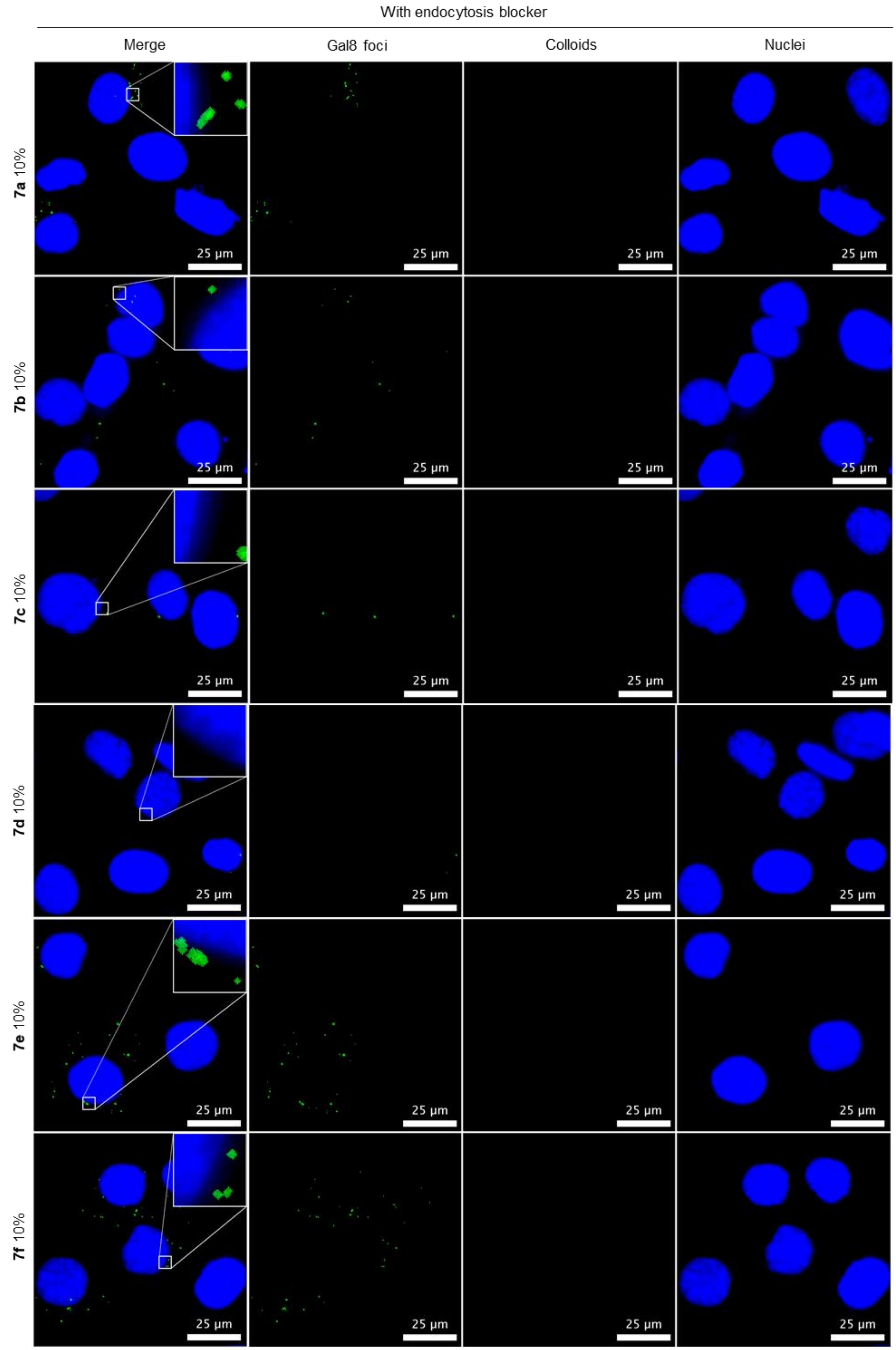
With endocytosis blocker
Merge
Gal8 foci
Colloids
Nuclei
7a 10%
7b 10%
7c 10%
7d 10%
7e 10%
7f 10%
25 µm

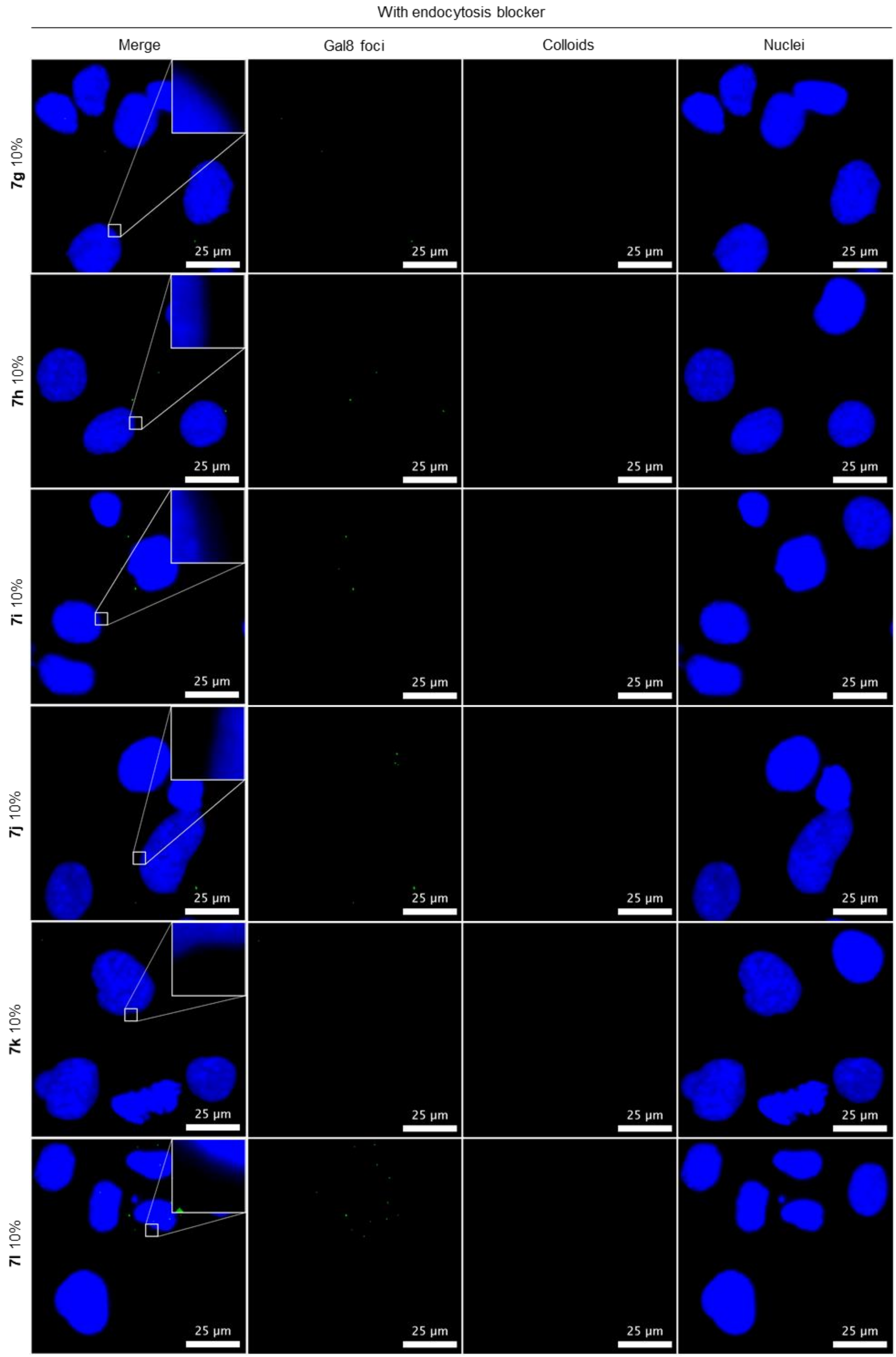

With endocytosis blocker
Merge
Gal8 foci
Colloids
Nuclei
7g 10%
7h 10%
7i 10%
7j 10%
7k 10%
7l 10%
25 µm

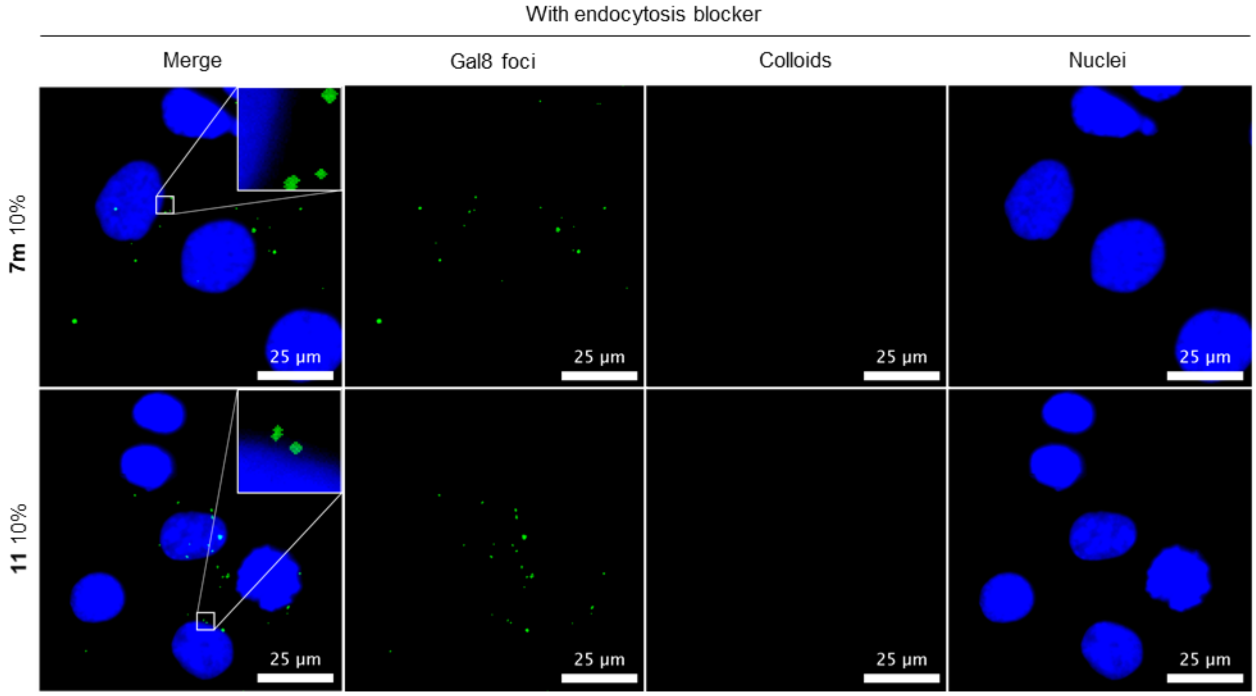

Figure S24. Sample fluorescence images of SKOV3-mChG8 cells after 3 h of treatment with colloidal formulations containing 5 µM fulvestrant or fulvestrant/analog mixture. The cells were pre-treated with 20 µM hydroxy-dynasore for 30 min prior to the addition of the colloids. Galectin 8 (mCherry-Gal8) foci are shown in green, colloids (DiD puncta) are shown in magenta, and nuclei (Hoechst) are shown in blue. “10%” indicates fulvestrant colloids with 10 mol% of the relevant analog (and 90 mol% fulvestrant).

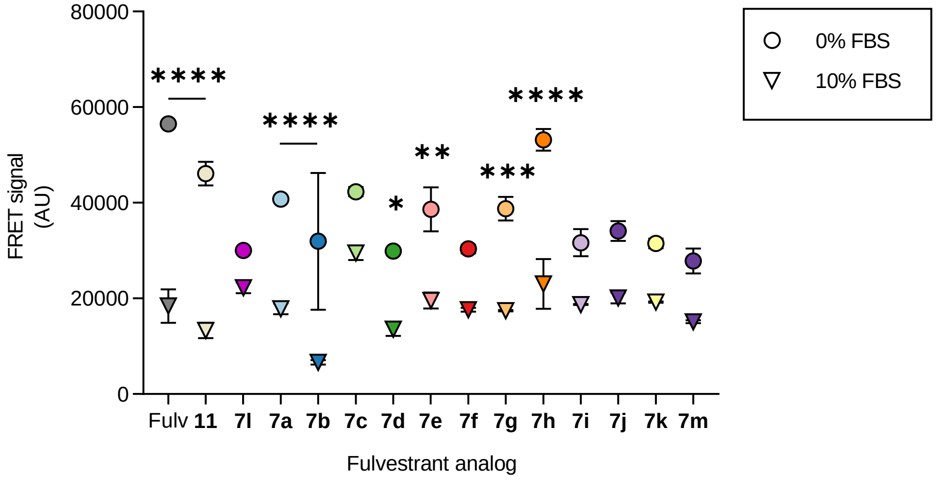

Figure S25. Serum proteins increase fulvestrant analog solubility. Colloids were formulated with 50 µM fulvestrant analog, 10 µM DSPC, 1.5 µM DMG-PEG 2000, 0.9 µM CholEsteryl BODIPY FL C12 (FRET donor), and 0.1 µM CholEsteryl BODIPY 542/563 C11 (FRET acceptor). Then, the colloids were diluted 10x into either PBS or media containing 10% FBS. The FRET signal represents the fluorescence of the acceptor (λ_{em} = 575 nm) following excitation of the donor (λ_{ex} = 490 nm). A higher FRET signal indicates greater numbers of intact colloids (n = 3 separate mixing events, mean ± SEM).

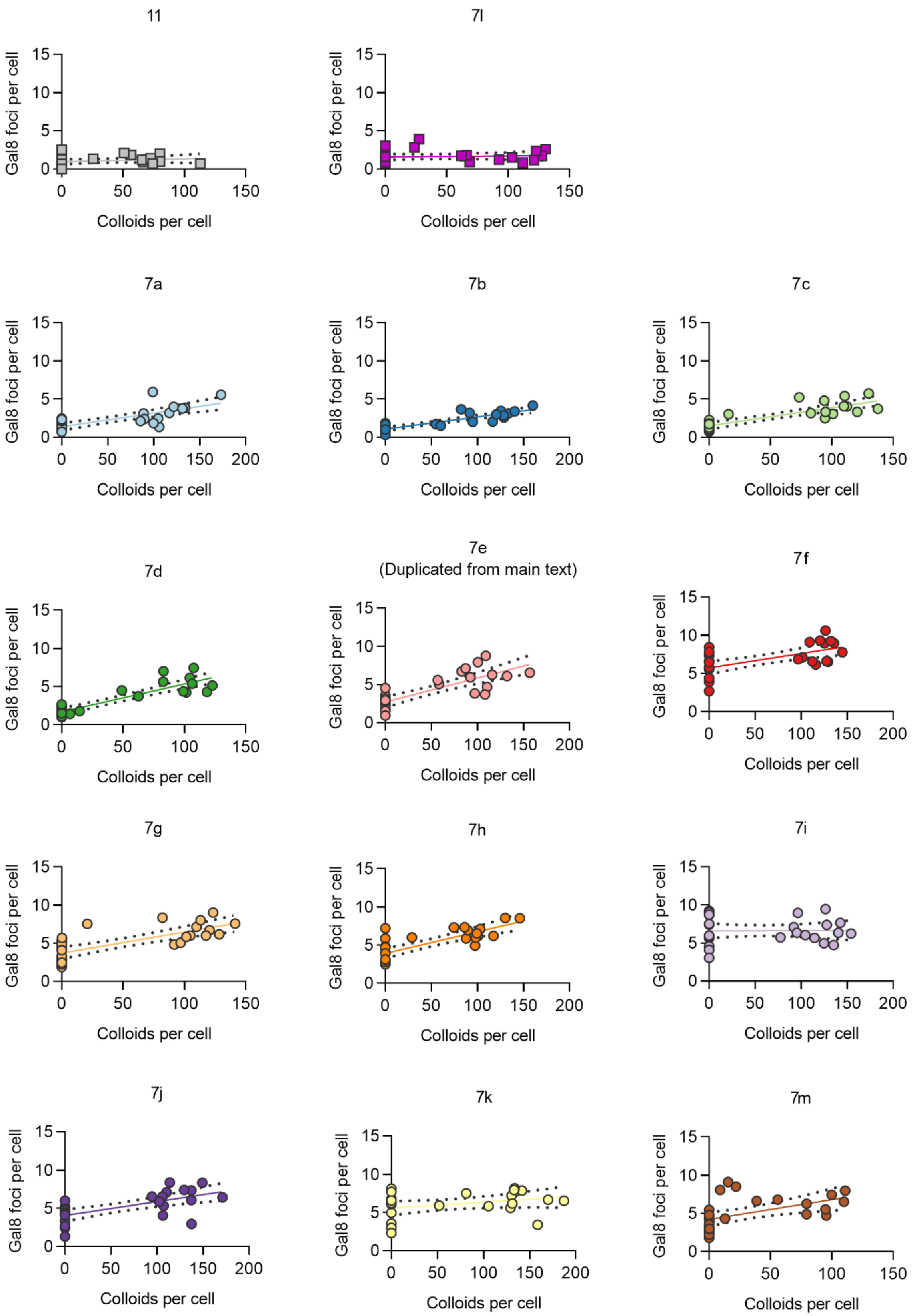
11
7l
7a
7b
7c
7d
7e
(Duplicated from main text)
7f
7g
7h
7i
7j
7k
7m
Gal8 foci per cell
Colloids per cell

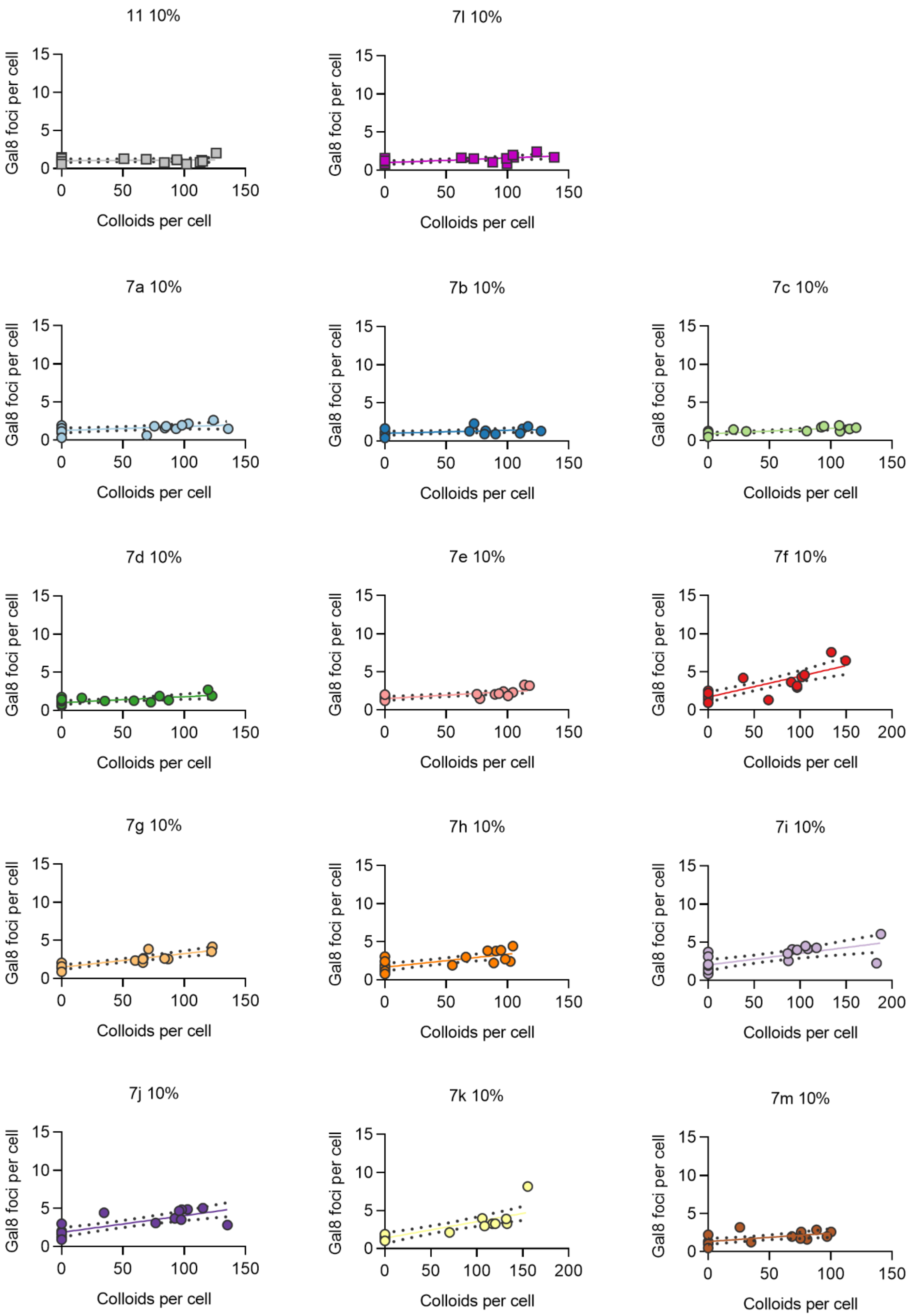

Figure S26. Image quantification of Gal8 foci and colloids per cell. The number of Gal8 foci correlated positively with the uptake ionizable fulvestrant analog colloids. Each data point represents the average of 3 technical replicates. “10%” indicates fulvestrant colloids with 10 mol% of the relevant analog (and 90 mol% fulvestrant).

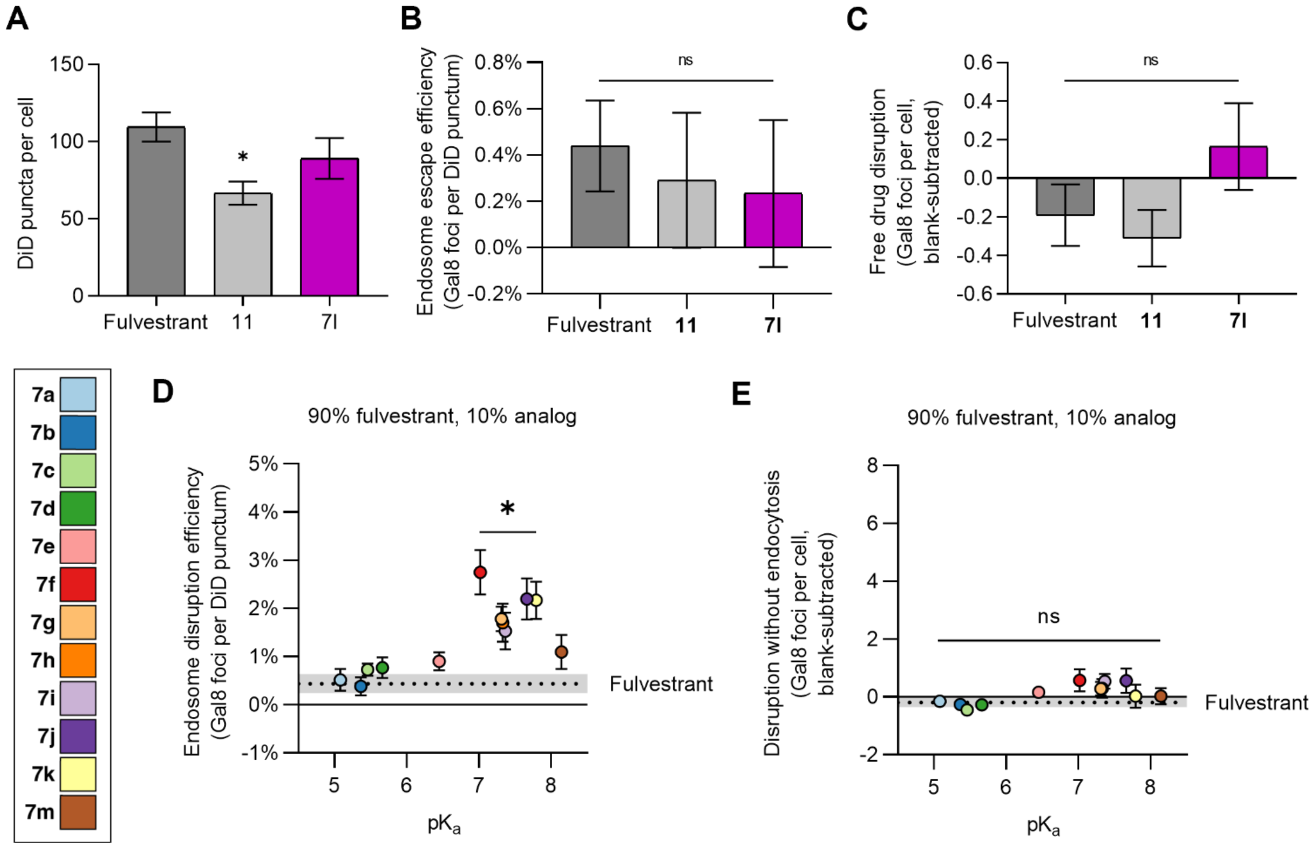

Figure S27. Characterization of uptake and endosomal disruption by non-ionizable colloids. **A** Colloids per cell, **B** endosome disruption efficiency, and **C** free drug disruption of fulvestrant and non-ionizable fulvestrant analog colloids. **D** Endosome disruption efficiency and **E** free drug disruption by 10% ionizable fulvestrant colloids as a function of ionizable fulvestrant analog pK_a. Statistics for all panels: n ≥ 9 biological replicates, mean ± SEM, Brown-Forsythe and Welch ANOVA tests with Dunnett T3 post-hoc test comparing each group to fulvestrant, *p < 0.05.

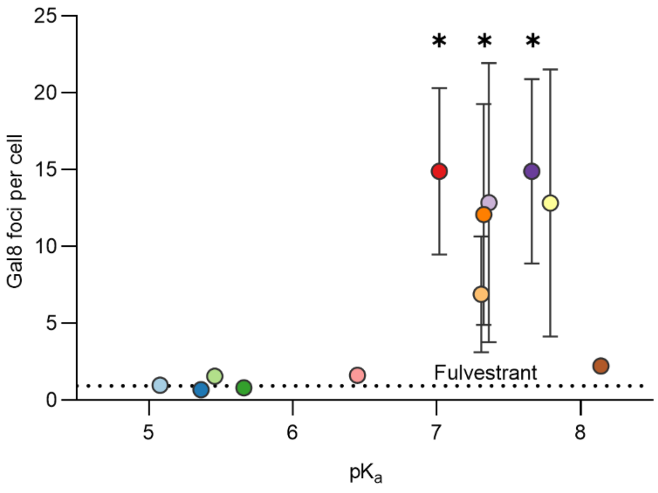

Figure S28. Gal8 foci quantification of cells treated with non-colloidal fulvestrant analog formulations (5 µM without excipients) for 24 h (n = 3, mean ± SEM, Kruskal-Wallis test with Dunn's post-hoc tests comparing each group to fulvestrant, dotted line, *p < 0.05).

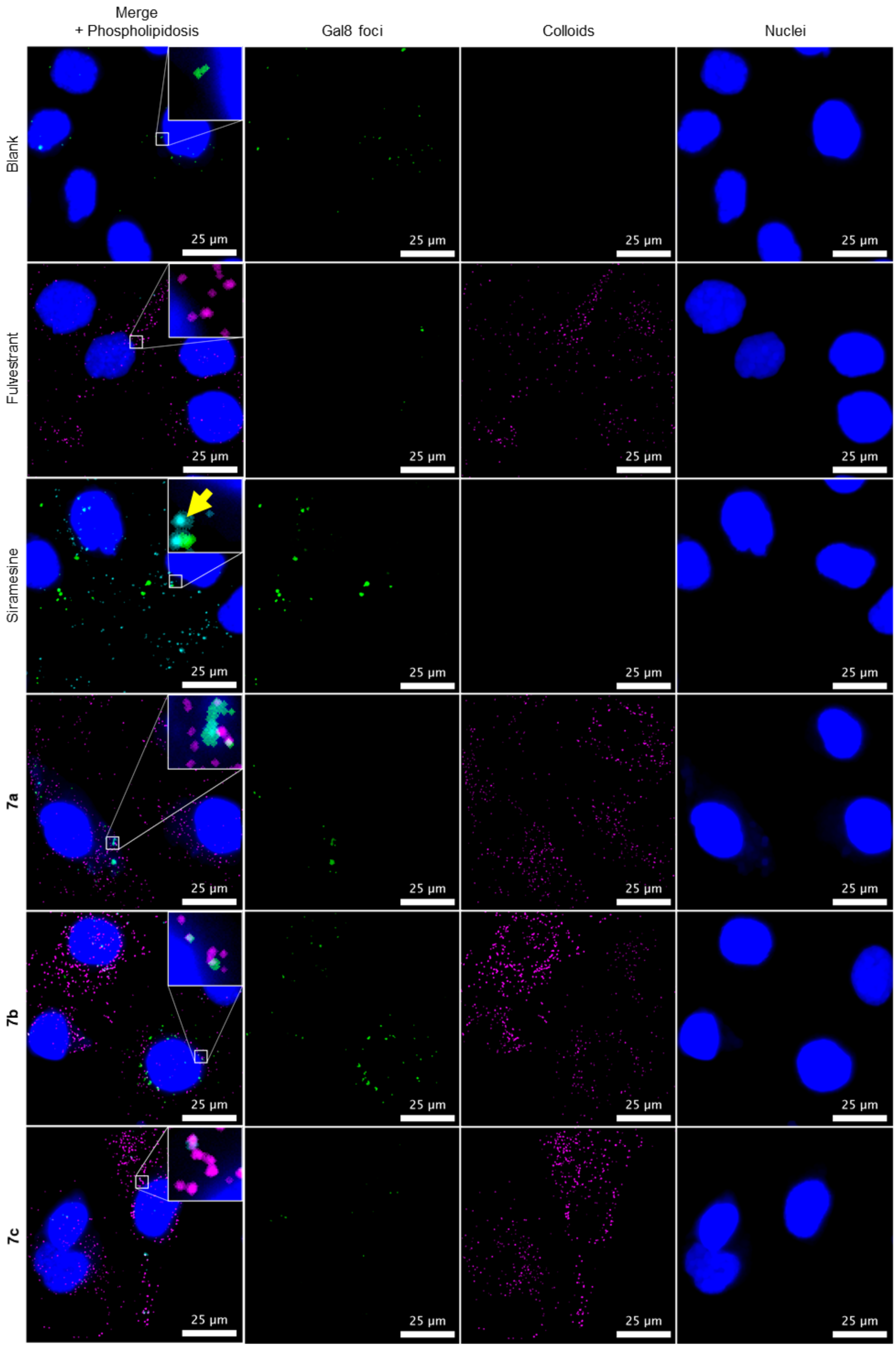
Merge
+ Phospholipidosis
Gal8 foci
Colloids
Nuclei
Blank
Fulvestrant
Siramesine
7a
7b
7c
25 µm

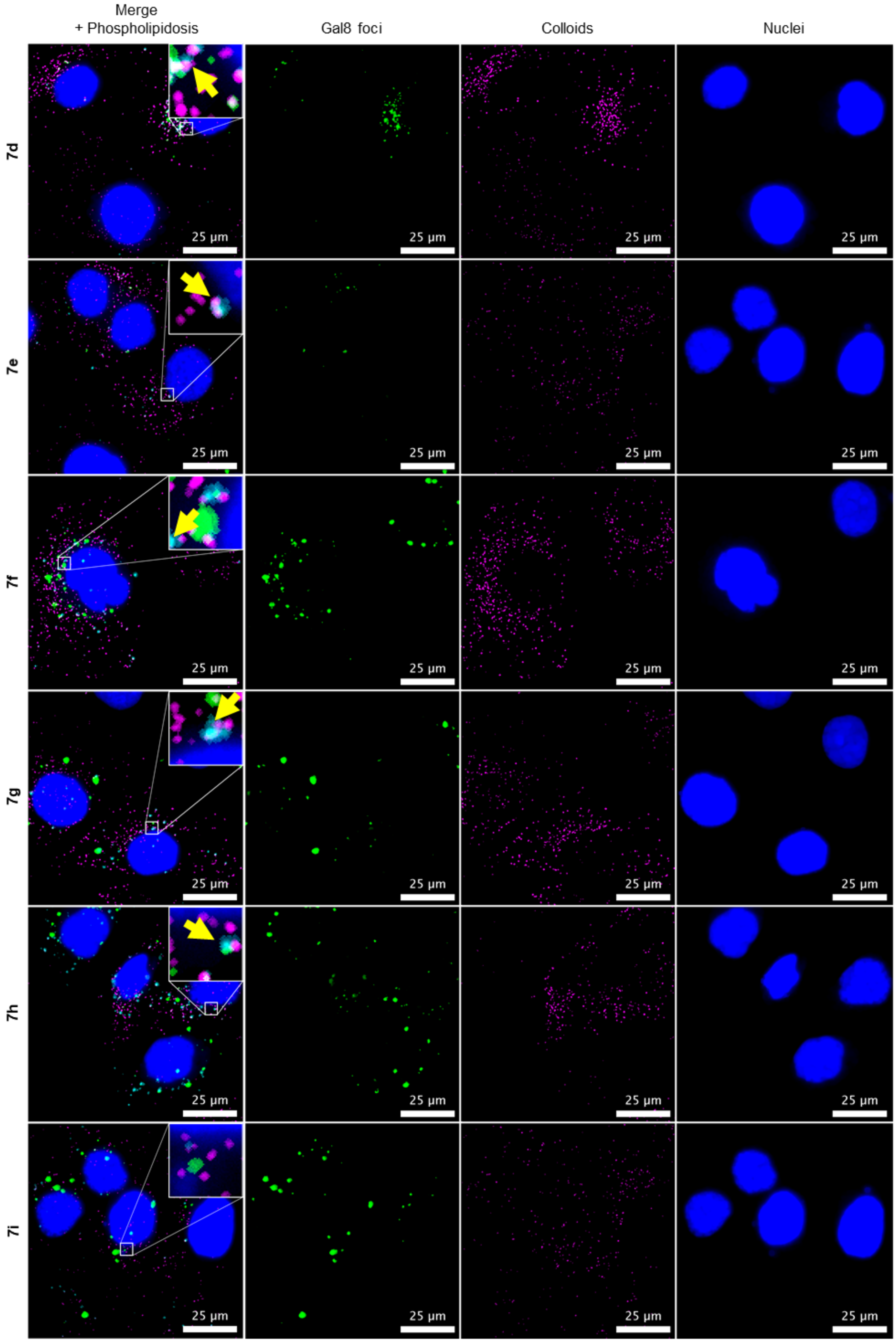
Merge
+ Phospholipidosis
Gal8 foci
Colloids
Nuclei
7d
7e
7f
7g
7h
7i
25 µm

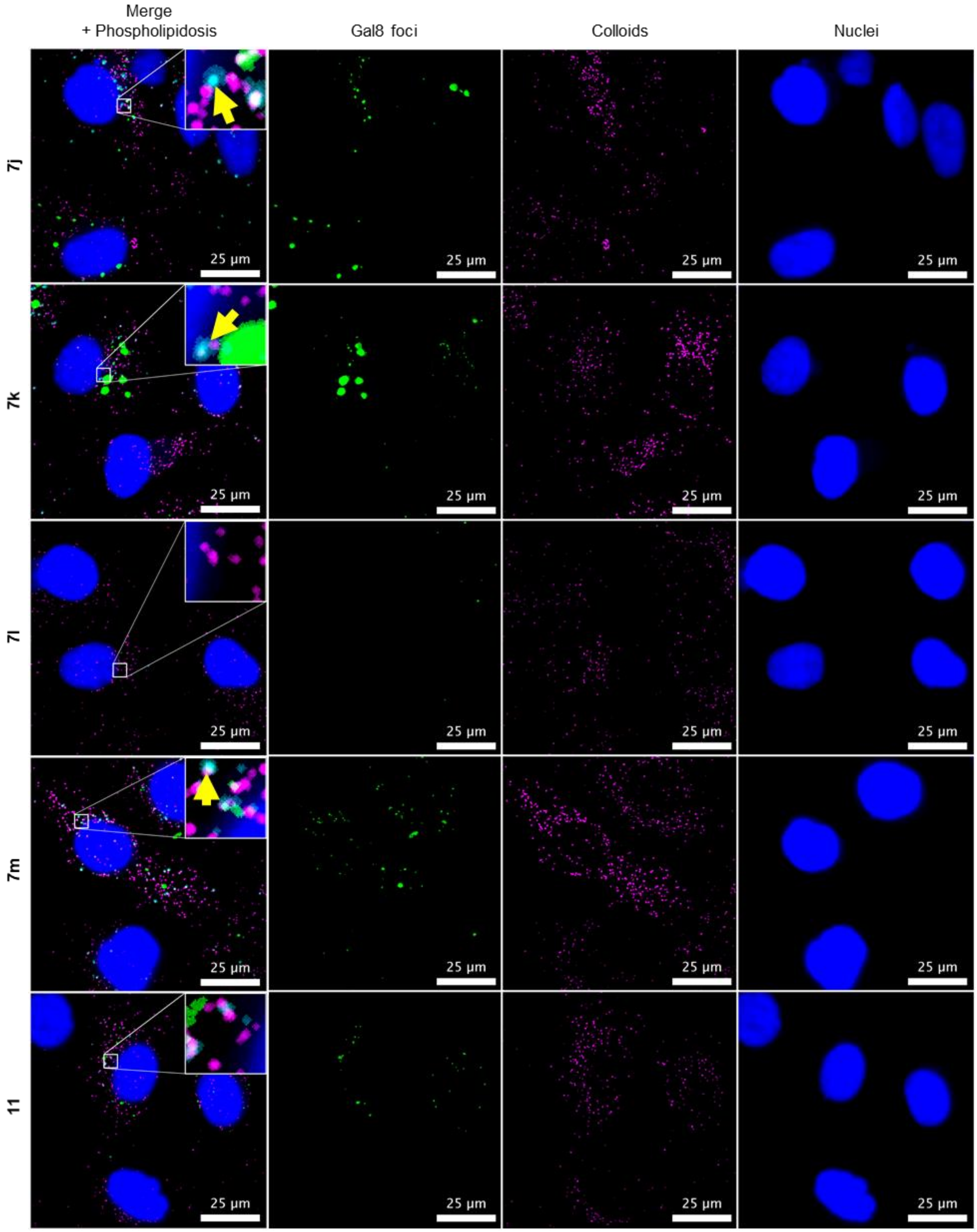

Figure S29. Sample fluorescence images of SKOV3-mChG8 cells after 3 h of treatment with colloidal formulations containing 5 μM of either fulvestrant or fulvestrant analog. The cells were treated for 21 h with 12.5 μM NBD-PE prior to the addition of the colloids. Galectin 8 (mCherry-Gal8) foci are shown in green, colloids (DiD puncta) are shown in magenta, and nuclei (Hoechst) are shown in blue. Phospholipidosis vesicles, visualized by the NBD-PE fluorescence, are shown in cyan in the merge panel.

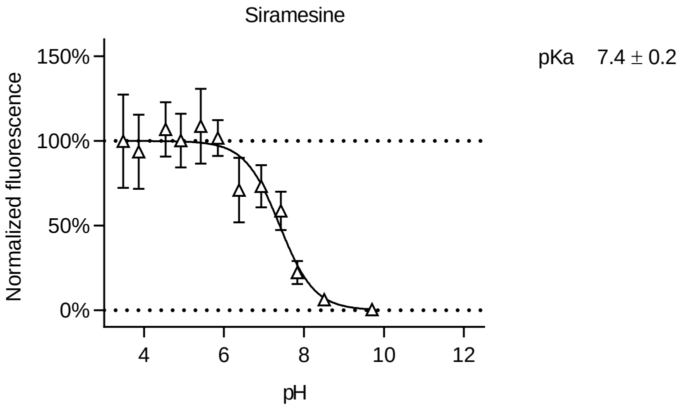

Figure S30. Plot of 6-(*p*-Toluidino)-2-naphthalenesulfonic acid (TNS) fluorescence versus pH and the resulting pK_a of siramesine. The raw fluorescence values were baseline-subtracted and normalized to the signal of the top plateau. The pK_a is calculated as the pH at which the normalized TNS fluorescence is 50% (n = 3 separate mixing events, mean ± SEM).

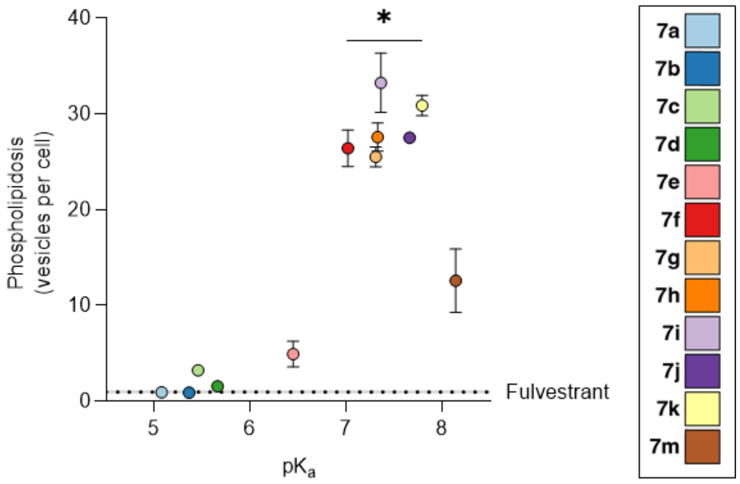

Figure S31. Phospholipidosis quantification of cells treated with non-colloidal fulvestrant analog formulations (2 μM) for 24 h. The cells were co-treated with 12.5 μM NBD-PE for visualization of phospholipidosis (n = 3, mean ± SEM, Brown-Forsythe and Welch ANOVA with Dunnett T3 post-hoc tests comparing each group to fulvestrant, *$p < 0.05$).

Synthetic procedures

3,17β-Bis((tetrahydro-2*H*-pyran-2-yl)oxy)-7-α-[9-(4,4,5,5,5,-pentafluoropentylsulphinyl) nonyl]estra-1,3,5-(10)-triene (2)

3,4-Dihydro-2*H*-pyran (451 µL, 4.94 mmol) and trifluoroacetic acid (25.2 µL, 330 µmol) were added under stirring to a solution of fulvestrant (**1**, 1.0 g, 1.6 mmol) in dichloromethane (10 mL). The reaction was stirred at room temperature for 3 d, then extracted (1x sat. $NaHCO_3$, 1x sat. NaCl), dried over $MgSO_4$, and filtered. The solvent was removed under reduced pressure, yielding a clear viscous oil which was used without further purification. R_f = 0.6-0.7 (1:1 hexanes:ethyl acetate).

3,17β-Bis((tetrahydro-2*H*-pyran-2-yl)oxy)-7-α-[9-(4,4,5,5,5,-pentafluoropentyl sulphonimidoyl)nonyl] estra-1,3,5-(10)-triene (3)

Finely ground ammonium carbamate (530.9 mg, 6.8 mmol), (diacetoxyiodo)benzene (1.656 g, 5.0 mmol), and DIPEA (28.7 µL, 165 µmol) were added to a stirred solution of crude **2** (~1.3 g, ~1.6 mmol) in methanol (20 mL). After a few minutes, gas was evolved, and a yellow color developed. After 4 h, the reaction was diluted in ethyl acetate and extracted (2x PBS, 1x brine), dried over $MgSO_4$, and filtered. The solvent was removed, and the product was purified by column chromatography (silica, 1:1 hexanes:ethyl acetate + 0.1% DIPEA) to give **3** as a clear oil (1.04 g, 1.32 mmol, 80% cumulative yield) R_f = 0.2 (1:1 hexanes:ethyl acetate); ^{1}H NMR (500 MHz, $CDCl_3$) δ 7.21 – 7.16 (m, 1H), 6.86 – 6.82 (m, 1H), 6.75 (d, J = 2.6 Hz, 1H), 5.37 (dt, J = 10.7, 3.3 Hz, 1H), 4.69 – 4.62 (m, 1H), 3.99 – 3.84 (m, 2H), 3.73 (td, J = 8.4, 3.7 Hz, 1H), 3.63 – 3.56 (m, 1H), 3.48 (ddt, J = 8.9, 6.4, 4.3 Hz, 1H), 3.08 (td, J = 7.9, 1.5 Hz, 2H), 3.02 (ddd, J = 10.3, 6.1, 1.3 Hz, 2H), 2.87 (dd, J = 14.2, 7.2 Hz, 1H), 2.98 – 2.65 (m, 2H), 2.77 – 2.68 (m, 1H), 2.36 – 2.05 (m, 7H), 2.03 – 1.93 (m, 2H), 1.93 – 1.29 (m, 30H), 1.21 – 1.07 (m, 2H), 1.06 – 0.96 (m, 1H), 0.80 (d, J = 9.2 Hz, 3H); ^{13}C NMR (126 MHz, $CDCl_3$) δ 136.87, 136.86, 136.81, 133.13, 133.08, 133.00, 132.95, 126.77, 126.74, 117.35, 113.86, 113.80, 99.38, 96.50, 96.28, 86.62, 84.15, 77.26, 77.00, 76.75, 62.74, 62.07, 62.04, 55.49, 53.32, 46.51, 46.44, 43.41, 43.40, 42.90, 42.89, 41.69, 41.62, 38.18, 38.13, 38.08, 38.04, 37.96, 37.39, 33.28, 33.19, 31.08, 31.06, 30.49, 30.45, 29.87, 29.54, 29.37, 29.26, 29.19, 29.08, 28.75, 28.41, 28.20, 27.27, 27.22, 27.15, 25.64, 25.53, 25.27, 22.69, 22.56, 19.92, 19.31, 18.88, 18.85, 11.74, 11.70, -0.03; MS (ESI+) m/z $[M+H]^+$ calculated for $C_{42}H_{65}F_5NO_5S$: 790.4511, found: 790.4503.

N-(2-Bromoacetyl)-(3,17β-bis((tetrahydro-2*H*-pyran-2-yl)oxy)-7-α-[9-(4,4,5,5,5,-pentafluoropentylsulphonimidoyl)nonyl]estra-1,3,5-(10)-triene) (4)

3 (57.9 mg, 73.3 µmol) was dissolved in dichloromethane (2 mL) and *N*,*N*-diisopropylethylamine (63.8 µL, 366 µmol) was added. The solution was cooled on ice, and then a solution of bromoacetyl bromide (12.8 µL, 147 µmol) in dichloromethane (2 mL) added under agitation. The now amber-coloured solution was allowed to warm to room temperature and stirred for 30 minutes. Then, 100 µL of saturated Na_2CO_3 was added and the solvent removed under air. The residue was then dispersed in ethyl acetate and extracted (3x PBS, 1x brine), dried over $MgSO_4$, filtered, and evaporated under a stream of nitrogen. The crude product was purified by column chromatography (silica, 4:1 hexanes:ethyl acetate) to give **4** as a slightly yellow oil (40.5 mg, 44.1 µmol, 60% yield). R_f = 0.4 (4:1 hexanes:ethyl acetate), 0.85 (1:1 hexanes:ethyl acetate); ^{1}H NMR (500 MHz, $CDCl_3$) δ 7.21 – 7.15 (m, 1H), 6.86 – 6.81 (m, 1H), 6.75 (d, J = 2.6 Hz, 1H), 5.37 (dt, J = 10.9, 3.3 Hz, 1H), 4.70 – 4.62 (m, 1H), 3.99 – 3.83 (m, 4H), 3.73 (td, J = 8.4, 3.7 Hz, 1H), 3.64 – 3.56 (m, 2H), 3.53 – 3.45 (m, 2H), 3.33 (dtd, J = 13.7, 8.7, 8.2, 2.8 Hz, 2H), 2.87 (dd, J = 14.2, 7.3 Hz, 1H), 2.72 (dd, J = 16.9, 6.8 Hz, 1H), 2.36 – 2.15 (m, 6H), 1.95 –

1.11 (m, 39H), 1.07 – 0.95 (m, 1H), 0.80 (d, J = 9.2 Hz, 3H); ^{13}C NMR (126 MHz, $CDCl_3$) δ 174.79, 154.92, 136.87, 126.75, 117.35, 113.87, 113.82, 99.39, 96.52, 96.30, 86.62, 84.16, 77.25, 76.99, 76.74, 62.75, 62.06, 51.78, 49.88, 46.51, 46.45, 43.41, 42.90, 42.89, 41.69, 37.96, 37.38, 34.71, 33.29, 33.20, 32.17, 31.09, 31.06, 30.46, 29.86, 29.49, 29.18, 28.94, 28.21, 27.15, 25.64, 25.53, 25.27, 22.55, 21.43, 19.92, 18.86, 11.74, 11.70, -0.02; MS (ESI+) m/z $[M+Na]^+$ calculated for $C_{44}H_{65}BrF_5NNaO_6S$: 932.3528, found: 932.3529.

N-(Acrylyl)-(3,17β-bis((tetrahydro-2*H*-pyran-2-yl)oxy)-7-α-[9-(4,4,5,5,5,-pentafluoropentylsulphonimidoyl)nonyl]estra-1,3,5-(10)-triene) (5)

3 (150 mg, 0.19 mmol) was dissolved in dichloromethane (3 mL) and *N*,*N*-diisopropylethylamine (166 µL, 950 µmol) was added. The solution was cooled on ice, and then a solution of 3-bromopropionoyl chloride (38.3 µL, 380 µmol) in dichloromethane (1 mL) was added under stirring. An acryloyl group is formed rather than the expected 3-bromopropanoyl moiety because the acidity of the α-proton favors the elimination of HBr under the basic reaction conditions. The now faint yellow solution was allowed to warm to room temperature and was stirred for one hour. The solvent was removed under nitrogen, after which the residue was diluted in ethyl acetate and extracted (2x 100 mM pH 8 sodium bicarbonate, 1x sat. NaCl), dried over $MgSO_4$, filtered, and evaporated under a stream of nitrogen. The crude product was purified by column chromatography (silica, 1:1 hexanes:ethyl acetate) to give **5** as an off-white foam after removal of the solvent by evacuation (146 mg, 173 µmol, 91% yield). R_f = 0.9 (ethyl acetate), 0.7 (1:1 hexanes:ethyl acetate); ^{1}H NMR (500 MHz, $CDCl_3$) δ 7.21 – 7.15 (m, 1H), 6.87 – 6.82 (m, 1H), 6.75 (d, J = 2.6 Hz, 1H), 6.34 (dd, J = 17.2, 1.8 Hz, 1H), 6.21 (dd, J = 17.2, 10.1 Hz, 1H), 5.71 (dd, J = 10.1, 1.8 Hz, 1H), 5.38 (dt, J = 11.1, 3.3 Hz, 1H), 4.69 – 4.63 (m, 1H), 3.98 – 3.88 (m, 2H), 3.74 (td, J = 8.4, 3.6 Hz, 1H), 3.68 – 3.56 (m, 2H), 3.50 (tddd, J = 10.7, 9.0, 6.1, 2.3 Hz, 2H), 3.38 – 3.29 (m, 2H), 2.91 – 2.82 (m, 1H), 2.72 (dd, J = 16.8, 6.8 Hz, 1H), 2.35 – 2.14 (m, 6H), 2.12 – 1.09 (m, 40H), 0.80 (d, J = 9.2 Hz, 3H); ^{13}C NMR (126 MHz, $CDCl_3$) δ 173.62, 154.95, 154.88, 154.85, 136.87, 136.83, 136.82, 134.45, 133.14, 133.08, 133.01, 132.96, 128.22, 126.75, 126.72, 117.36, 113.87, 113.81, 99.39, 96.52, 96.51, 96.29, 86.62, 84.17, 77.25, 77.00, 76.75, 62.75, 62.08, 62.04, 61.76, 51.95, 50.01, 46.51, 46.44, 43.42, 43.41, 42.91, 42.90, 41.69, 41.63, 38.18, 38.14, 38.09, 38.04, 37.96, 37.39, 34.70, 33.29, 33.20, 31.09, 31.07, 30.50, 30.46, 29.87, 29.52, 29.37, 29.21, 29.19, 29.02, 28.97, 28.76, 28.26, 28.19, 27.27, 27.22, 27.16, 25.64, 25.53, 25.27, 22.70, 22.56, 21.44, 19.93, 19.31, 18.85, 13.53, 11.74, 11.70, -0.02; MS (ESI+) m/z $[M+Na]^+$ calculated for $C_{45}H_{66}F_5NO_6S$: 866.44, found: 866.44 ($[M+H-2THP]^+$, $[M+H-THP]^+$, $[M+Na-Br+Cl]^+$ also observed).

General procedure for the synthesis of 6

A solution of **4** (14.5 mg, 15.9 µmol) in 1 mL of dichloromethane or **5** (18.2 mg, 21.6 µmol) in 500 µL of acetonitrile was prepared. Then, this solution was added to either 50 equiv. nucleophile hydrochloride mixed with 100 equiv. triethylamine or 10 equiv. of free base nucleophile. The reaction mixture was capped and agitated for 4 h (for reactions involving **4**) or 48 h (for reactions involving **5**), and then the solvent was removed under nitrogen. The residue was diluted in ethyl acetate an extracted (2x PBS, 1x sat. NaCl), dried over $MgSO_4$, filtered, and evaporated under a stream of nitrogen. The crude product was used without further purification.

N-(Morpholin-4-ylacetyl)-(3,17β-bis((tetrahydro-2*H*-pyran-2-yl)oxy)-7-α-[9-(4,4,5,5,5,-pentafluoropentylsulphonimidoyl)nonyl]estra-1,3,5-(10)-triene) (6a)

4 was reacted with morpholine (13.7 µL, 159 µmol) as the nucleophile. Crude **6a** was collected as orange-tinted oil (10.87 mg, 11.9 µmol, 75% yield). R_f = 0.45 (ethyl acetate); MS (ESI+) m/z $[M+H]^+$ calculated for $C_{48}H_{73}F_5N_2O_7S$: 917.5131, found: 917.5136.

***N*-(1*H*-Imidazol-1-ylacetyl)-(3,17β-bis((tetrahydro-2*H*-pyran-2-yl)oxy)-7-α-[9-(4,4,5,5,5,-pentafluoropentylsulphonimidoyl)nonyl]estra-1,3,5-(10)-triene) (**6b**)**

4 (18 mg, 20 µmol) was reacted with imidazole (16 mg, 0.23 mmol) as the nucleophile. Crude **6b** was collected as an off-white solid (18 mg, 20 µmol, 99% yield). R_f = 0 (1:1 hexanes:ethyl acetate). MS (ESI+) m/z $[M+H]^+$ calculated for $C_{47}H_{69}F_5N_3O_6S$: 898.4822, found: 898.4820 ($[M+H\text{-}THP]^+$ and $[M+H\text{-}2THP]^+$ also observed).

***N*-(1*H*-Imidazol-1-ylpropanoyl)-(3,17β-bis((tetrahydro-2*H*-pyran-2-yl)oxy)-7-α-[9-(4,4,5,5,5,-pentafluoropentylsulphonimidoyl)nonyl]estra-1,3,5-(10)-triene) (**6c**)**

5 was reacted with imidazole (45.3 mg, 665 µmol) as the nucleophile. Crude **6c** was collected as an off-white solid (18.13 mg, 19.9 µmol, 92% yield). R_f = 0.4 (ethyl acetate). MS (ESI+) m/z $[M+H]^+$ calculated for $C_{48}H_{70}F_5N_3O_6S$: 912.4978, found: 912.4969.

***N*-(Morpholin-4-ylpropanoyl)-(3,17β-bis((tetrahydro-2*H*-pyran-2-yl)oxy)-7-α-[9-(4,4,5,5,5,-pentafluoropentylsulphonimidoyl)nonyl]estra-1,3,5-(10)-triene) (**6d**)**

5 was reacted with morpholine (18.6 µL, 216 µmol) as the nucleophile. Crude **6d** was collected as an orange-tinted oil (13.15 mg, 14.1 µmol, 65% yield). R_f = 0.0.4 (ethyl acetate). MS (ESI+) m/z $[M+H]^+$ calculated for $C_{49}H_{75}F_5N_2O_7S$: 931.5288, found: 931.5285 ([**5**+Na]$^+$ and [**5**-2THP+H]$^+$ also observed).

***N*-(Piperidinylacetyl)-(3,17β-bis((tetrahydro-2*H*-pyran-2-yl)oxy)-7-α-[9-(4,4,5,5,5,-pentafluoropentylsulphonimidoyl)nonyl]estra-1,3,5-(10)-triene) (**6e**)**

4 was reacted with piperidine (15.7 µL, 159 µmol) as the nucleophile. Crude **6e** was collected as an orange-tinted oil (12.51 mg, 13.7 µmol, 86% yield). R_f = 0.2 (ethyl acetate). MS (ESI+) m/z $[M+H]^+$ calculated for $C_{49}H_{75}F_5N_2O_6S$: 915.5339, found: 915.5339.

***N*-(2-Dimethylaminoacetyl)-(3,17β-bis((tetrahydro-2*H*-pyran-2-yl)oxy)-7-α-[9-(4,4,5,5,5,-pentafluoropentylsulphonimidoyl)nonyl]estra-1,3,5-(10)-triene) (**6f**)**

4 was reacted with dimethylamine hydrochloride (70.64 mg, 867 µmol) mixed with triethylamine (221.6 µL, 1.59 mmol) as the nucleophile. Crude **6f** was collected as an orange-tinted oil (16.13 mg, 18.4 µmol, 116% yield). R_f = 0.05 (ethyl acetate). MS (ESI+) m/z $[M+H]^+$ calculated for $C_{46}H_{71}F_5N_2O_6S$: 875.5032, found: 875.5032.

***N*-(Piperidinylpropanoyl)-(3,17β-bis((tetrahydro-2*H*-pyran-2-yl)oxy)-7-α-[9-(4,4,5,5,5,-pentafluoropentylsulphonimidoyl)nonyl]estra-1,3,5-(10)-triene) (**6g**)**

5 was reacted with piperidine (21.3 µL, 216 µmol) as the nucleophile. Crude **6g** was collected as an off-white solid (17.81 mg, 19.2 µmol, 89% yield). R_f = 0-0.15 (ethyl acetate). MS (ESI+) m/z $[M+H]^+$ calculated for $C_{50}H_{78}F_5N_2O_6S$: 929.5495, found: 929.5488.

***N*-(Pyrrolidinylacetyl)-(3,17β-bis((tetrahydro-2*H*-pyran-2-yl)oxy)-7-α-[9-(4,4,5,5,5,-pentafluoropentyl sulphonimidoyl)nonyl]estra-1,3,5-(10)-triene) (**6h**)**

4 was reacted with pyrrolidine (13.1 µL, 159 µmol) as the nucleophile. Crude **6h** was collected as an orange-tinted oil. R_f = 0.05 (ethyl acetate). MS (ESI+) m/z $[M+H]^+$ calculated for $C_{49}H_{75}F_5N_2O_6S$: 915.5339, found: 915.5339.

***N*-(4-Methylpiperazinylacetyl)-(3,17β-bis((tetrahydro-2*H*-pyran-2-yl)oxy)-7-α-[9-(4,4,5,5,5,-pentafluoropentyl sulphonimidoyl)nonyl]estra-1,3,5-(10)-triene) (**6i**)**

4 was reacted with 1-methylpiperazine (17.6 µL, 159 µmol) as the nucleophile. Crude **6i** was collected as an orange-tinted oil (11.04 mg, 11.9 µmol, 75% yield). R_f = 0.01 (ethyl acetate). MS (ESI+) m/z $[M+H]^+$ calculated for $C_{49}H_{76}F_5N_3O_6S$: 930.5448, found: 930.5455.

***N*-(Pyrrolidinylpropanoyl)-(3,17β-bis((tetrahydro-2*H*-pyran-2-yl)oxy)-7-α-[9-(4,4,5,5,5,-pentafluoropentyl sulphonimidoyl)nonyl]estra-1,3,5-(10)-triene) (**6j**)**

5 was reacted with pyrrolidine (17.7 µL, 216 µmol) as the nucleophile. Crude **6j** was collected as an orange-tinted oil (19.63 mg, 21.4 µmol, 99% yield). R_f = 0 (ethyl acetate).

***N*-(2-Dimethylaminopropanoyl)-(3,17β-bis((tetrahydro-2*H*-pyran-2-yl)oxy)-7-α-[9-(4,4,5,5,5,-pentafluoropentylsulphonimidoyl)nonyl]estra-1,3,5-(10)-triene) (**6k**)**

5 was reacted with dimethylamine hydrochloride (100.9 mg, 1.24 mmol) mixed with triethylamine (301 µL, 2.16 mmol) as the nucleophile. Crude **6k** was collected as an off-white solid (16.05 mg, 18.1 µmol, 84% yield). R_f = 0.1 (ethyl acetate). MS (ESI+) m/z $[M+H]^+$ calculated for $C_{47}H_{73}F_5N_2O_6S$: 889.5182, found: 889.5191.

***N*,*N*,*N*-trimethyl-2-(((9-((7R,8R,9S,13S,14S,17S)-13-methyl-3,17-bis((tetrahydro-2*H*-pyran-2-yl)oxy)-7,8,9,11,12,13,14,15,16,17-decahydro-6*H*-cyclopenta[a]phenanthren-7-yl)nonyl)(oxo)(4,4,5,5,5-pentafluoropentyl)-l6-sulfaneylidene)amino)-2-oxoethan-1-aminium bicarbonate (**6l**)**

4 was reacted with trimethylamine hydrochloride (73.73 mg, 771 µmol) mixed with triethylamine (222 µL, 1.59 mmol) as the nucleophile. Crude **6l** was collected as an off-white solid (6.81 mg, 7.14 µmol, 45% yield). R_f = 0.1 (ethyl acetate).

General procedure for the synthesis of 7a–7l

6 was dissolved in 1 mL of a 2% (v v^{-1}) solution of trifluoroacetic acid in methanol. The mixture was agitated for 24 h, and then the reaction was quenched by addition of 1 mL saturated sodium bicarbonate. The resulting mixture was dispersed in ethyl acetate and extracted (2x PBS, 1x sat. NaCl), dried over $MgSO_4$, filtered, and evaporated under a stream of nitrogen. Crude **7** was purified by column chromatography (basic alumina, dichloromethane with 0–10% (v v^{-1}) methanol) followed by removal of the solvent, dissolution in acetonitrile, filtration through a 0.22 µm PTFE syringe filter, and evaporation under nitrogen. **7a-7l** were then dissolved in DMSO-d6 (optionally including DMF as an internal standard at 1/5 of the concentration of **7**) for characterization and use in experiments.

***N*-(Morpholin-4-ylacetyl)-(7-α-[9-(4,4,5,5,5,-pentafluoropentylsulphonimidoyl)nonyl] estra-1,3,5-(10)-triene-3,17β-diol) (**7a**)**

6a was reacted using the standard procedure, yielding **7a** as an oily residue (5.68 mg, 7.58 µmol, 48% from **4**). R_f = 0.1 (ethyl acetate), 0.8 (acetone), 0.9 (ethanol); ^{1}H NMR (500 MHz, DMSO-d6) δ 8.97 (s, 1H), 7.04 (d, J = 8.5 Hz, 1H), 6.50 (dd, J = 8.4, 2.7 Hz, 1H), 6.41 (d, J = 2.6 Hz, 1H), 4.47 (d, J = 4.9 Hz, 1H), 3.66 – 3.40 (m, 10H), 3.04 (s, 2H), 2.60 (d, J = 16.6 Hz, 1H), 2.48 – 2.43 (m, 4H), 2.30 – 0.74 (m, 32H), 0.66 (s, 3H); ^{13}C NMR (126 MHz, DMSO-d6) δ 177.68, 154.94, 135.98, 129.62, 126.63, 115.75, 112.84, 80.09, 66.15, 63.27, 52.66, 50.57, 49.24, 45.99, 42.95, 41.74, 37.78, 36.76, 35.77, 34.14, 32.74, 31.29, 31.27, 30.76, 29.86, 29.59, 29.35, 29.00, 28.67, 28.39, 28.01, 27.84, 27.68, 27.54, 27.38, 27.06, 26.60, 25.10, 22.27, 22.09, 21.46, 13.95, 13.59, 11.30; MS (ESI+) m/z $[M+H]^+$ calculated for $C_{38}H_{58}F_5N_2O_5S$: 749.3981, found: 749.3978.

***N*-(1*H*-Imidazol-1-ylacetyl)-(7-α-[9-(4,4,5,5,5,-pentafluoropentylsulphonimidoyl)nonyl]estra-1,3,5-(10)-triene-3,17β-diol) (**7b**)**

6b was reacted using the standard procedure, yielding **7b** as an oily residue (1.59 mg, 2.18 µmol, 11% from **4**). R_f = 0.1-0.3 (acetone), 0-0.2 (ethyl acetate), 0.9 (ethanol); ^{1}H NMR (500 MHz, DMSO-d6) δ 8.97 (d, J = 6.6 Hz, 1H), 7.64 (s, 1H), 7.09 (s, 1H), 7.04 (d, J = 8.5 Hz, 1H), 6.89 (s, 1H), 6.50 (dd, J = 8.4, 2.6 Hz, 1H), 6.41 (d, J = 2.6 Hz, 1H), 4.77 (s, 2H), 4.48 (s, 1H),

3.65 – 3.43 (m, 6H), 2.46 – 0.78 (m, 33H), 0.66 (s, 3H); ^{13}C NMR (126 MHz, DMSO-d6) δ 174.99, 154.94, 135.99, 129.62, 126.64, 115.75, 112.85, 103.02, 80.09, 62.93, 51.18, 50.65, 49.31, 45.99, 42.96, 41.74, 37.78, 36.77, 34.15, 33.65, 32.75, 29.87, 29.36, 29.02, 28.72, 28.42, 27.79, 27.56, 27.41, 27.06, 25.10, 24.48, 22.28, 22.09, 21.43, 21.05, 18.39, 13.95, 13.53, 11.30; MS (ESI+) m/z $[M+H]^+$ calculated for $C_{37}H_{53}F_5N_3O_4S$: 730.3671, found: 730.3676.

_N_-(1_H_-Imidazol-1-ylpropanoyl)-(7-α-[9-(4,4,5,5,5,-pentafluoropentylsulphonimidoyl)nonyl]estra-1,3,5-(10)-triene-3,17β-diol) (7c**)**

6c was reacted using the standard procedure, yielding **7c** as an oily residue (7.13 mg, 9.57 µmol, 44% from **5**). R_f = 0.6 (ethanol). ^{1}H NMR (500 MHz, DMSO-d6) δ 9.00 (s, 1H), 7.57 (d, J = 1.2 Hz, 1H), 7.12 (d, J = 1.4 Hz, 1H), 7.10 – 7.01 (m, 1H), 6.82 (d, J = 1.2 Hz, 1H), 6.50 (dd, J = 8.4, 2.5 Hz, 1H), 6.41 (d, J = 2.5 Hz, 1H), 4.49 (s, 1H), 4.18 – 4.10 (m, 2H), 3.65 – 3.41 (m, 6H), 2.86 – 1.00 (m, 34H), 0.96 – 0.83 (m, 1H), 0.66 (d, J = 1.2 Hz, 3H); ^{13}C NMR (126 MHz, DMSO-d6) δ 178.14, 154.95, 137.26, 135.97, 135.11, 129.61, 128.34, 128.12, 126.62, 119.20, 115.82, 115.76, 115.52, 112.84, 80.10, 53.90, 52.03, 51.54, 50.51, 49.18, 46.00, 42.97, 42.96, 42.50, 41.76, 41.70, 40.06, 40.02, 39.85, 39.69, 39.52, 39.35, 39.19, 39.02, 37.79, 36.78, 34.16, 32.76, 29.88, 29.37, 29.03, 28.74, 28.62, 28.40, 28.00, 27.83, 27.66, 27.57, 27.46, 27.08, 25.11, 22.29, 22.12, 21.41, 13.48, 11.31, 11.30, 1.16; MS (ESI+) m/z $[M+H]^+$ calculated for $C_{38}H_{55}F_5N_3O_4S$: 744.3828, found: 744.3826.

_N_-(Morpholin-4-ylpropanoyl)-(7-α-[9-(4,4,5,5,5,-pentafluoropentylsulphonimidoyl)nonyl] estra-1,3,5-(10)-triene-3,17β-diol) (7d**)**

6d was reacted using the standard procedure, yielding **7d** as an oily residue (7.15 mg, 9.36 µmol, 43% from **5**). R_f = 0.05 (ethyl acetate). ^{1}H NMR (500 MHz, DMSO-d6) δ 8.96 (s, 1H), 7.04 (d, J = 8.5 Hz, 1H), 6.50 (dd, J = 8.4, 2.6 Hz, 1H), 6.41 (d, J = 2.6 Hz, 1H), 4.57 – 4.38 (m, 1H), 3.83 – 3.42 (m, 9H), 2.82 – 1.04 (m, 40H), 1.05 – 0.72 (m, 2H), 0.66 (s, 3H); ^{13}C NMR (126 MHz, DMSO-d6) δ 170.31, 154.96, 135.96, 135.87, 129.60, 129.09, 126.62, 115.76, 112.85, 86.83, 80.11, 69.79, 63.54, 59.76, 59.74, 53.70, 52.42, 51.90, 51.34, 50.62, 49.25, 46.03, 46.01, 45.19, 43.20, 43.18, 42.98, 42.96, 41.77, 41.75, 41.27, 40.02, 39.85, 39.70, 39.68, 39.53, 39.52, 39.37, 39.35, 39.19, 39.02, 37.80, 37.43, 36.79, 36.24, 34.16, 34.03, 32.77, 32.70, 29.87, 29.38, 29.06, 29.04, 28.79, 28.78, 28.60, 28.49, 28.47, 28.01, 27.85, 27.83, 27.68, 27.58, 27.50, 27.49, 27.08, 26.70, 26.58, 25.11, 22.29, 22.04, 21.49, 21.07, 20.77, 20.75, 14.09, 14.08, 13.61, 11.73, 11.71, 11.31, 11.30; MS (ESI+) m/z $[M+H]^+$ calculated for $C_{38}H_{57}F_5N_2O_5S$: 763.4138, found: 763.4131.

_N_-(Piperidinylacetyl)-(7-α-[9-(4,4,5,5,5,-pentafluoropentylsulphonimidoyl)nonyl] estra-1,3,5-(10)-triene-3,17β-diol) (7e**)**

6e was reacted using the standard procedure, yielding **7e** as an oily residue (9.36 mg, 12.5 µmol, 79% from **4**). R_f = 0.1 (ethyl acetate). ^{1}H NMR (500 MHz, DMSO-d6) δ 9.05 (s, 1H), 7.04 (d, J = 8.5 Hz, 1H), 6.50 (dd, J = 8.4, 2.6 Hz, 1H), 6.41 (d, J = 2.6 Hz, 1H), 4.55 – 4.40 (m, 1H), 3.96 (s, 1H), 3.74 – 0.74 (m, 50H), 0.66 (s, 3H); ^{13}C NMR (126 MHz, DMSO-d6) δ 158.21, 157.96, 157.72, 155.00, 135.96, 129.58, 126.62, 120.84, 118.45, 116.06, 115.76, 113.67, 112.86, 80.11, 62.55, 52.84, 52.00, 50.84, 49.50, 46.02, 45.63, 42.97, 41.76, 40.02, 39.85, 39.69, 39.52, 39.35, 39.19, 39.02, 37.80, 36.78, 34.16, 32.77, 29.87, 29.39, 29.05, 28.74, 28.44, 27.95, 27.78, 27.61, 27.58, 27.41, 27.07, 25.12, 22.28, 22.10, 21.42, 21.28, 13.53, 11.30, 8.54, 7.38, 7.16; MS (ESI+) m/z $[M+H]^+$ calculated for $C_{39}H_{59}F_5N_2O_4S$: 747.4173, found: 747.4181.

***N*-(2-Dimethylaminoacetyl)-(7-α-[9-(4,4,5,5,5,-pentafluoropentylsulphonimidoyl)nonyl] estra-1,3,5-(10)-triene-3,17β-diol)** (7f)

6f was reacted using the standard procedure, yielding **7f** as an oily residue (6.12 mg, 8.68 µmol, 55% from **4**). R_f = 0 (ethyl acetate), 0.2 (2% (v v^{-1}) methanol in dichloromethane, basic alumina). ^1H NMR (500 MHz, DMSO-d6) δ 8.97 (s, 1H), 7.04 (d, J = 8.4 Hz, 1H), 6.50 (dd, J = 8.4, 2.6 Hz, 1H), 6.41 (d, J = 2.5 Hz, 1H), 4.48 (d, J = 4.7 Hz, 1H), 3.63 – 3.43 (m, 5H), 3.01 (d, J = 1.0 Hz, 2H), 2.79 – 0.72 (m, 40H), 0.66 (d, J = 1.0 Hz, 3H); ^{13}C NMR (126 MHz, DMSO-d6) δ 154.96, 135.97, 129.61, 126.63, 115.75, 112.85, 80.10, 61.72, 50.72, 49.40, 46.01, 43.81, 42.96, 41.74, 37.79, 36.78, 34.15, 32.76, 29.86, 29.37, 29.02, 28.72, 28.42, 27.81, 27.56, 27.41, 27.06, 25.81, 25.11, 22.28, 21.42, 13.55, 11.30; MS (ESI+) m/z $[M+H]^+$ calculated for $C_{36}H_{56}F_5N_2O_4S$: 707.3875, found: 707.3876.

***N*-(Piperidinylpropanoyl)-(7-α-[9-(4,4,5,5,5,-pentafluoropentylsulphonimidoyl)nonyl] estra-1,3,5-(10)-triene-3,17β-diol) (**7g**)**

6g was reacted using the standard procedure, yielding **7g** as an oily residue (9.65 mg, 12.7 µmol, 58% from **5**). R_f = 0.5 (ethanol). ^1H NMR (500 MHz, DMSO-d6) δ 9.07 – 8.92 (m, 1H), 7.10 – 7.01 (m, 1H), 6.50 (dt, J = 8.5, 2.1 Hz, 1H), 6.42 (q, J = 3.4, 2.0 Hz, 1H), 4.48 (dd, J = 4.9, 1.8 Hz, 1H), 4.03 (qd, J = 7.2, 1.6 Hz, 2H), 3.74 – 3.45 (m, 5H), 3.19 (t, J = 7.6 Hz, 2H), 3.11 – 0.76 (m, 44H), 0.66 (d, J = 1.7 Hz, 3H); ^{13}C NMR (126 MHz, DMSO-d6) δ 177.39, 170.31, 154.97, 135.96, 135.87, 129.59, 126.62, 118.47, 117.64, 116.08, 115.75, 112.85, 86.83, 80.11, 59.74, 53.90, 52.17, 52.02, 50.62, 49.25, 46.01, 45.19, 43.18, 42.96, 41.75, 40.02, 39.85, 39.69, 39.52, 39.35, 39.19, 39.02, 37.80, 37.42, 36.78, 34.16, 33.14, 32.77, 32.69, 29.87, 29.38, 29.05, 28.77, 28.62, 28.47, 28.12, 28.00, 27.83, 27.76, 27.66, 27.58, 27.52, 27.48, 27.07, 25.11, 22.64, 22.28, 22.11, 21.49, 21.29, 21.05, 20.75, 14.08, 13.60, 11.72, 11.30; MS (ESI+) m/z $[M+H]^+$ calculated for $C_{40}H_{61}F_5N_2O_4S$: 761.4358, found: 763.4362.

***N*-(Pyrrolidinylacetyl)-(7-α-[9-(4,4,5,5,5,-pentafluoropentylsulphonimidoyl)nonyl] estra-1,3,5-(10)-triene-3,17β-diol)** (7h)

6 was reacted using the standard procedure, yielding **7h** as an oily residue (10.2 mg, 13.7 µmol, 63% from **4**). R_f = 0 (ethyl acetate), 0.2 (ethanol). ^1H NMR (500 MHz, DMSO-d6) δ 8.96 (s, 1H), 7.02 (d, J = 8.5 Hz, 1H), 6.48 (dd, J = 8.4, 2.6 Hz, 1H), 6.39 (d, J = 2.6 Hz, 1H), 4.53 – 4.40 (m, 1H), 3.63 – 3.42 (m, 5H), 3.14 (s, 2H), 2.78 – 0.72 (m, 42H), 0.64 (s, 3H); ^{13}C NMR (126 MHz, DMSO-d6) δ 178.66, 155.40, 136.40, 132.15, 130.04, 127.06, 116.20, 113.28, 80.54, 67.83, 61.02, 53.50, 50.99, 49.68, 46.44, 43.40, 42.20, 40.46, 40.30, 40.13, 39.96, 39.80, 39.63, 39.46, 38.53, 38.23, 37.22, 36.22, 34.60, 33.20, 31.21, 30.31, 30.24, 29.82, 29.45, 29.10, 28.83, 28.81, 28.47, 28.30, 28.13, 28.00, 27.82, 27.51, 25.55, 23.82, 23.69, 22.85, 22.72, 21.90, 14.33, 14.02, 11.74, 11.24; MS (ESI+) m/z $[M+H]^+$ calculated for $C_{38}H_{57}F_5N_2O_4S$: 733.4032, found: 733.4036.

***N*-(4-Methylpiperazinylacetyl)-(7-α-[9-(4,4,5,5,5,-pentafluoropentylsulphonimidoyl)nonyl] estra-1,3,5-(10)-triene-3,17β-diol)** (7i)

6i was reacted using the standard procedure, yielding **7i** as an oily residue (4.87 mg, 6.39 µmol, 40% from **4**). R_f = 0 (ethyl acetate), 0-0.5 (2% (v v^{-1}) methanol in dichloromethane, basic alumina). ^1H NMR (500 MHz, DMSO-d6) δ 8.99 (s, 1H), 7.10 – 7.01 (m, 1H), 6.50 (dd, J = 8.4, 2.4 Hz, 1H), 6.41 (d, J = 2.3 Hz, 1H), 4.48 (s, 1H), 3.85 – 0.76 (m, 52H), 0.66 (d, J = 1.9 Hz, 3H); ^{13}C NMR (126 MHz, DMSO-d6) δ 177.72, 154.96, 135.96, 129.60, 126.61, 115.85, 115.75, 115.55, 112.84, 80.10, 62.75, 57.89, 54.25, 51.48, 50.60, 49.26, 46.01, 45.70, 45.10, 42.96, 41.75, 40.02, 39.85, 39.69, 39.52, 39.35, 39.19, 39.02, 37.79, 36.78, 34.16, 32.76, 29.87, 29.38,

29.03, 28.70, 28.42, 28.03, 27.87, 27.70, 27.57, 27.41, 27.07, 25.11, 22.28, 21.47, 13.61, 11.30, 7.63, 1.15.; MS (ESI+) m/z $[M+H]^+$ calculated for $C_{39}H_{60}F_5N_3O_4S$: 762.4297, found: 762.4294.

_N_-(Pyrrolidinylpropanoyl)-(7-α-[9-(4,4,5,5,5,-pentafluoropentylsulphonimidoyl)nonyl] estra-1,3,5-(10)-triene-3,17β-diol) (7j**)**

6j was reacted using the standard procedure, yielding **7j** as an oily residue (12.2 mg, 16.3 µmol, 76% from **5**). R_f = 0.2 (ethanol). ^{1}H NMR (500 MHz, DMSO-d6) δ 9.00 (s, 1H), 7.04 (d, J = 8.5 Hz, 1H), 6.50 (dd, J = 8.4, 2.6 Hz, 1H), 6.41 (d, J = 2.5 Hz, 1H), 4.48 (d, J = 4.7 Hz, 1H), 4.03 (qd, J = 7.1, 1.1 Hz, 1H), 3.78 – 0.72 (m, 50H), 0.71 – 0.61 (m, 3H); ^{13}C NMR (126 MHz, DMSO-d6) δ 177.39, 170.32, 154.97, 135.97, 129.60, 126.62, 118.47, 116.08, 115.85, 115.76, 112.86, 80.11, 69.79, 59.75, 53.90, 53.20, 52.03, 50.62, 50.14, 49.26, 46.01, 43.19, 42.97, 41.76, 40.02, 39.85, 39.69, 39.52, 39.35, 39.19, 39.02, 37.80, 36.78, 34.16, 32.77, 29.87, 29.38, 29.04, 28.79, 28.77, 28.62, 28.46, 28.12, 28.00, 27.83, 27.76, 27.67, 27.58, 27.55, 27.48, 27.07, 25.11, 22.61, 22.28, 22.12, 21.48, 21.06, 20.76, 14.08, 13.61, 11.72, 11.30; MS (ESI+) m/z $[M+H]^+$ calculated for $C_{39}H_{59}F_5N_2O_4S$: 747.4188, found: 747.4182.

_N_-(Dimethylaminopropanoyl)-(7-α-[9-(4,4,5,5,5,-pentafluoropentylsulphonimidoyl)nonyl] estra-1,3,5-(10)-triene-3,17β-diol) (7k**)**

6k was reacted using the standard procedure, yielding **7k** as an oily residue (9.99 mg, 13.8 µmol, 64% from **5**). R_f = 0.05 (ethyl acetate). ^{1}H NMR (500 MHz, DMSO-d6) δ 8.98 (s, 1H), 7.04 (d, J = 8.5 Hz, 1H), 6.50 (dd, J = 8.5, 2.6 Hz, 1H), 6.41 (d, J = 2.5 Hz, 1H), 4.48 (s, 1H), 3.82 – 0.75 (m, 59H), 0.66 (s, 3H); ^{13}C NMR (126 MHz, DMSO-d6) δ 179.56, 154.95, 135.96, 129.60, 126.61, 117.65, 115.84, 115.75, 115.54, 112.84, 80.10, 55.12, 53.91, 52.03, 50.46, 49.12, 46.00, 44.56, 43.18, 42.96, 42.48, 41.75, 40.02, 39.85, 39.69, 39.52, 39.35, 39.19, 39.02, 37.79, 36.88, 36.78, 34.15, 32.75, 29.87, 29.37, 29.02, 28.80, 28.70, 28.41, 28.05, 27.89, 27.72, 27.56, 27.44, 27.07, 25.11, 22.28, 22.11, 21.37, 19.07, 13.51, 11.30, 1.15; MS (ESI+) m/z $[M+H]^+$ calculated for $C_{37}H_{57}F_5N_2O_4S$: 721.4032, found: 721.4032.

2-_N_-(((9-((7R,8R,9S,13S,14S,17S)-3,17-dihydroxy-13-methyl-7,8,9,11,12,13,14,15,16,17-decahydro-6_H_-cyclopenta[a]Phenanthren-7-yl)nonyl)(oxo)(4,4,5,5,5-pentafluoropentyl)-l6-sulfaneylidene)amino)-_N,N,N_-trimethyl-2-oxoethan-1-aminium trifluoroacetate (7l**)**

6l was reacted using the standard procedure, yielding **7l** as an oily residue (3.14 mg, 4.17 µmol, 26% from **4**). R_f = 0 (ethyl acetate), 0.4 (10% (v v^{-1}) methanol in dichloromethane, alumina). ^{1}H NMR (500 MHz, DMSO-d6) δ 9.10 – 8.95 (m, 1H), 7.05 (dd, J = 8.5, 3.1 Hz, 1H), 6.51 (dt, J = 8.4, 3.0 Hz, 1H), 6.42 (q, J = 3.2 Hz, 1H), 4.59 (td, J = 5.5, 2.9 Hz, 1H), 4.49 (dd, J = 4.9, 2.9 Hz, 1H), 4.25 (d, J = 3.2 Hz, 2H), 3.76 – 0.74 (m, 47H), 0.67 (d, J = 3.2 Hz, 3H); ^{13}C NMR (126 MHz, DMSO-d6) δ 171.60, 154.98, 135.97, 129.59, 126.62, 115.76, 112.86, 80.11, 74.58, 72.34, 69.82, 69.79, 69.77, 65.65, 60.21, 53.40, 52.77, 50.74, 49.45, 46.02, 43.69, 43.19, 42.97, 41.75, 40.02, 39.86, 39.69, 39.52, 39.35, 39.19, 39.02, 37.81, 36.78, 34.16, 32.77, 29.87, 29.38, 29.04, 28.73, 28.43, 27.93, 27.77, 27.59, 27.38, 27.08, 25.12, 22.29, 22.20, 21.43, 17.28, 13.57, 11.30, 7.39, 1.15; MS (ESI+) m/z [M] calculated for $C_{37}H_{58}F_5N_2O_4S^+$: 721.4030, found: 721.4025.

_N_-(2-Aminoacetyl)-(7-α-[9-(4,4,5,5,5,-pentafluoropentylsulphonimidoyl)nonyl]estra-1,3,5-(10)-triene-3,17β-diol) (7m**)**

10 (43.2 mg, 42.9 µmol) was dissolved in 1 mL tetrahydrofuran. Tetrabutylammonium fluoride (214.4 µmol, 1 M in tetrahydrofuran) and acetic acid (214 µmol) were added, and the reaction was stirred at 60 °C for 4 h. The solvent was removed under nitrogen, then the residue

was resuspended in ethyl acetate and extracted (1x 0.1M pH 8 $NaHCO_3$, 1x sat. NaCl). The solvent was removed and the crude material was purified by column chromatography (3:1 hexanes:ethyl acetate mobile phase) to yield 32.4 mg of an intermediate material with the phenolic TBS ether removed (data not shown). This intermediate was dissolved in 500 µL of dichloromethane, and 500 µL of ice-cold 5% (v v^{-1}) water in trifluoroacetic acid was added under stirring on ice. The reaction was allowed to warm to room temperature, and 1.5 mL of sat. Na_2CO_3 was added after 3 h to quench the reaction. This mixture was then diluted in ethyl acetate and extracted (1x water), dried over $MgSO_4$, filtered (0.45 µm PFTE), and evaporated under a stream of nitrogen. The crude product was purified by HPLC (1:1 water:acetonitrile + 0.1% trifluoroacetic acid to acetonitrile + 0.1% trifluoroacetic acid gradient mobile phase, C18-modified silica stationary phase). After removing the solvent under air, the product was redissolved in methyl-*tert*-butyl ether and deprotonated by extracting with sat. Na_2CO_3. The organic phase was isolated, dried over $MgSO_4$, filtered, and evaporated under a stream of nitrogen, yielding **7m** as an oily residue (1.74 mg, 2.56 µmol, 6.0% yield). ^{1}H NMR (500 MHz, DMSO-d6) δ 7.04 (d, J = 8.5 Hz, 1H), 6.50 (dd, J = 8.5, 2.6 Hz, 1H), 6.41 (d, J = 2.6 Hz, 1H), 4.48 (s, 1H), 3.66 – 3.42 (m, 5H), 3.23 (s, 2H), 2.75 (dd, J = 16.6, 5.2 Hz, 1H), 2.63 – 2.55 (m, 1H), 2.47 – 2.34 (m, 2H), 2.25 (dd, J = 12.1, 4.2 Hz, 1H), 2.21 – 2.11 (m, 1H), 2.05 – 1.58 (m, 7H), 1.58 – 1.13 (m, 22H), 0.97 – 0.79 (m, 1H), 0.66 (s, 3H); MS (ESI+) m/z $[M+H]^+$ calculated for $C_{34}H_{52}F_5N_2O_4S$: 679.3562, found: 679.3559.

3,17β-bis((*tert*-Butyldimethylsilyl)oxy)-7-α-[9-(4,4,5,5,5,-pentafluoropentylsulphinyl) nonyl]estra-1,3,5-(10)-triene (8)

8 was prepared as previously described.[2] Imidazole (537.5 mg, 7.90 mmol) and fulvestrant (488.9 mg, 805.7 µmol) were dissolved in 2.5 mL of dry DMF. *tert*-Butyl-dimethylsilyl chloride (445.6 mg, 2.96 mmol) was added, and the reaction was stirred for 24 h at room temperature. The mixture was diluted in 20 mL of methyl-*tert*-butyl ether and extracted (1x 0.1 M pH 8 $NaHCO_3$). The solvent was removed under reduced pressure, yielding crude **8** as a clear oil which was used without further purification. R_f = 0.8 (3:1 hexanes:ethyl acetate).

3,17β-bis((*tert*-Butyldimethylsilyl)oxy)-7-α-[9-(4,4,5,5,5,-pentafluoropentylsulphonimidoyl)nonyl]estra-1,3,5-(10)-triene (9)

9 was prepared as previously described.[2] **8** (~809 mg, ~806 µmol) was dissolved in 5 mL of methanol. Finely ground ammonium carbamate (236.0 mg, 3.02 mmol) and (diacetoxyiodo)benzene (712.2 mg, 2.21 mmol) were weighed out and added to the reaction mixture under stirring. After a few minutes, gas (presumably CO_2) was evolved and a yellow color developed. The reaction was stirred at room temperature for 4 h. Then, the reaction was diluted in 15 mL of ethyl acetate and extracted (2x water, 1x sat. NaCl), dried over $MgSO_4$, filtered, and evaporated under reduced pressure. The crude product was purified by column chromatography (1:1 hexanes:ethyl acetate mobile phase, silica stationary phase), yielding **9** (547.4 mg, 643.8 µmol, 80% from fulvestrant) as a yellow-tinted oil after evaporation of the solvent. R_f = 0.4 (1:1 hexanes:ethyl acetate). ^{1}H NMR (500 MHz, $CDCl_3$) δ 7.11 (dd, J = 8.6, 0.9 Hz, 1H), 6.64 – 6.57 (m, 1H), 6.53 (d, J = 2.6 Hz, 1H), 3.69 – 3.61 (m, 1H), 3.08 (td, J = 7.9, 1.5 Hz, 2H), 3.02 (ddd, J = 10.3, 6.0, 1.2 Hz, 2H), 2.84 (dd, J = 16.7, 5.4 Hz, 1H), 2.68 (dd, J = 17.0, 1.5 Hz, 1H), 2.34 – 2.12 (m, 6H), 2.01 – 1.08 (m, 27H), 0.97 (s, 10H), 0.89 (s, 10H), 0.87 – 0.78 (m, 1H), 0.74 (s, 3H), 0.19 (s, 6H), 0.07 (s, 6H); ^{13}C NMR (126 MHz, $CDCl_3$) δ 153.18, 136.82, 132.57, 126.66, 120.80, 117.13, 81.85, 77.25, 76.99, 76.74, 56.84, 55.46, 53.36, 46.11, 43.71, 41.95, 38.26, 37.38, 34.60, 33.29, 30.94, 29.95, 29.69, 29.59, 29.54, 29.37, 29.28, 29.19, 29.07, 28.42, 28.22, 27.29, 25.86, 25.68, 25.59, 22.79, 22.32, 18.13, 18.11, 14.28, 11.37, -0.02, -

4.38, -4.40, -4.43, -4.47, -4.79; MS (ESI+) m/z $[M+H]^+$ calculated for $C_{44}H_{76}F_5NO_3SSi_2$: 850.5073, found: 850.5068.

_N_-((_N_-_tert_-Butoxycarbonyl)-2-aminoacetyl)-(3,17β-bis((_tert_-butyldimethylsilyl)oxy)-7-α-[9-(4,4,5,5,5,-pentafluoropentylsulphonimidoyl)nonyl]estra-1,3,5-(10)-triene) (10)

N-butoxycarbonyl-glycine (15.9 mg, 90.6 µmol), HCTU·PF_6 (30.0 mg, 72.5 µmol), and DIPEA (30.1 µL, 172 µmol) were dissolved in 500 µL of dry DMF and stirred for 30 min at room temperature. This solution was then added to **9** and stirred for 18 h. Then, the reaction was diluted in methyl-*tert*-butyl ether and extracted (3x water, 1x sat. NaCl), dried over $MgSO_4$, filtered, and evaporated under a stream of nitrogen. The crude product was then purified with column chromatography (3:1 hexanes:ethyl acetate mobile phase, silica stationary phase) and evaporated to yield **10** (43.2 mg, 42.9 µmol, 86% yield). R_f = 0.4 (3:1 hexanes:ethyl acetate). ^{1}H NMR (500 MHz, $CDCl_3$) δ 7.15 – 7.08 (m, 1H), 6.60 (dd, J = 8.4, 2.6 Hz, 1H), 6.57 – 6.51 (m, 1H), 5.08 (s, 2H), 3.89 (s, 4H), 3.65 (t, J = 8.3 Hz, 1H), 3.56 (dt, J = 11.3, 7.7 Hz, 3H), 3.46 (s, 2H), 3.32 (dt, J = 14.7, 7.5 Hz, 3H), 2.84 (dd, J = 16.7, 5.4 Hz, 1H), 2.67 (d, J = 16.7 Hz, 1H), 2.42 (dd, J = 13.4, 2.3 Hz, 1H), 2.26 (s, 8H), 2.18 (h, J = 7.2, 6.6 Hz, 4H), 1.91 (s, 1H), 1.82 (s, 6H), 1.57 (s, 15H), 1.44 (d, J = 1.3 Hz, 23H), 1.21 – 1.09 (m, 3H), 1.09 – 1.00 (m, 2H), 0.97 (d, J = 2.0 Hz, 16H), 0.96 – 0.83 (m, 25H), 0.87 – 0.81 (m, 1H), 0.80 (s, 2H), 0.74 (s, 3H), 0.36 (s, 3H), 0.26 – 0.15 (m, 2H), 0.18 (s, 5H), 0.10 (s, 1H), 0.05 – -0.01 (m, 12H); MS (ESI+) m/z $[M+H]^+$ calculated for $C_{51}H_{87}F_5N_2O_6SSi_2$: 1007.5830, found: 1007.5825.

7-α-[9-(4,4,5,5,5,-Pentafluoropentylsulphonimidoyl)nonyl]estra-1,3,5-(10)-triene-3,17β-diol (11)

9 (128.5 mg, 151.1 µmol) was dissolved in 3 mL of methanol. 30 µL of 6 M aqueous HCl was added under stirring, and the reaction was stirred at room temperature for 18 h. The reaction was then quenched with 200 µL sat. Na_2CO_3 and the solvent was partially removed under nitrogen. The residue was then resuspended in ethyl acetate and extracted (1x water, 1x sat. NaCl), dried over $MgSO_4$, filtered, and evaporated. The crude product was purified using column chromatography using 1:1 hexanes:ethyl acetate as the mobile phase and silica as the stationary phase. The product was then redissolved in acetonitrile and filtered (0.22 µm PTFE), evaporated, redissolved in dichloromethane, and evaporated again, yielding **11** as a yellow-tinted solid (61.6 mg, 99.0 µmol, 66% yield). R_f = 0.35 (ethyl acetate); ^{1}H NMR (500 MHz, DMSO-d6) δ 8.94 (s, 1H), 7.02 (d, J = 8.5 Hz, 1H), 6.48 (dd, J = 8.4, 2.6 Hz, 1H), 6.40 (d, J = 2.6 Hz, 1H), 5.73 (s, 1H), 4.46 (d, J = 4.7 Hz, 1H), 3.84 – 3.58 (m, 1H), 3.52 (td, J = 8.5, 3.4 Hz, 1H), 3.40 – 3.20 (m, 1H), 3.14 – 3.02 (m, 2H), 3.02 – 2.88 (m, 2H), 2.73 (dd, J = 16.7, 5.3 Hz, 1H), 2.63 – 2.53 (m, 1H), 2.52 (s, 1H), 2.46 – 2.30 (m, 2H), 2.29 – 2.09 (m, 2H), 1.90 (dddd, J = 22.8, 14.1, 9.1, 5.1 Hz, 4H), 1.77 (dt, J = 13.2, 3.6 Hz, 1H), 1.71 – 1.55 (m, 3H), 1.55 – 1.04 (m, 24H), 0.94 – 0.75 (m, 2H), 0.64 (s, 3H); ^{13}C NMR (126 MHz, DMSO-d6) δ 155.40, 136.40, 130.05, 127.04, 120.42, 118.44, 118.15, 117.85, 116.44, 116.20, 116.15, 115.85, 113.28, 90.37, 80.55, 55.33, 54.35, 52.47, 46.45, 43.40, 42.20, 40.46, 40.30, 40.13, 39.96, 39.80, 39.63, 39.46, 38.23, 37.22, 36.66, 34.60, 33.91, 33.21, 30.32, 29.82, 29.50, 29.25, 29.07, 28.74, 28.70, 28.57, 28.41, 28.22, 28.00, 27.52, 25.98, 25.54, 24.86, 24.73, 24.53, 24.12, 22.72, 22.56, 21.47, 21.13, 18.79, 18.30, 14.65, 14.62, 14.58, 11.73, 8.29, -4.08; MS (ESI+) m/z $[M+H]^+$ calculated for $C_{32}H_{49}F_5NO_3S$: 622.3348, found: 622.3344.

Characterization data

Nuclear magnetic resonance (^{1}H and ^{13}C NMR) spectra were collected using a 500 MHz Agilent DD2 spectrometer or a 400 MHz Bruker Avance III spectrometer. Mass spectra were obtained using an Agilent 6538 UHD spectrometer operating in positive electrospray ionization (ESI+) mode.

1

^{1}H, $CDCl_3$, 500 MHz

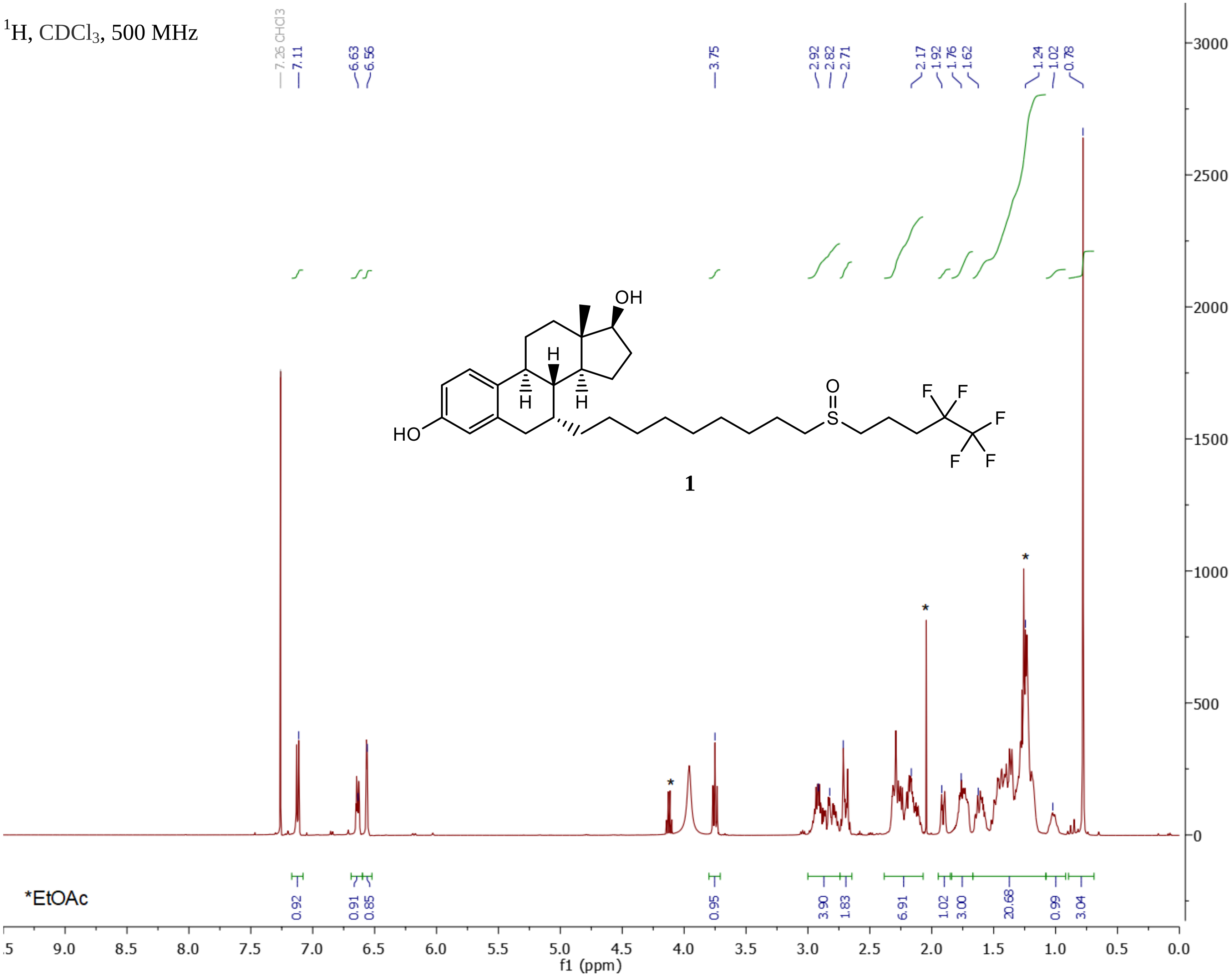

^{13}C, $CDCl_3$, 126 MHz

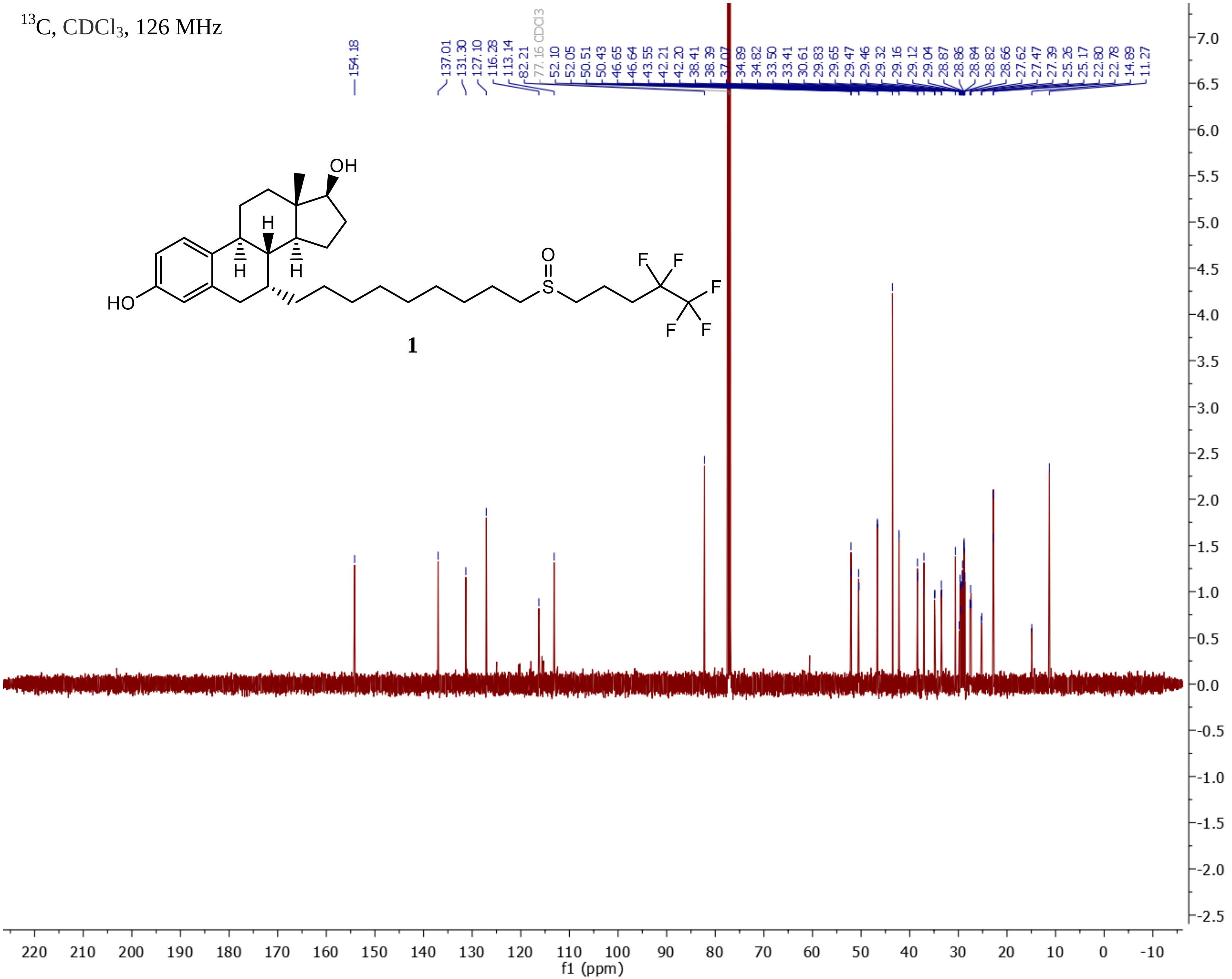

3

^{1}H, $CDCl_3$, 500 MHz

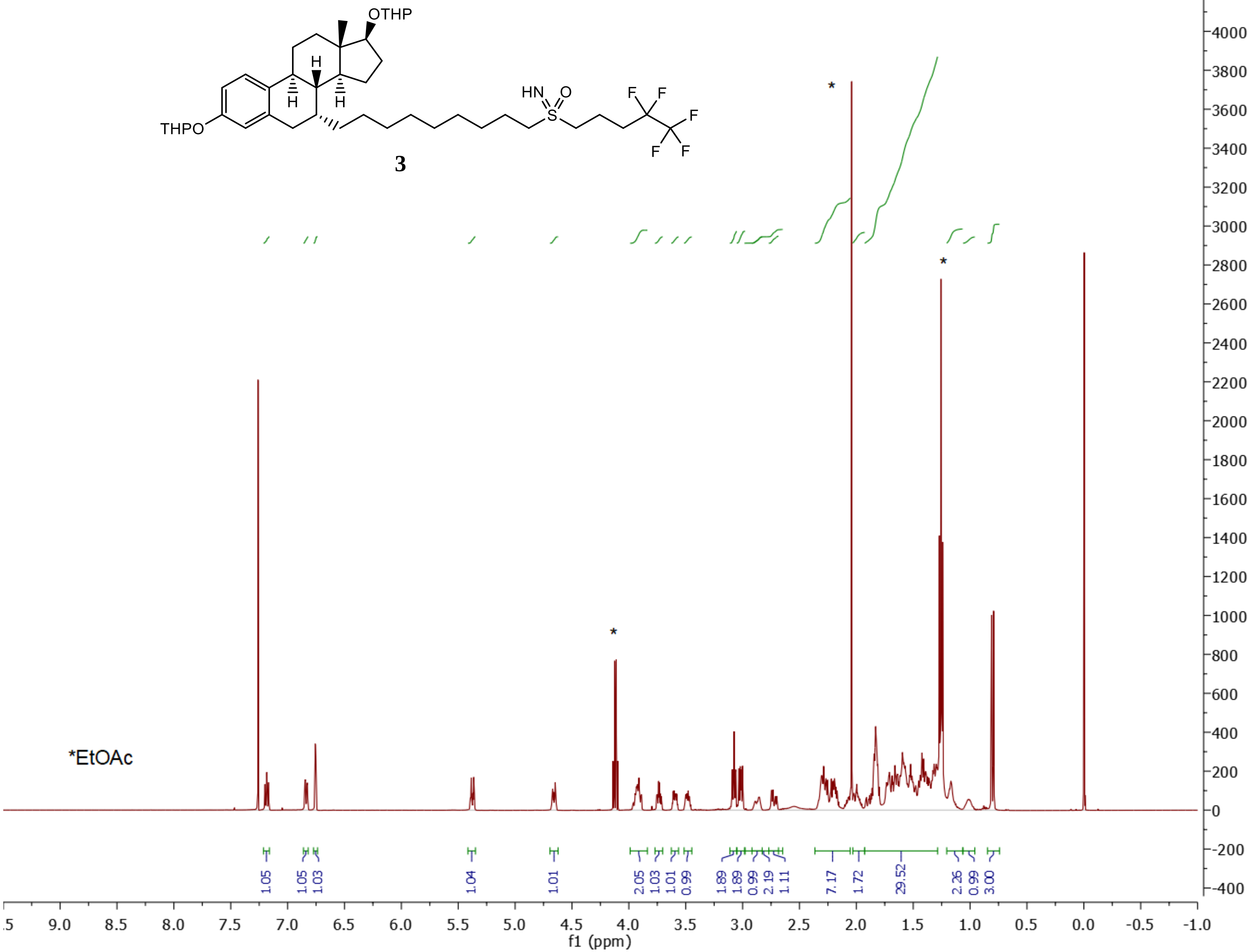

^{13}C, $CDCl_3$, 126 MHz

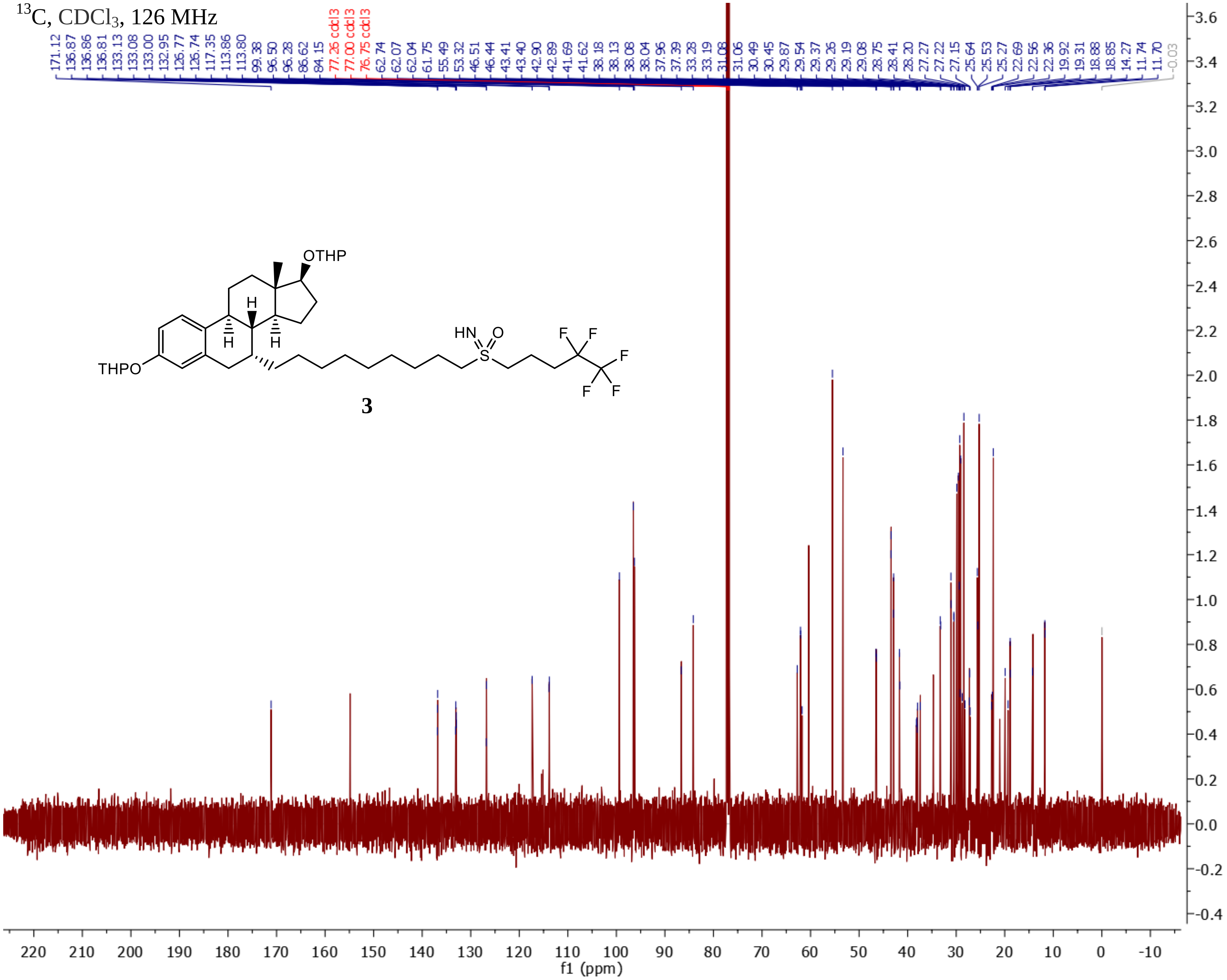

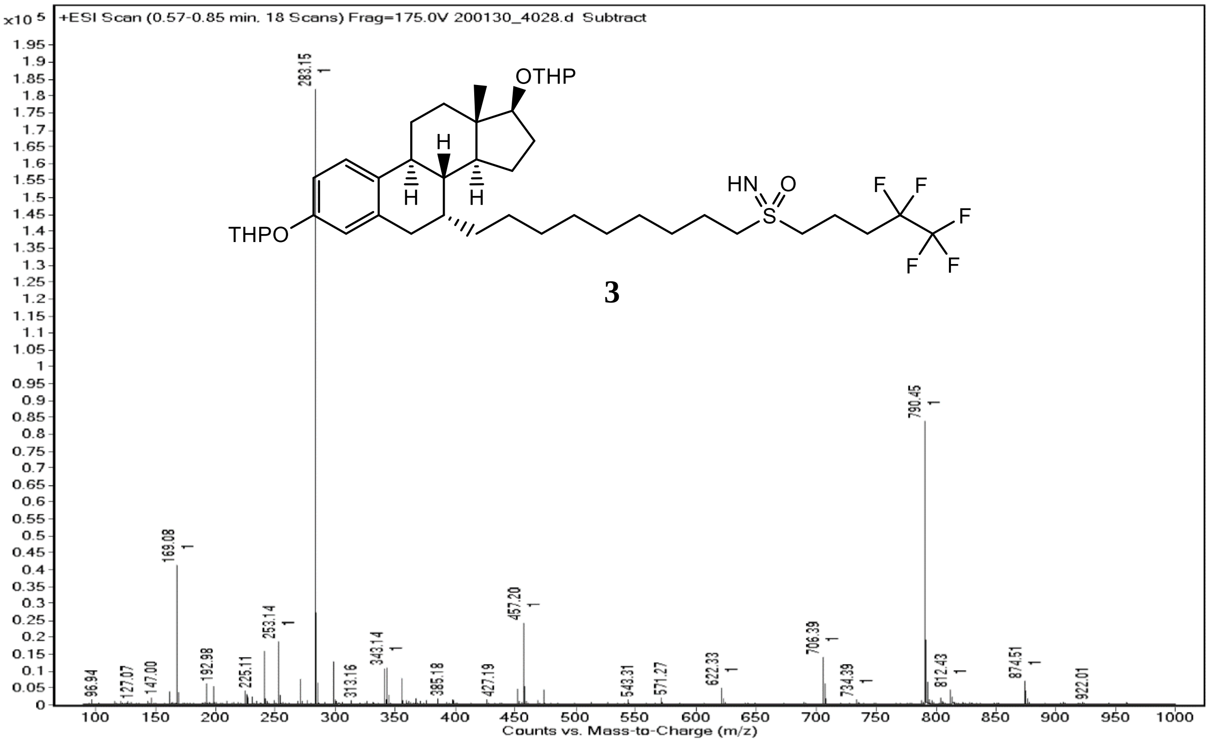

Target Ion Species

Ion Species	m/z	Ionic Formula
(M+H)+	790.4503	C42 H65 F5 N O5 S

MFG Calculator Results

Target m/z	Ionic Formula	Calc m/z	+/- (mDa)	+/- (ppm)	DBE	MFG Score
790.4503	C43 H61 F5 N5 O S	790.4511	-0.8	-1.0	14.0	94.99
790.4503	C42 H65 F5 N O5 S	790.4498	0.5	0.6	9.0	94.64
790.4503	C34 H61 F5 N7 O8	790.4496	0.7	0.9	6.0	82.97
790.4503	C46 H57 F5 N5 O	790.4478	2.5	3.2	19.0	81.87
790.4503	C31 H65 F5 N7 O8 S	790.4530	-2.7	-3.4	1.0	80.55
790.4503	C38 H65 F5 N O10	790.4523	-2.0	-2.5	5.0	80.49
790.4503	C38 H61 F5 N7 O3 S	790.4471	3.2	4.0	10.0	78.97
790.4503	C45 H61 F5 N O5	790.4464	3.9	4.9	14.0	76.16
790.4503	C39 H61 F5 N5 O6	790.4537	-3.4	-4.3	10.0	73.98
790.4503	C37 H65 F5 N3 O7 S	790.4458	4.5	5.7	5.0	70.26

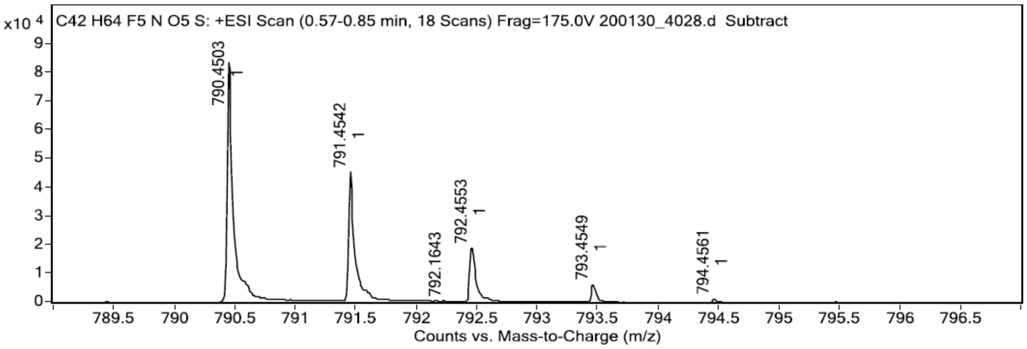

Predicted Isotope Match Table

Isotope	m/z	Calc m/z	Diff (mDa)	Abund (%)	Calc Abund (%)	+/-
1	790.4503	790.4498	0.5	100.0	100.0	0.0
2	791.4542	791.4531	1.1	52.6	47.5	-5.1
3	792.4553	792.4533	2.0	22.3	16.5	-5.8
4	793.4549	793.4541	0.8	7.5	4.3	-3.2
5	794.4561	794.4556	0.5	1.7	0.8	-0.9
6	795.4601	795.4574	2.7	0.3	0.1	-0.2

4

^{1}H, $CDCl_3$, 500 MHz

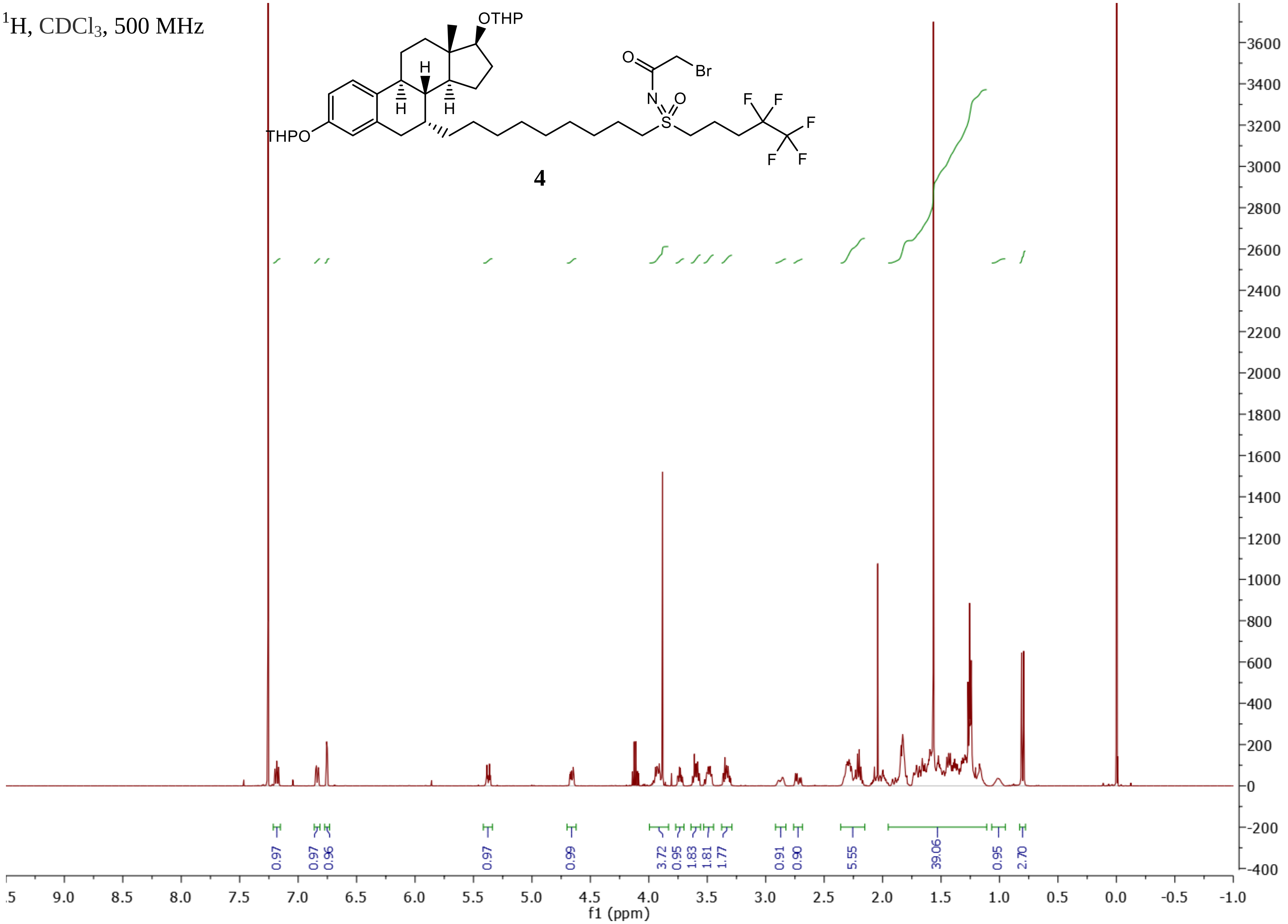

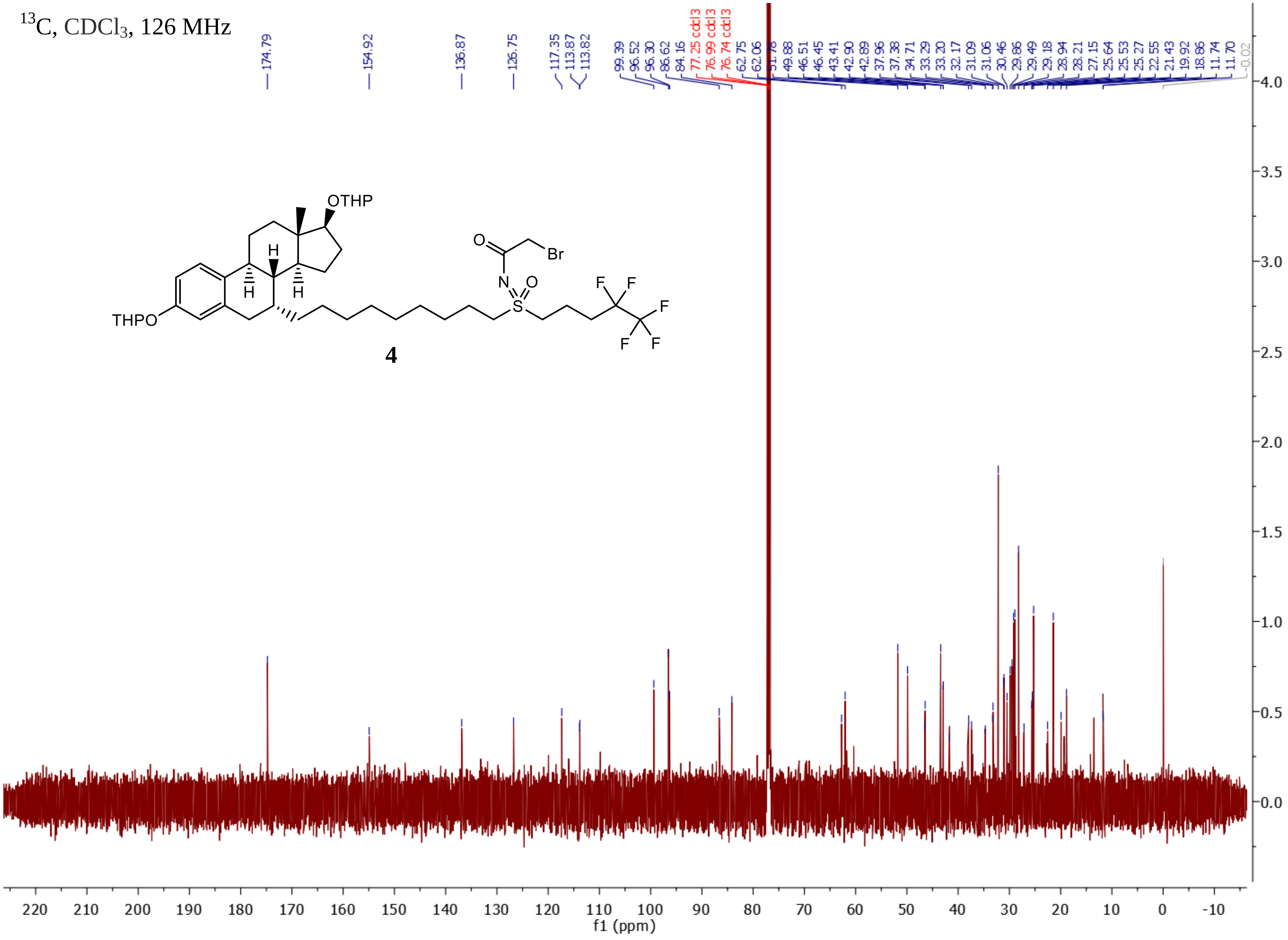
13C, CDCl3, 126 MHz
174.79
154.92
136.87
126.75
117.35
113.87
113.82
99.39
96.52
96.30
86.62
84.16
77.25 cdcl3
76.99 cdcl3
76.74 cdcl3
62.75
62.06
51.78
49.88
46.51
46.45
43.41
42.90
42.89
37.96
37.38
34.71
33.29
33.20
32.17
31.09
31.06
30.46
29.86
29.49
29.18
28.94
28.21
27.15
25.64
25.53
25.27
22.55
21.43
19.92
18.86
11.74
11.70
-0.02
OTHP
H
H
H
THPO
O
Br
N
O
S
F
F
F
F
F
4
f1 (ppm)

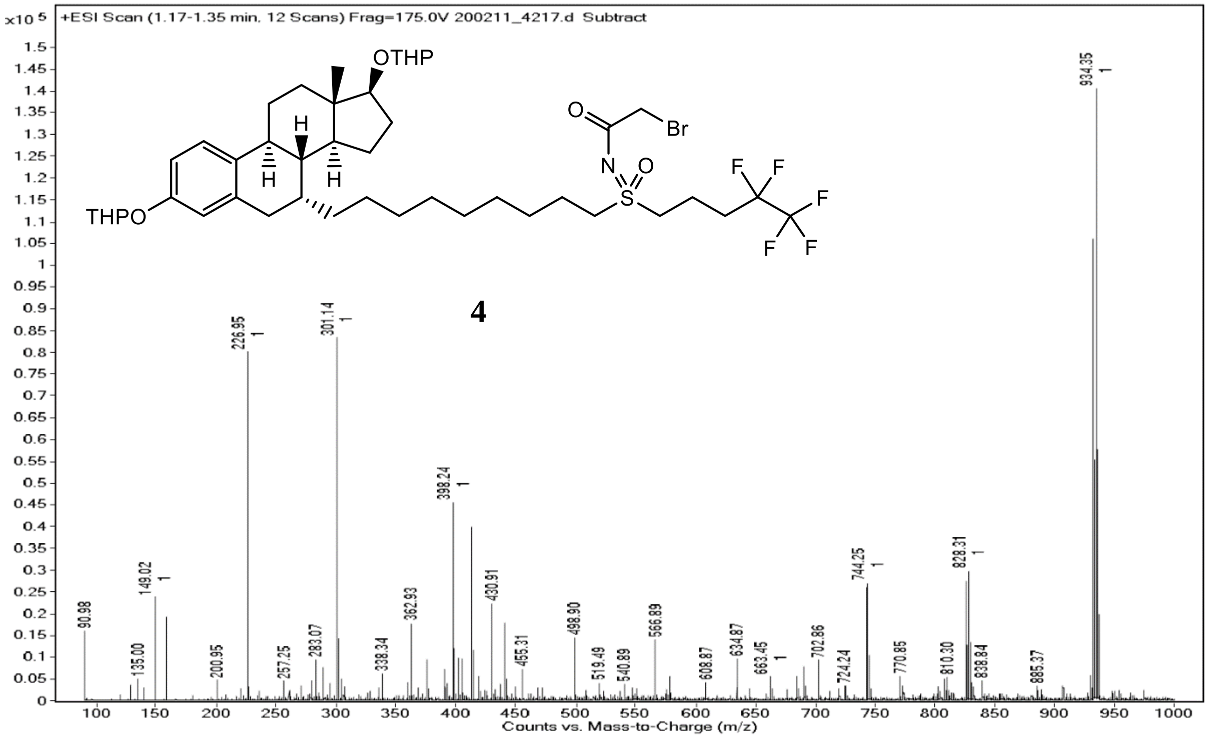

Target Ion Species

Ion Species	m/z	Ionic Formula
(M+Na)+	932.3529	C44 H65 Br F5 N Na O6 S

MFG Calculator Results

Target m/z	Ionic Formula	Calc m/z	+/- (mDa)	+/- (ppm)	DBE	MFG Score
932.3529	C44 H65 Br F5 N Na O6 S	932.3528	0.1	0.1	10.0	97.84
932.3529	C45 H61 Br F5 N5 Na O2 S	932.3542	-1.3	-1.4	15.0	96.25
932.3529	C48 H57 Br F5 N5 Na O2	932.3508	2.1	2.3	20.0	93.73
932.3529	C40 H61 Br F5 N7 Na O4 S	932.3501	2.8	3.0	11.0	90.48
932.3529	C53 H57 Br F5 N3 Na	932.3548	-1.9	-2.0	24.0	89.46
932.3529	C47 H61 Br F5 N Na O6	932.3495	3.4	3.6	15.0	89.12
932.3529	C36 H61 Br F5 N7 Na O9	932.3527	0.2	0.2	7.0	87.07
932.3529	C39 H65 Br F5 N3 Na O8 S	932.3488	4.1	4.4	6.0	82.87
932.3529	C33 H65 Br F5 N7 Na O9 S	932.3560	-3.1	-3.3	2.0	80.47
932.3529	C41 H61 Br F5 N5 Na O7	932.3567	-3.8	-4.1	11.0	77.80

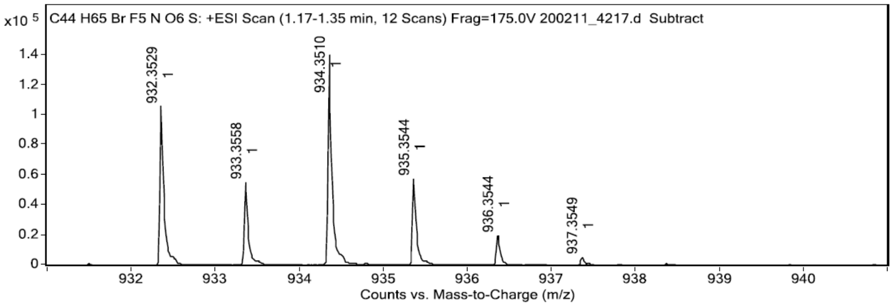

Predicted Isotope Match Table

Isotope	m/z	Calc m/z	Diff (mDa)	Abund (%)	Calc Abund (%)	+/-
1	932.3529	932.3528	0.1	75.7	86.9	11.2
2	933.3558	933.3561	-0.3	40.2	43.2	3.0
3	934.3510	934.3517	-0.7	100.0	100.0	0.0
4	935.3544	935.3544	0.0	42.6	46.1	3.5
5	936.3544	936.3547	-0.3	14.4	15.9	1.5
6	937.3549	937.3556	-0.7	3.8	4.1	0.3

5

^{1}H, $CDCl_3$, 500 MHz

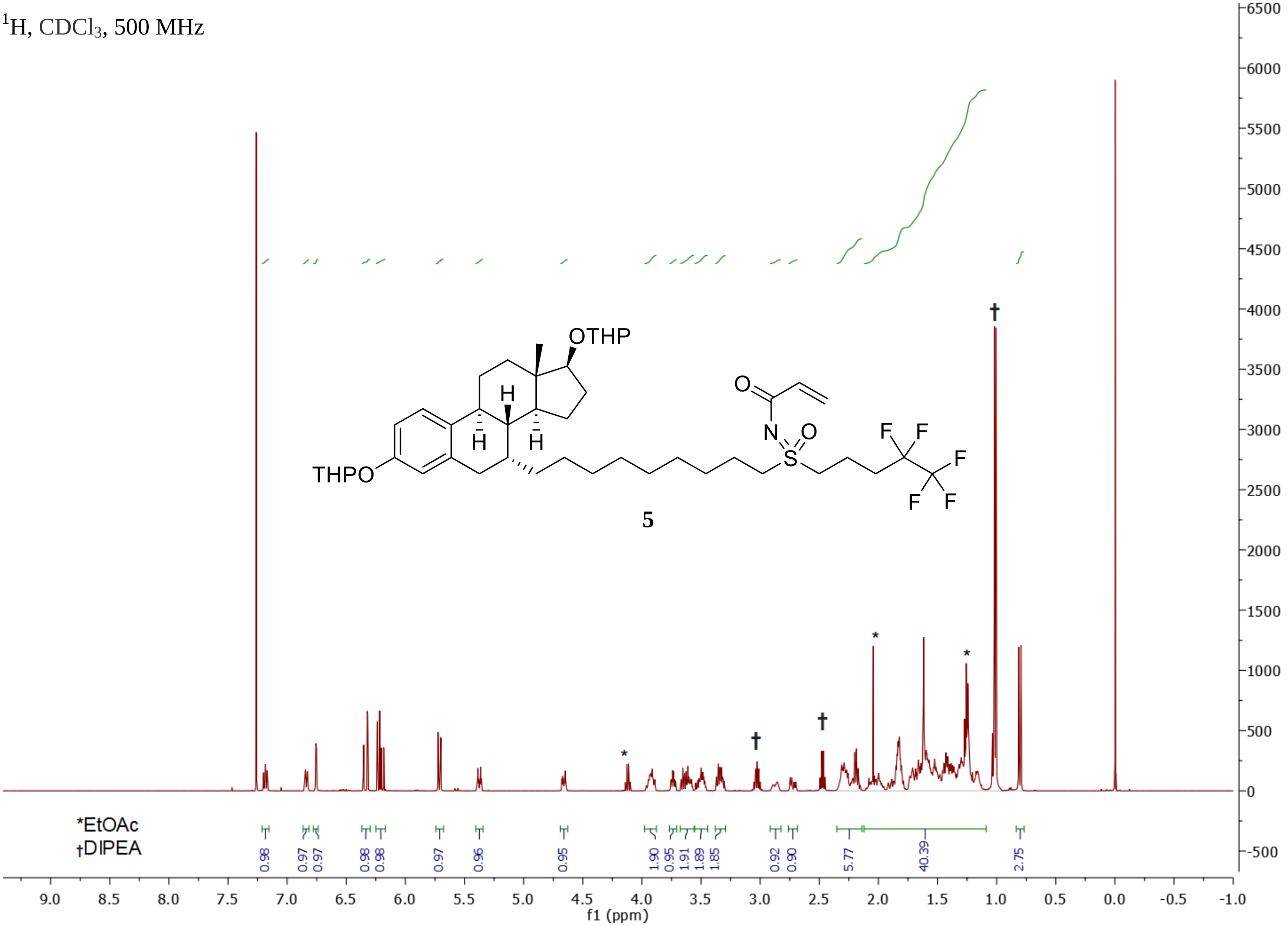

^{13}C, $CDCl_3$, 126 MHz

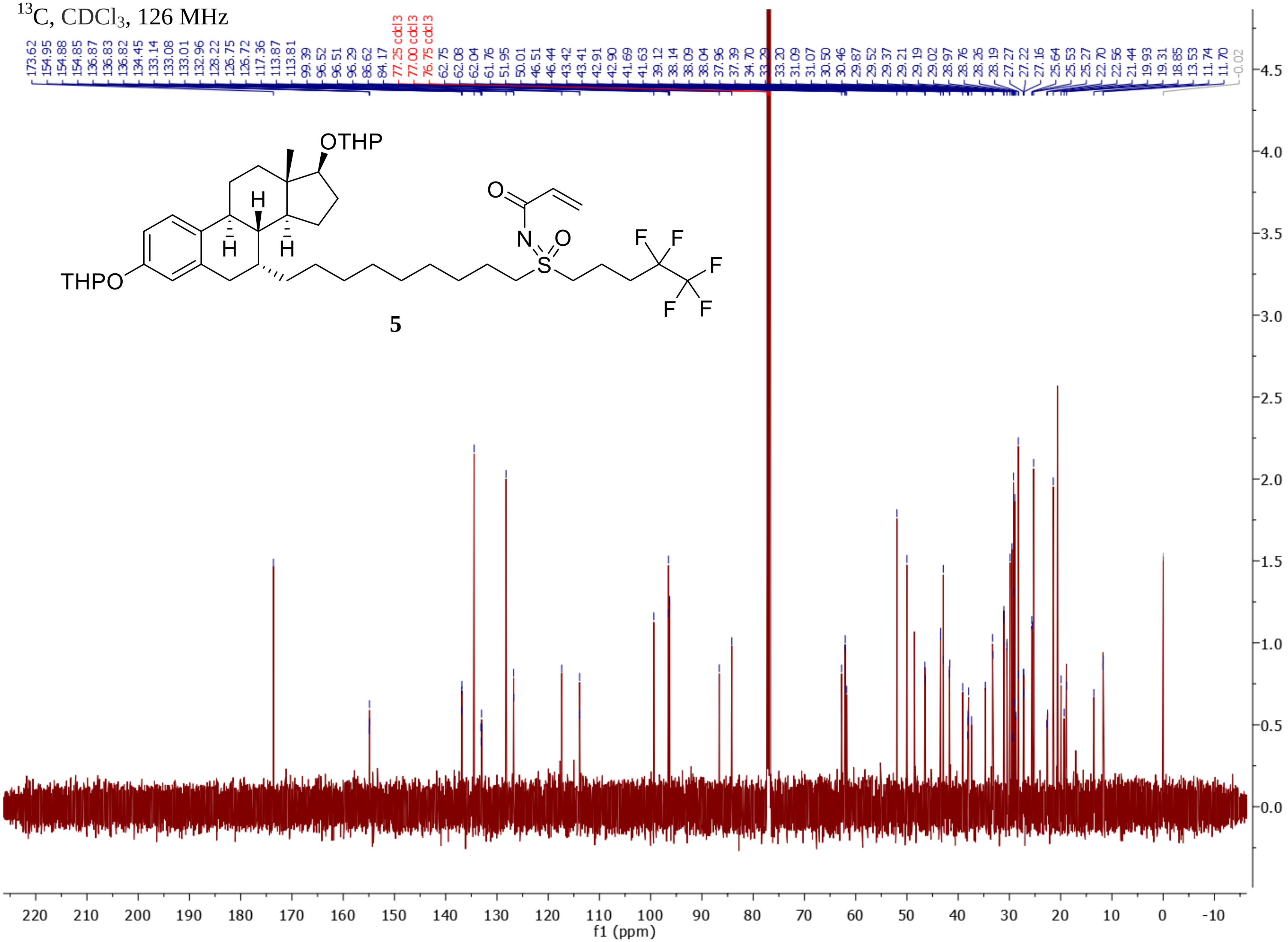

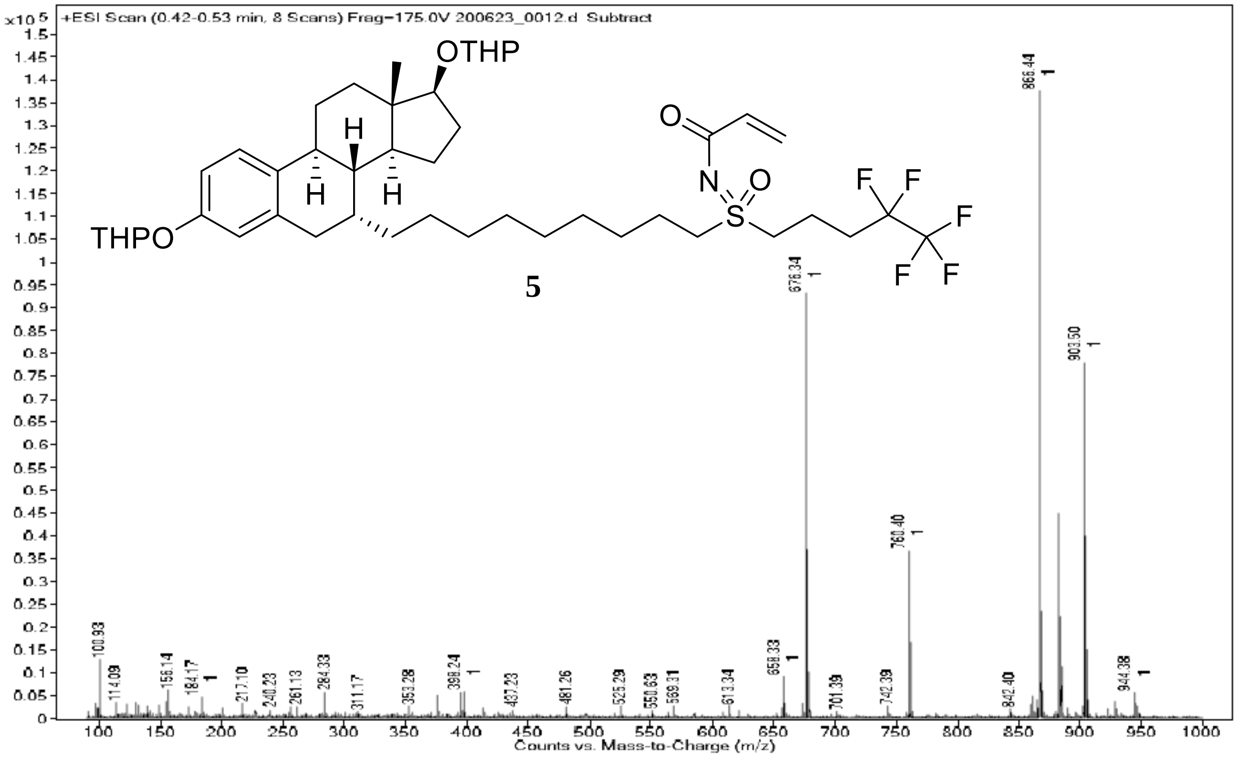
+ESI Scan (0.42-0.53 min, 8 Scans) Frag=175.0V 200623_0012.d Subtract
x10^5
OTHP
THPO
O
N
S
O
F
5
866.44
903.50
676.34
760.40
100.93
114.09
156.14
184.17
217.10
240.23
261.13
284.33
311.17
353.28
398.24
437.23
481.26
525.29
550.63
569.31
613.34
658.33
701.39
742.39
842.40
944.38
Counts vs. Mass-to-Charge (m/z)

6a

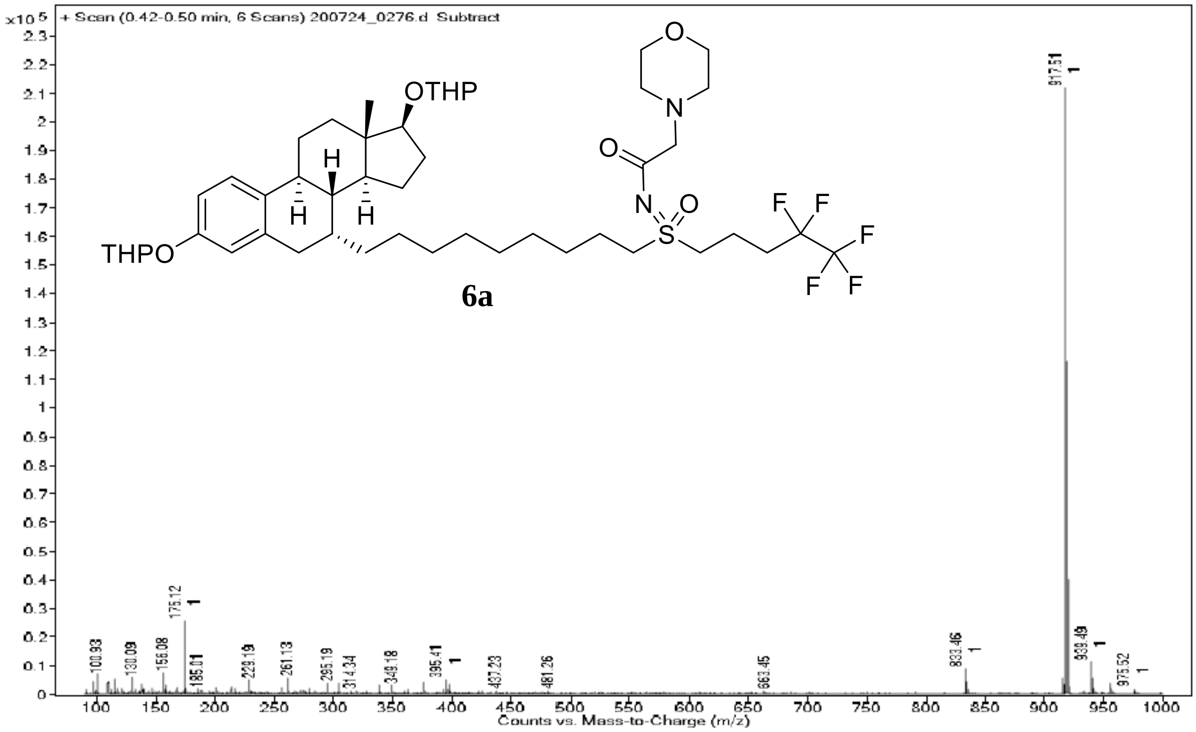

Target Ion Species

Ion Species	m/z	Ionic Formula
(M+H)+	917.5136	C48 H74 F5 N2 O7 S

MFG Calculator Results

Target m/z	Ionic Formula	Calc m/z	+/- (mDa)	+/- (ppm)	DBE	MFG Score
917.5136	C48 H74 F5 N2 O7 S	917.5131	0.5	0.5	11.0	99.29
917.5136	C49 H70 F5 N6 O3 S	917.5145	-0.9	-1.0	16.0	98.40
917.5136	C40 H70 F5 N8 O10	917.5130	0.6	0.7	8.0	94.37
917.5136	C56 H70 F5 O5	917.5138	-0.2	-0.2	20.0	94.22
917.5136	C52 H66 F5 N6 O3	917.5111	2.5	2.7	21.0	92.78
917.5136	C44 H70 F5 N8 O5 S	917.5105	3.1	3.4	12.0	91.49
917.5136	C60 H70 F5 S	917.5113	2.3	2.5	24.0	90.52
917.5136	C57 H66 F5 N4 O	917.5151	-1.5	-1.6	25.0	88.96
917.5136	C51 H70 F5 N2 O7	917.5098	3.8	4.1	16.0	88.71
917.5136	C37 H74 F5 N8 O10 S	917.5163	-2.7	-2.9	3.0	87.52

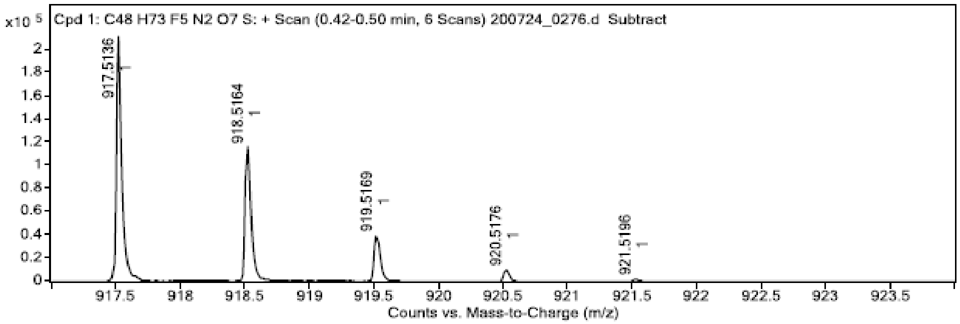

Predicted Isotope Match Table

Isotope	m/z	Calc m/z	Diff (mDa)	Abund (%)	Calc Abund (%)	+/-
1	917.5136	917.5131	0.5	100.0	100.0	0.0
2	918.5164	918.5164	0.0	54.9	54.6	-0.3
3	919.5169	919.5172	-0.3	18.9	20.5	1.6
4	920.5176	920.5181	-0.5	4.9	5.7	0.8
5	921.5196	921.5196	0.0	1.2	1.3	0.1
6	922.5162	922.5214	-5.2	0.1	0.2	0.1

6b

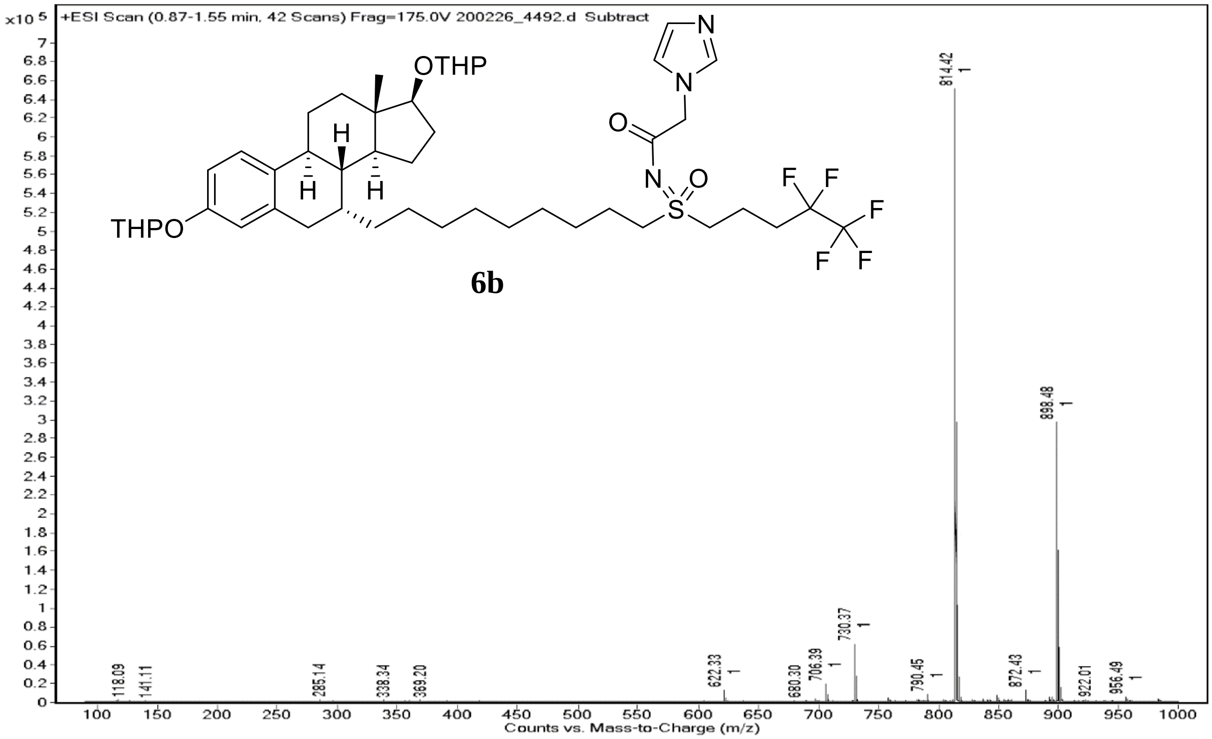

Target Ion Species

Ion Species	m/z	Ionic Formula
(M+H)+	898.482	C47 H69 F5 N3 O6 S

MFG Calculator Results

Target m/z	Ionic Formula	Calc m/z	+/- (mDa)	+/- (ppm)	DBE	MFG Score
898.4820	C47 H69 F5 N3 O6 S	898.4822	-0.2	-0.2	13.0	99.69
898.4820	C48 H65 F5 N7 O2 S	898.4835	-1.5	-1.7	18.0	96.90
898.4820	C39 H65 F5 N9 O9	898.4820	0.0	0.0	10.0	94.42
898.4820	C43 H65 F5 N9 O4 S	898.4795	2.5	2.8	14.0	93.95
898.4820	C51 H61 F5 N7 O2	898.4801	1.9	2.1	23.0	91.91
898.4820	C55 H65 F5 N O4	898.4828	-0.8	-0.9	22.0	90.87
898.4820	C50 H65 F5 N3 O6	898.4788	3.2	3.6	18.0	89.11
898.4820	C42 H69 F5 N5 O8 S	898.4782	3.8	4.2	9.0	87.12
898.4820	C36 H69 F5 N9 O9 S	898.4854	-3.4	-3.8	5.0	86.98
898.4820	C56 H61 F5 N5	898.4842	-2.2	-2.4	27.0	84.19

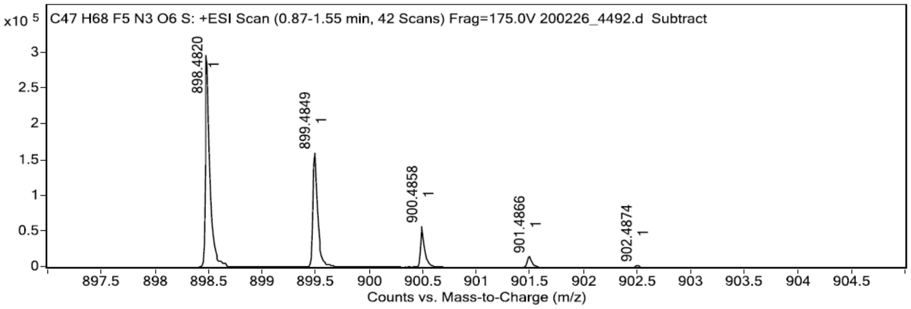

Predicted Isotope Match Table

Isotope	m/z	Calc m/z	Diff (mDa)	Abund (%)	Calc Abund (%)	+/-
1	898.4820	898.4822	-0.2	100.0	100.0	0.0
2	899.4849	899.4854	-0.5	52.1	53.7	1.6
3	900.4858	900.4861	-0.3	18.7	19.9	1.2
4	901.4866	901.4870	-0.4	5.0	5.5	0.5
5	902.4874	902.4884	-1.0	1.1	1.2	0.1
6	903.4873	903.4901	-2.8	0.2	0.2	0.0

6c

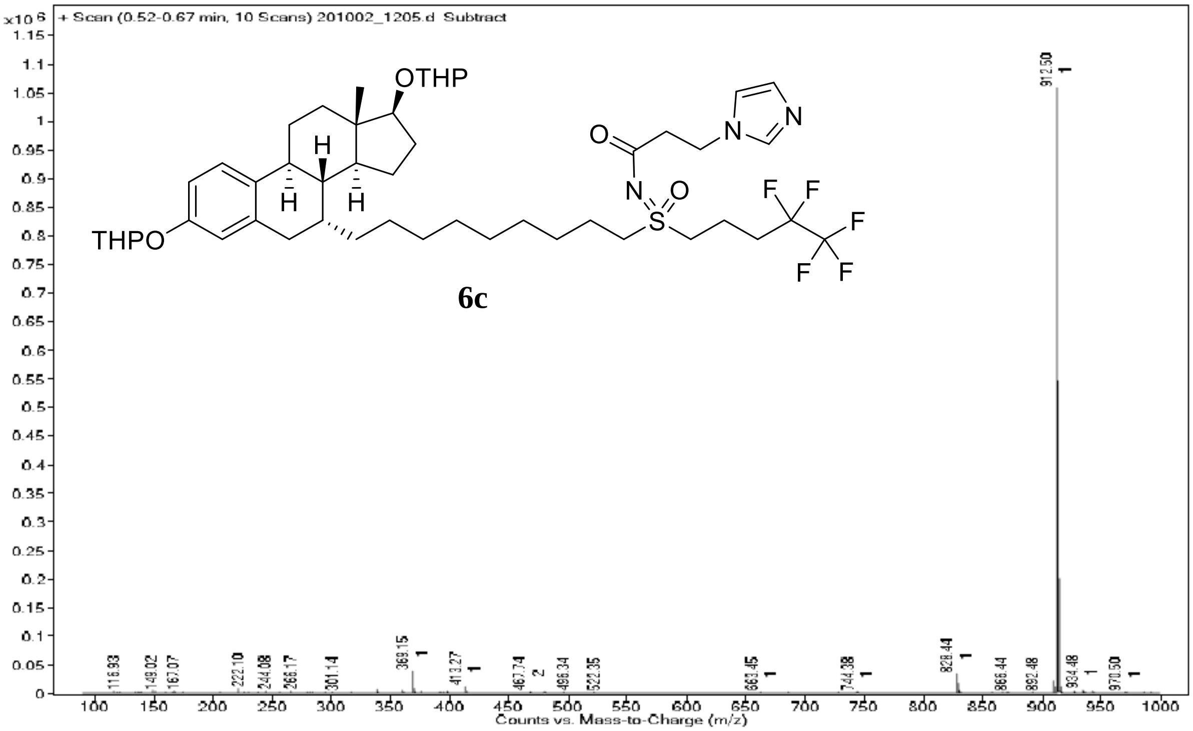

Target Ion Species

Ion Species	m/z	Ionic Formula
(M+H)+	912.4969	C48 H71 F5 N3 O6 S

MFG Calculator Results

Target m/z	Ionic Formula	Calc m/z	+/- (mDa)	+/- (ppm)	DBE	MFG Score
912.4969	C48 H71 F5 N3 O6 S	912.4978	-0.9	-1.0	13.0	98.83
912.4969	C44 H67 F5 N9 O4 S	912.4951	1.8	2.0	14.0	95.76
912.4969	C40 H67 F5 N9 O9	912.4976	-0.7	-0.8	10.0	94.77
912.4969	C49 H67 F5 N7 O2 S	912.4992	-2.3	-2.5	18.0	94.57
912.4969	C52 H63 F5 N7 O2	912.4958	1.1	1.2	23.0	92.48
912.4969	C51 H67 F5 N3 O6	912.4945	2.4	2.6	18.0	91.01
912.4969	C43 H71 F5 N5 O8 S	912.4938	3.1	3.4	9.0	90.20
912.4969	C56 H67 F5 N O4	912.4985	-1.6	-1.8	22.0	88.98
912.4969	C37 H71 F5 N9 O9 S	912.5010	-4.1	-4.5	5.0	85.45
912.4969	C57 H63 F5 N5	912.4998	-2.9	-3.2	27.0	81.41

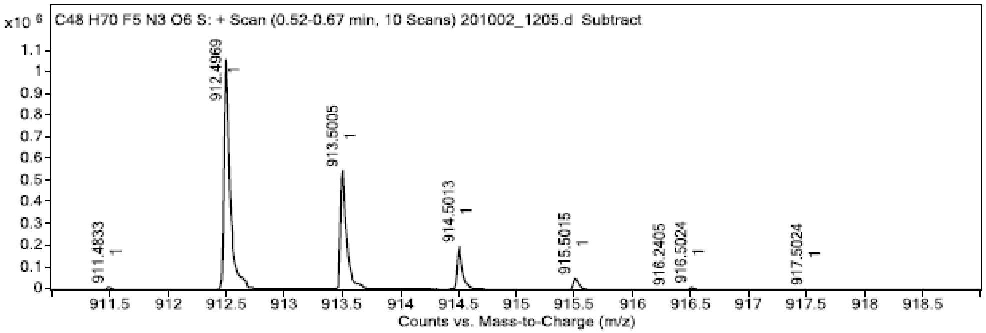

Predicted Isotope Match Table

Isotope	m/z	Calc m/z	Diff (mDa)	Abund (%)	Calc Abund (%)	+/-
1	912.4969	912.4978	-0.9	100.0	100.0	0.0
2	913.5005	913.5010	-0.5	51.8	54.8	3.0
3	914.5013	914.5018	-0.5	18.7	20.5	1.8
4	915.5015	915.5027	-1.2	4.9	5.7	0.8
5	916.5024	916.5041	-1.7	1.1	1.2	0.1
6	917.5024	917.5059	-3.5	0.2	0.2	0.0

6d

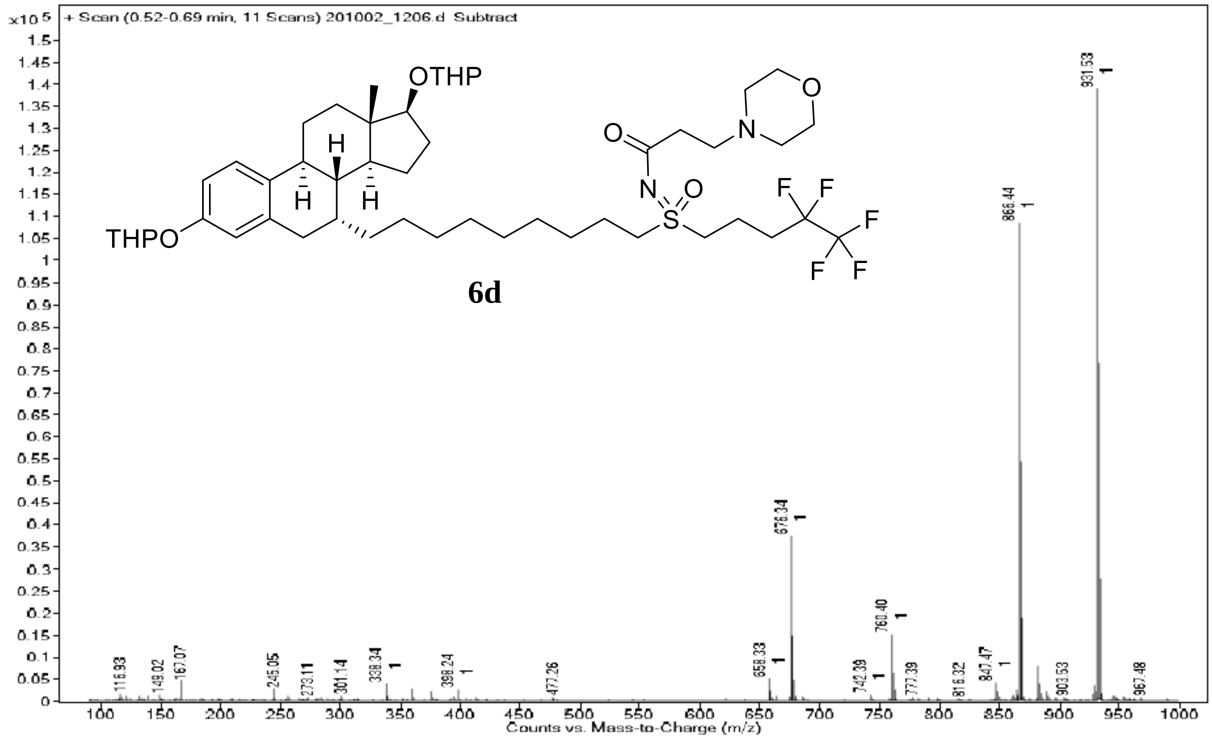

Target Ion Species

Ion Species	*m/z*	*Ionic Formula*
(M+H)+	931.5285	C49 H76 F5 N2 O7 S

MFG Calculator Results

Target m/z	*Ionic Formula*	*Calc m/z*	*+/- (mDa)*	*+/- (ppm)*	*DBE*	*MFG Score*
931.5285	C49 H76 F5 N2 O7 S	931.5288	-0.3	-0.3	11.0	99.25
931.5285	C50 H72 F5 N6 O3 S	931.5301	-1.6	-1.7	16.0	96.05
931.5285	C45 H72 F5 N8 O5 S	931.5261	2.4	2.6	12.0	95.09
931.5285	C41 H72 F5 N8 O10	931.5286	-0.1	-0.1	8.0	94.92
931.5285	C53 H68 F5 N6 O3	931.5268	1.7	1.8	21.0	92.19
931.5285	C61 H72 F5 S	931.5269	1.6	1.7	24.0	90.43
931.5285	C57 H72 F5 O5	931.5294	-0.9	-1.0	20.0	90.11
931.5285	C52 H72 F5 N2 O7	931.5254	3.1	3.3	16.0	89.96
931.5285	C44 H76 F5 N4 O9 S	931.5248	3.7	4.0	7.0	88.94
931.5285	C38 H76 F5 N8 O10 S	931.5320	-3.5	-3.8	3.0	87.42

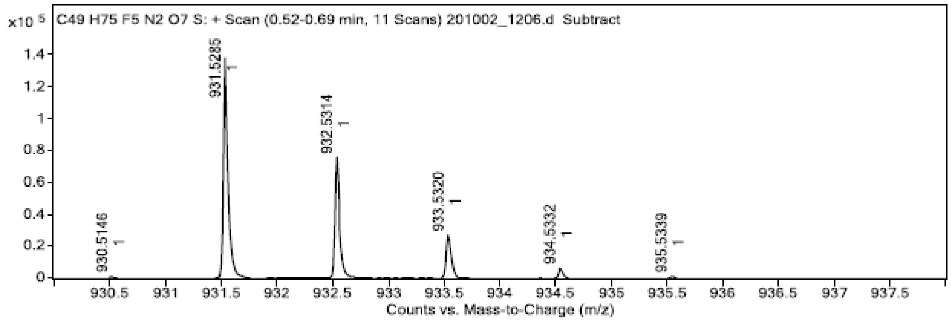

Predicted Isotope Match Table

Isotope	*m/z*	*Calc m/z*	Diff (mDa)	Abund (%)	Calc Abund (%)	+/-
1	931.5285	931.5288	-0.3	100.0	100.0	0.0
2	932.5314	932.5321	-0.7	53.1	55.7	2.6
3	933.5320	933.5329	-0.9	19.3	21.1	1.8
4	934.5332	934.5339	-0.7	5.0	6.0	1.0
5	935.5339	935.5353	-1.4	1.2	1.3	0.1
6	936.5313	936.5371	-5.8	0.2	0.2	0.0

6e

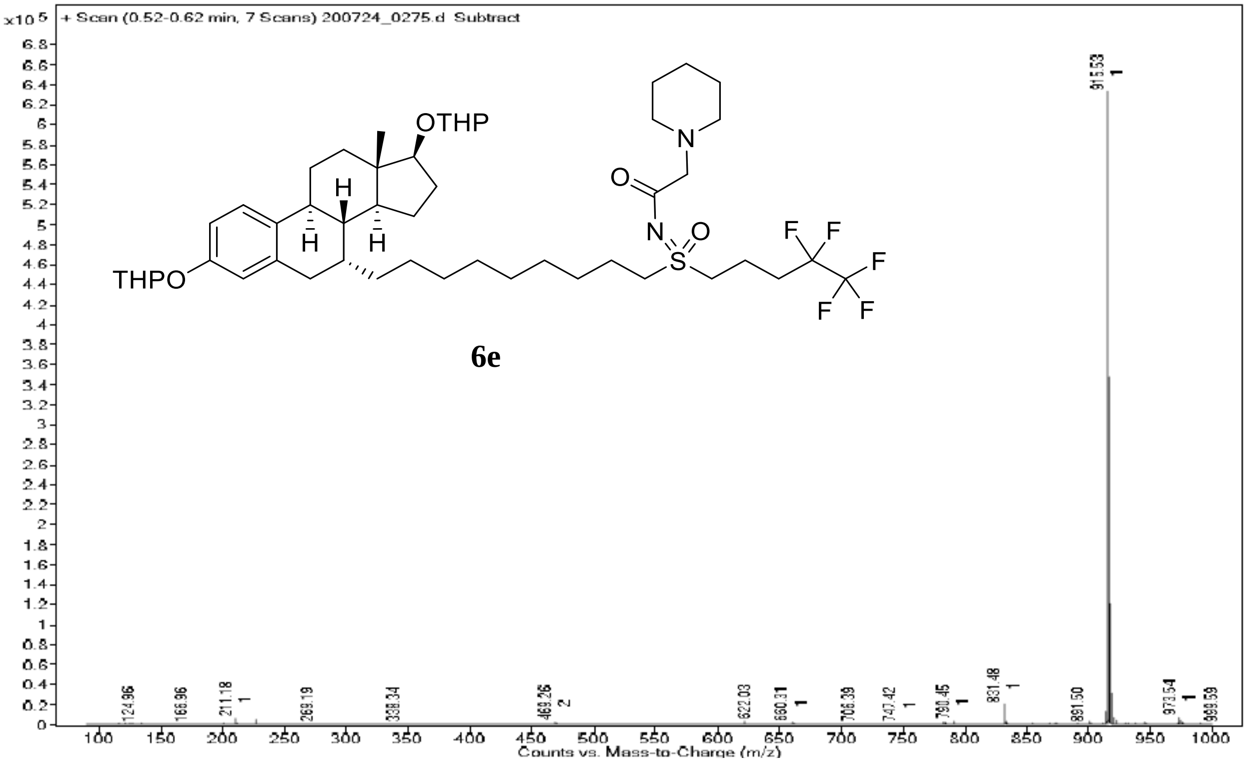

Target Ion Species

Ion Species	m/z	Ionic Formula
(M+H)+	915.5339	C49 H76 F5 N2 O6 S

MFG Calculator Results

Target m/z	Ionic Formula	Calc m/z	+/- (mDa)	+/- (ppm)	DBE	MFG Score
915.5339	C49 H76 F5 N2 O6 S	915.5339	0.0	0.0	11.0	99.61
915.5339	C50 H72 F5 N6 O2 S	915.5352	-1.3	-1.4	16.0	98.03
915.5339	C41 H72 F5 N8 O9	915.5337	0.2	0.2	8.0	95.24
915.5339	C57 H72 F5 O4	915.5345	-0.6	-0.7	20.0	94.15
915.5339	C53 H68 F5 N6 O2	915.5318	2.1	2.3	21.0	93.18
915.5339	C45 H72 F5 N8 O4 S	915.5312	2.7	2.9	12.0	92.22
915.5339	C52 H72 F5 N2 O6	915.5305	3.4	3.7	16.0	89.58
915.5339	C58 H68 F5 N4	915.5359	-2.0	-2.2	25.0	88.44
915.5339	C38 H76 F5 N8 O9 S	915.5371	-3.2	-3.5	3.0	87.67
915.5339	C54 H76 F5 O4 S	915.5379	-4.0	-4.4	15.0	85.24

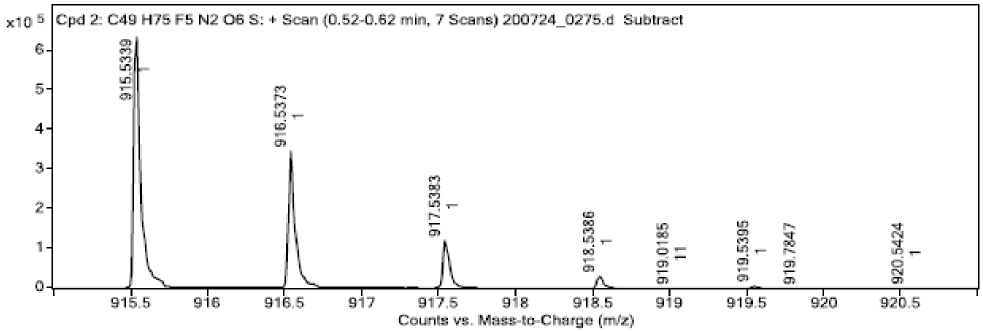

Predicted Isotope Match Table

Isotope	m/z	Calc m/z	Diff (mDa)	Abund (%)	Calc Abund (%)	+/-
1	915.5339	915.5339	0.0	100.0	100.0	0.0
2	916.5373	916.5371	0.2	55.0	55.6	0.6
3	917.5383	917.5380	0.3	19.3	20.9	1.6
4	918.5386	918.5389	-0.3	4.9	5.8	0.9
5	919.5395	919.5404	-0.9	1.0	1.3	0.3
6	920.5424	920.5422	0.2	0.2	0.2	0.0

6f

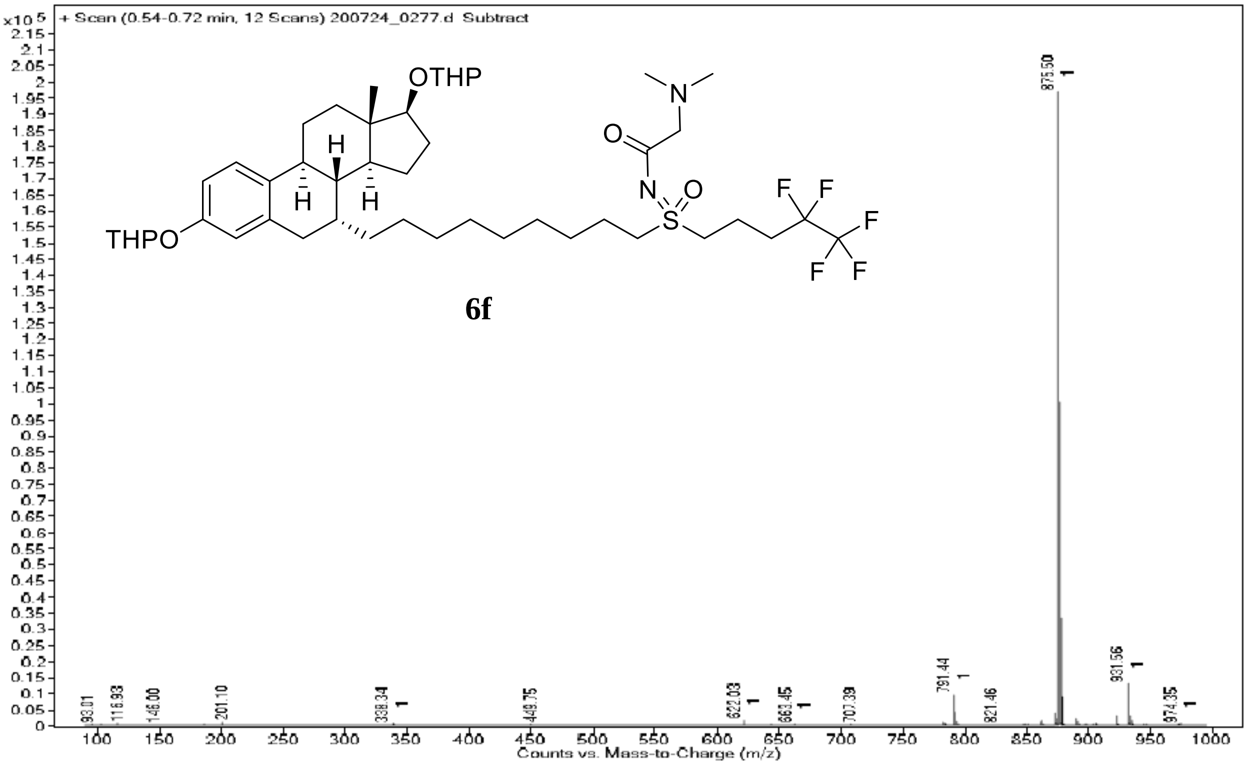

Target Ion Species

Ion Species	*m/z*	*Ionic Formula*
(M+H)+	875.5032	C46 H72 F5 N2 O6 S

MFG Calculator Results

Target m/z	*Ionic Formula*	*Calc m/z*	*+/- (mDa)*	*+/- (ppm)*	*DBE*	*MFG Score*
875.5032	C46 H72 F5 N2 O6 S	875.5026	0.6	0.7	10.0	99.05
875.5032	C47 H68 F5 N6 O2 S	875.5039	-0.7	-0.8	15.0	98.72
875.5032	C38 H68 F5 N8 O9	875.5024	0.8	0.9	7.0	94.57
875.5032	C54 H68 F5 O4	875.5032	0.0	0.0	19.0	94.18
875.5032	C50 H64 F5 N6 O2	875.5005	2.7	3.1	20.0	91.17
875.5032	C42 H68 F5 N8 O4 S	875.4999	3.3	3.8	11.0	89.43
875.5032	C55 H64 F5 N4	875.5046	-1.4	-1.6	24.0	89.16
875.5032	C35 H72 F5 N8 O9 S	875.5058	-2.6	-3.0	2.0	88.31
875.5032	C51 H72 F5 O4 S	875.5066	-3.4	-3.9	14.0	86.58
875.5032	C49 H68 F5 N2 O6	875.4992	4.0	4.6	15.0	86.51

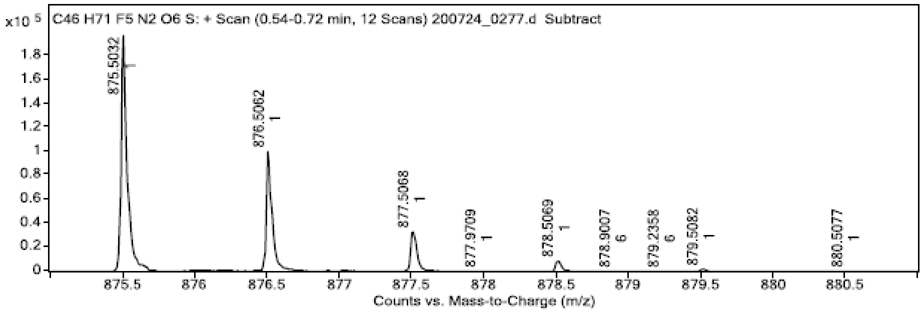

Predicted Isotope Match Table

Isotope	*m/z*	*Calc m/z*	Diff (mDa)	Abund (%)	Calc Abund (%)	+/-
1	875.5032	875.5026	0.6	100.0	100.0	0.0
2	876.5062	876.5058	0.4	52.2	52.3	0.1
3	877.5068	877.5064	0.4	17.1	19.1	2.0
4	878.5069	878.5073	-0.4	4.5	5.2	0.7
5	879.5082	879.5088	-0.6	0.9	1.1	0.2
6	880.5077	880.5106	-2.9	0.2	0.2	0.0

6g

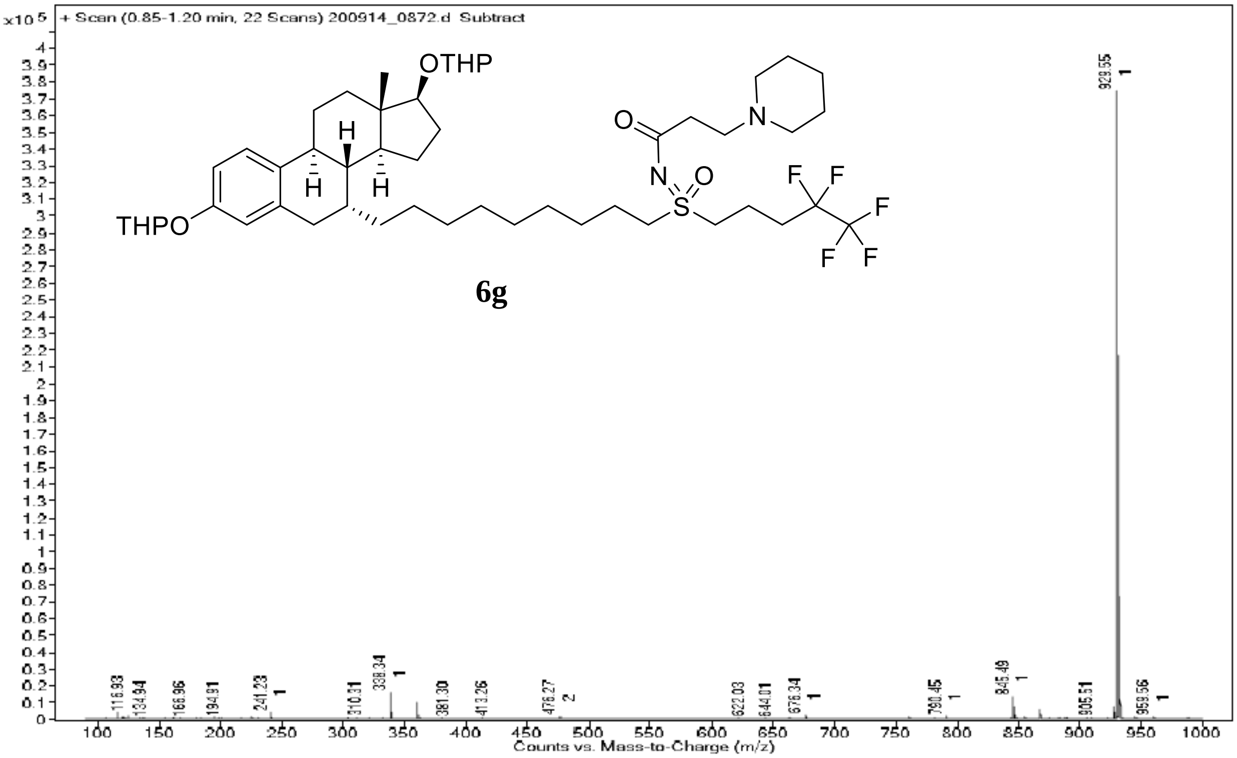

Target Ion Species

Ion Species	m/z	Ionic Formula
(M+H)+	929.5488	C50 H78 F5 N2 O6 S

MFG Calculator Results

Target m/z	Ionic Formula	Calc m/z	+/- (mDa)	+/- (ppm)	DBE	MFG Score
929.5488	C50 H78 F5 N2 O6 S	929.5495	-0.7	-0.8	11.0	98.58
929.5488	C46 H74 F5 N8 O4 S	929.5468	2.0	2.2	12.0	96.31
929.5488	C51 H74 F5 N6 O2 S	929.5509	-2.1	-2.3	16.0	95.06
929.5488	C54 H70 F5 N6 O2	929.5475	1.3	1.4	21.0	95.02
929.5488	C42 H74 F5 N8 O9	929.5493	-0.5	-0.5	8.0	94.67
929.5488	C53 H74 F5 N2 O6	929.5462	2.6	2.8	16.0	93.17
929.5488	C43 H78 F5 N8 O4 S2	929.5502	-1.4	-1.5	7.0	93.16
929.5488	C42 H82 F5 N4 O8 S2	929.5489	-0.1	-0.1	2.0	92.29
929.5488	C58 H74 F5 O4	929.5502	-1.4	-1.5	20.0	90.97
929.5488	C54 H78 F5 N2 O S2	929.5470	1.8	1.9	15.0	90.85

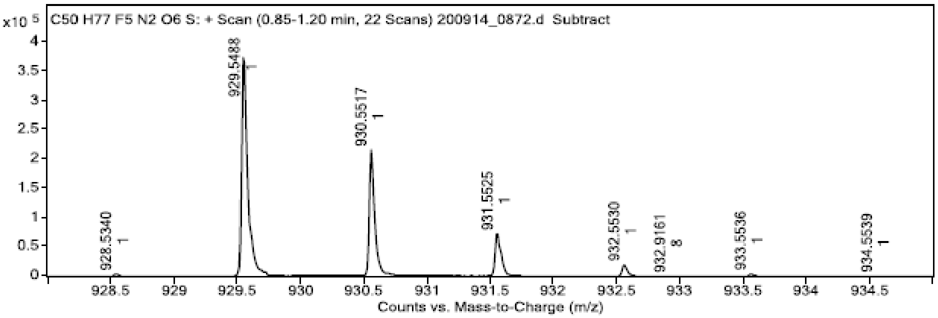

Predicted Isotope Match Table

Isotope	m/z	Calc m/z	Diff (mDa)	Abund (%)	Calc Abund (%)	+/-
1	929.5488	929.5495	-0.7	100.0	100.0	0.0
2	930.5517	930.5528	-1.1	55.8	56.7	0.9
3	931.5525	931.5537	-1.2	19.2	21.5	2.3
4	932.5530	932.5547	-1.7	5.2	6.1	0.9
5	933.5536	933.5561	-2.5	1.2	1.3	0.1

6h

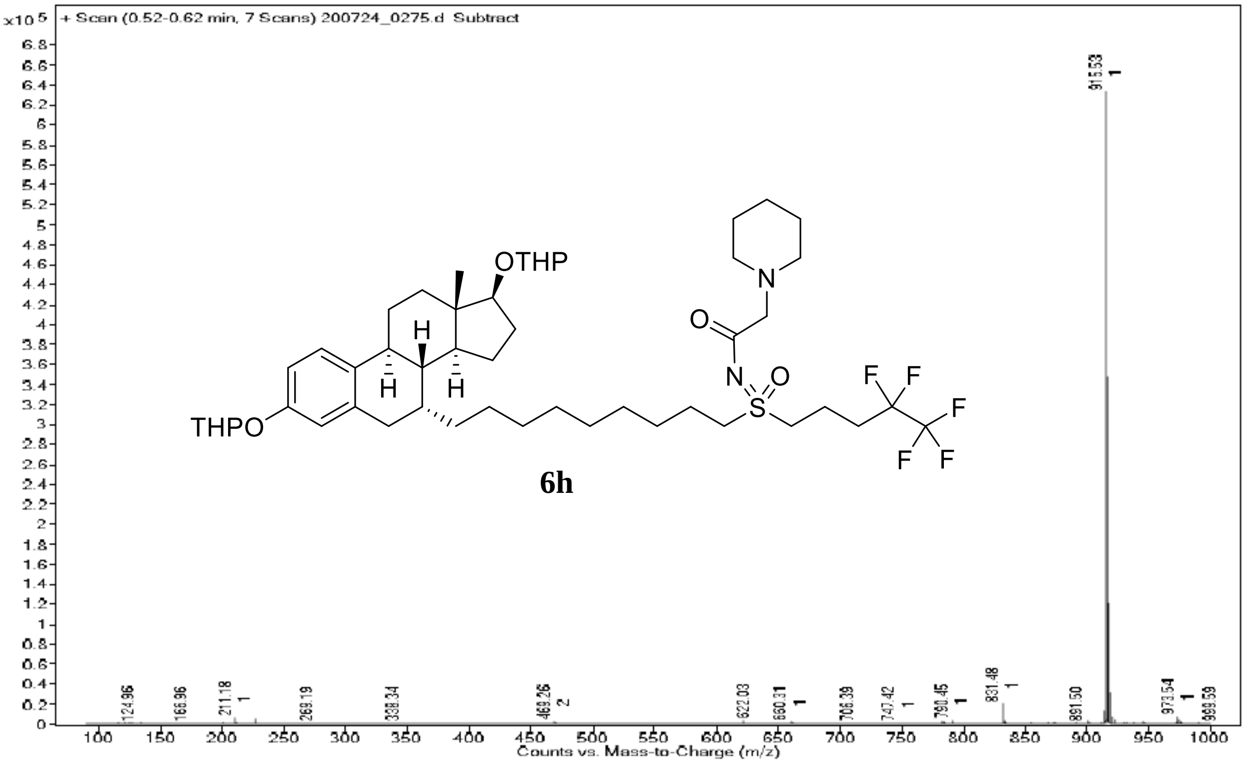

Target Ion Species

Ion Species	m/z	Ionic Formula
(M+H)+	915.5339	C49 H76 F5 N2 O6 S

MFG Calculator Results

Target m/z	Ionic Formula	Calc m/z	+/- (mDa)	+/- (ppm)	DBE	MFG Score
915.5339	C49 H76 F5 N2 O6 S	915.5339	0.0	0.0	11.0	99.61
915.5339	C50 H72 F5 N6 O2 S	915.5352	-1.3	-1.4	16.0	98.03
915.5339	C41 H72 F5 N8 O9	915.5337	0.2	0.2	8.0	95.24
915.5339	C57 H72 F5 O4	915.5345	-0.6	-0.7	20.0	94.15
915.5339	C53 H68 F5 N6 O2	915.5318	2.1	2.3	21.0	93.18
915.5339	C45 H72 F5 N8 O4 S	915.5312	2.7	2.9	12.0	92.22
915.5339	C52 H72 F5 N2 O6	915.5305	3.4	3.7	16.0	89.58
915.5339	C58 H68 F5 N4	915.5359	-2.0	-2.2	25.0	88.44
915.5339	C38 H76 F5 N8 O9 S	915.5371	-3.2	-3.5	3.0	87.67
915.5339	C54 H76 F5 O4 S	915.5379	-4.0	-4.4	15.0	85.24

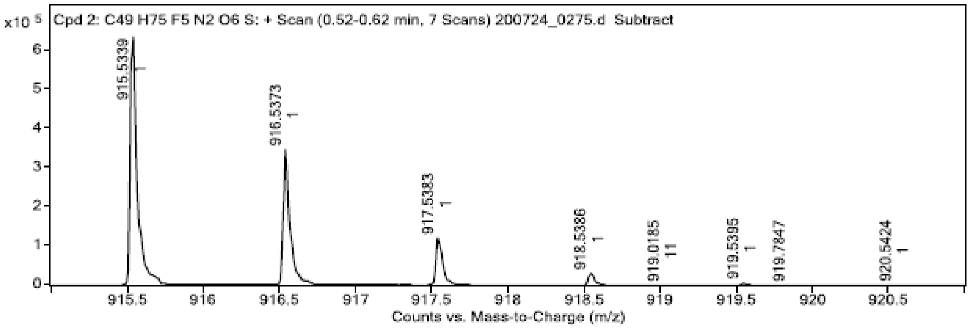

Predicted Isotope Match Table

Isotope	m/z	Calc m/z	Diff (mDa)	Abund (%)	Calc Abund (%)	+/-
1	915.5339	915.5339	0.0	100.0	100.0	0.0
2	916.5373	916.5371	0.2	55.0	55.6	0.6
3	917.5383	917.5380	0.3	19.3	20.9	1.6
4	918.5386	918.5389	-0.3	4.9	5.8	0.9
5	919.5395	919.5404	-0.9	1.0	1.3	0.3
6	920.5424	920.5422	0.2	0.2	0.2	0.0

6i

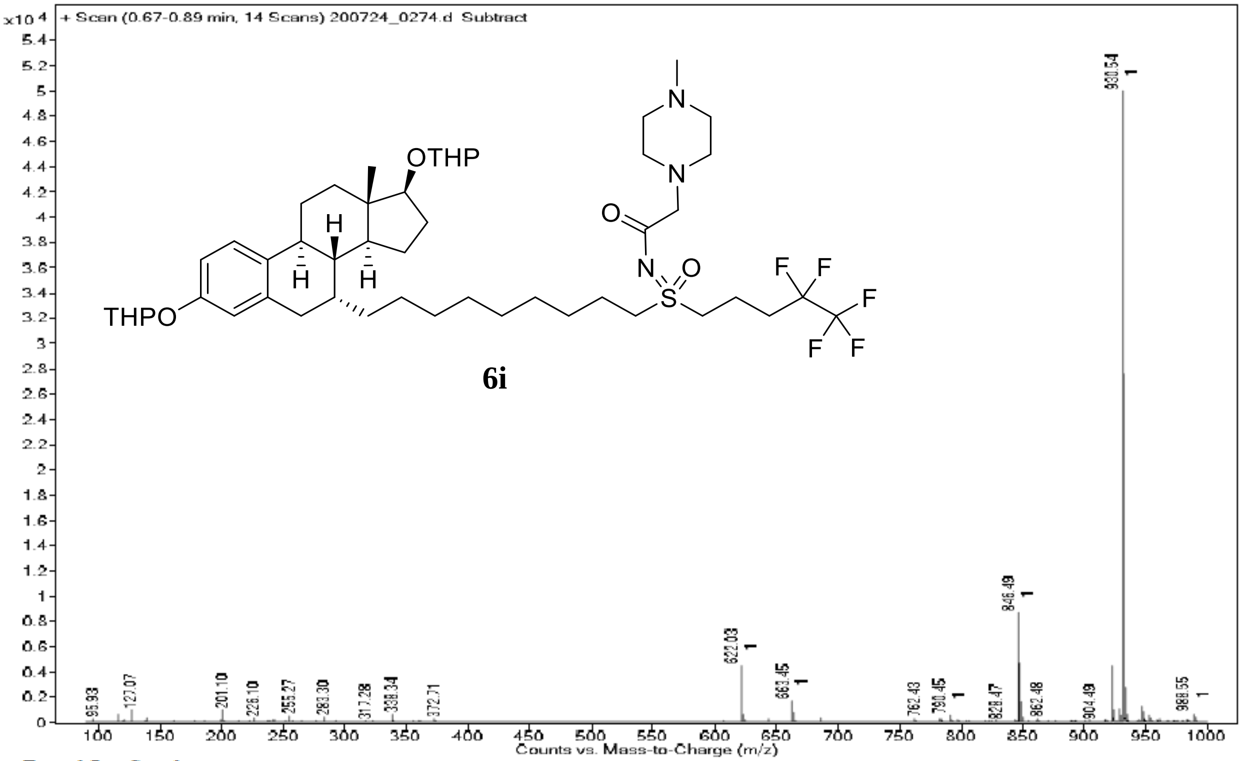

Target Ion Species

Ion Species	m/z	Ionic Formula
(M+H)+	930.5455	C49 H77 F5 N3 O6 S

MFG Calculator Results

Target m/z	Ionic Formula	Calc m/z	+/- (mDa)	+/- (ppm)	DBE	MFG Score
930.5455	C49 H77 F5 N3 O6 S	930.5448	0.7	0.8	11.0	99.04
930.5455	C50 H73 F5 N7 O2 S	930.5461	-0.6	-0.6	16.0	98.73
930.5455	C41 H73 F5 N9 O9	930.5446	0.9	1.0	8.0	95.15
930.5455	C57 H73 F5 N O4	930.5454	0.1	0.1	20.0	93.55
930.5455	C38 H77 F5 N9 O9 S	930.5480	-2.5	-2.7	3.0	92.30
930.5455	C53 H69 F5 N7 O2	930.5427	2.8	3.0	21.0	89.30
930.5455	C58 H69 F5 N5	930.5468	-1.3	-1.4	25.0	89.25
930.5455	C45 H73 F5 N9 O4 S	930.5421	3.4	3.7	12.0	89.14
930.5455	C54 H77 F5 N O4 S	930.5488	-3.3	-3.5	15.0	88.61
930.5455	C46 H73 F5 N7 O7	930.5486	-3.1	-3.3	12.0	87.56

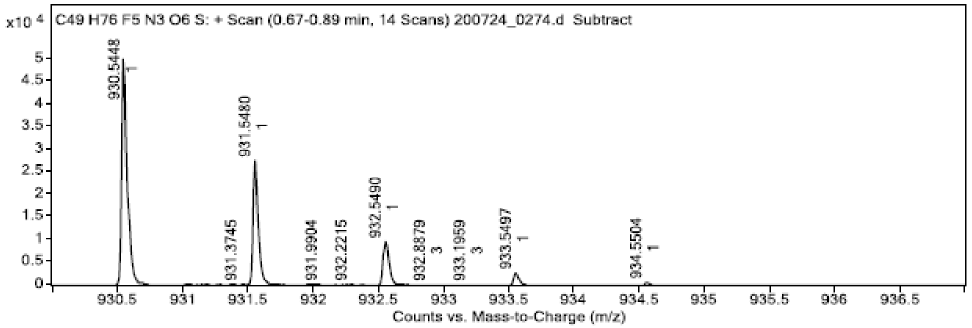

Predicted Isotope Match Table

Isotope	m/z	Calc m/z	Diff (mDa)	Abund (%)	Calc Abund (%)	+/-
1	930.5455	930.5448	0.7	100.0	100.0	0.0
2	931.5488	931.5480	0.8	53.9	56.0	2.1
3	932.5498	932.5488	1.0	19.1	21.1	2.0
4	933.5505	933.5498	0.7	5.4	5.9	0.5
5	934.5511	934.5512	-0.1	1.2	1.3	0.1

6k

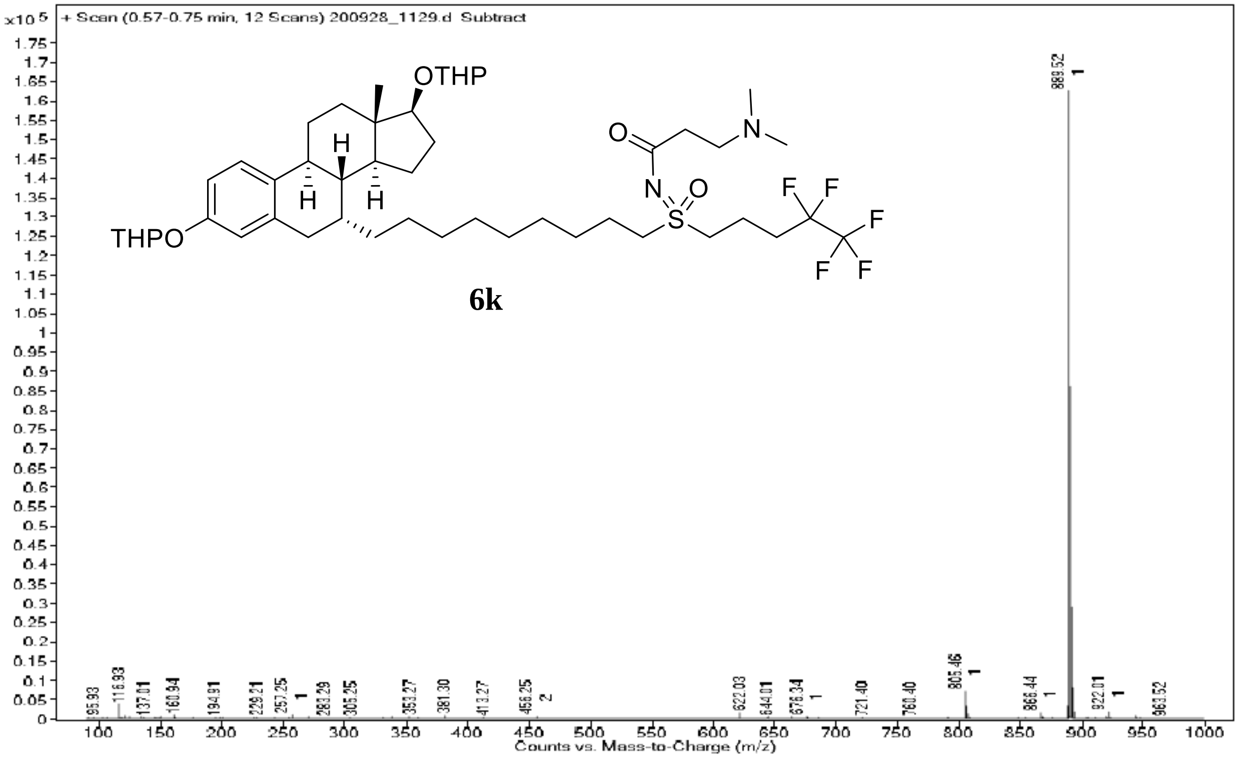

Target Ion Species

Ion Species	m/z	Ionic Formula
(M+H)+	889.5191	C47 H74 F5 N2 O6 S

MFG Calculator Results

Target m/z	Ionic Formula	Calc m/z	+/- (mDa)	+/- (ppm)	DBE	MFG Score
889.5191	C47 H74 F5 N2 O6 S	889.5182	0.9	1.0	10.0	99.05
889.5191	C46 H68 F5 N9 O S	889.5182	0.9	1.0	15.5	98.83
889.5191	C48 H70 F5 N6 O2 S	889.5196	-0.5	-0.6	15.0	98.62
889.5191	C45 H72 F5 N5 O5 S	889.5169	2.2	2.5	10.5	95.75
889.5191	C41 H72 F5 N5 O10	889.5194	-0.3	-0.3	6.5	95.16
889.5191	C39 H70 F5 N8 O9	889.5180	1.1	1.2	7.0	94.64
889.5191	C50 H72 F5 N3 O3 S	889.5209	-1.8	-2.0	14.5	94.44
889.5191	C53 H68 F5 N3 O3	889.5175	1.6	1.8	19.5	92.68
889.5191	C55 H70 F5 O4	889.5189	0.2	0.2	19.0	92.64
889.5191	C42 H68 F5 N9 O6	889.5207	-1.6	-1.8	11.5	92.07

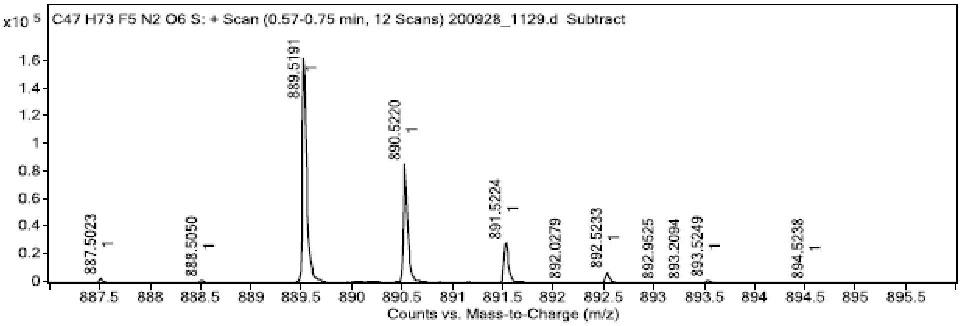

Predicted Isotope Match Table

Isotope	m/z	Calc m/z	Diff (mDa)	Abund (%)	Calc Abund (%)	+/-
1	889.5191	889.5182	0.9	100.0	100.0	0.0
2	890.5220	890.5215	0.5	51.8	53.4	1.6
3	891.5224	891.5222	0.2	17.7	19.7	2.0
4	892.5233	892.5231	0.2	4.7	5.4	0.7
5	893.5249	893.5245	0.4	1.0	1.2	0.2

7a

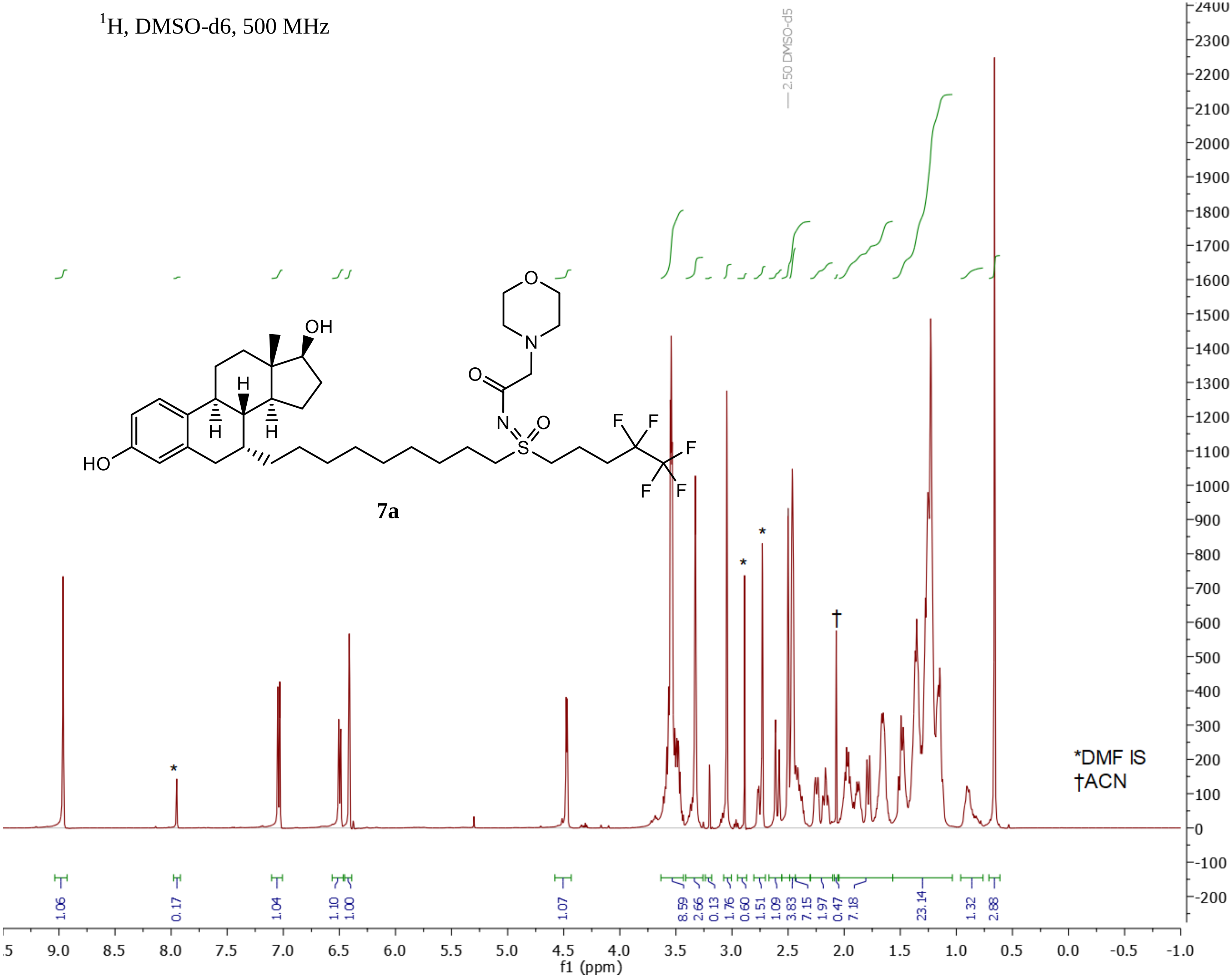

1H, DMSO-d6, 500 MHz
2.50 DMSO-d5
OH
HO
H
O
N
O
N
S
O
F
F
F
F
F
7a
*
†
*DMF IS
†ACN
1.06
0.17
1.04
1.10
1.00
1.07
8.59
2.66
0.13
1.76
0.60
1.51
1.09
3.83
7.15
1.97
0.47
7.18
23.14
1.32
2.88
f1 (ppm)

^{13}C, DMSO-d6, 126 MHz

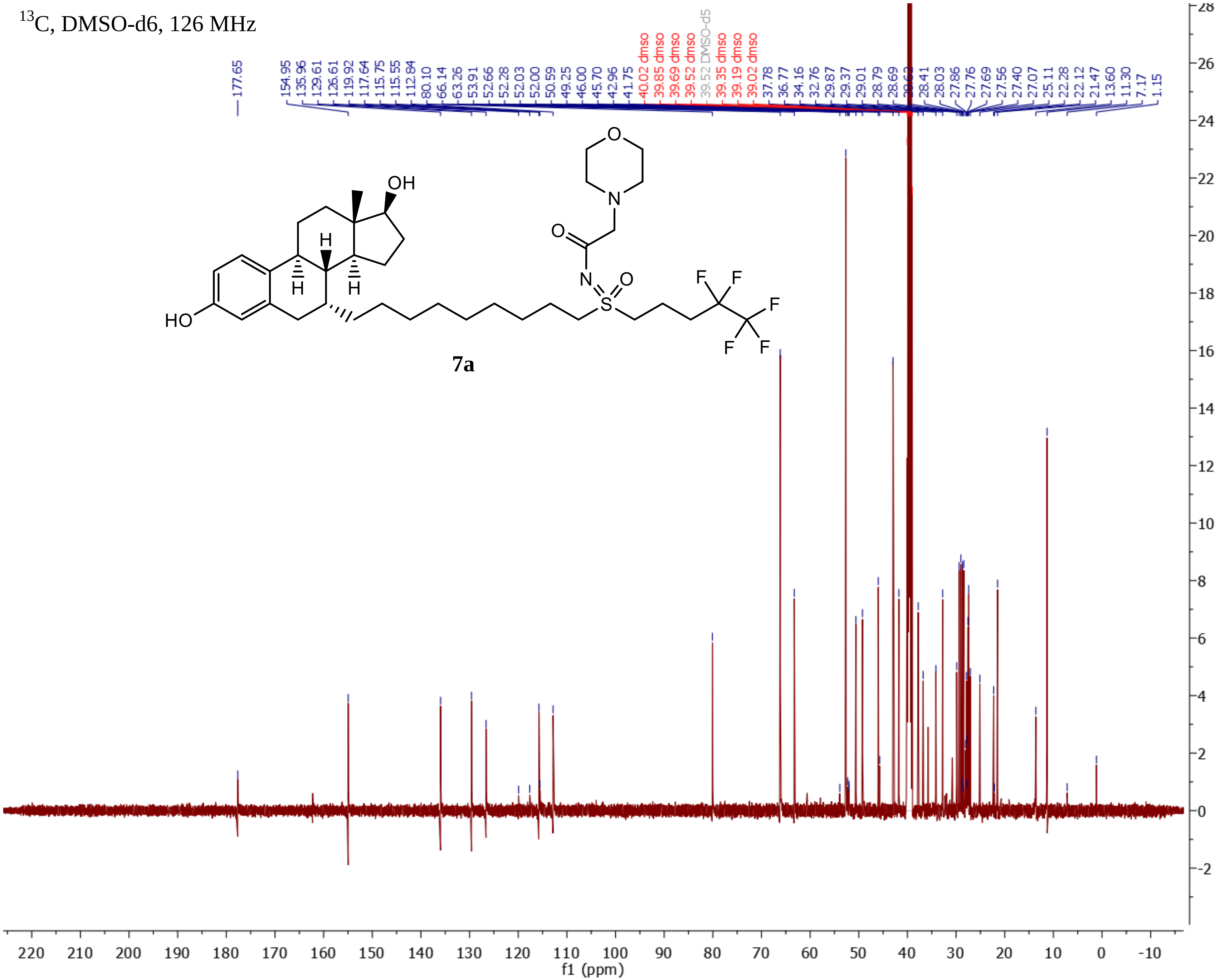

7a

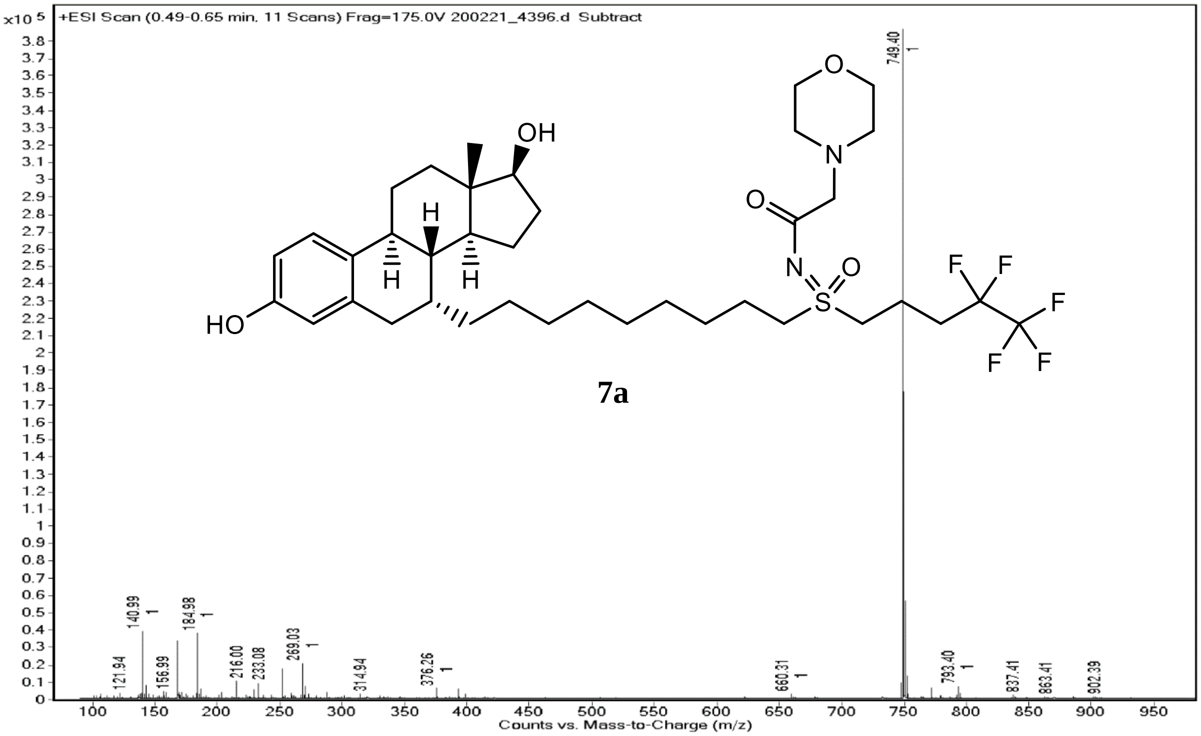

Target Ion Species

Ion Species	m/z	Ionic Formula
(M+H)+	749.3978	C38 H58 F5 N2 O5 S

MFG Calculator Results

Target m/z	Ionic Formula	Calc m/z	+/- (mDa)	+/- (ppm)	DBE	MFG Score
749.3978	C38 H58 F5 N2 O5 S	749.3981	-0.3	-0.4	9.0	99.58
749.3978	C39 H54 F5 N6 O S	749.3994	-1.6	-2.1	14.0	96.77
749.3978	C34 H54 F5 N8 O3 S	749.3954	2.4	3.2	10.0	91.67
749.3978	C42 H50 F5 N6 O	749.3961	1.7	2.3	19.0	91.58
749.3978	C30 H54 F5 N8 O8	749.3979	-0.1	-0.1	6.0	90.84
749.3978	C46 H54 F5 O3	749.3988	-1.0	-1.3	18.0	89.86
749.3978	C41 H54 F5 N2 O5	749.3947	3.1	4.1	14.0	87.58
749.3978	C29 H58 F5 N4 O12	749.3966	1.2	1.6	1.0	87.52
749.3978	C33 H58 F5 N4 O7 S	749.3941	3.7	4.9	5.0	82.27
749.3978	C34 H58 F5 N2 O10	749.4006	-2.8	-3.7	5.0	82.03

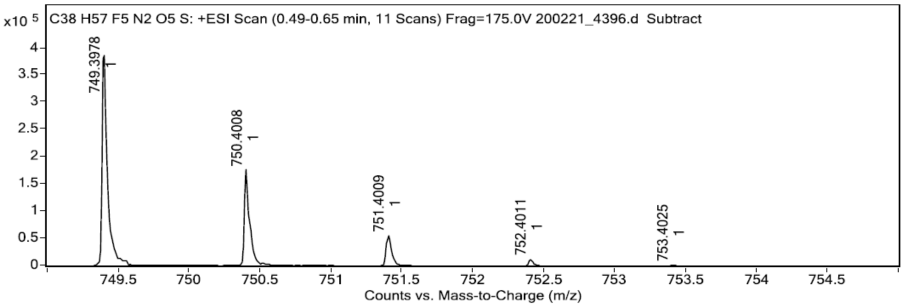

Predicted Isotope Match Table

Isotope	m/z	Calc m/z	Diff (mDa)	Abund (%)	Calc Abund (%)	+/-
1	749.3978	749.3981	-0.3	100.0	100.0	0.0
2	750.4008	750.4013	-0.5	44.5	43.5	-1.0
3	751.4009	751.4012	-0.3	14.2	14.7	0.5
4	752.4011	752.4019	-0.8	3.4	3.6	0.2
5	753.4025	753.4034	-0.9	0.6	0.7	0.1

7b

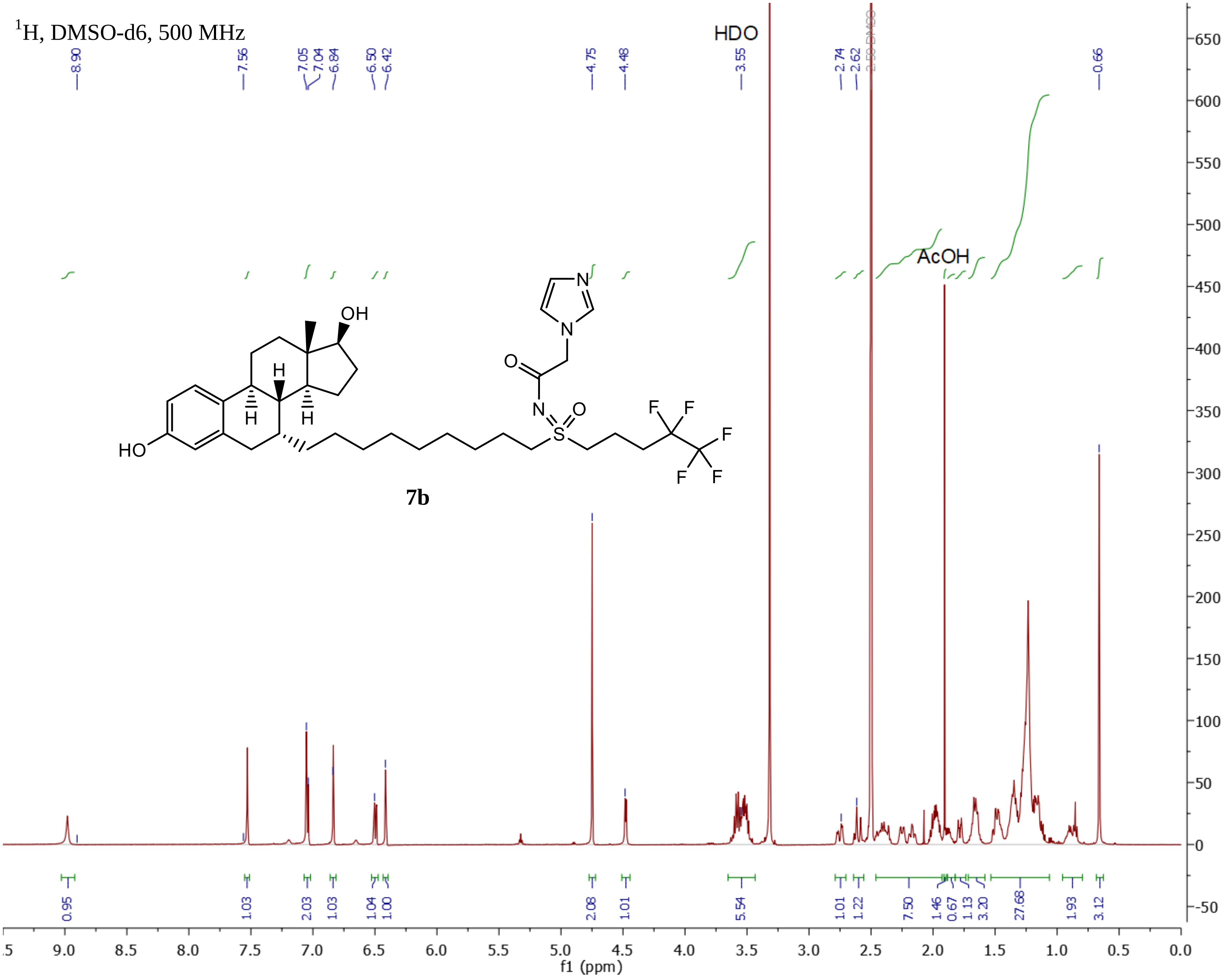

¹H, DMSO-d6, 500 MHz
8.90
7.56
7.05
7.04
6.84
6.50
6.42
4.75
4.48
3.55
2.74
2.62
2.50 DMSO
0.66
HDO
AcOH
7b
0.95
1.03
2.03
1.03
1.04
1.00
2.08
1.01
5.54
1.01
1.22
7.50
1.46
0.67
1.13
3.20
27.68
1.93
3.12
f1 (ppm)

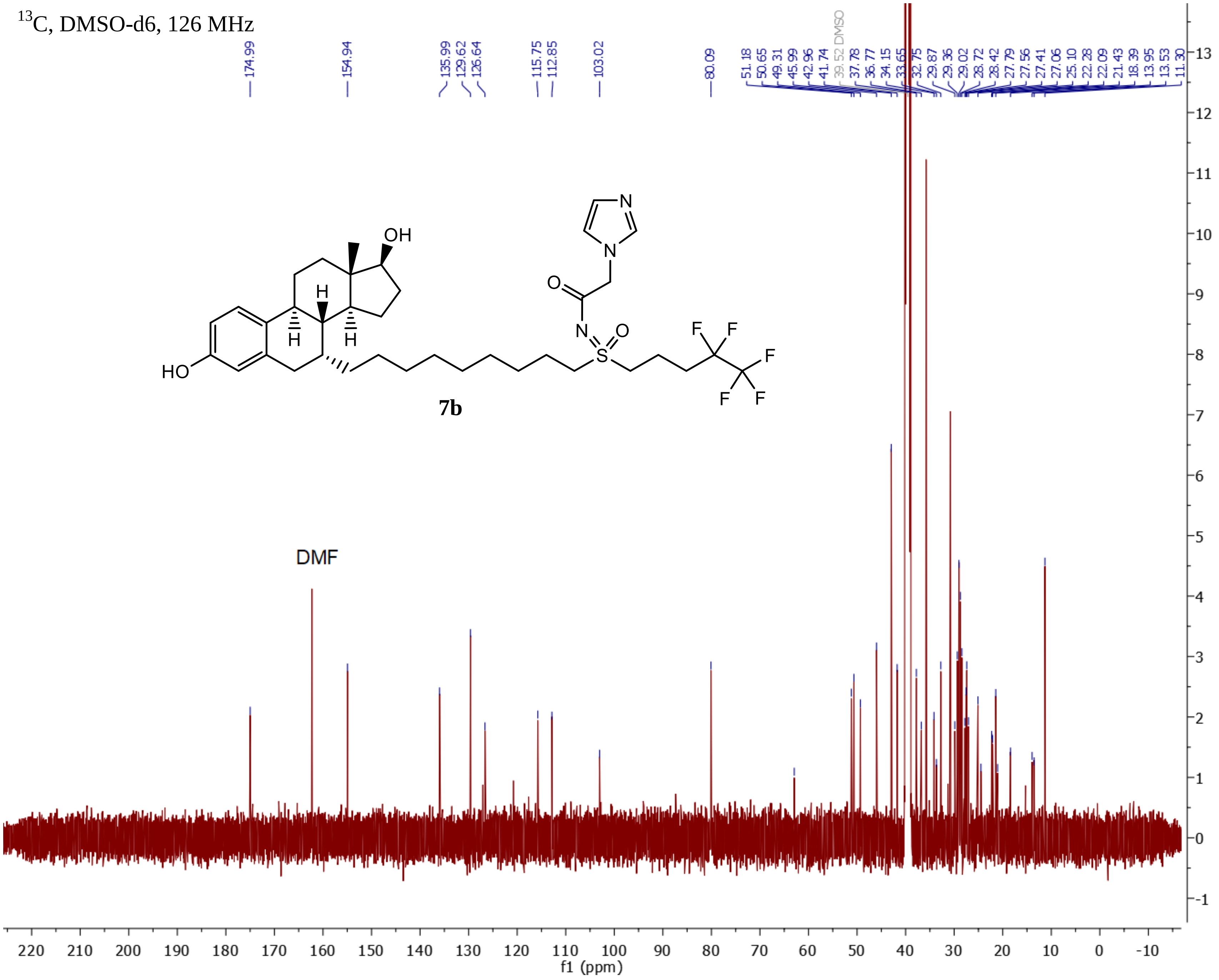

13C, DMSO-d6, 126 MHz
174.99
154.94
135.99
129.62
126.64
115.75
112.85
103.02
80.09
51.18
50.65
49.31
45.99
42.96
41.74
39.52 DMSO
37.78
36.77
34.15
33.65
32.75
29.87
29.36
29.02
28.72
28.42
27.79
27.56
27.41
27.06
25.10
22.28
22.09
21.43
18.39
13.95
13.53
11.30
OH
HO
H
N
O
S
F
7b
DMF
f1 (ppm)

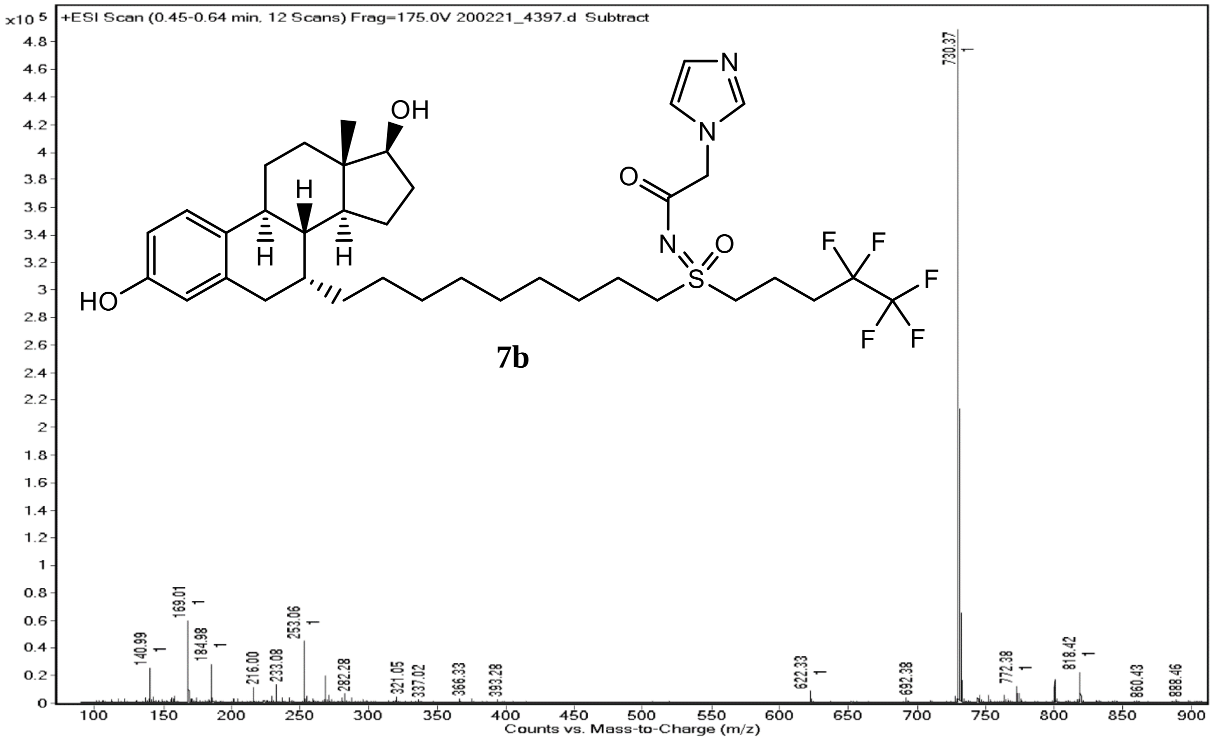

Target Ion Species

Ion Species	m/z	Ionic Formula
(M+H)+	730.3666	C37 H53 F5 N3 O4 S

MFG Calculator Results

Target m/z	Ionic Formula	Calc m/z	+/- (mDa)	+/- (ppm)	DBE	MFG Score
730.3666	C37 H53 F5 N3 O4 S	730.3671	-0.5	-0.7	11.0	99.28
730.3666	C38 H49 F5 N7 S	730.3685	-1.9	-2.6	16.0	95.24
730.3666	C29 H49 F5 N9 O7	730.3670	-0.4	-0.5	8.0	93.19
730.3666	C33 H49 F5 N9 O2 S	730.3645	2.1	2.9	12.0	92.19
730.3666	C41 H45 F5 N7	730.3651	1.5	2.1	21.0	91.07
730.3666	C28 H53 F5 N5 O11	730.3656	1.0	1.4	3.0	90.47
730.3666	C45 H49 F5 N O2	730.3678	-1.2	-1.6	20.0	88.27
730.3666	C40 H49 F5 N3 O4	730.3638	2.8	3.8	16.0	87.80
730.3666	C32 H53 F5 N5 O6 S	730.3631	3.5	4.8	7.0	83.44
730.3666	C33 H53 F5 N3 O9	730.3696	-3.0	-4.1	7.0	83.42

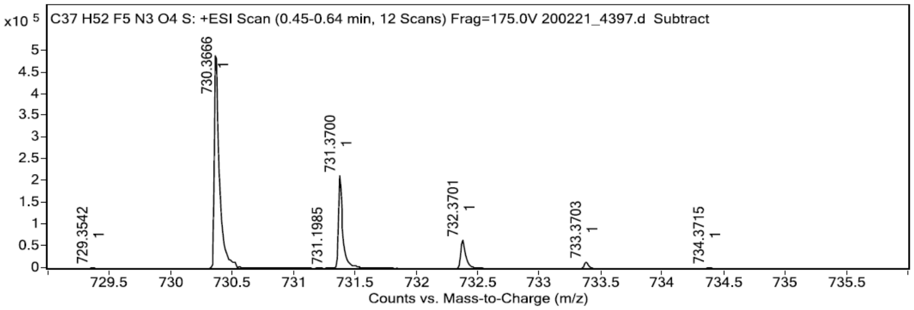

Predicted Isotope Match Table

Isotope	m/z	Calc m/z	Diff (mDa)	Abund (%)	Calc Abund (%)	+/-
1	730.3666	730.3671	-0.5	100.0	100.0	0.0
2	731.3700	731.3703	-0.3	41.8	42.7	0.9
3	732.3701	732.3700	0.1	12.7	14.2	1.5
4	733.3703	733.3707	-0.4	3.0	3.4	0.4
5	734.3715	734.3721	-0.6	0.6	0.6	0.0
6	735.3725	735.3739	-1.4	0.1	0.1	0.0

7c

^{1}H, DMSO-d6, 500 MHz

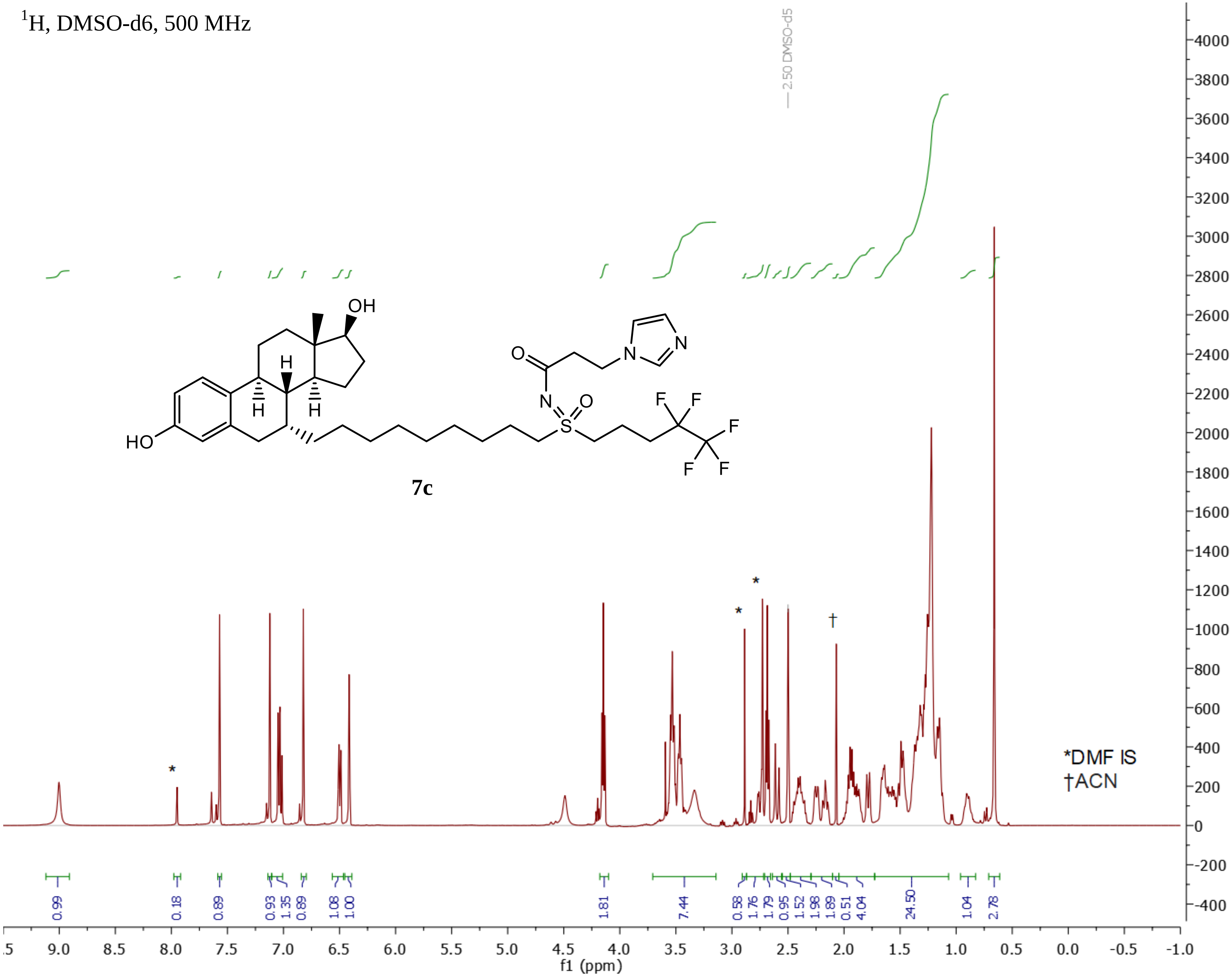

^{13}C, DMSO-d6, 126 MHz

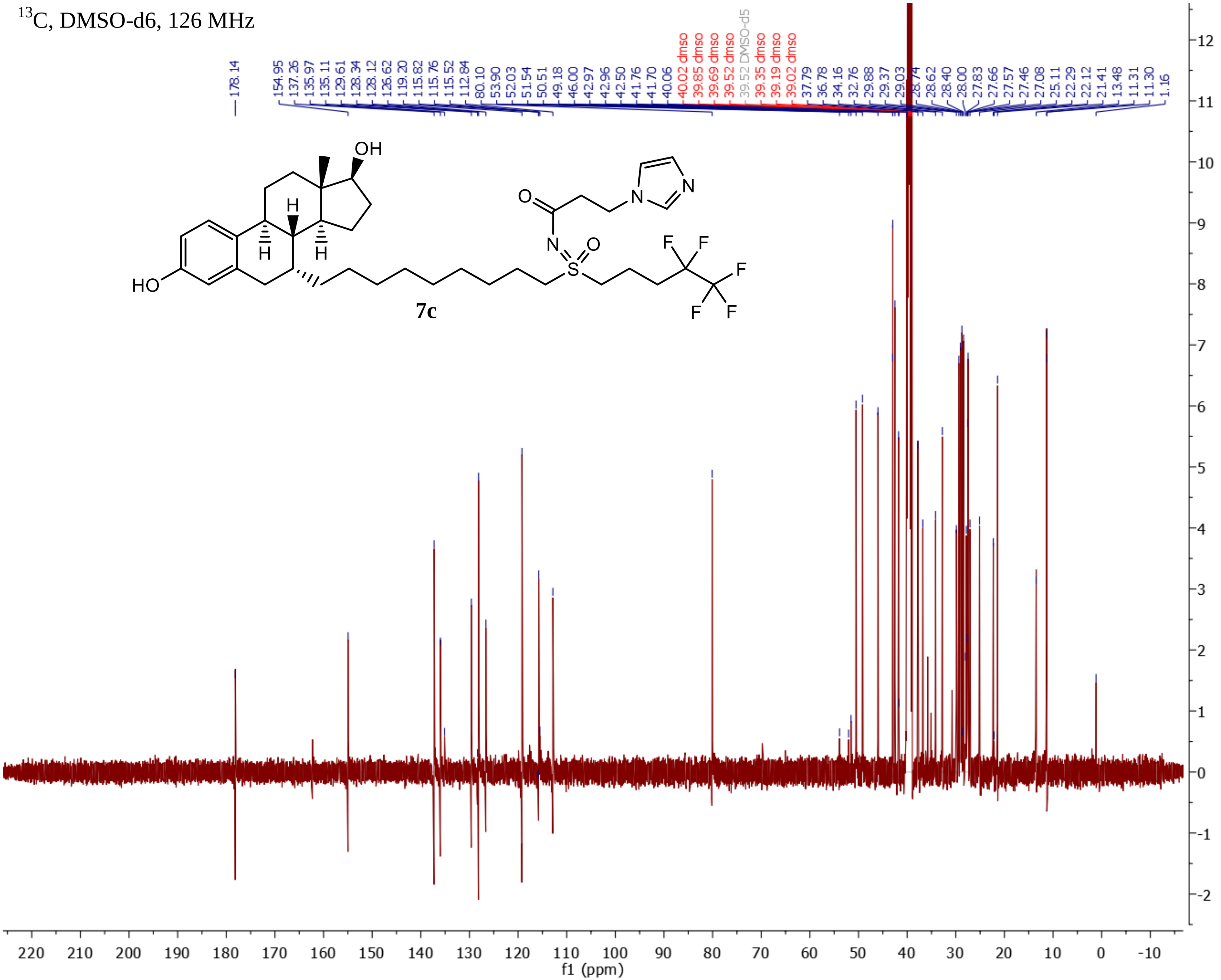

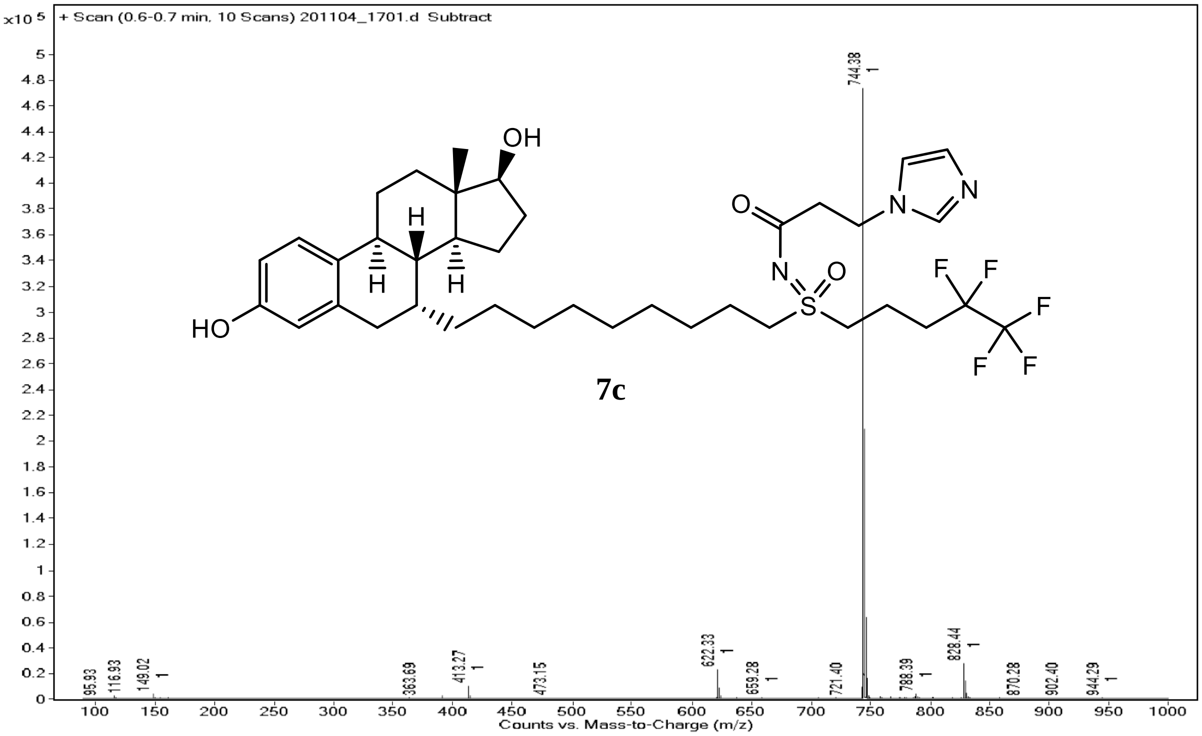

Target Ion Species

Ion Species	m/z	Ionic Formula
(M+H)+	744.3826	C38 H55 F5 N3 O4 S

MFG Calculator Results

Target m/z	Ionic Formula	Calc m/z	+/- (mDa)	+/- (ppm)	DBE	MFG Score
744.3826	C38 H55 F5 N3 O4 S	744.3828	-0.2	-0.3	11.0	99.53
744.3826	C36 H52 F2 N9 O4 S	744.3826	0.0	0.0	15.0	99.47
744.3826	C33 H53 F3 N9 O5 S	744.3837	-1.1	-1.5	11.0	97.94
744.3826	C41 H54 F4 N3 O3 S	744.3817	0.9	1.2	15.0	97.34
744.3826	C32 H57 F3 N5 O9 S	744.3824	0.2	0.3	6.0	97.06
744.3826	C35 H56 F2 N5 O8 S	744.3812	1.4	1.9	10.0	96.47
744.3826	C39 H51 F5 N7 S	744.3841	-1.5	-2.0	16.0	96.45
744.3826	C38 H50 N9 O7	744.3828	-0.2	-0.3	19.0	96.38
744.3826	C39 H51 F N9 O3 S	744.3814	1.2	1.6	19.0	96.36
744.3826	C40 H53 F3 N3 O7	744.3830	-0.4	-0.5	15.0	95.18

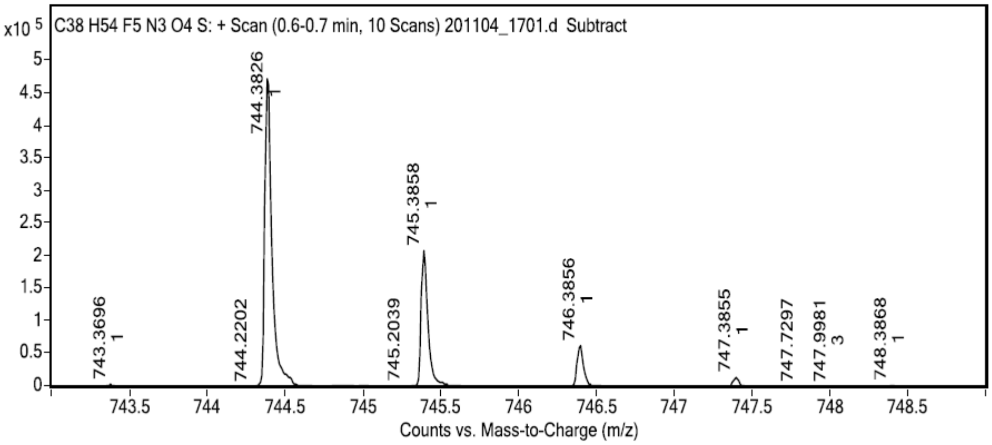

Predicted Isotope Match Table

Isotope	m/z	Calc m/z	Diff (mDa)	Abund (%)	Calc Abund (%)	+/-
1	744.3826	744.3828	-0.2	100.0	100.0	0.0
2	745.3858	745.3860	-0.2	42.9	43.8	0.9
3	746.3856	746.3858	-0.2	13.0	14.6	1.6
4	747.3855	747.3865	-1.0	3.1	3.6	0.5
5	748.3868	748.3879	-1.1	0.5	0.7	0.2
6	749.3862	749.3897	-3.5	0.1	0.1	0.0

7d

^{1}H, DMSO-d6, 500 MHz

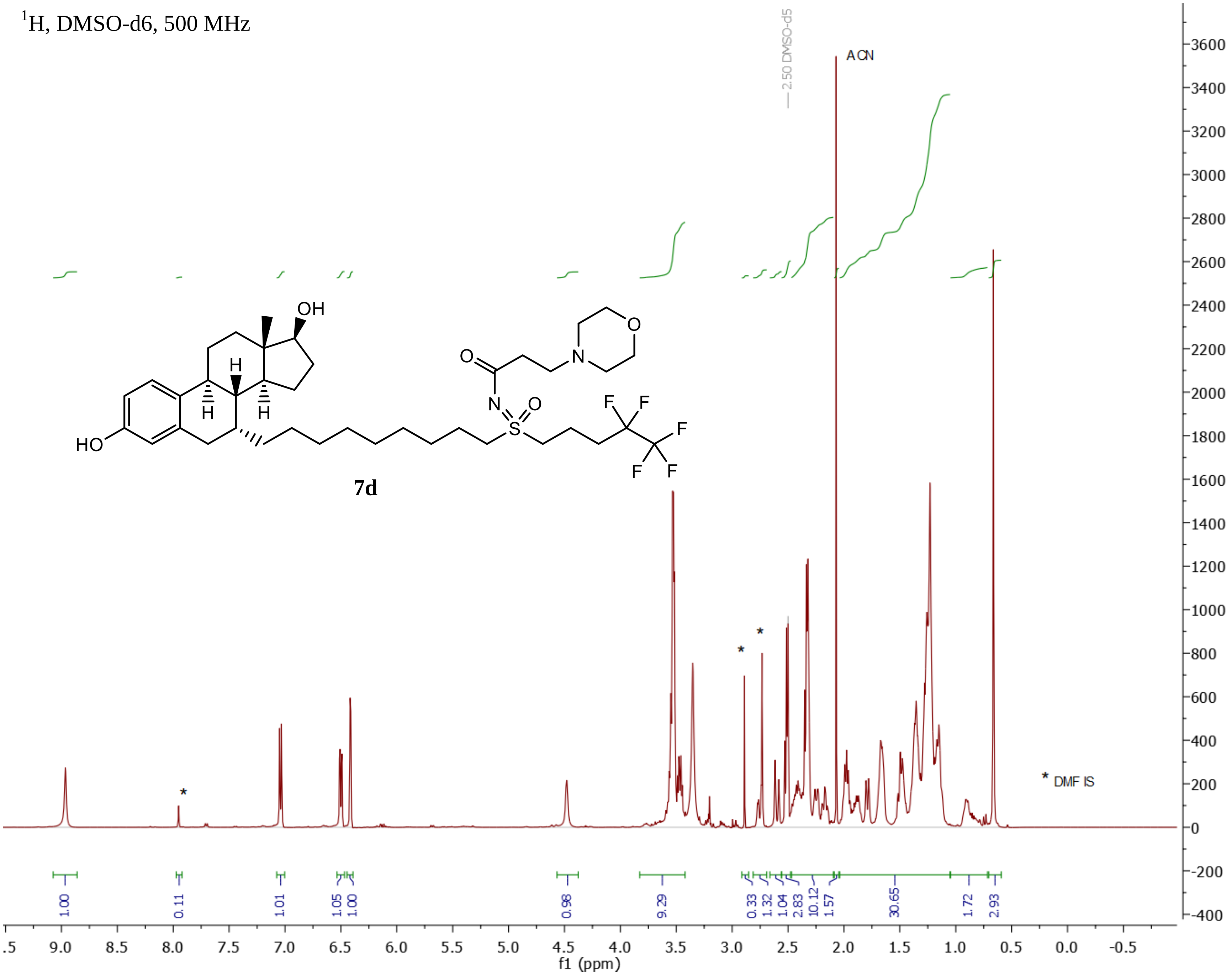

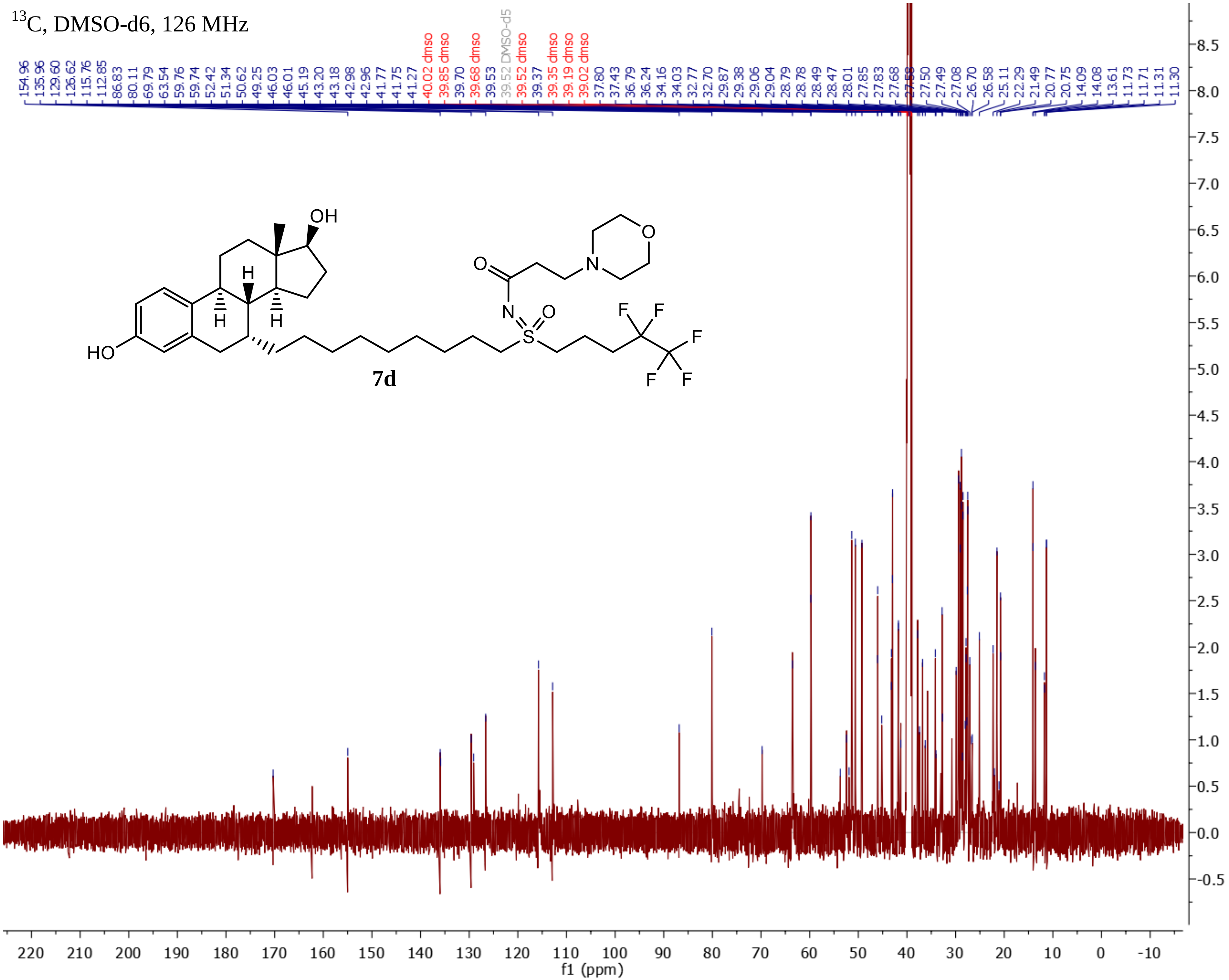
13C, DMSO-d6, 126 MHz
154.96
135.96
129.60
126.62
115.76
112.85
86.83
80.11
69.79
63.54
59.76
59.74
52.42
51.34
50.62
49.25
46.03
46.01
45.19
43.20
43.18
42.98
42.96
41.77
41.75
41.27
40.02 dmso
39.85 dmso
39.70
39.68 dmso
39.53
39.52 DMSO-d5
39.52 dmso
39.37
39.35 dmso
39.19 dmso
39.02 dmso
37.80
37.43
36.79
36.24
34.16
34.03
32.77
32.70
29.87
29.38
29.06
29.04
28.79
28.78
28.49
28.47
28.01
27.85
27.83
27.68
27.50
27.49
27.08
26.70
26.58
25.11
22.29
21.49
20.77
20.75
14.09
14.08
13.61
11.73
11.71
11.31
11.30
OH
H
H
H
HO
O
N
O
N
S
O
F
F
F
F
F
7d
f1 (ppm)

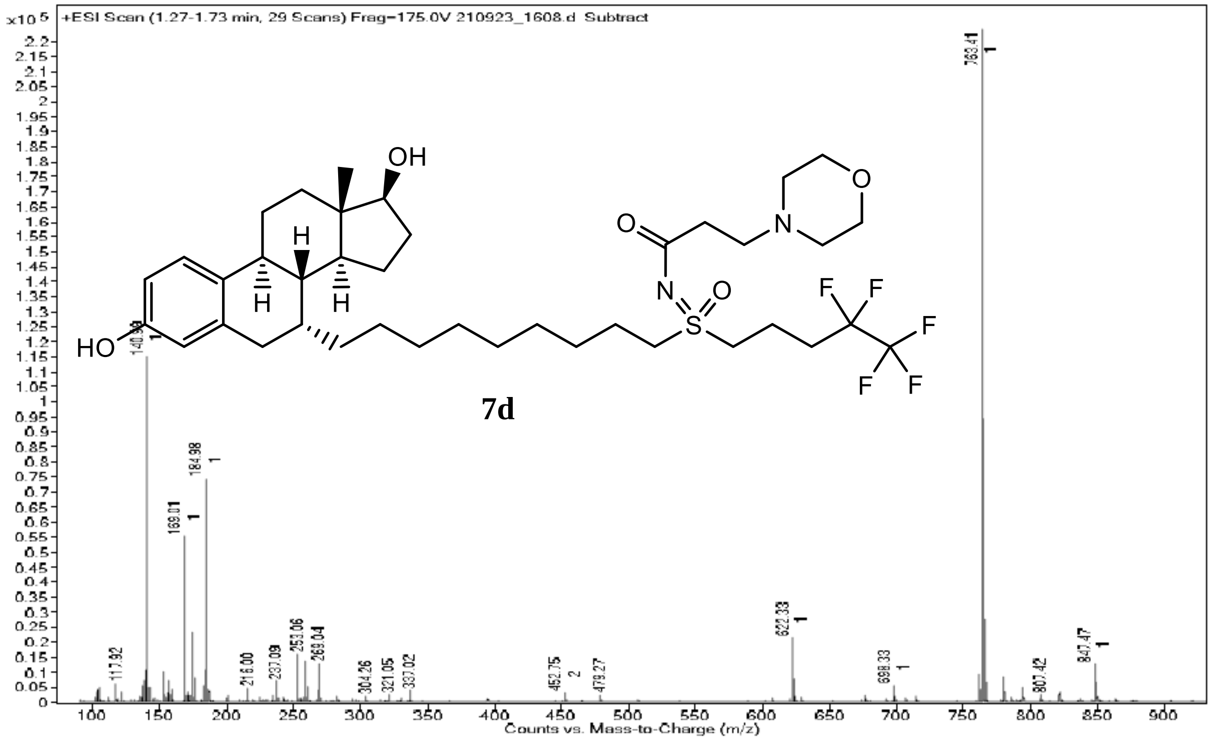

Target Ion Species

Ion Species	m/z	Ionic Formula
(M+H)+	763.4131	C39 H60 F5 N2 O5 S

MFG Calculator Results

Target m/z	Ionic Formula	Calc m/z	+/- (mDa)	+/- (ppm)	DBE	MFG Score
763.4131	C39 H60 F5 N2 O5 S	763.4138	-0.7	-0.9	9.0	98.65
763.4131	C35 H56 F5 N8 O3 S	763.4111	2.0	2.6	10.0	94.65
763.4131	C40 H56 F5 N6 O S	763.4151	-2.0	-2.6	14.0	94.48
763.4131	C43 H52 F5 N6 O	763.4117	1.4	1.8	19.0	92.49
763.4131	C31 H56 F5 N8 O8	763.4136	-0.5	-0.7	6.0	90.72
763.4131	C42 H56 F5 N2 O5	763.4104	2.7	3.5	14.0	89.86
763.4131	C47 H56 F5 O3	763.4144	-1.3	-1.7	18.0	87.29
763.4131	C34 H60 F5 N4 O7 S	763.4097	3.4	4.5	5.0	86.20
763.4131	C35 H60 F5 N2 O10	763.4163	-3.2	-4.2	5.0	79.45
763.4131	C28 H60 F5 N8 O8 S	763.4169	-3.8	-5.0	1.0	77.19

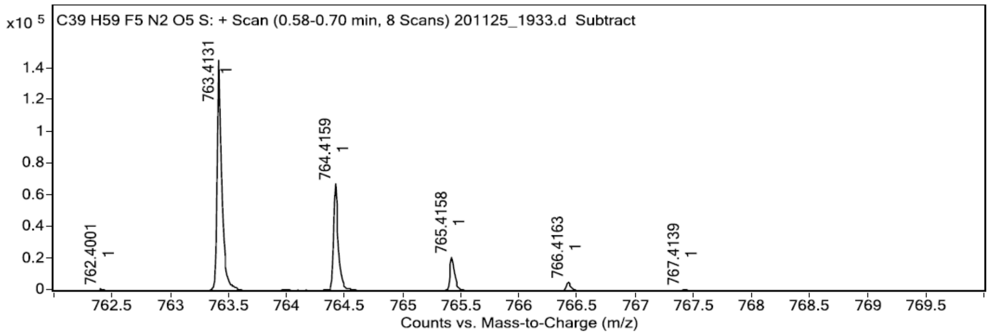

Predicted Isotope Match Table

Isotope	m/z	Calc m/z	Diff (mDa)	Abund (%)	Calc Abund (%)	+/-
1	763.4131	763.4138	-0.7	100.0	100.0	0.0
2	764.4159	764.4170	-1.1	45.1	44.6	-0.5
3	765.4158	765.4169	-1.1	14.1	15.2	1.1
4	766.4163	766.4177	-1.4	3.6	3.8	0.2
5	767.4139	767.4192	-5.3	0.7	0.7	0.0

7e

^{1}H, DMSO-d6, 500 MHz

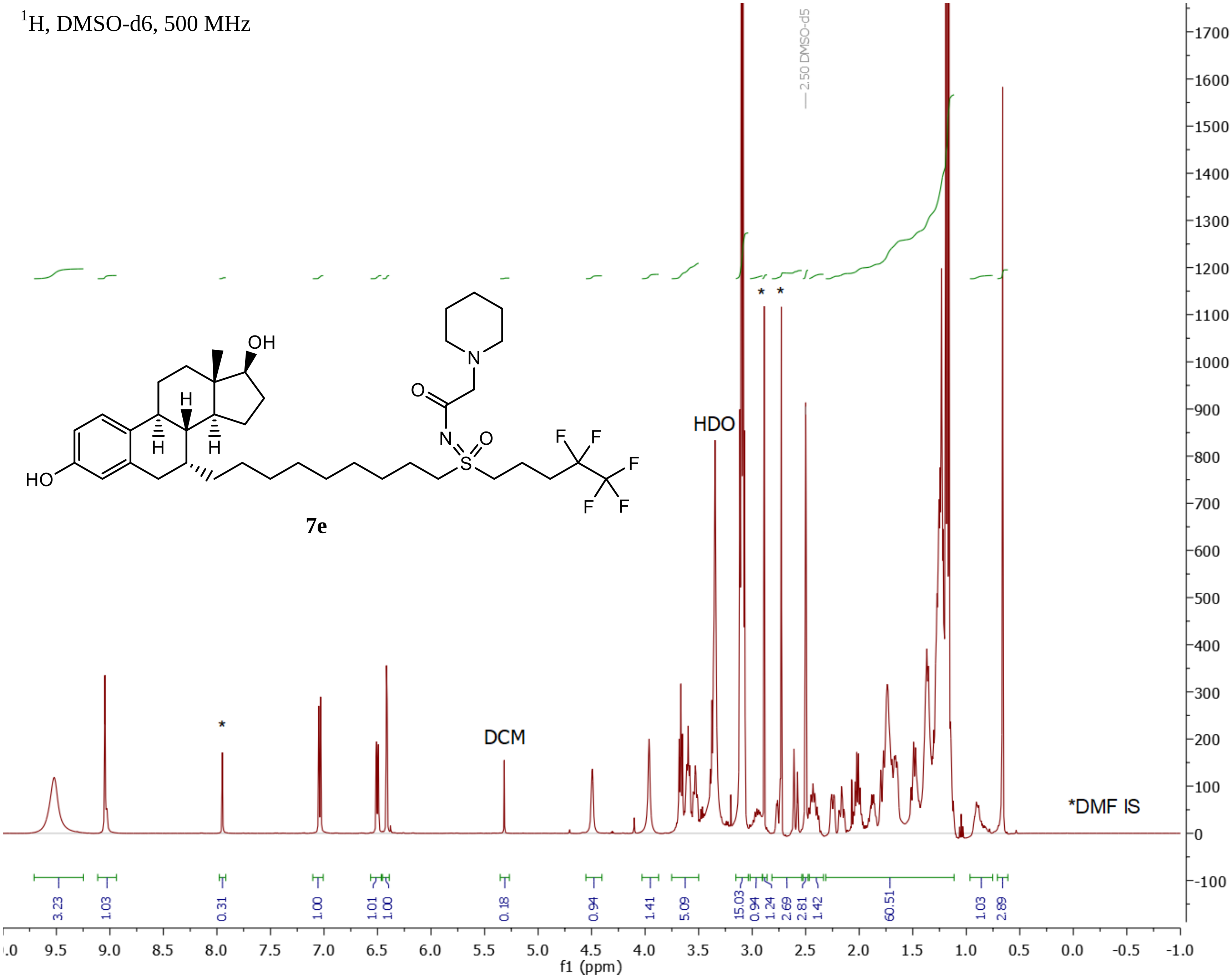

^{13}C, DMSO-d6, 126 MHz

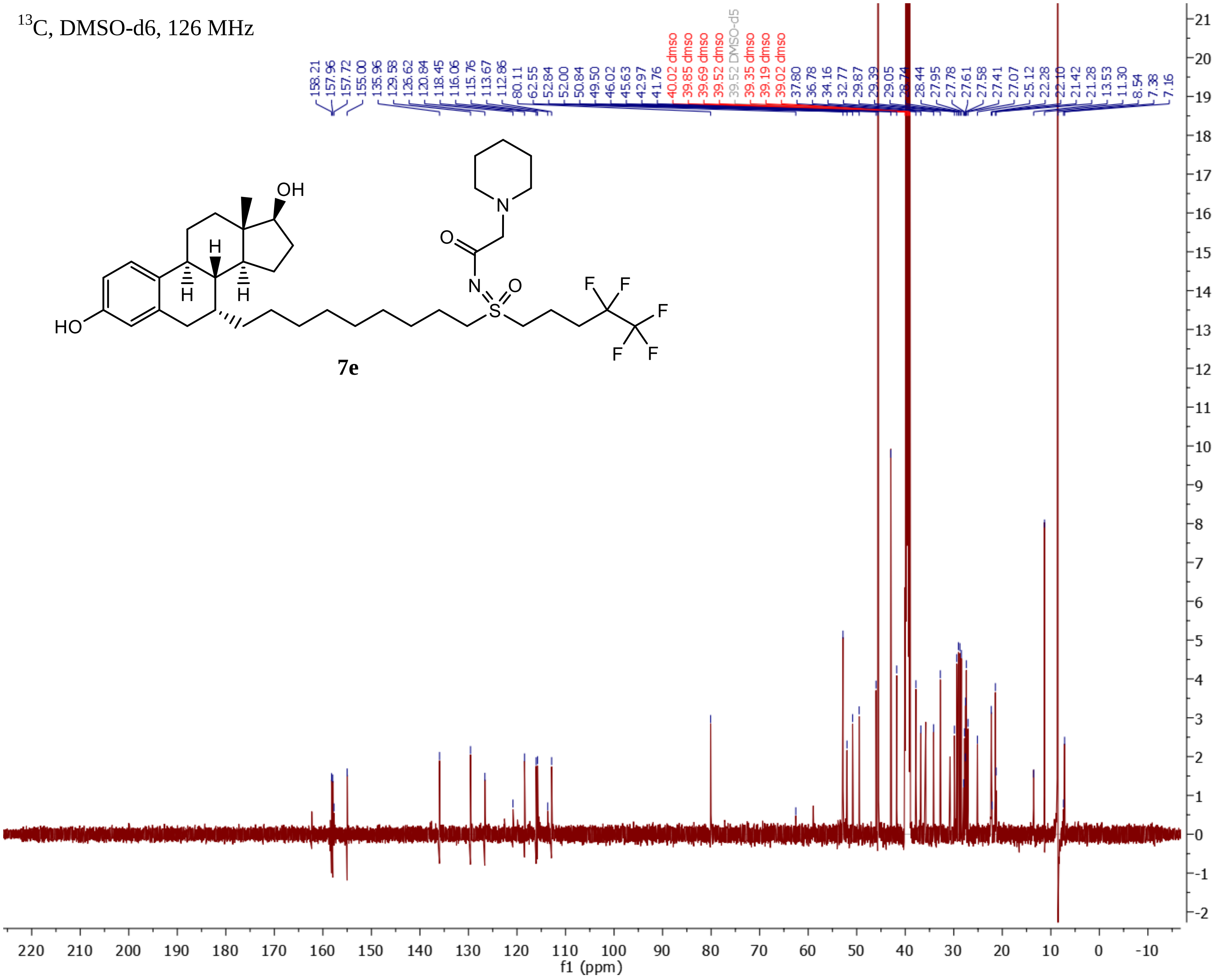

7e

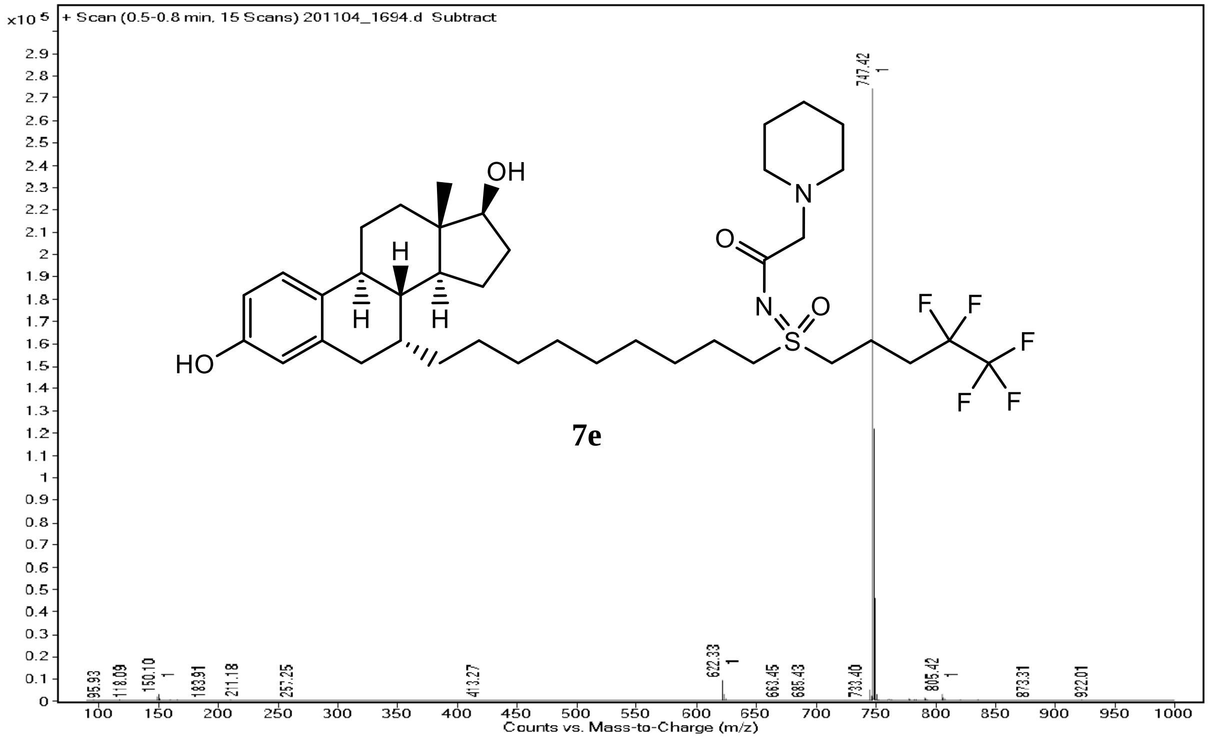

Target Ion Species

Ion Species	*m/z*	*Ionic Formula*
(M+H)+	747.4181	C39 H60 F5 N2 O4 S

MFG Calculator Results

Target m/z	*Ionic Formula*	*Calc m/z*	*+/- (mDa)*	*+/- (ppm)*	*DBE*	*MFG Score*
747.4181	C36 H61 F2 N4 O8 S	747.4173	0.8	1.1	8.0	96.79
747.4181	C31 H59 N10 O9 S	747.4182	-0.1	-0.1	8.0	96.08
747.4181	C33 H62 F3 N4 O9 S	747.4184	-0.3	-0.4	4.0	94.80
747.4181	C37 H57 F2 N8 O4 S	747.4186	-0.5	-0.7	13.0	94.76
747.4181	C40 H56 F N8 O3 S	747.4175	0.6	0.8	17.0	94.60
747.4181	C35 H56 F5 N8 O2 S	747.4162	1.9	2.5	10.0	94.27
747.4181	C39 H60 F N4 O7 S	747.4161	2.0	2.7	12.0	94.22
747.4181	C42 H59 F4 N2 O3 S	747.4177	0.4	0.5	13.0	93.75
747.4181	C39 H60 F5 N2 O4 S	747.4188	-0.7	-0.9	9.0	92.92
747.4181	C34 H58 F3 N8 O5 S	747.4197	-1.6	-2.1	9.0	90.94

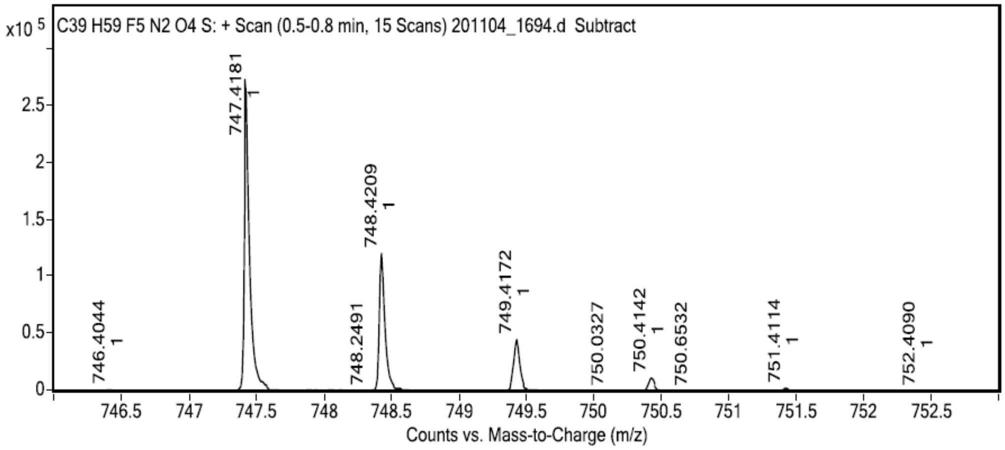

Predicted Isotope Match Table

Isotope	*m/z*	*Calc m/z*	Diff (mDa)	Abund (%)	Calc Abund (%)	+/-
1	747.4181	747.4188	-0.7	100.0	100.0	0.0
2	748.4209	748.4221	-1.2	43.0	44.5	1.5
3	749.4172	749.4220	-4.8	16.2	15.0	-1.2
4	750.4142	750.4227	-8.5	4.4	3.7	-0.7
5	751.4114	751.4241	-12.7	1.1	0.7	-0.4
6	752.4090	752.4260	-17.0	0.2	0.1	-0.1

7f

^{1}H, DMSO-d6, 500 MHz

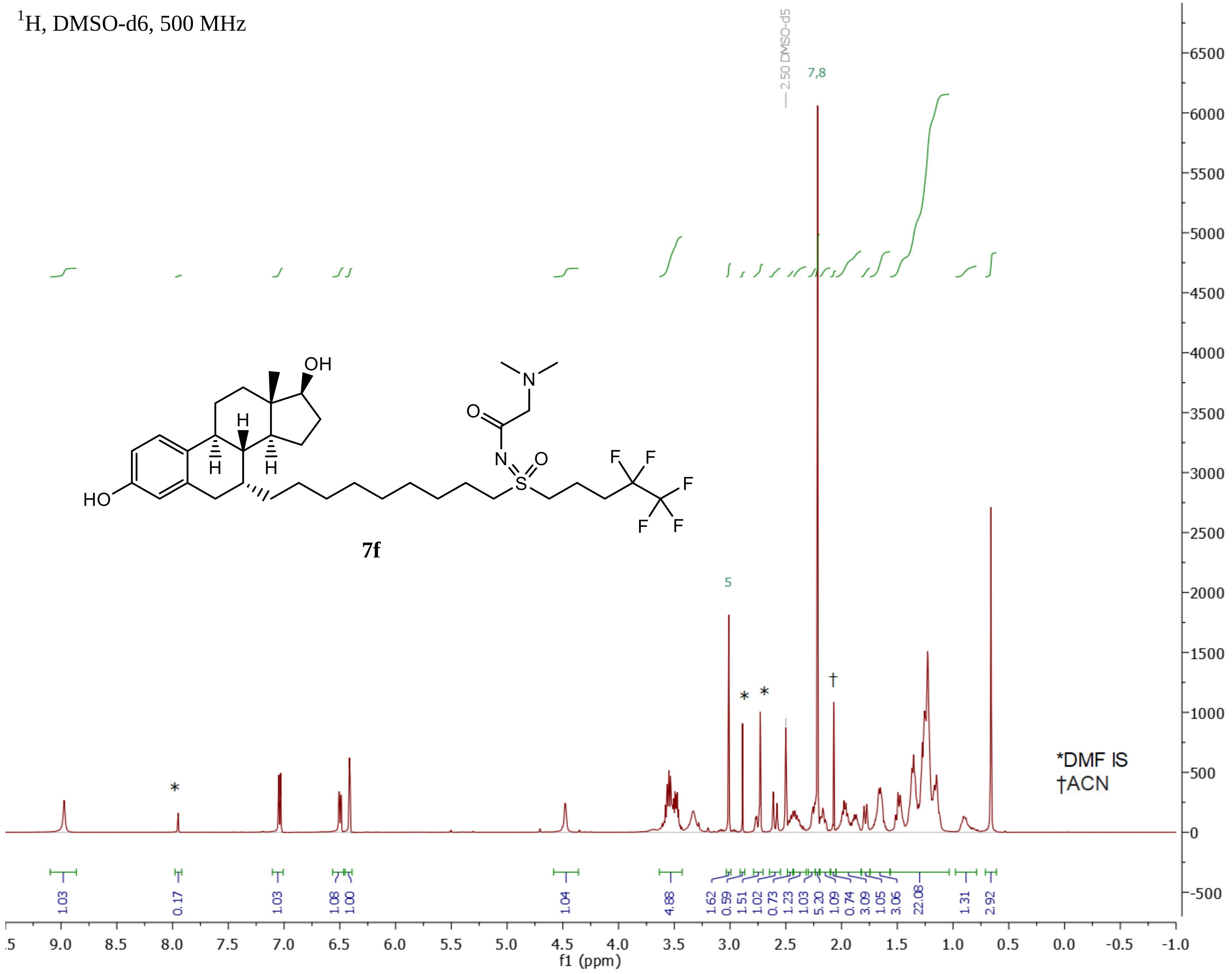

^{13}C, DMSO-d6, 126 MHz

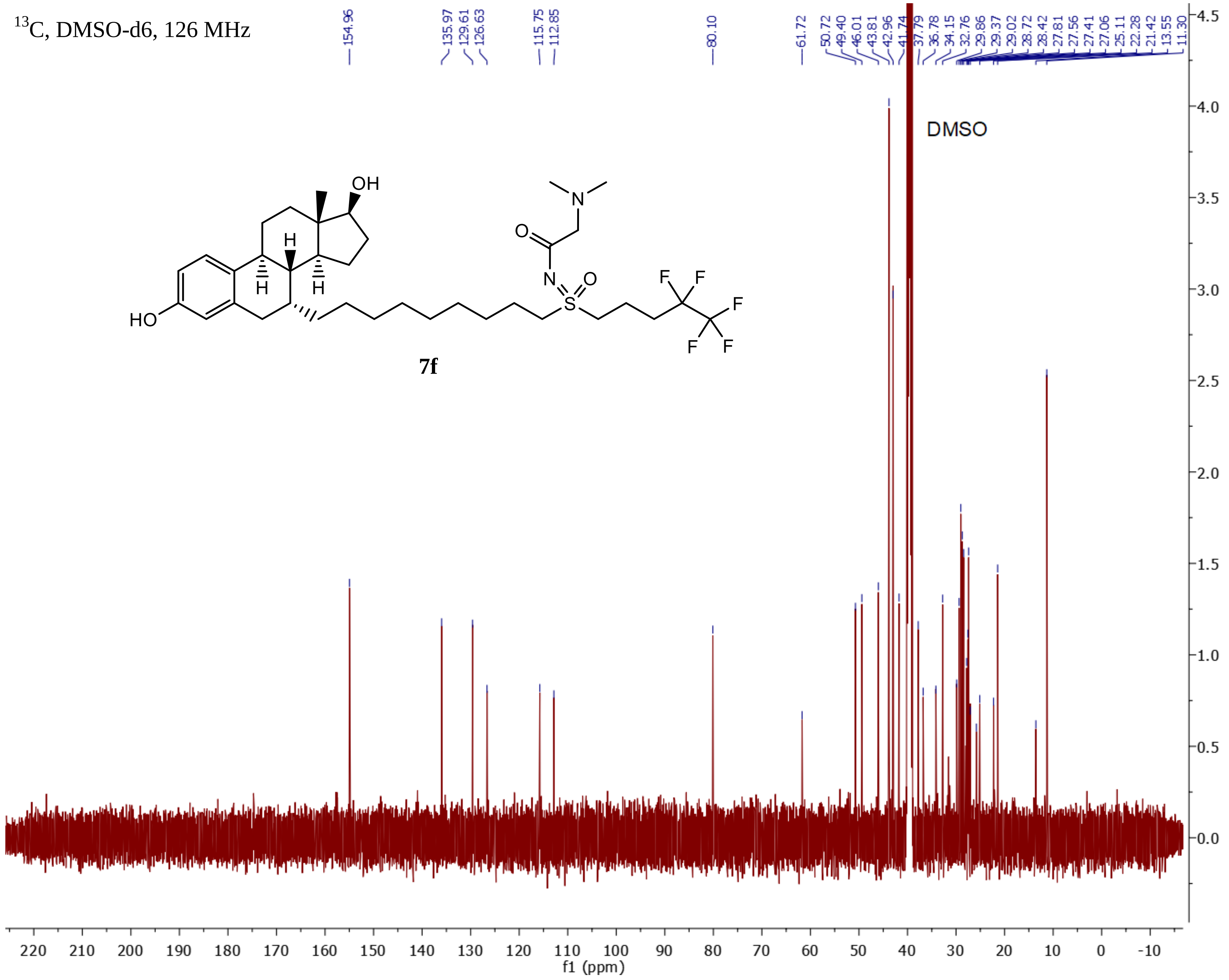

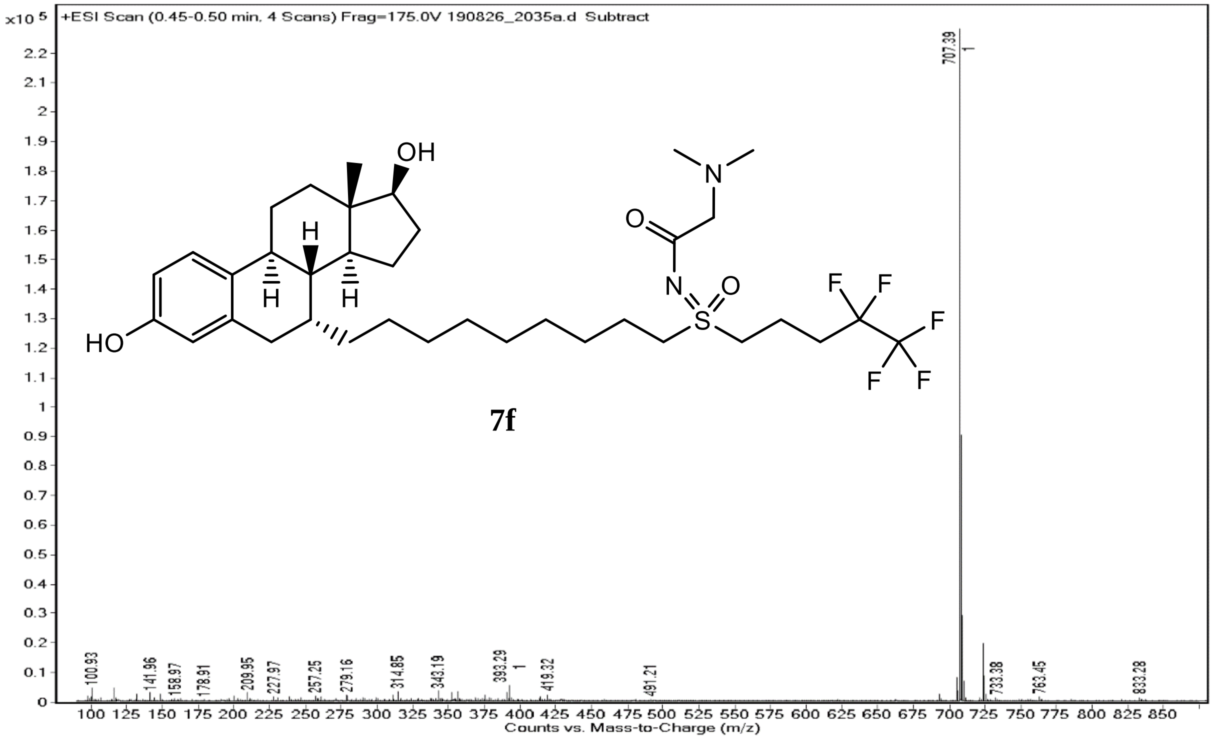

Target Ion Species

Ion Species	m/z	Ionic Formula
(M+H)+	707.3876	C36 H56 F5 N2 O4 S

MFG Calculator Results

Target m/z	Ionic Formula	Calc m/z	+/- (mDa)	+/- (ppm)	DBE	MFG Score
707.3876	C36 H56 F5 N2 O4 S	707.3875	0.1	0.1	8.0	99.89
707.3876	C37 H52 F5 N6 S	707.3889	-1.3	-1.8	13.0	97.39
707.3876	C32 H52 F5 N8 O2 S	707.3849	2.7	3.8	9.0	89.68
707.3876	C25 H56 F5 N8 O7 S	707.3907	-3.1	-4.4	0.0	80.38
707.3876	C41 H56 F5 O2 S	707.3916	-4.0	-5.7	12.0	79.99
707.3876	C31 H56 F5 N4 O6 S	707.3835	4.1	5.8	4.0	79.80

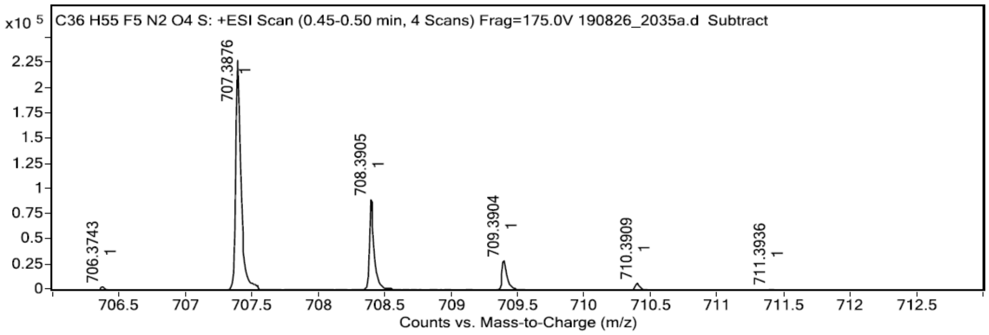

Predicted Isotope Match Table

Isotope	m/z	Calc m/z	Diff (mDa)	Abund (%)	Calc Abund (%)	+/-
1	707.3876	707.3875	0.1	100.0	100.0	0.0
2	708.3905	708.3908	-0.3	41.5	41.3	-0.2
3	709.3904	709.3903	0.1	13.1	13.6	0.5
4	710.3909	710.3910	-0.1	3.1	3.2	0.1
5	711.3936	711.3925	1.1	0.6	0.6	0.0

7g

^{1}H, DMSO-d6, 500 MHz

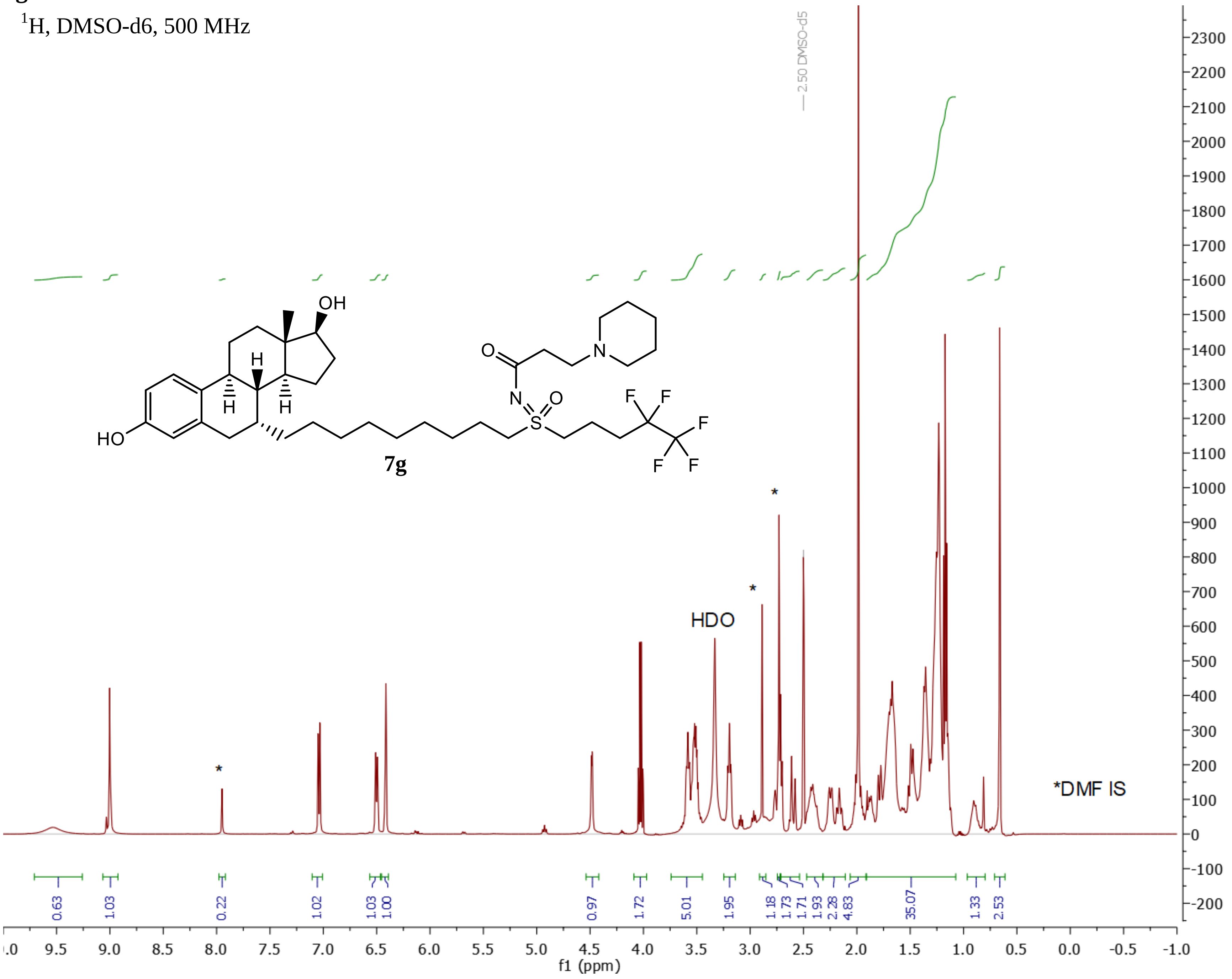

^{13}C, DMSO-d6, 126 MHz

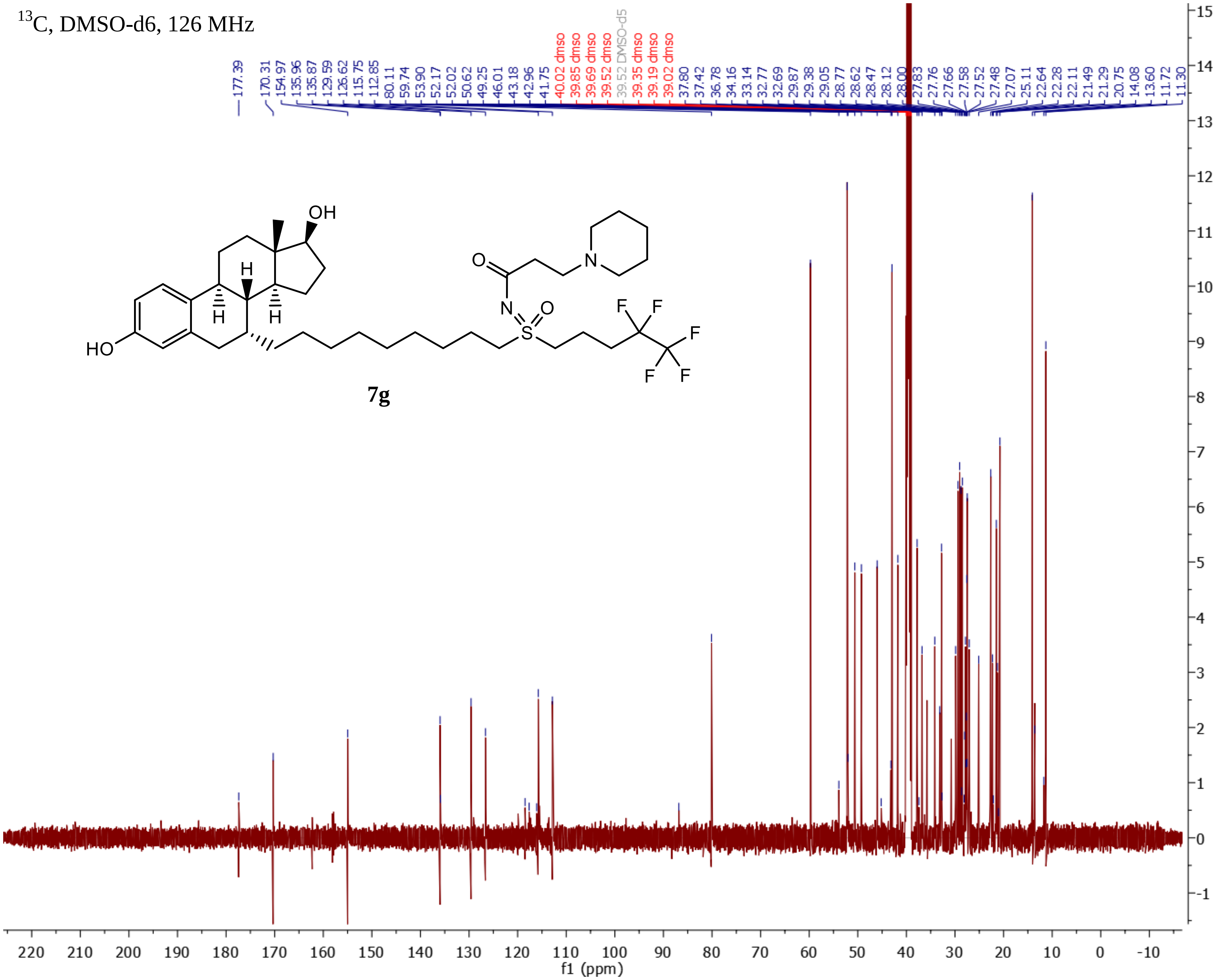

7g

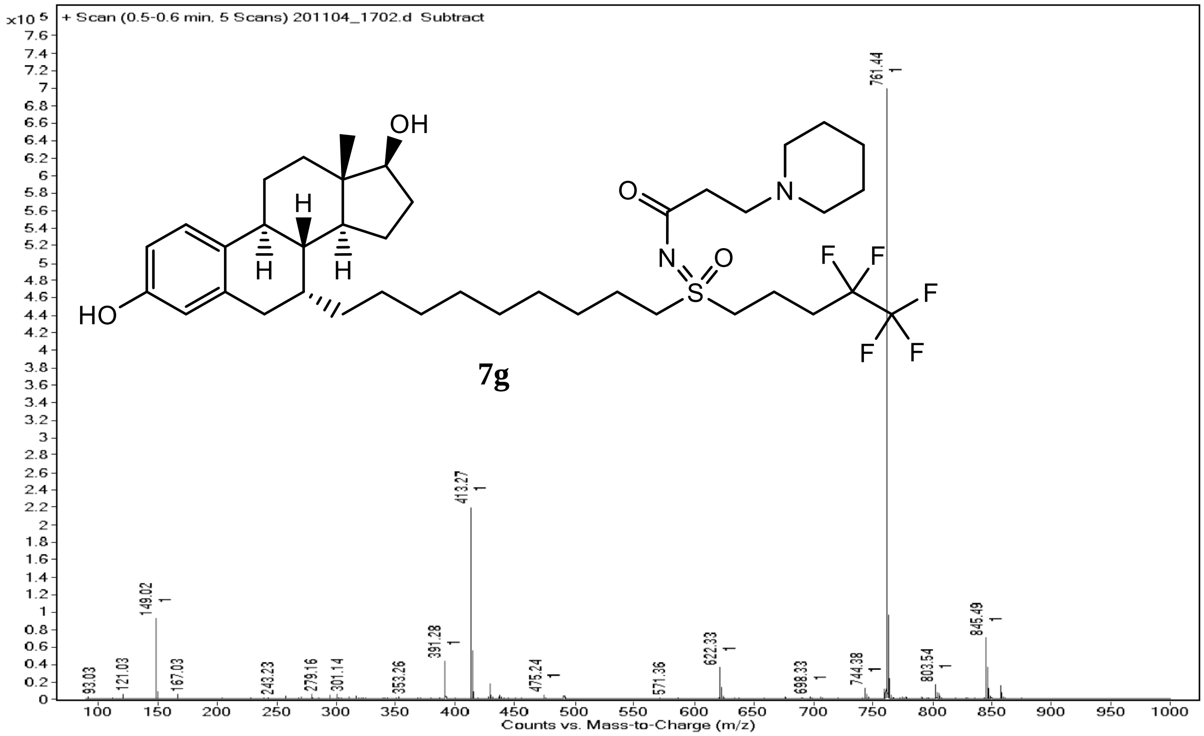

Target Ion Species

Ion Species	*m/z*	*Ionic Formula*
(M+H)+	761.4362	C40 H62 F5 N2 O4 S

MFG Calculator Results

Target m/z	*Ionic Formula*	*Calc m/z*	*+/- (mDa)*	*+/- (ppm)*	*DBE*	*MFG Score*
761.4362	C41 H58 F5 N6 S	761.4358	0.4	0.5	14.0	98.67
761.4362	C35 H60 F3 N8 O5 S	761.4354	0.8	1.1	9.0	98.36
761.4362	C42 H63 F2 N2 O6 S	761.4369	-0.7	-0.9	12.0	97.37
761.4362	C32 H61 F4 N8 O6 S	761.4365	-0.3	-0.4	5.0	97.33
761.4362	C37 H58 F N8 O8	761.4356	0.6	0.8	13.0	97.08
761.4362	C39 H61 F4 N2 O8	761.4359	0.3	0.4	9.0	96.54
761.4362	C40 H62 F5 N2 O4 S	761.4345	1.7	2.2	9.0	96.41
761.4362	C37 H61 N8 O7 S	761.4378	-1.6	-2.1	12.0	96.19
761.4362	C45 H62 F N2 O5 S	761.4358	0.4	0.5	16.0	96.03
761.4362	C34 H59 F2 N8 O9	761.4368	-0.6	-0.8	9.0	95.34

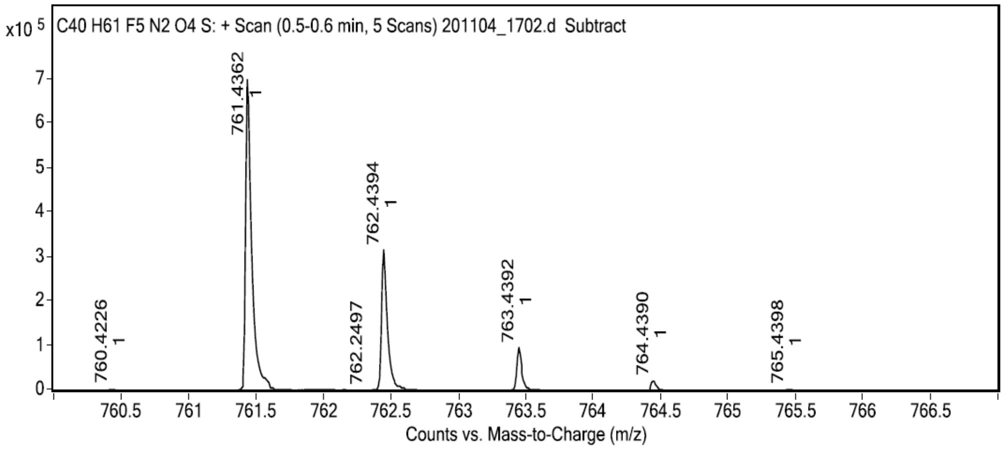

Predicted Isotope Match Table

Isotope	*m/z*	*Calc m/z*	Diff (mDa)	Abund (%)	Calc Abund (%)	+/-
1	761.4362	761.4345	1.7	100.0	100.0	0.0
2	762.4394	762.4377	1.7	44.3	45.6	1.3
3	763.4392	763.4378	1.4	13.6	15.5	1.9
4	764.4390	764.4385	0.5	3.2	3.9	0.7
5	765.4398	765.4399	-0.1	0.7	0.7	0.0
6	766.4379	766.4417	-3.8	0.1	0.1	0.0

7h

^{1}H, DMSO-d6, 500 MHz

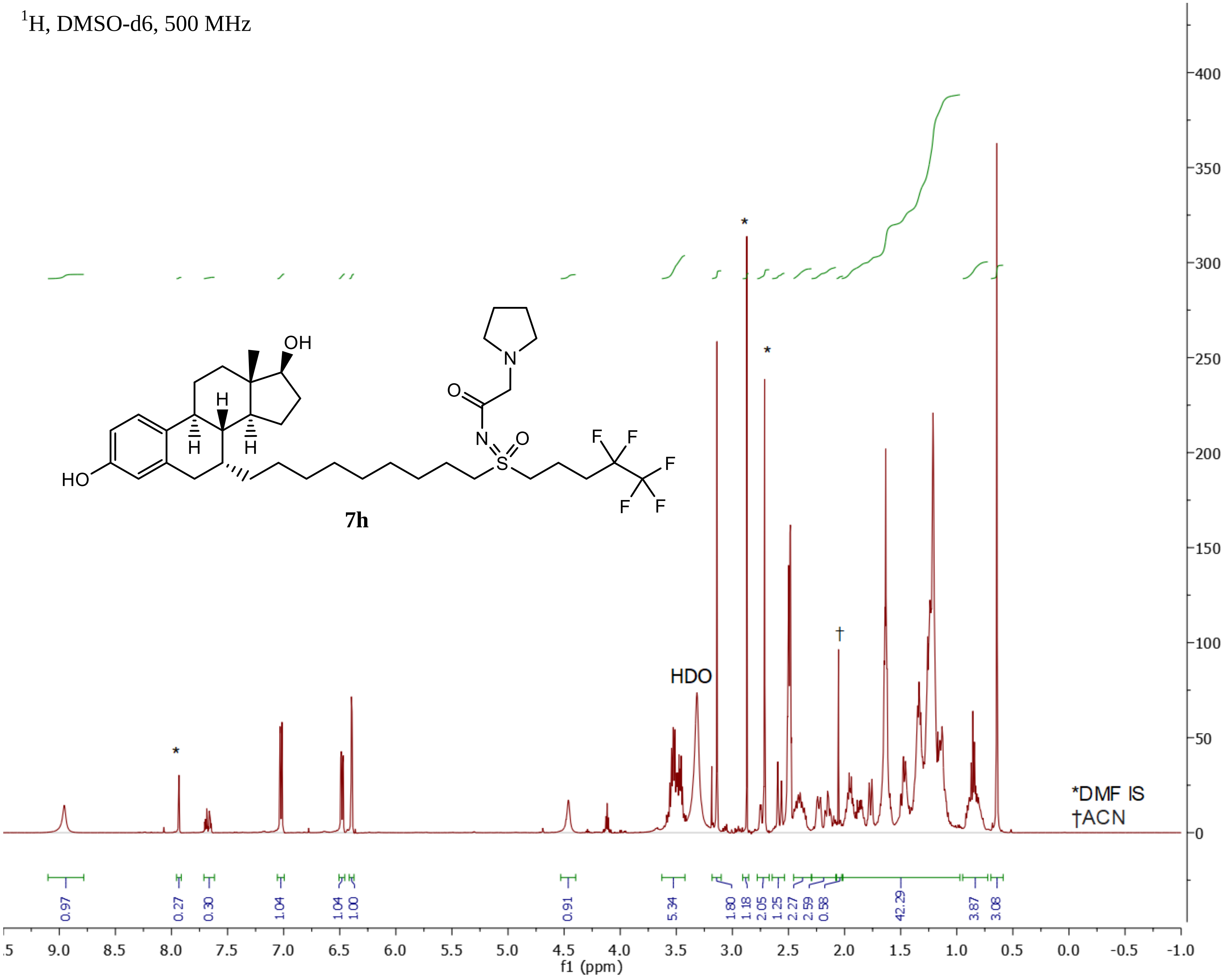

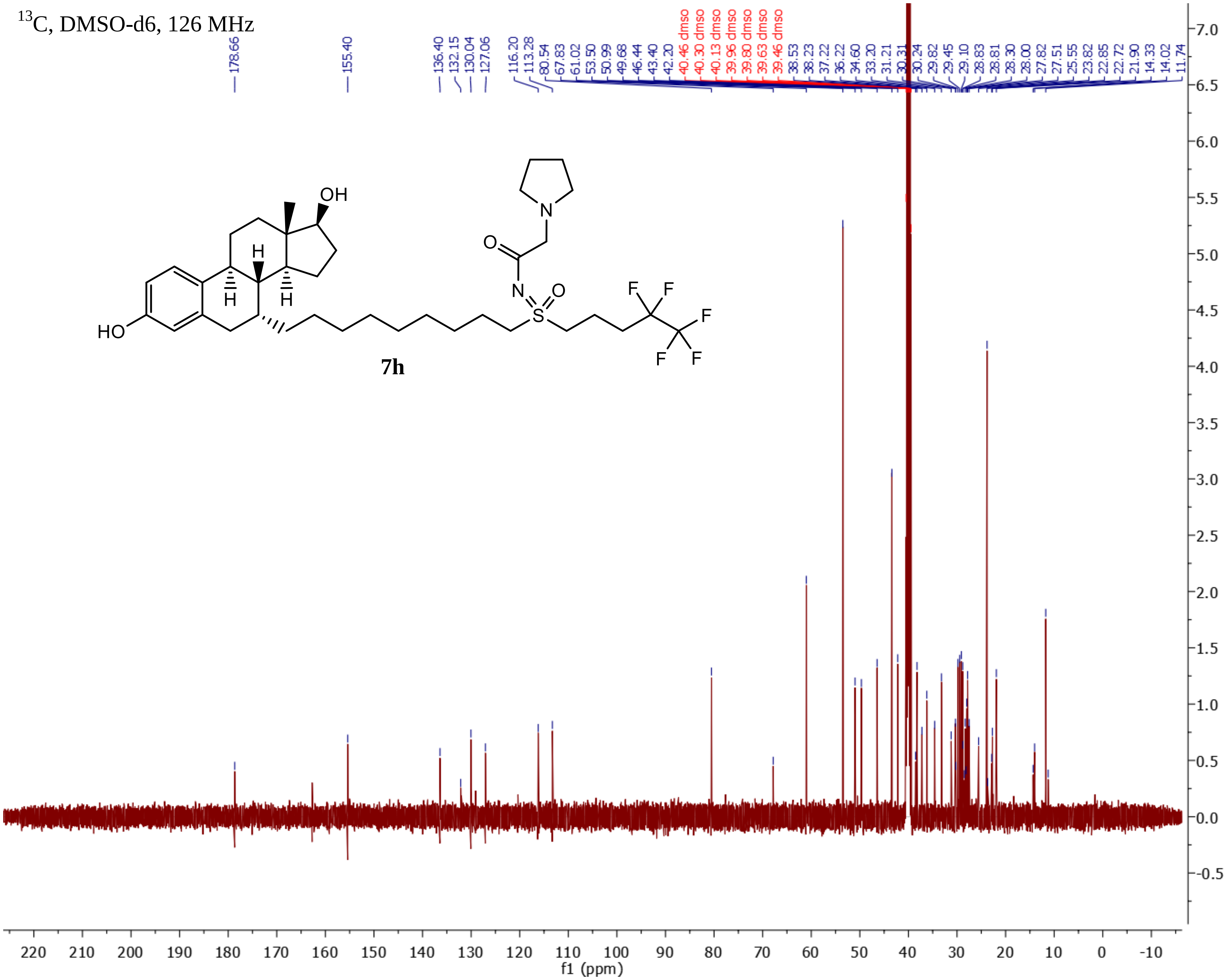

13C, DMSO-d6, 126 MHz
178.66
155.40
136.40
132.15
130.04
127.06
116.20
113.28
80.54
67.83
61.02
53.50
50.99
49.68
46.44
43.40
42.20
40.46 dmso
40.30 dmso
40.13 dmso
39.96 dmso
39.80 dmso
39.63 dmso
39.46 dmso
38.53
38.23
37.22
36.22
34.60
33.20
31.21
30.31
30.24
29.82
29.45
29.10
28.83
28.81
28.30
28.00
27.82
27.51
25.55
23.82
22.85
22.72
21.90
14.33
14.02
11.74
OH
HO
H
N
O
S
F
7h
f1 (ppm)

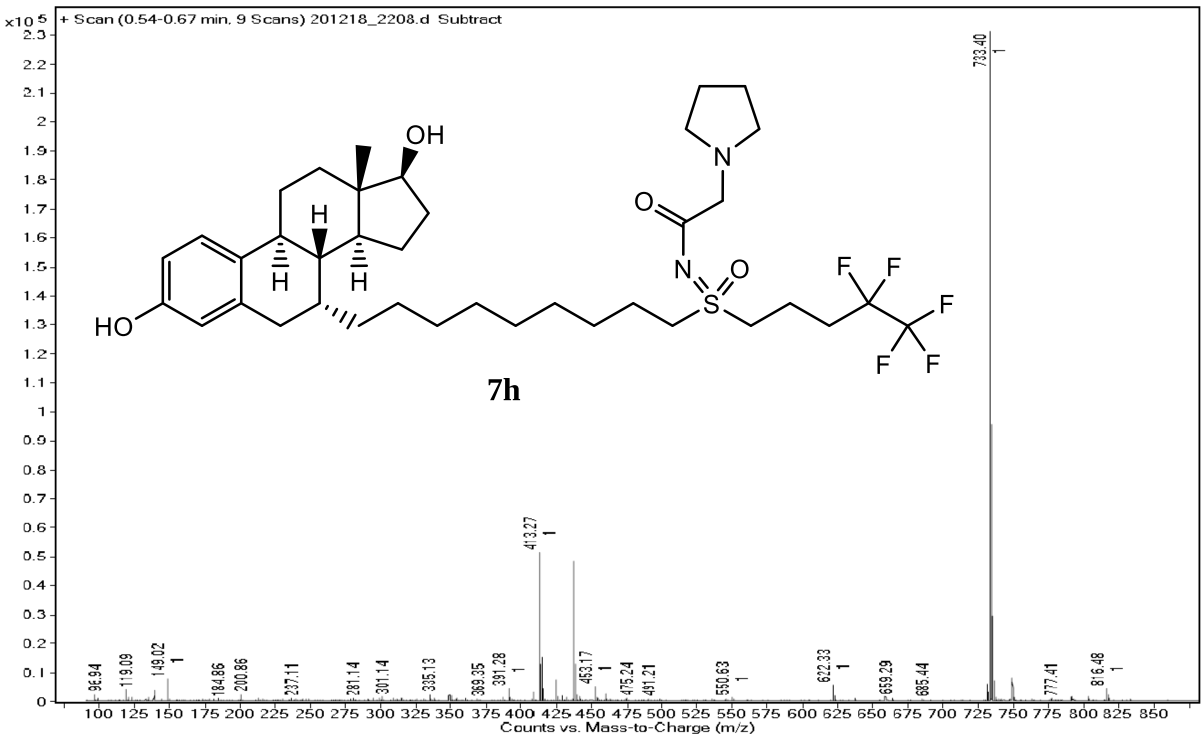

Target Ion Species

Ion Species	*m/z*	*Ionic Formula*
(M+H)+	733.4036	C38 H58 F5 N2 O4 S

MFG Calculator Results

Target m/z	*Ionic Formula*	*Calc m/z*	*+/- (mDa)*	*+/- (ppm)*	*DBE*	*MFG Score*
733.4036	C38 H58 F5 N2 O4 S	733.4032	0.4	0.5	9.0	99.66
733.4036	C30 H54 F5 N8 O7	733.4030	0.6	0.8	6.0	98.38
733.4036	C39 H54 F5 N6 S	733.4045	-0.9	-1.2	14.0	96.88
733.4036	C46 H54 F5 O2	733.4038	-0.2	-0.3	18.0	95.02
733.4036	C42 H50 F5 N6	733.4012	2.4	3.3	19.0	89.93
733.4036	C34 H58 F5 N2 O9	733.4057	-2.1	-2.9	5.0	83.20
733.4036	C27 H58 F5 N8 O7 S	733.4064	-2.8	-3.8	1.0	82.93
733.4036	C34 H54 F5 N8 O2 S	733.4005	3.1	4.2	10.0	80.80
733.4036	C41 H54 F5 N2 O4	733.3998	3.8	5.2	14.0	78.07
733.4036	C43 H58 F5 O2 S	733.4072	-3.6	-4.9	13.0	69.61

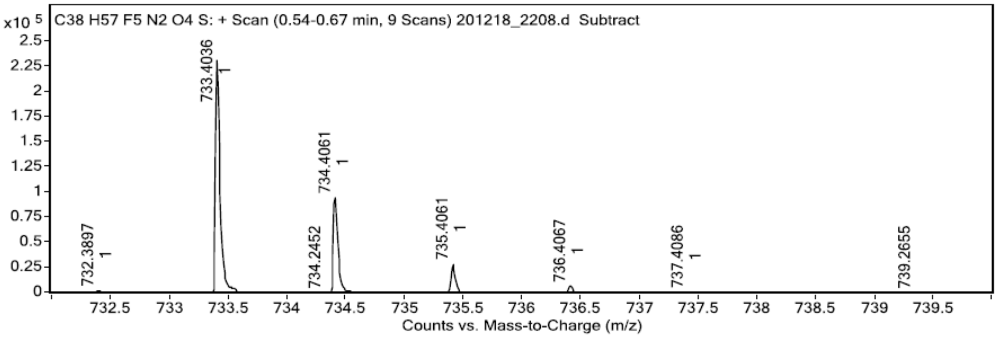

Predicted Isotope Match Table

Isotope	*m/z*	*Calc m/z*	Diff (mDa)	Abund (%)	Calc Abund (%)	+/-
1	733.4036	733.4032	0.4	100.0	100.0	0.0
2	734.4061	734.4064	-0.3	41.2	43.4	2.2
3	735.4061	735.4062	-0.1	12.6	14.5	1.9
4	736.4067	736.4069	-0.2	3.0	3.5	0.5
5	737.4086	737.4084	0.2	0.6	0.7	0.1
6	738.4057	738.4102	-4.5	0.1	0.1	0.0

7i

^{1}H, DMSO-d6, 500 MHz

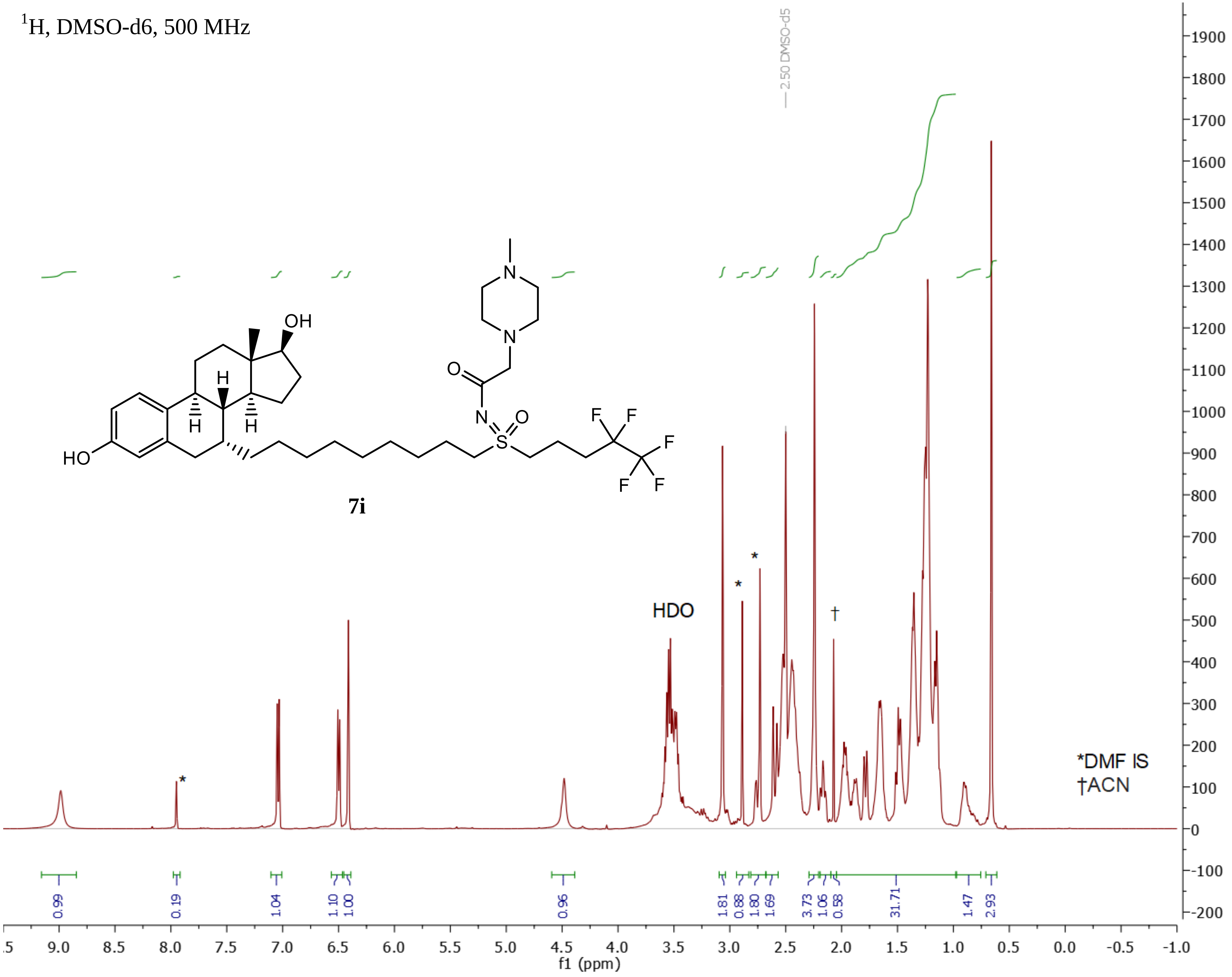

^{13}C, DMSO-d6, 126 MHz

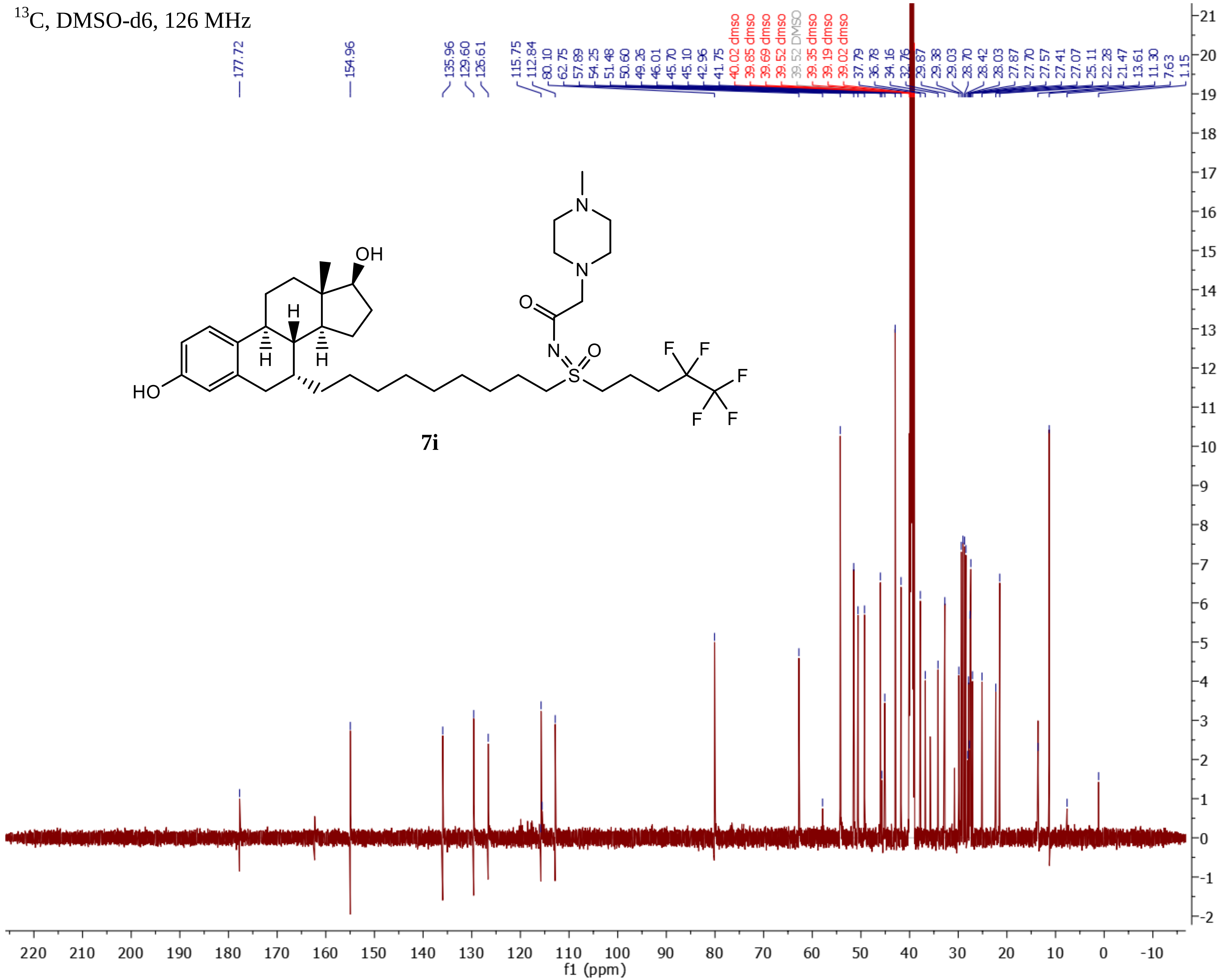

7i

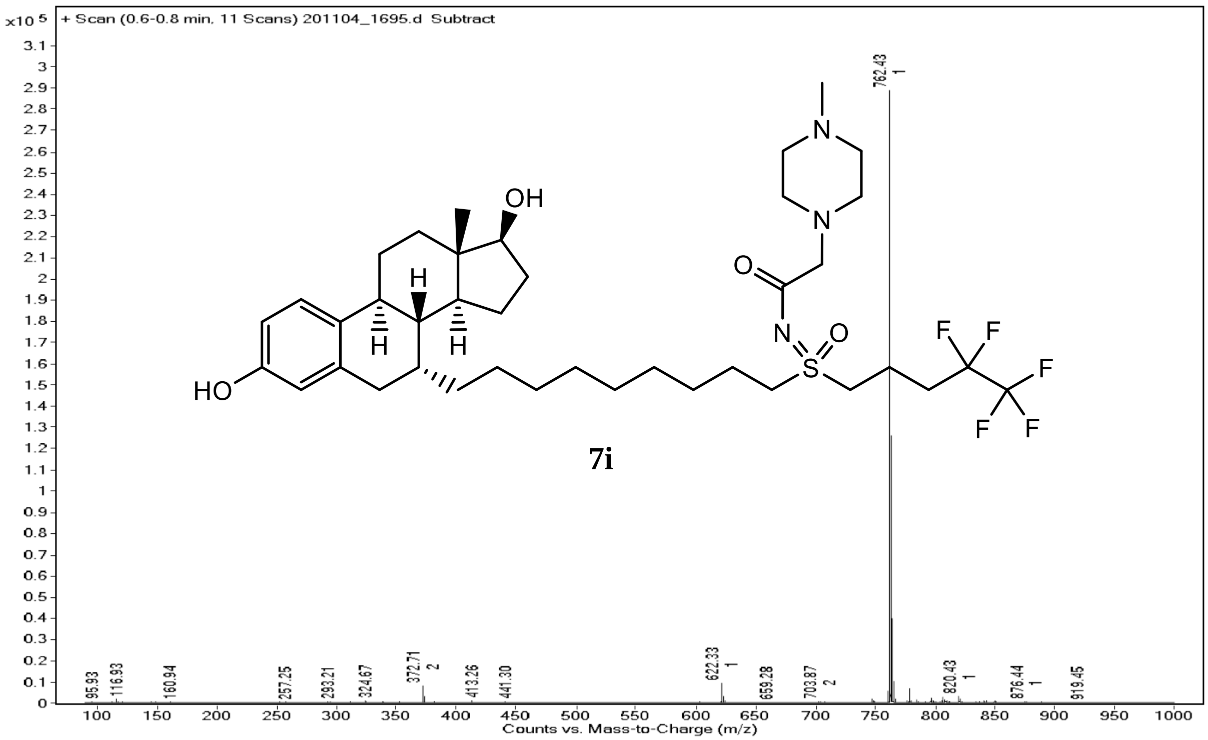

Target Ion Species

Ion Species	m/z	Ionic Formula
(M+H)+	762.4294	C39 H61 F5 N3 O4 S

MFG Calculator Results

Target m/z	Ionic Formula	Calc m/z	+/- (mDa)	+/- (ppm)	DBE	MFG Score
762.4294	C39 H61 F5 N3 O4 S	762.4297	-0.3	-0.4	9.0	99.47
762.4294	C37 H58 F2 N9 O4 S	762.4295	-0.1	-0.1	13.0	99.35
762.4294	C34 H59 F3 N9 O5 S	762.4306	-1.2	-1.6	9.0	97.78
762.4294	C33 H63 F3 N5 O9 S	762.4293	0.1	0.1	4.0	97.50
762.4294	C42 H60 F4 N3 O3 S	762.4286	0.8	1.0	13.0	97.49
762.4294	C36 H62 F2 N5 O8 S	762.4282	1.2	1.6	8.0	97.02
762.4294	C40 H57 F N9 O3 S	762.4284	1.0	1.3	17.0	96.47
762.4294	C39 H56 N9 O7	762.4297	-0.3	-0.4	17.0	96.41
762.4294	C40 H57 F5 N7 S	762.4311	-1.7	-2.2	14.0	95.94
762.4294	C34 H56 F4 N9 O6	762.4284	1.0	1.3	10.0	95.34

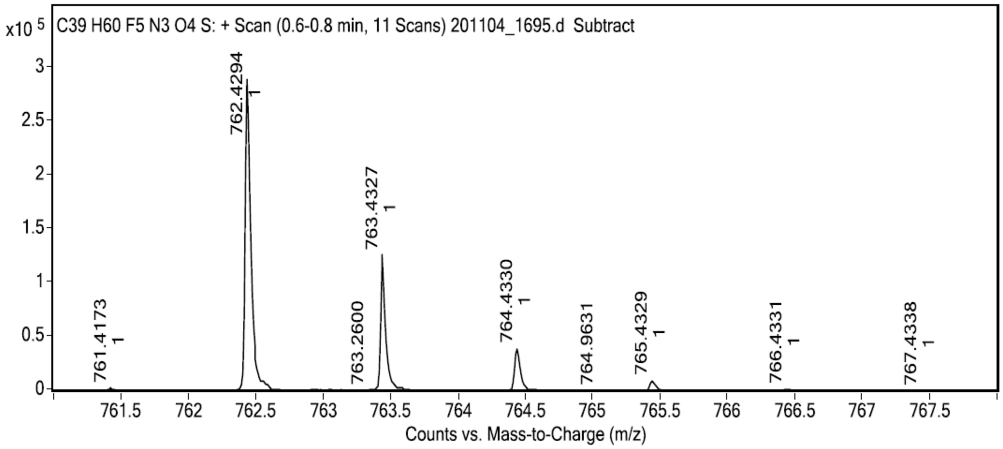

Predicted Isotope Match Table

Isotope	m/z	Calc m/z	Diff (mDa)	Abund (%)	Calc Abund (%)	+/-
1	762.4294	762.4297	-0.3	100.0	100.0	0.0
2	763.4327	763.4329	-0.2	43.6	44.9	1.3
3	764.4330	764.4329	0.1	13.6	15.2	1.6
4	765.4329	765.4335	-0.6	3.3	3.8	0.5
5	766.4331	766.4350	-1.9	0.6	0.7	0.1

7j

^{1}H, DMSO-d6, 500 MHz

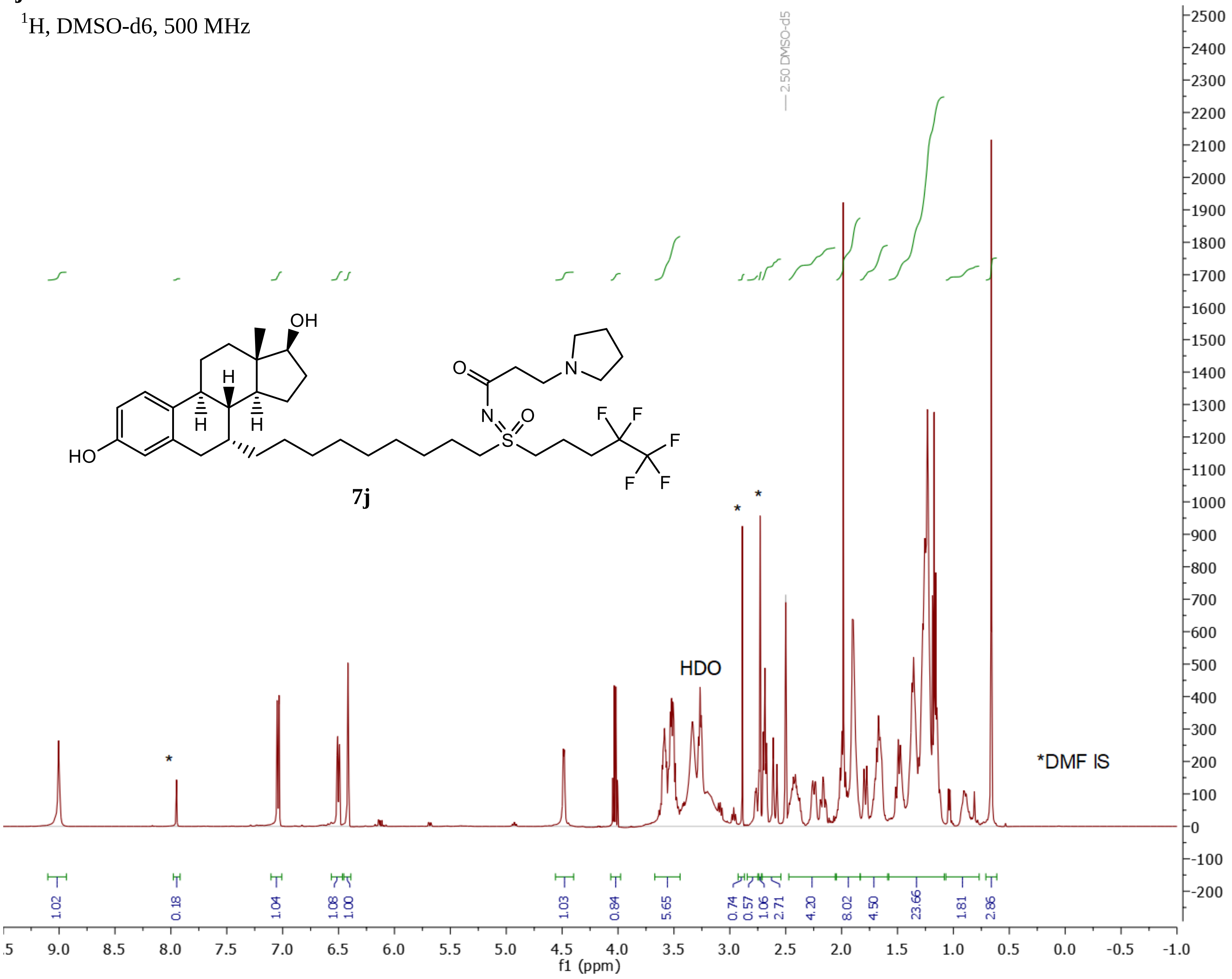

^{13}C, DMSO-d6, 126 MHz

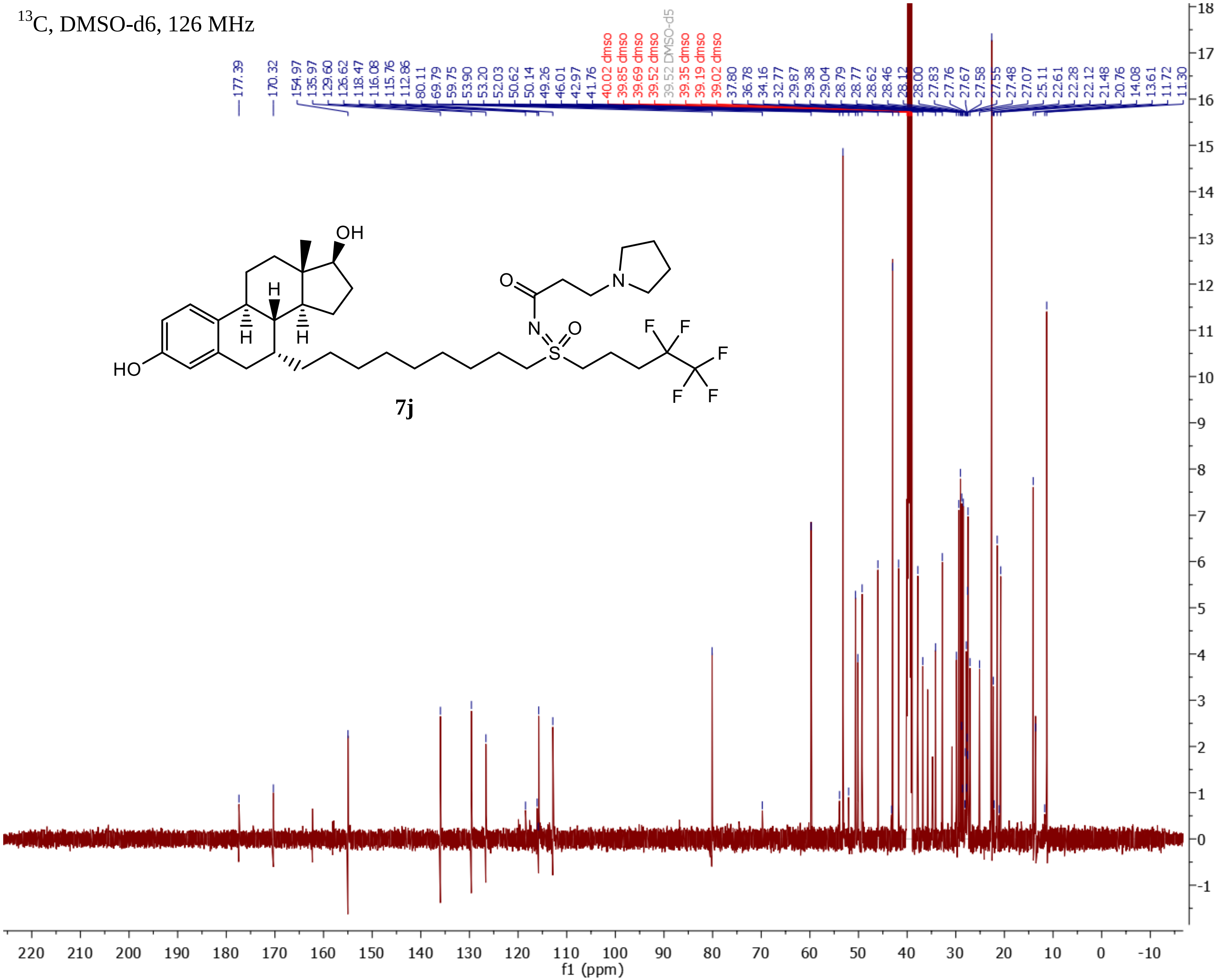

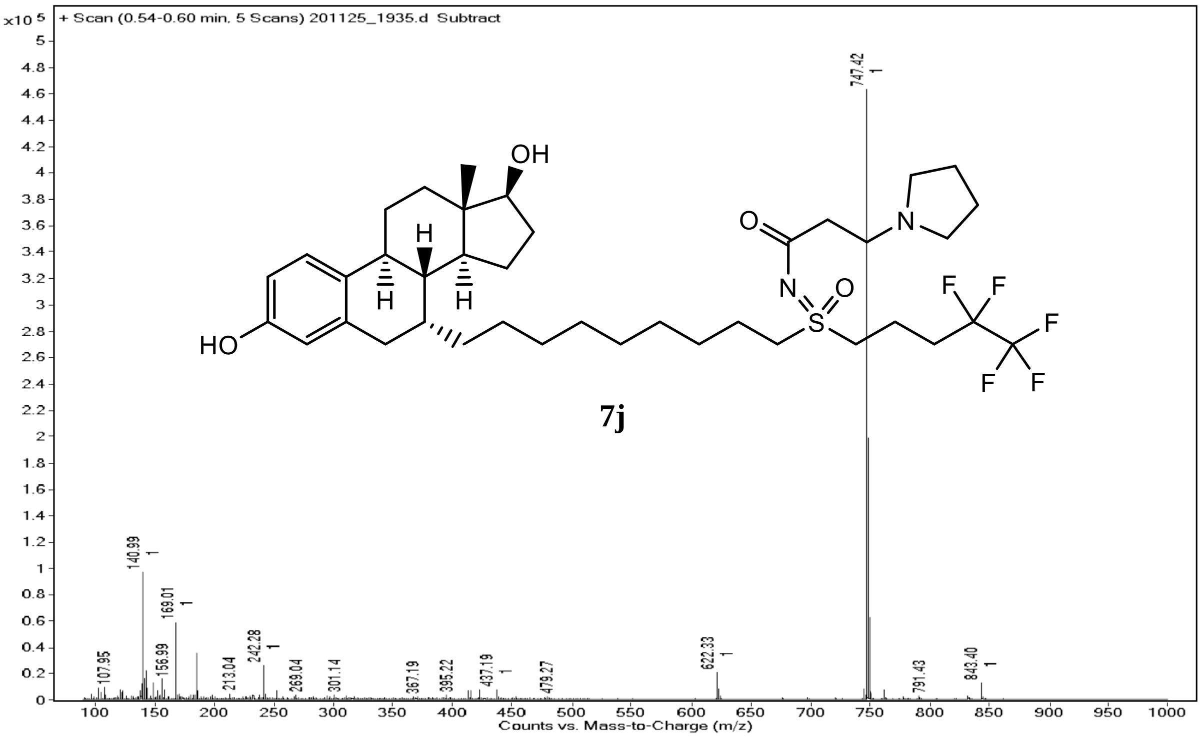

Target Ion Species

Ion Species	m/z	Ionic Formula
(M+H)+	747.4182	C39 H60 F5 N2 O4 S

MFG Calculator Results

Target m/z	Ionic Formula	Calc m/z	+/- (mDa)	+/- (ppm)	DBE	MFG Score
747.4182	C39 H60 F5 N2 O4 S	747.4188	-0.6	-0.8	9.0	98.17
747.4182	C35 H56 F5 N8 O2 S	747.4162	2.0	2.7	10.0	95.39
747.4182	C40 H56 F5 N6 S	747.4202	-2.0	-2.7	14.0	92.83
747.4182	C31 H56 F5 N8 O7	747.4187	-0.5	-0.7	6.0	92.24
747.4182	C43 H52 F5 N6	747.4168	1.4	1.9	19.0	90.03
747.4182	C42 H56 F5 N2 O4	747.4155	2.7	3.6	14.0	88.03
747.4182	C34 H60 F5 N4 O6 S	747.4148	3.4	4.5	5.0	87.78
747.4182	C47 H56 F5 O2	747.4195	-1.3	-1.7	18.0	83.89
747.4182	C35 H60 F5 N2 O9	747.4213	-3.1	-4.1	5.0	79.76
747.4182	C28 H60 F5 N8 O7 S	747.4220	-3.8	-5.1	1.0	79.46

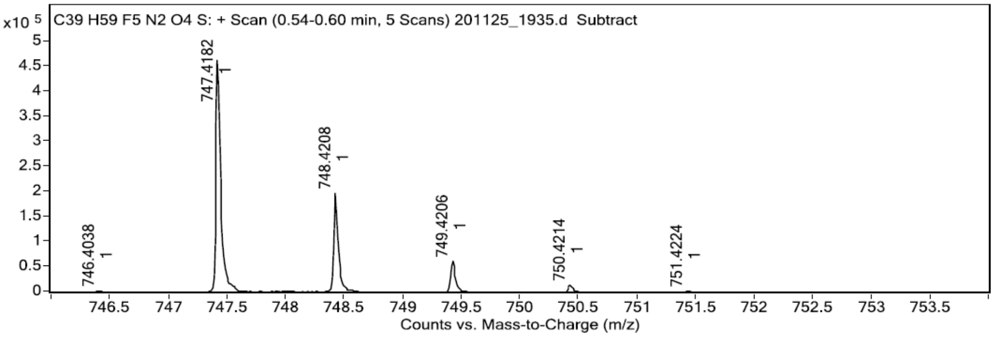

Predicted Isotope Match Table

Isotope	m/z	Calc m/z	Diff (mDa)	Abund (%)	Calc Abund (%)	+/-
1	747.4182	747.4188	-0.6	100.0	100.0	0.0
2	748.4208	748.4221	-1.3	42.6	44.5	1.9
3	749.4206	749.4220	-1.4	13.2	15.0	1.8
4	750.4214	750.4227	-1.3	3.1	3.7	0.6
5	751.4224	751.4241	-1.7	0.6	0.7	0.1
6	752.4216	752.4260	-4.4	0.1	0.1	0.0

7k

^{1}H, DMSO-d6, 500 MHz

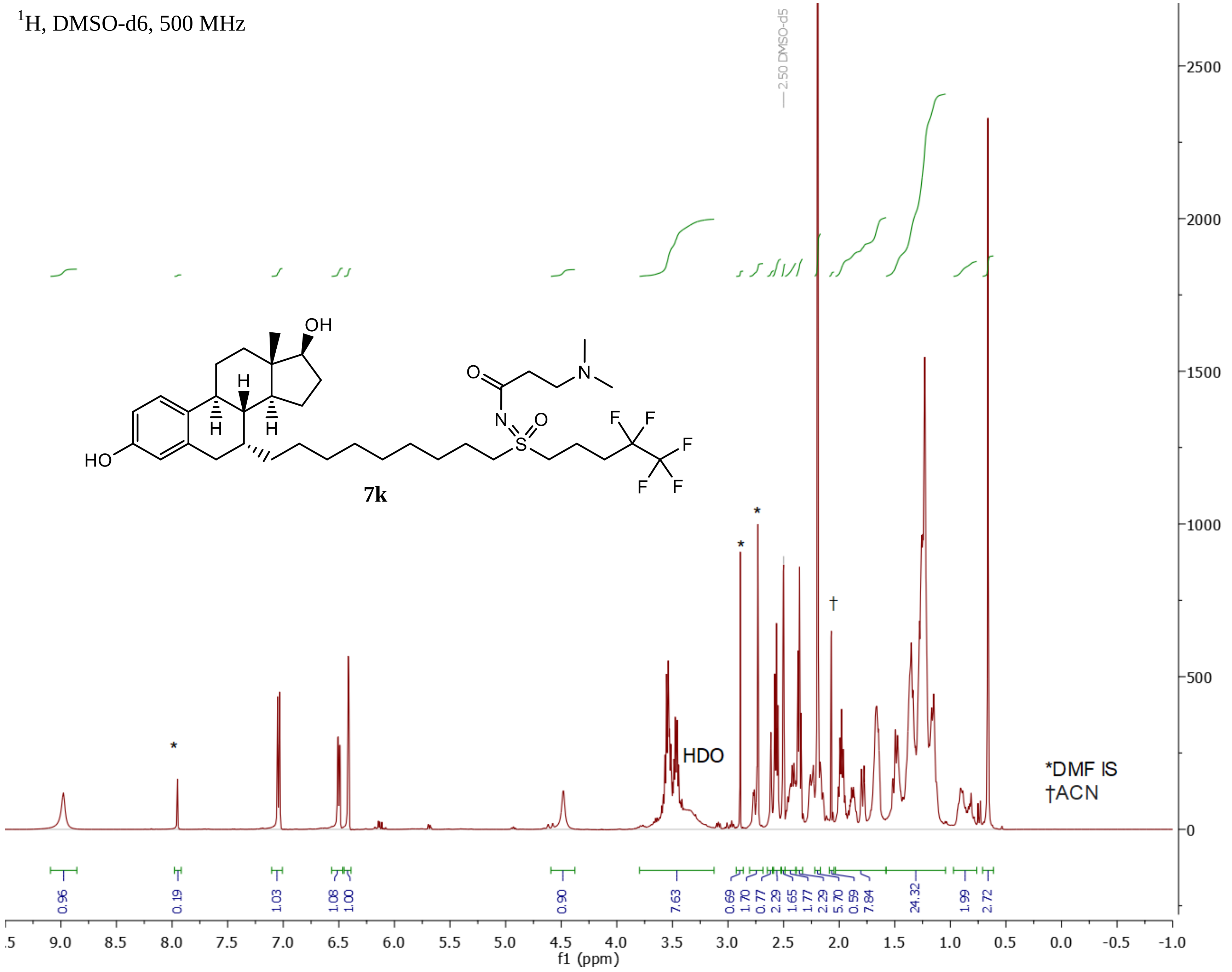

13C, DMSO-d6, 126 MHz

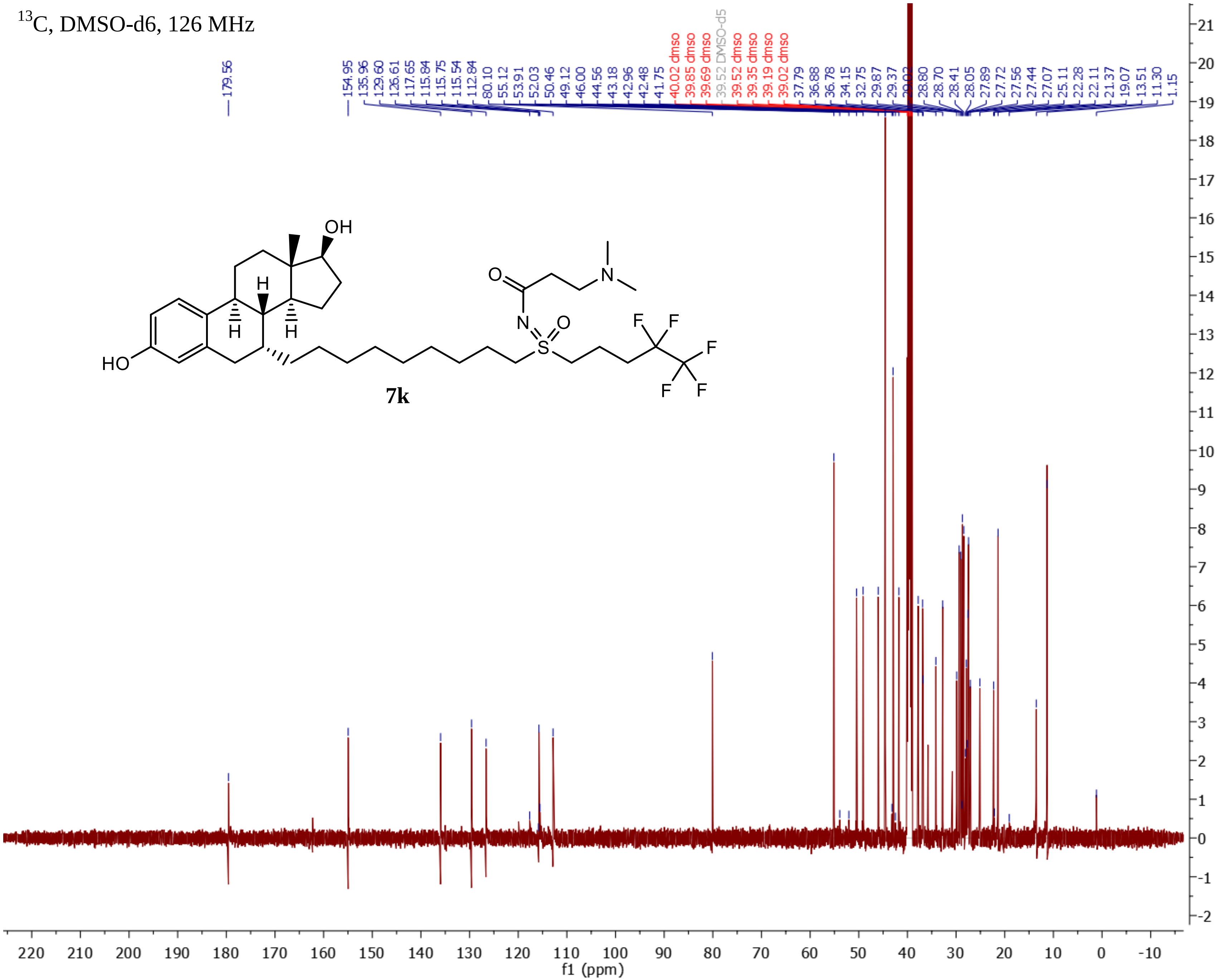

7k

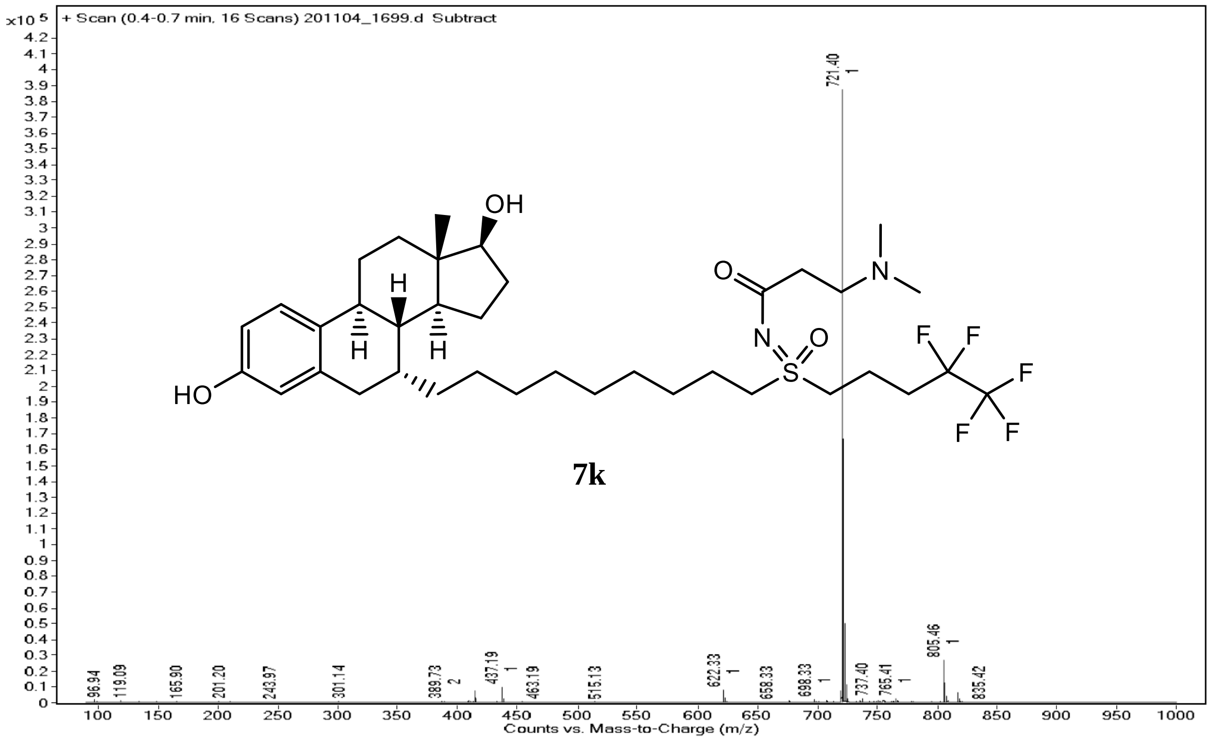

Target Ion Species

Ion Species	m/z	Ionic Formula
(M+H)+	721.4032	C37 H58 F5 N2 O4 S

MFG Calculator Results

Target m/z	Ionic Formula	Calc m/z	+/- (mDa)	+/- (ppm)	DBE	MFG Score
721.4032	C37 H58 F5 N2 O4 S	721.4032	0.0	0.0	8.0	99.65
721.4032	C35 H55 F2 N8 O4 S	721.4030	0.2	0.3	12.0	99.49
721.4032	C32 H56 F3 N8 O5 S	721.4041	-0.9	-1.2	8.0	98.13
721.4032	C38 H54 F5 N6 S	721.4045	-1.3	-1.8	13.0	97.10
721.4032	C40 H57 F4 N2 O3 S	721.4021	1.1	1.5	12.0	97.10
721.4032	C31 H60 F3 N4 O9 S	721.4028	0.4	0.6	3.0	96.51
721.4032	C37 H53 N8 O7	721.4032	0.0	0.0	16.0	96.40
721.4032	C38 H54 F N8 O3 S	721.4018	1.4	1.9	16.0	95.99
721.4032	C34 H59 F2 N4 O8 S	721.4016	1.6	2.2	7.0	95.70
721.4032	C29 H57 N10 O9 S	721.4025	0.7	1.0	7.0	95.64

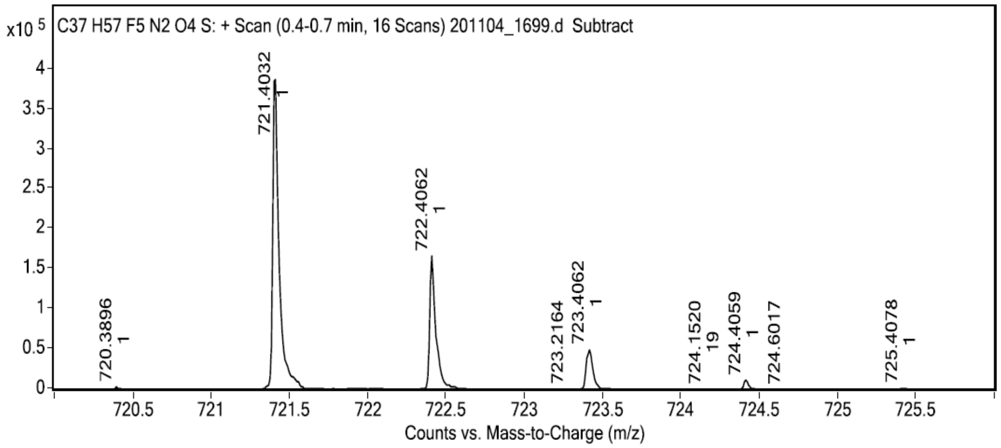

Predicted Isotope Match Table

Isotope	m/z	Calc m/z	Diff (mDa)	Abund (%)	Calc Abund (%)	+/-
1	721.4032	721.4032	0.0	100.0	100.0	0.0
2	722.4062	722.4064	-0.2	41.8	42.4	0.6
3	723.4062	723.4061	0.1	12.7	14.0	1.3
4	724.4059	724.4068	-0.9	2.9	3.4	0.5
5	725.4078	725.4083	-0.5	0.5	0.6	0.1

7l

^{1}H, DMSO-d6, 500 MHz

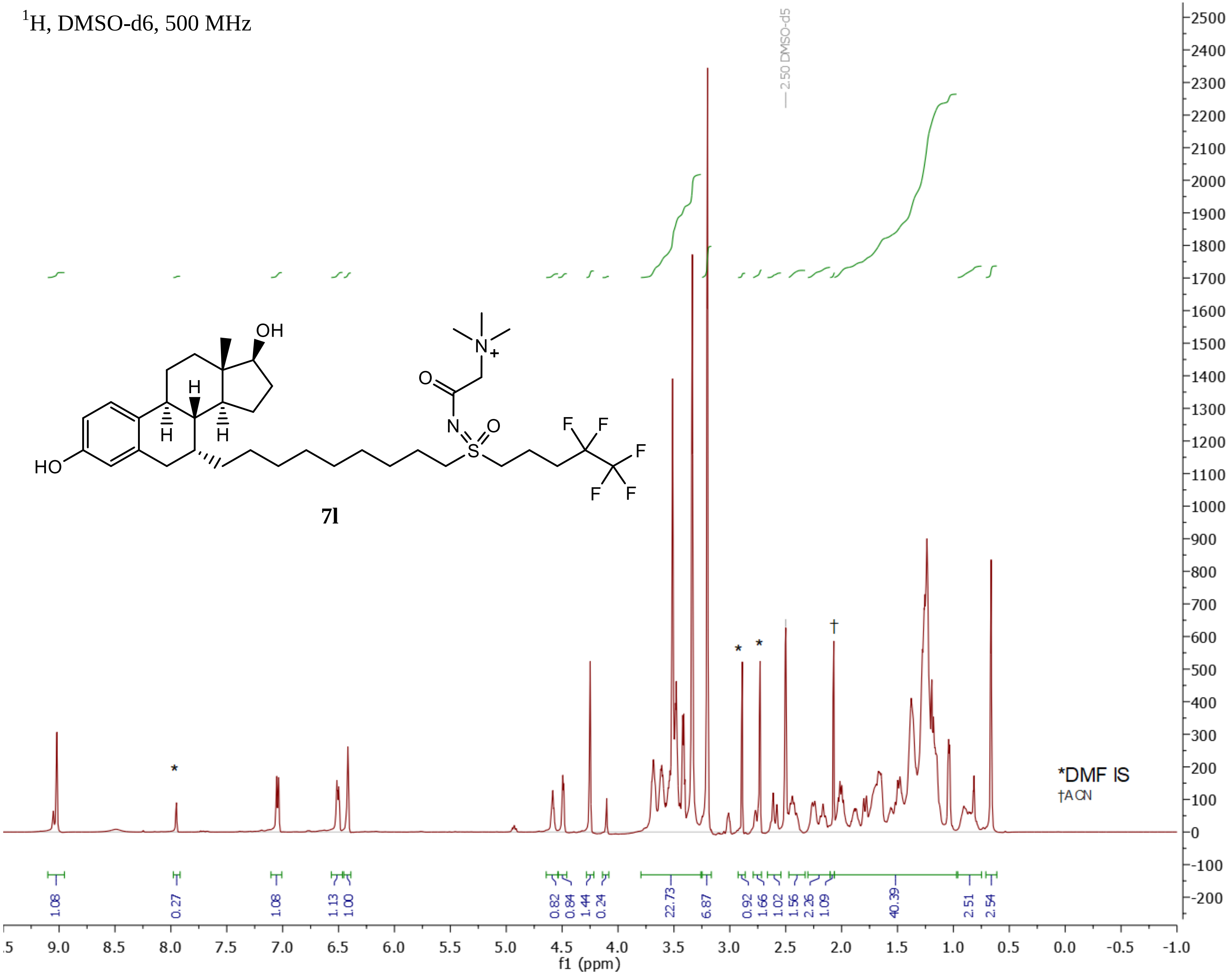

^{13}C, DMSO-d6, 126 MHz

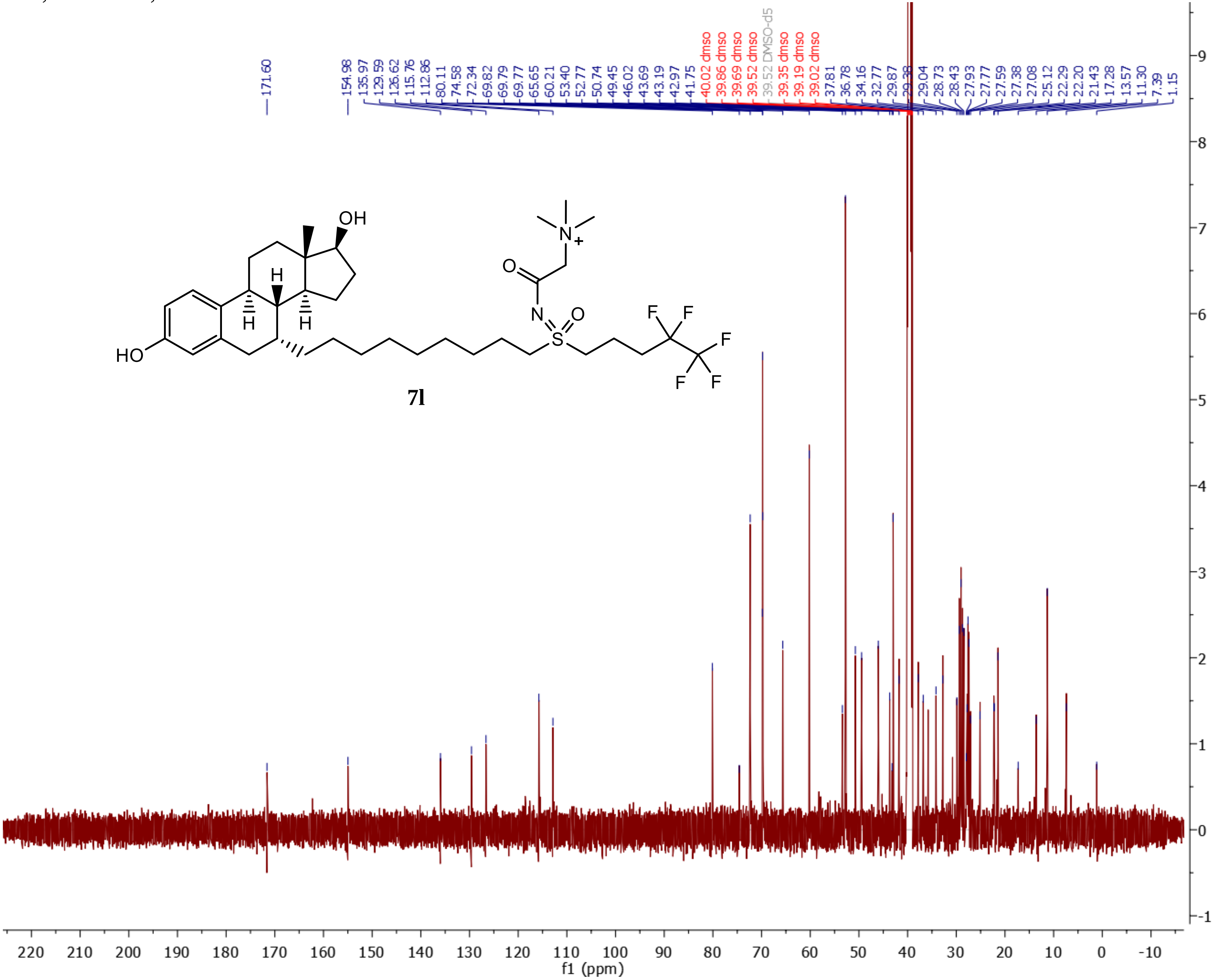

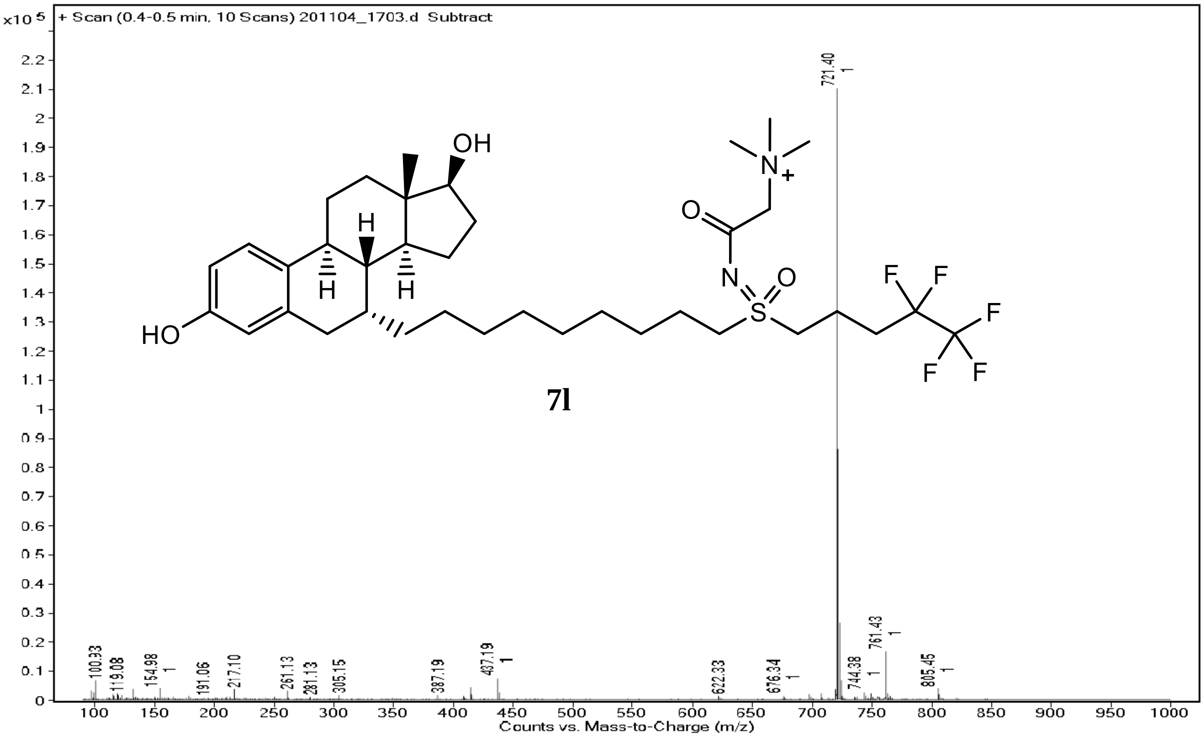

7l

Target Ion Species

Ion Species	m/z	Ionic Formula
M+	721.4025	C37 H58 F5 N2 O4 S

MFG Calculator Results

Target m/z	Ionic Formula	Calc m/z	+/- (mDa)	+/- (ppm)	DBE	MFG Score
721.4025	C35 H55 F2 N8 O4 S	721.4030	-0.5	-0.7	11.5	98.92
721.4025	C34 H59 F2 N4 O8 S	721.4016	0.9	1.2	6.5	98.70
721.4025	C29 H57 N10 O9 S	721.4025	0.0	0.0	6.5	98.32
721.4025	C31 H60 F3 N4 O9 S	721.4028	-0.3	-0.4	2.5	98.02
721.4025	C37 H58 F5 N2 O4 S	721.4032	-0.7	-1.0	7.5	97.96
721.4025	C40 H57 F4 N2 O3 S	721.4021	0.4	0.6	11.5	97.06
721.4025	C38 H54 F N8 O3 S	721.4018	0.7	1.0	15.5	97.02
721.4025	C32 H56 F3 N8 O5 S	721.4041	-1.6	-2.2	7.5	96.16
721.4025	C33 H54 F5 N8 O2 S	721.4005	2.0	2.8	8.5	95.31
721.4025	C32 H53 F4 N8 O6	721.4019	0.6	0.8	8.5	94.77

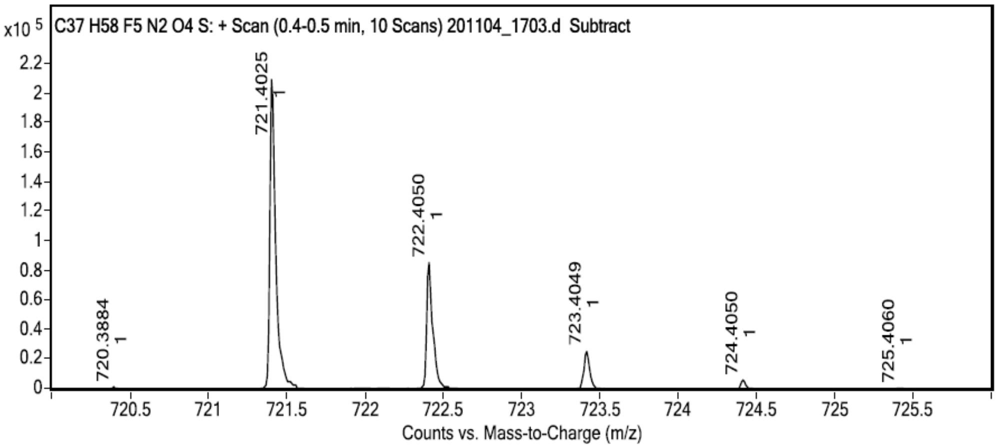

Predicted Isotope Match Table

Isotope	m/z	Calc m/z	Diff (mDa)	Abund (%)	Calc Abund (%)	+/-
1	721.4025	721.4032	-0.7	100.0	100.0	0.0
2	722.4050	722.4064	-1.4	40.1	42.4	2.3
3	723.4049	723.4061	-1.2	12.4	14.0	1.6
4	724.4050	724.4068	-1.8	3.0	3.4	0.4
5	725.4060	725.4083	-2.3	0.6	0.6	0.0

7m

^{1}H, DMSO-d6, 500 MHz

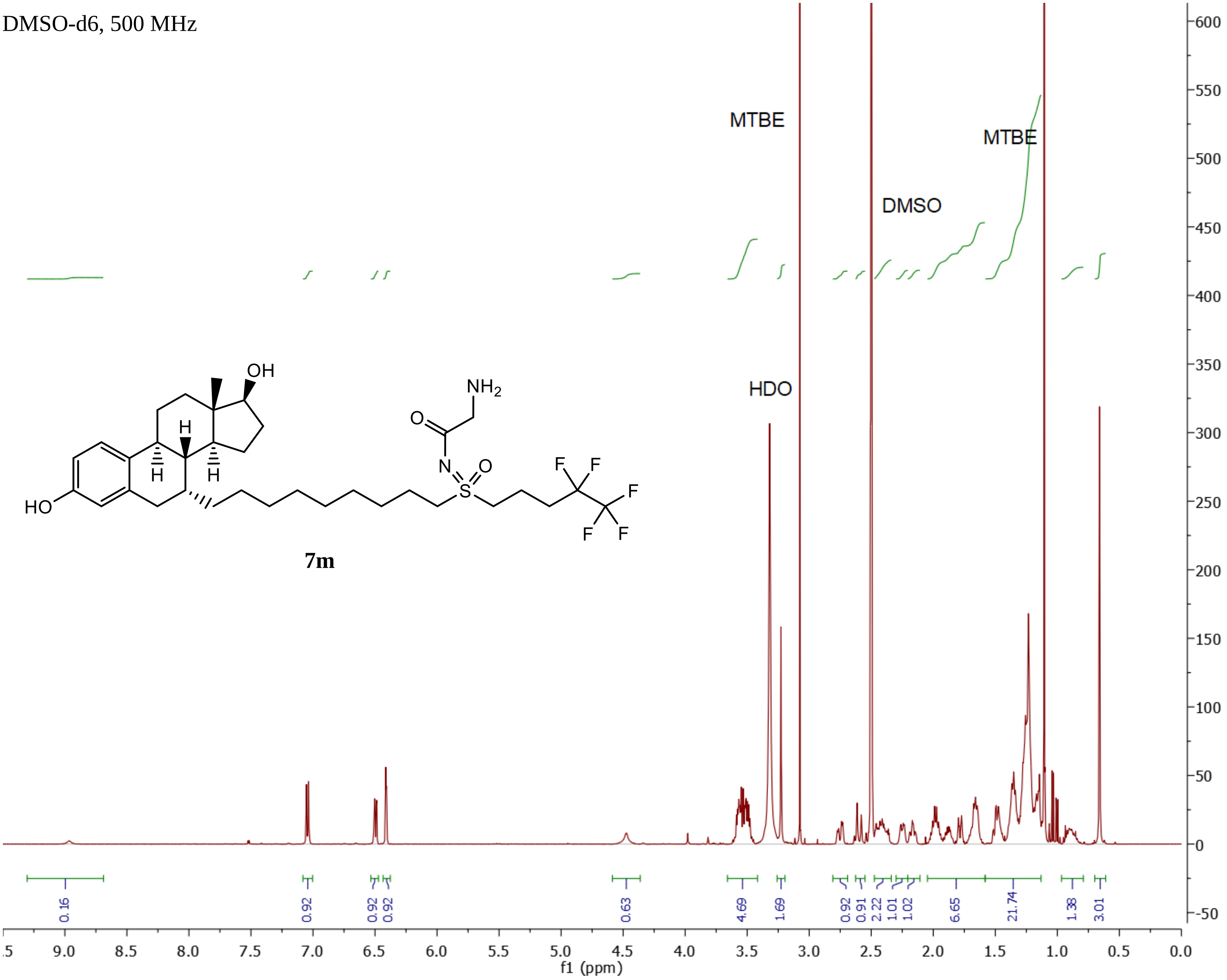

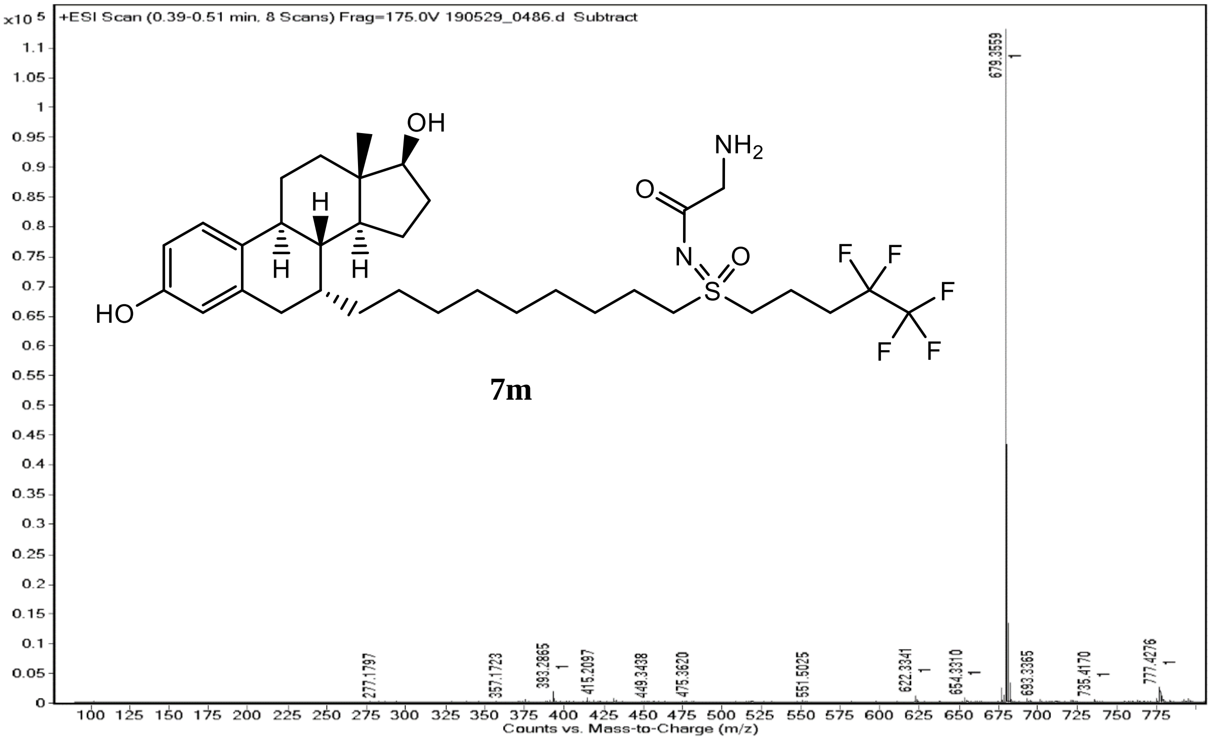

Target Ion Species

Ion Species	m/z	Ionic Formula
(M+H)+	679.3559	C34 H52 F5 N2 O4 S

MFG Calculator Results

Target m/z	Ionic Formula	Calc m/z	+/- (mDa)	+/- (ppm)	DBE	MFG Score
679.3559	C34 H52 F5 N2 O4 S	679.3562	-0.3	-0.4	8.0	99.60
679.3559	C32 H49 F2 N8 O4 S	679.3560	-0.1	-0.1	12.0	99.48
679.3559	C37 H51 F4 N2 O3 S	679.3551	0.8	1.2	12.0	97.82
679.3559	C29 H50 F3 N8 O5 S	679.3571	-1.2	-1.8	8.0	96.95
679.3559	C31 H53 F2 N4 O8 S	679.3547	1.2	1.8	7.0	96.67
679.3559	C35 H48 F N8 O3 S	679.3549	1.0	1.5	16.0	96.66
679.3559	C28 H54 F3 N4 O9 S	679.3558	0.1	0.1	3.0	96.44
679.3559	C35 H48 F5 N6 S	679.3576	-1.7	-2.5	13.0	95.55
679.3559	C26 H51 N10 O9 S	679.3556	0.3	0.4	7.0	95.53
679.3559	C34 H47 N8 O7	679.3562	-0.3	-0.4	16.0	95.42

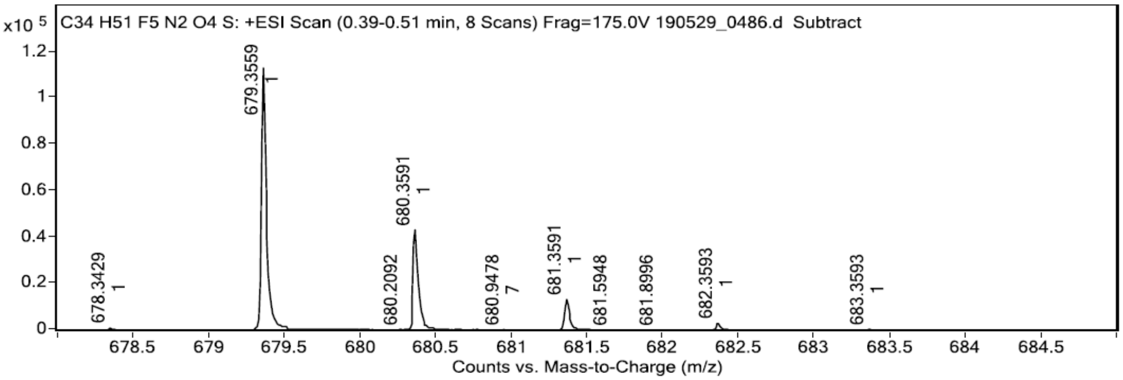

Predicted Isotope Match Table

Isotope	m/z	Calc m/z	Diff (mDa)	Abund (%)	Calc Abund (%)	+/-
1	679.3559	679.3562	-0.3	100.0	100.0	0.0
2	680.3591	680.3595	-0.4	38.5	39.0	0.5
3	681.3591	681.3588	0.3	11.8	12.7	0.9

9

^{1}H, DMSO-d6, 500 MHz

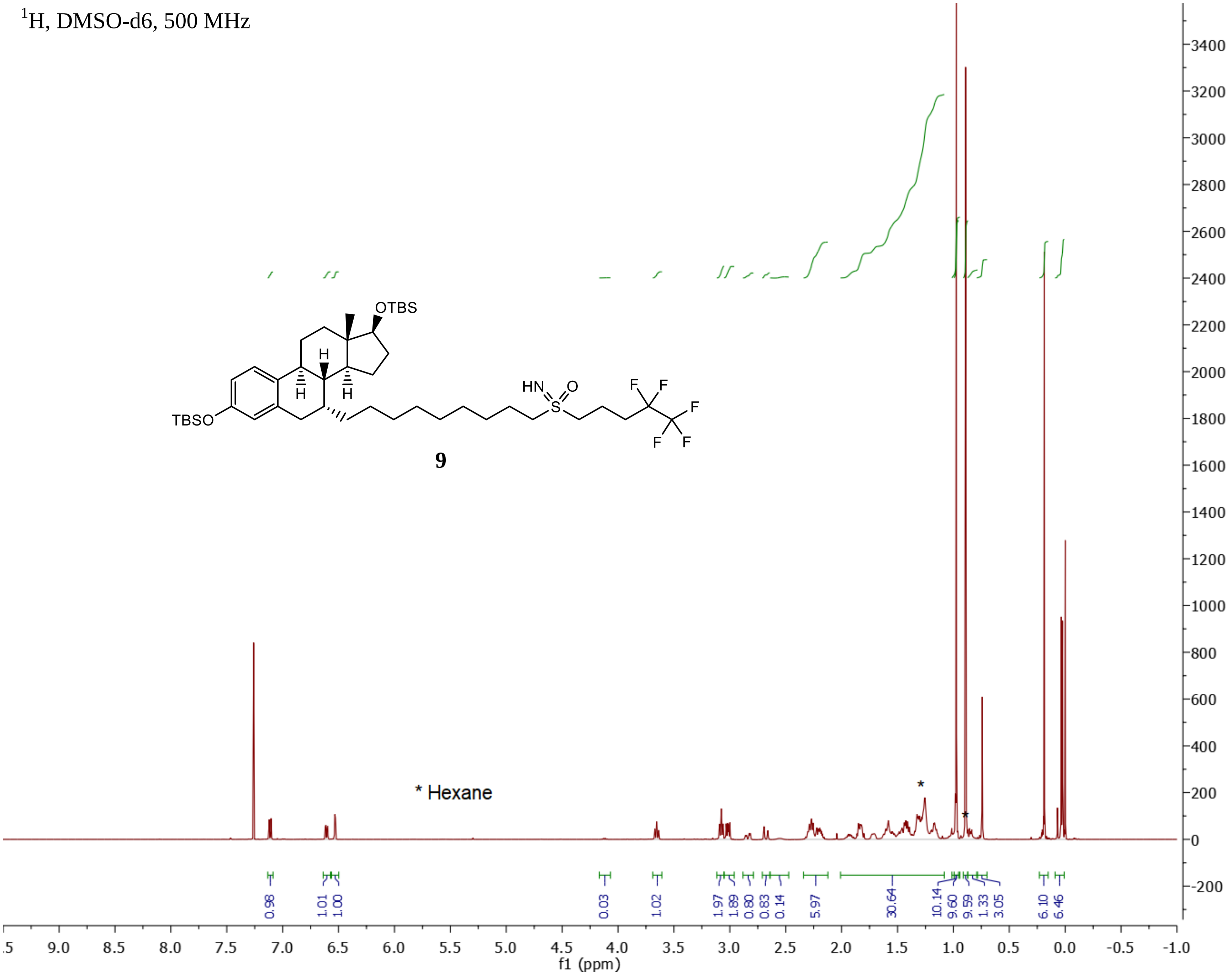

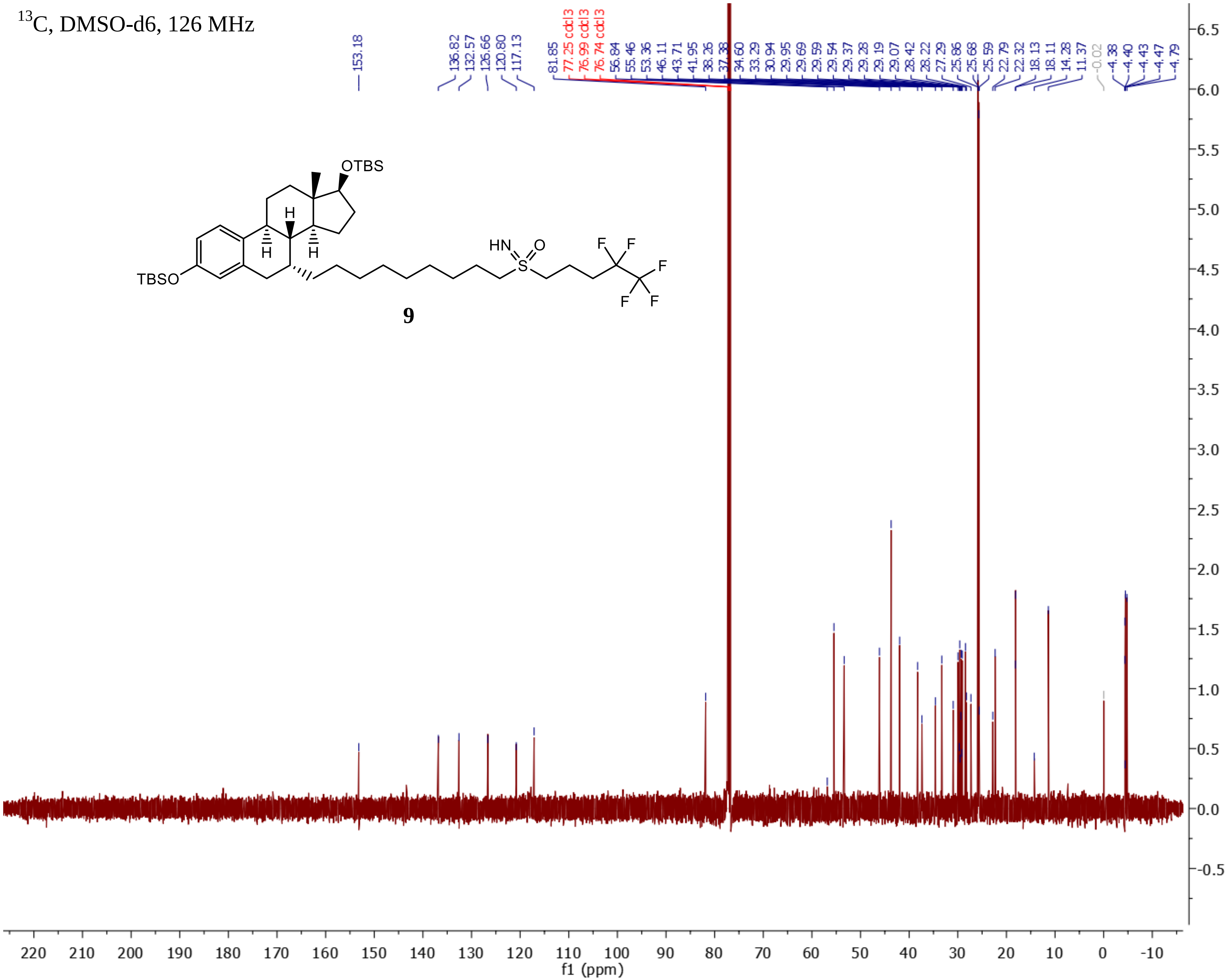

13C, DMSO-d6, 126 MHz
OTBS
TBSO
HN
O
S
F
9
153.18
136.82
132.57
126.66
120.80
117.13
81.85
77.25 cdcl3
76.99 cdcl3
76.74 cdcl3
56.84
55.46
53.36
46.11
43.71
41.95
38.26
37.38
34.60
33.29
30.94
29.95
29.69
29.59
29.54
29.37
29.28
29.19
29.07
28.42
28.22
27.29
25.86
25.68
25.59
22.79
22.32
18.13
18.11
14.28
11.37
-0.02
-4.38
-4.40
-4.43
-4.47
-4.79
f1 (ppm)

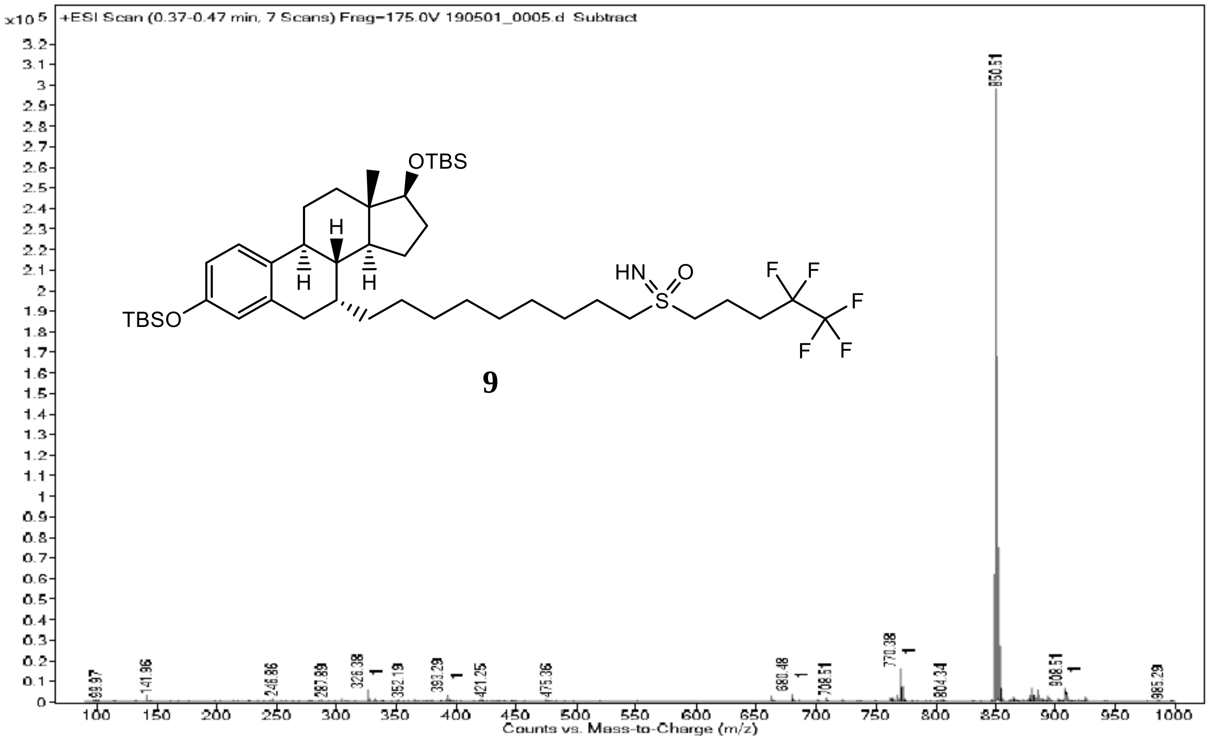

Target Ion Species

Ion Species	m/z	Ionic Formula
(M+H)+	850.5068	C44 H77 F5 N O3 S Si2

MFG Calculator Results

Target m/z	Ionic Formula	Calc m/z	+/- (mDa)	+/- (ppm)	DBE	MFG Score
850.5068	C38 H79 F3 N3 O8 S Si2	850.5073	-0.5	-0.6	2.0	99.20
850.5068	C36 H76 N9 O8 S Si2	850.5071	-0.3	-0.4	6.0	99.04
850.5068	C42 H74 F2 N7 O3 S Si2	850.5075	-0.7	-0.8	11.0	99.00
850.5068	C43 H76 N3 O10 Si2	850.5064	0.4	0.5	10.0	98.77
850.5068	C41 H78 F2 N3 O7 S Si2	850.5062	0.6	0.7	6.0	98.72
850.5068	C44 H77 F5 N O3 S Si2	850.5077	-0.9	-1.1	7.0	98.63
850.5068	C47 H76 F4 N O2 S Si2	850.5066	0.2	0.2	11.0	98.08
850.5068	C39 H72 F4 N7 O5 Si2	850.5064	0.4	0.5	8.0	97.88
850.5068	C45 H73 F N7 O2 S Si2	850.5064	0.4	0.5	15.0	97.65
850.5068	C39 H75 F3 N7 O4 S Si2	850.5086	-1.8	-2.1	7.0	97.28

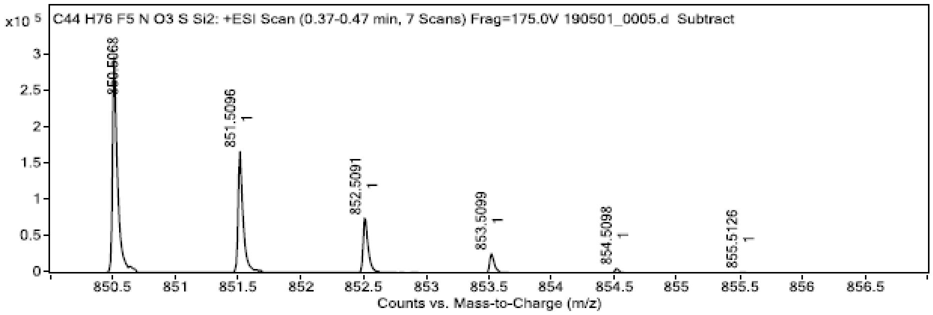

Predicted Isotope Match Table

Isotope	m/z	Calc m/z	Diff (mDa)	Abund (%)	Calc Abund (%)	+/-
1	850.5068	850.5077	-0.9	100.0	100.0	0.0
2	851.5096	851.5104	-0.8	56.4	59.9	3.5
3	852.5091	852.5097	-0.6	26.3	29.2	2.9
4	853.5099	853.5102	-0.3	9.0	10.0	1.0
5	854.5098	854.5102	-0.4	2.4	2.8	0.4
6	855.5126	855.5105	2.1	0.5	0.6	0.1
7	856.5131	856.5109	2.2	0.1	0.1	0.0

10

^1H, $CDCl_3$, 500 MHz

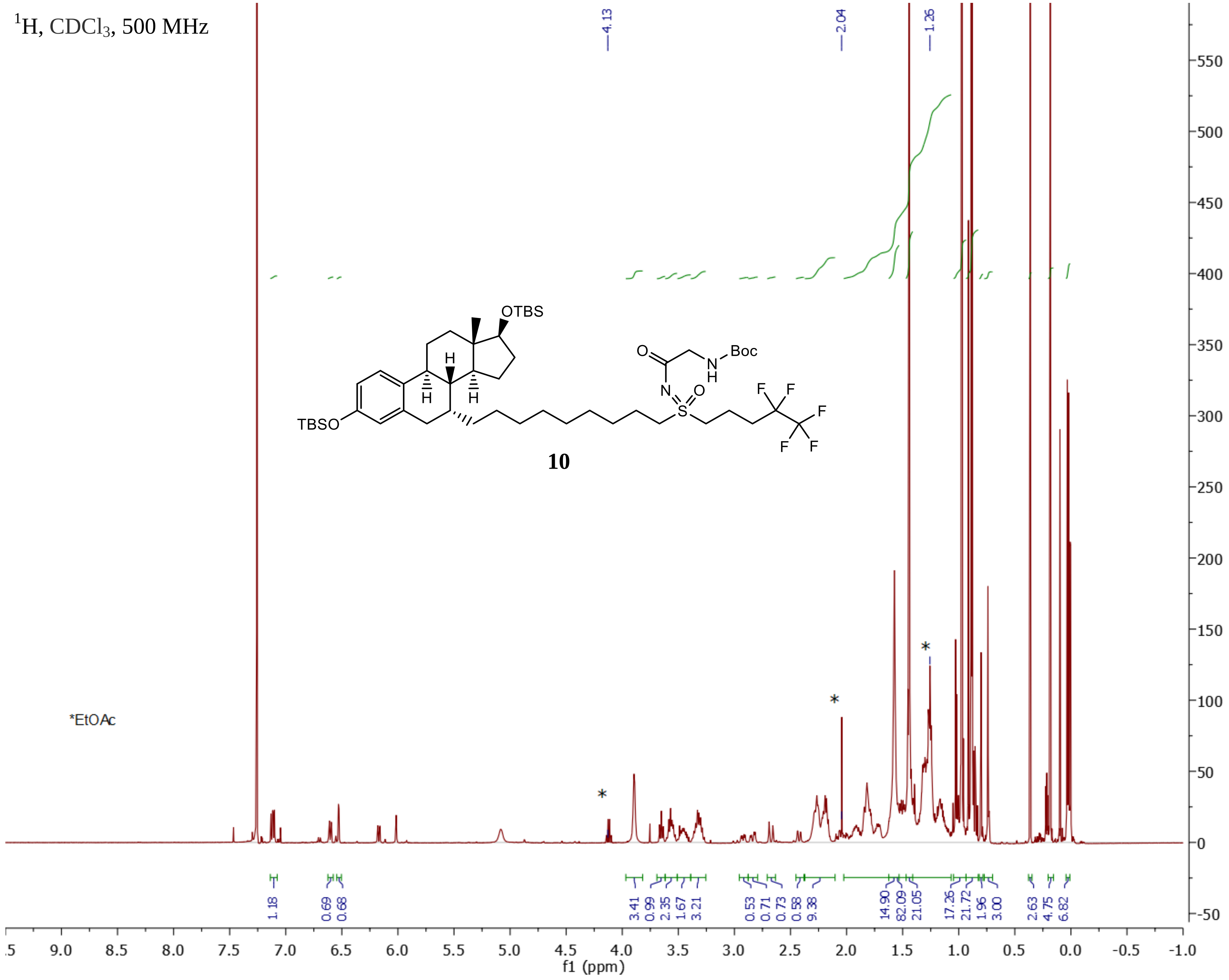

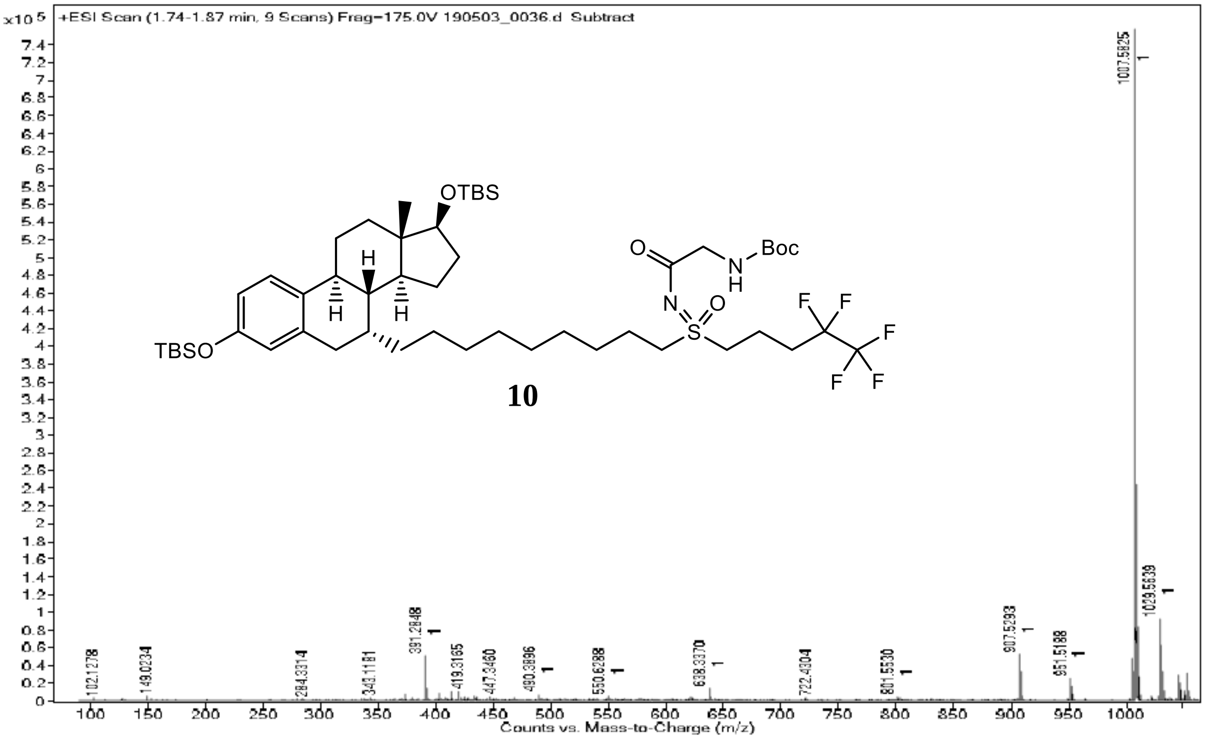

Target Ion Species

Ion Species	m/z	Ionic Formula
(M+H)+	1007.5825	C51 H88 F5 N2 O6 S Si2

MFG Calculator Results

Target m/z	Ionic Formula	Calc m/z	+/- (mDa)	+/- (ppm)	DBE	MFG Score
1007.5825	C52 H84 F5 N6 O2 S Si2	1007.5830	-0.5	-0.5	14.0	98.48
1007.5825	C59 H84 F5 O4 Si2	1007.5823	0.2	0.2	18.0	98.44
1007.5825	C51 H88 F5 N2 O6 S Si2	1007.5816	0.9	0.9	9.0	97.70
1007.5825	C43 H84 F5 N8 O9 Si2	1007.5814	1.1	1.1	6.0	96.95
1007.5825	C60 H80 F5 N4 Si2	1007.5836	-1.1	-1.1	23.0	95.83
1007.5825	C55 H80 F5 N6 O2 Si2	1007.5796	2.9	2.9	19.0	93.81
1007.5825	C40 H88 F5 N8 O9 S Si2	1007.5848	-2.3	-2.3	1.0	92.12
1007.5825	C48 H84 F5 N6 O7 Si2	1007.5855	-3.0	-3.0	10.0	91.89
1007.5825	C56 H88 F5 O4 S Si2	1007.5857	-3.2	-3.2	13.0	89.59
1007.5825	C54 H84 F5 N2 O6 Si2	1007.5783	4.2	4.2	14.0	88.69

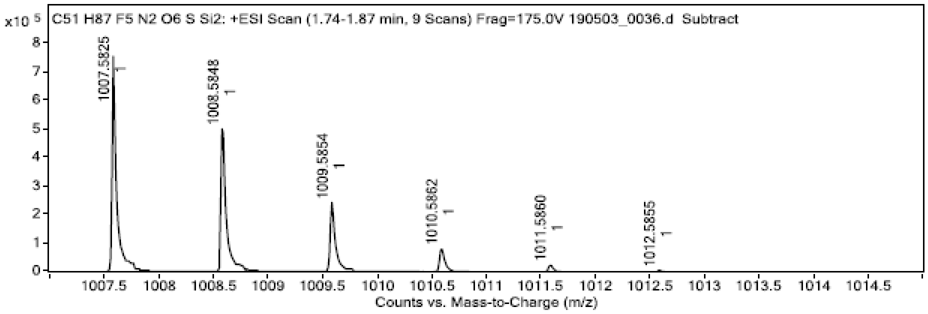

Predicted Isotope Match Table

Isotope	m/z	Calc m/z	Diff (mDa)	Abund (%)	Calc Abund (%)	+/-
1	1007.5825	1007.5816	0.9	100.0	100.0	0.0
2	1008.5848	1008.5844	0.4	68.7	68.1	-0.6
3	1009.5854	1009.5842	1.2	31.7	35.0	3.3
4	1010.5862	1010.5848	1.4	11.0	13.0	2.0
5	1011.5860	1011.5851	0.9	3.5	3.9	0.4

11

^{1}H, DMSO-d6, 500 MHz

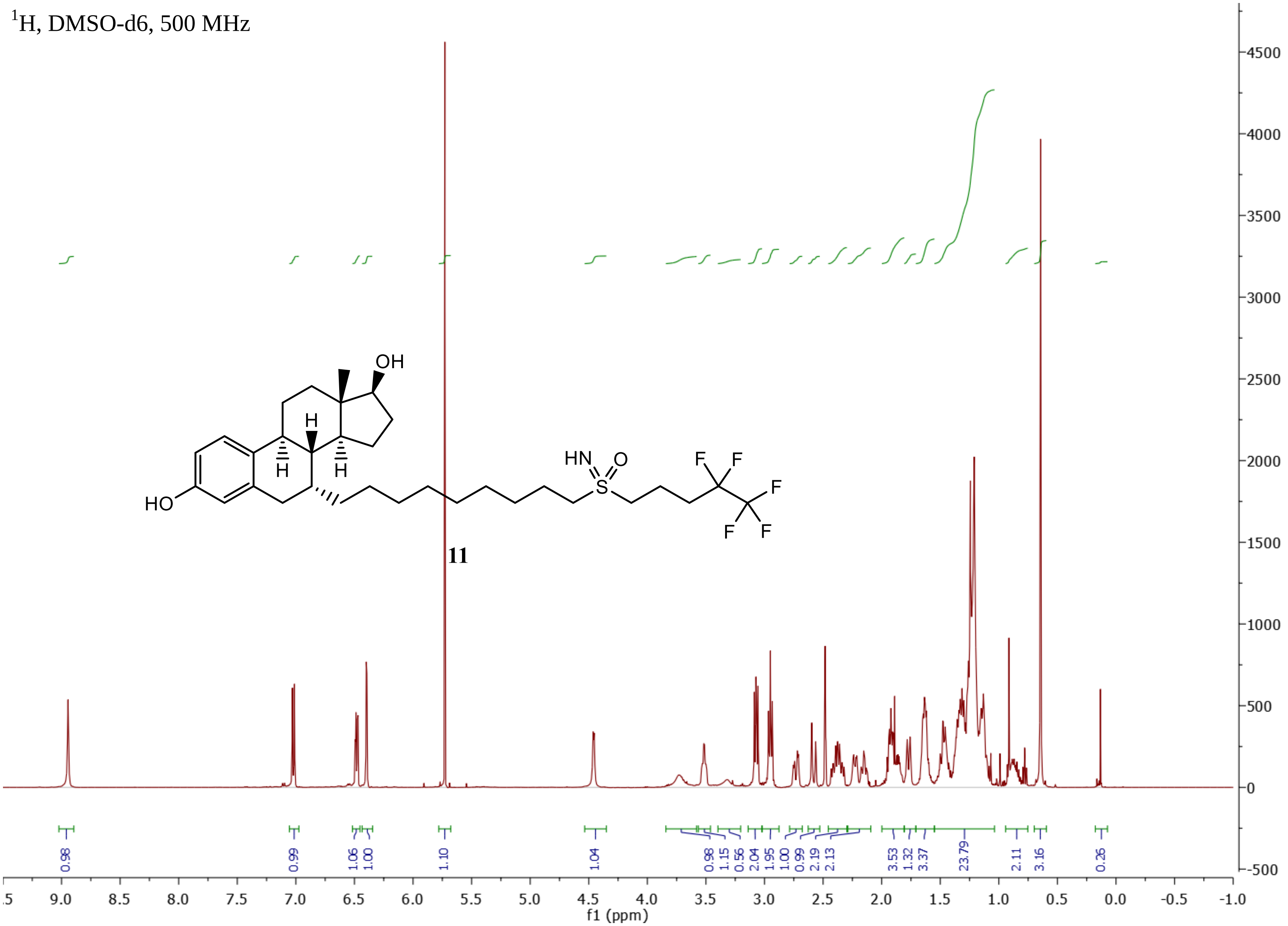

^{13}C, DMSO-d6, 126 MHz

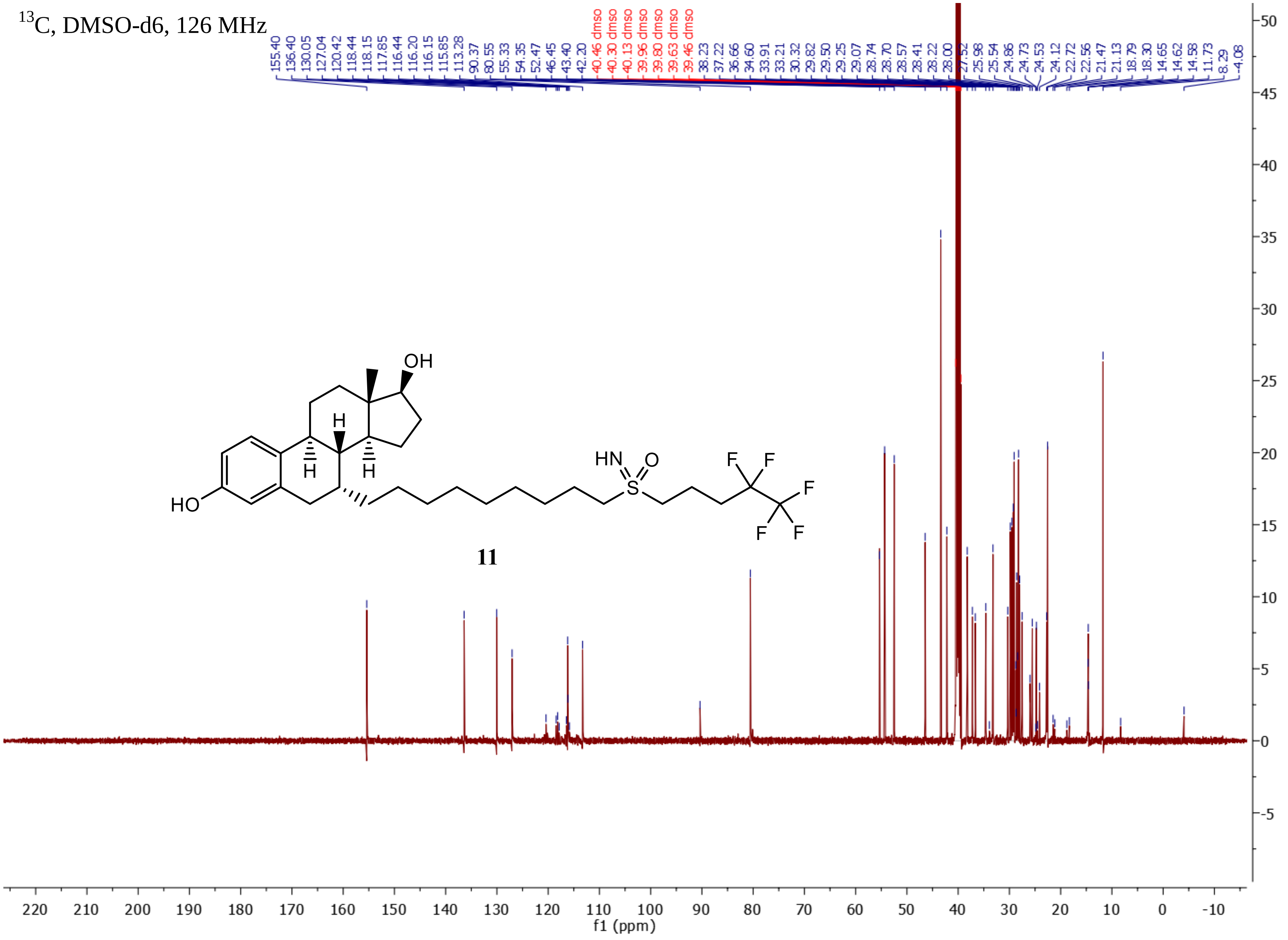

11

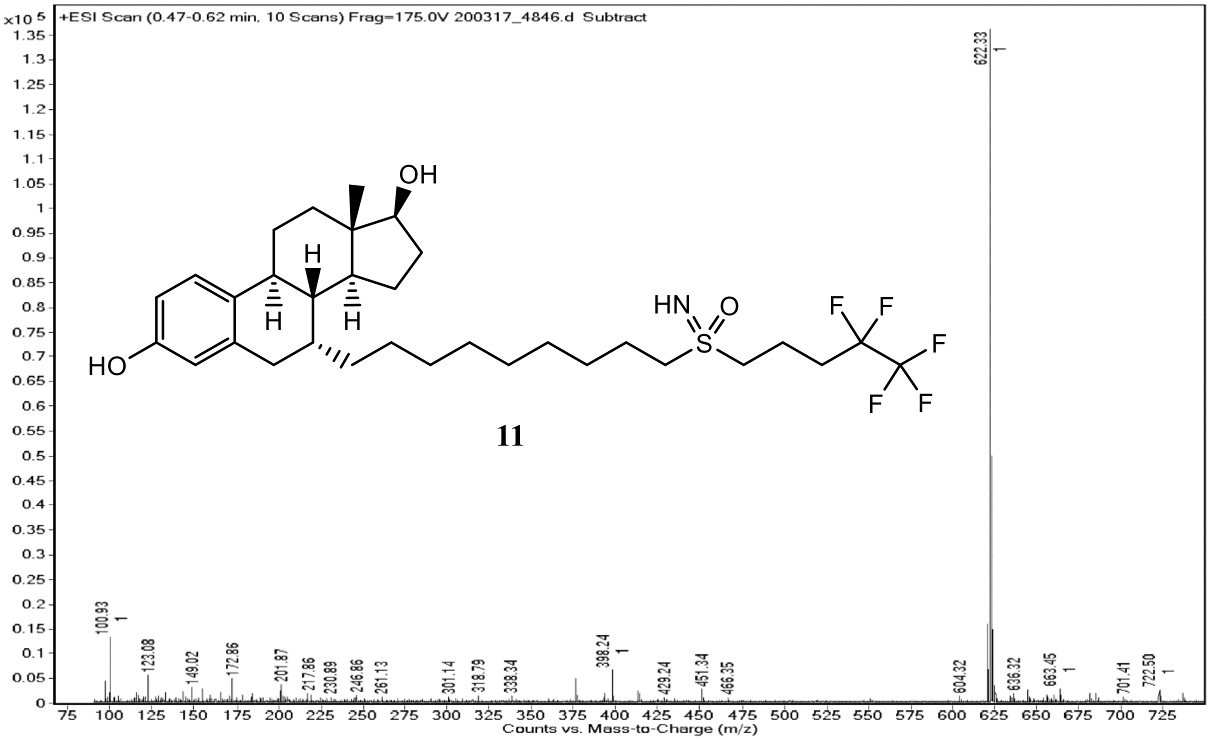

Target Ion Species

Ion Species	*m/z*	*Ionic Formula*
(M+H)+	622.3344	C32 H49 F5 N O3 S

MFG Calculator Results

Target m/z	*Ionic Formula*	*Calc m/z*	*+/- (mDa)*	*+/- (ppm)*	*DBE*	*MFG Score*
622.3344	C32 H49 F5 N O3 S	622.3348	-0.4	-0.6	7.0	99.38
622.3344	C24 H45 F5 N7 O6	622.3346	-0.2	-0.3	4.0	90.81
622.3344	C28 H45 F5 N7 O S	622.3321	2.3	3.7	8.0	90.00
622.3344	C35 H45 F5 N O3	622.3314	3.0	4.8	12.0	83.79
622.3344	C27 H49 F5 N3 O5 S	622.3308	3.6	5.8	3.0	79.71
622.3344	C28 H49 F5 N O8	622.3373	-2.9	-4.7	3.0	78.98
622.3344	C29 H45 F5 N5 O4	622.3386	-4.2	-6.7	8.0	70.42
622.3344	C19 H45 F5 N9 O8	622.3306	3.8	6.1	0.0	67.14

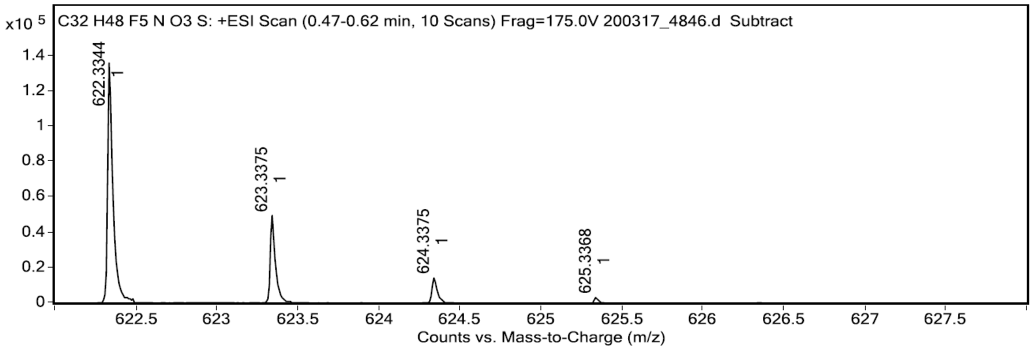

Predicted Isotope Match Table

Isotope	*m/z*	*Calc m/z*	Diff (mDa)	Abund (%)	Calc Abund (%)	+/-
1	622.3344	622.3348	-0.4	100.0	100.0	0.0
2	623.3375	623.3380	-0.5	35.9	36.4	0.5
3	624.3375	624.3370	0.5	10.7	11.5	0.8
4	625.3368	625.3377	-0.9	2.6	2.6	0.0
5	626.3351	626.3393	-4.2	0.5	0.4	-0.1

References

1. Donders EN, Ganesh AN, Torosyan H, Lak P, Shoichet BK, Shoichet MS. Triggered Release Enhances the Cytotoxicity of Stable Colloidal Drug Aggregates. *ACS Chem Biol*. 2019;14(7):1507-1514. doi:10.1021/acschembio.9b00247

2. Sirvent JA, Lücking U. Novel Pieces for the Emerging Picture of Sulfoximines in Drug Discovery: Synthesis and Evaluation of Sulfoximine Analogues of Marketed Drugs and Advanced Clinical Candidates. *ChemMedChem*. 2017;12(7):487-501. doi:10.1002/cmdc.201700044
